# Structural and ITC Characterization of Peptide‐Protein Binding: Thermodynamic Consequences of Cyclization Constraints, a Case Study on Vascular Endothelial Growth Factor Ligands

**DOI:** 10.1002/chem.202200465

**Published:** 2022-07-07

**Authors:** Jean‐François Gaucher, Marie Reille‐Seroussi, Sylvain Broussy

**Affiliations:** ^1^ CiTCoM UMR CNRS 8038 Université Paris Cité, Faculté de Santé, UFR de Pharmacie 4 av. de l'Observatoire 75006 Paris France; ^2^ CitCoM UMR CNRS 8038 U1268 INSERM Université Paris Cité, Faculté de Santé, UFR de Pharmacie 4 av. de l'Observatoire 75006 Paris France

**Keywords:** drug design, helix stabilization, peptide folding thermodynamics, vibrational entropy

## Abstract

Macrocyclization constraints are widely used in the design of protein ligands to stabilize their bioactive conformation and increase their affinities. However, the resulting changes in binding entropy can be puzzling and uncorrelated to affinity gains. Here, the thermodynamic (Isothermal Titration Calorimetry) and structural (X‐ray, NMR and CD) analysis of a complete series of lactam‐bridged peptide ligands of the vascular endothelial growth factor, and their unconstrained analogs are reported. It is shown that differences in thermodynamics arise mainly from the folding energy of the peptide upon binding. The systematic reduction in conformational entropy penalty due to helix pre‐organization can be counterbalanced by an unfavorable vibrational entropy change if the constraints are too rigid. The gain in configurational entropy partially escapes the enthalpy/entropy compensation and leads to an improvement in affinity. The precision of the analytical ITC method makes this study a possible benchmark for constrained peptides optimization.

## Graphical abstract

**Does affinity come from constraint ?** The thermodynamic and structural analysis of a series of constrained peptide ligands and unconstrained analogues accurately describes the binding process. It highlights how the variation in conformational and vibrational entropies associated with the constraints impacts the affinity.

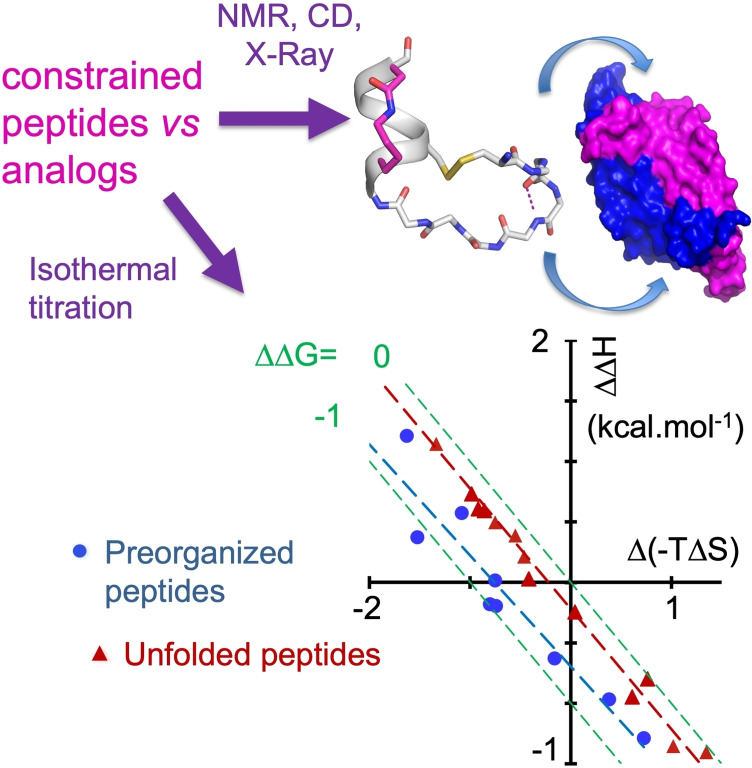

## Introduction

Peptides have gained increased interest as pharmaceuticals. They share many strengths, such as high target selectivity, good efficiency, safety, and tolerability. However, a short plasma half‐life, chemical and physical instabilities and low membrane permeability are weaknesses that must be overcome for therapeutic use. Among the possible strategies, a common approach is to constrain peptides, which can reduce susceptibility to proteolysis, stabilize their bioactive form and improve both affinity and specificity. However, attempting to correlate constraints to binding thermodynamics is puzzling, if not impossible. While a gain in binding affinity is often observed, inconsistencies in change of entropy and enthalpy upon binding are also noted,^[1]^ making interpretation of thermodynamics difficult. In thermodynamical analysis, Isothermal Titration Calorimetry (ITC) is considered as a golden standard because it measures unlabeled partner interactions with high precision, but also because it is the only experimental method capable of simultaneously measuring the enthalpy of binding and the Gibbs free energy, and therefore entropy. However, several points raise issues, such as imprecisions resulting from non‐analytical titration methods or underestimation of measurements uncertainty, lack of structural information, poor understanding of the binding process, involvement of the constrained chemical functions in the binding site and the role of solvation. The generally observed opposite variation in enthalpy and entropy of binding upon constraining the ligand, the so‐called Enthalpy/Entropy Compensation (EEC), is also a subject of debate.^[2]^ This established physical property^[3]^ is sometimes seen as a phantom phenomenon related to artifacts of measurements.^[4]^ Therefore a structure–activity relationship study of ligand–receptor binding, combining analytical quantification of thermodynamics and complete structural data of related ligands and receptors, is a recurring request from the scientific community:[^1b^, ^4b^, ^5^] It would make it possible, through a concrete example, to review the relevance of models for the prediction of affinity or for the design of ligands. Such a study questions the validity of thermodynamic‐guided approaches and sheds light on the relevant parameters. Finally, it examines the practical utility of reducing the conformational space of ligands to increase their affinity.

Consequently, we used an appropriate peptide‐protein interaction as a model to precisely decipher the binding process of closely related peptides, using analytical ITC and structure determination in an experimental approach. In the present work, we focus on a class of monocyclic disulfide‐containing 19 amino acid peptides targeting the Vascular Endothelial Growth Factor‐A, the main pro‐angiogenic factor in Human.^[6]^ The prototype peptides, v107 and v114*, were identified by phage display.^[7]^ The structure of v107 bound to VEGF_11‐109_ in solution was determined by NMR,^[8]^ and its dynamic of binding was explored by molecular dynamics simulations.^[9]^ A minimal sequence was later defined, consisting in 15 amino acids peptide molecules and dissociation constants in the range of 100 nM at 20 °C.^[10]^ We assumed that it must be possible to gain affinity by stabilizing the peptide structure observed in the bound state through pre‐folding of the C‐terminal part into the bioactive α‐helical conformation. We proposed to use this peptide as a model to rationalize the thermodynamics of α‐helix stabilization by an i to i+4 lactam bridge. The modified residues were chosen not to be directly involved in the binding site, to isolate the folding entropy contribution. We systematically compared KxxxD/E and D/ExxxK substitution series, bridged or not, and measured their thermodynamics by ITC. Next, we determined the unbound and bound structures of both peptides and proteins by circular dichroism, NMR spectrometry, and X‐ray crystallography. These studies provide precise thermodynamic data for a series of structurally well‐characterized and closely related protein–ligand complexes, for the use by the computational community who needs reliable dataset for theoretical prediction of enthalpies and entropies of binding.^[5c]^

The structure‐activity relationship demonstrated that neither the protein nor the peptide binds in a conformation corresponding to its energy minimum, and that the peptide must fold upon binding. We proposed that the main affinity differences across the series arose from the folding energy of the peptide. Moreover, we deciphered the effect of the amino acid sequence on the measured counterbalancing changes of binding enthalpy and entropy. Seeming inconsistencies of the effect of the lactam bridge on binding entropies are discussed, involving the role of vibrational entropy variation. Finally, we have shown that, for peptides pre‐folded into helices, a part of the conformational entropy gain escaped EEC, and was converted into favorable free energy of binding.

## Results and Discussion

### Structures of unbound peptides

#### Peptide synthesis

**Figure 1:**
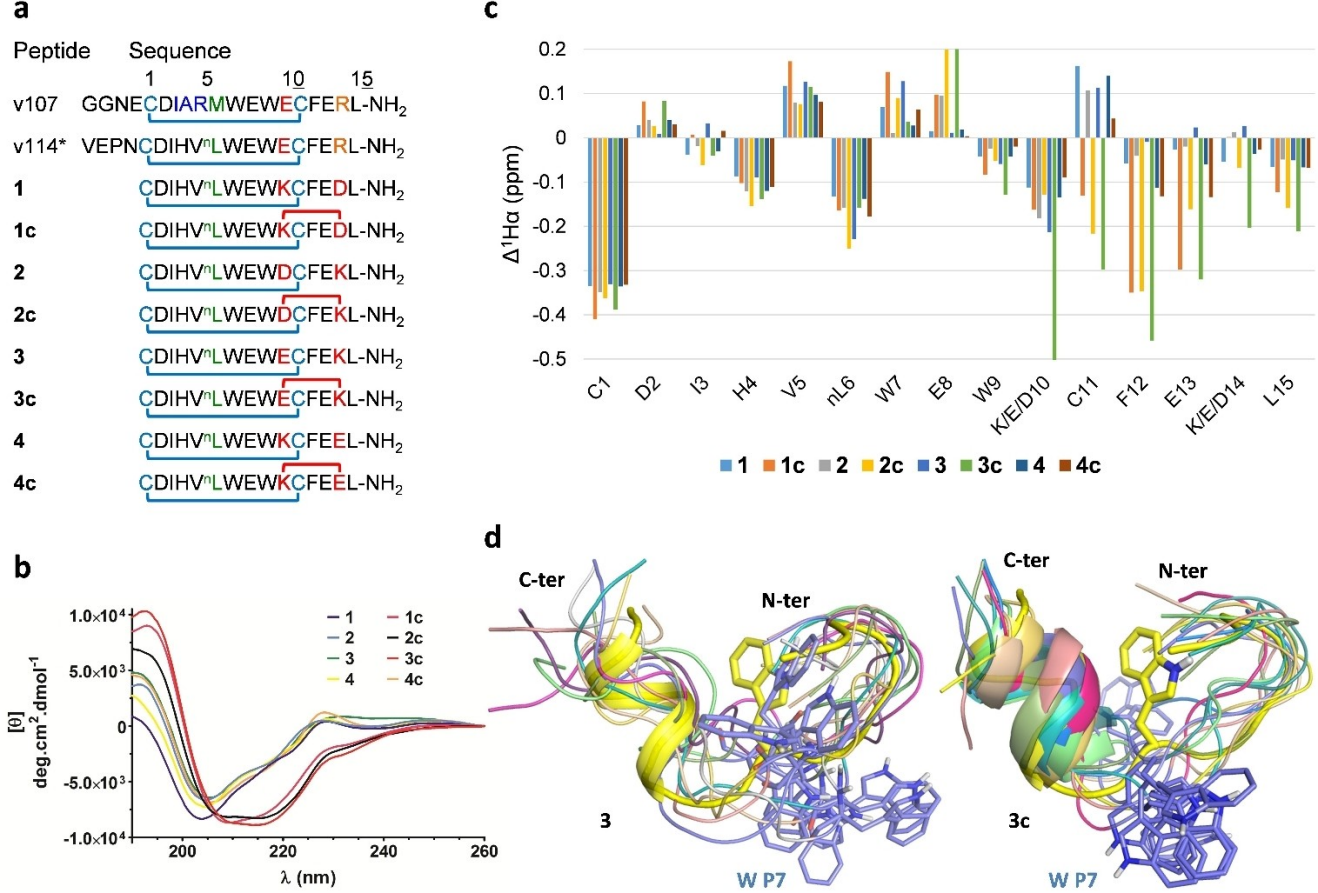
Design and spectroscopic characterization of unbound monocyclic peptides **1–4** and bicyclic peptides **1 c**–**4 c**. a) Sequences of the peptides, ^n^L denotes norleucine, blue lines represent disulfide bonds, and red lines lactam bonds formed between side chains of residues in i to i+4 positions. b) CD spectra in 10 mM phosphate buffer pH 7.4 at 25 °C. c) Chemical shift index (CSI) calculated as the deviation from random coil values. d) Superposition of the 10 lowest energy NMR structures of peptides **3** (left) and **3 c** (right) free in solution (thin ribbons) to the crystal structure of **3 c** bound to VEGF (thick yellow ribbon). Backbone atoms of residues P2‐5 and P10‐14 were used for the superposition. Trp P7 is highlighted in yellow in the crystal structure and in slate blue in the NMR structures, showing their different orientations. The C‐terminal segment is folded into an α‐helix in **3 c** but not in **3**.

Monocyclic peptides and bicyclic analogs (Figure 1a) were synthesized on solid phase with Rink amide resin, with standard Fmoc protected amino acids. The disulfide bonds were formed in solution after cleavage of the peptide from the resin and side chain deprotection, under basic pH and diluted conditions in aqueous solution. For the bicyclic peptides **1 c**, **2 c**, **3 c** and **4 c**, the lactam cyclization was achieved on resin with PyBOP/DIEA conditions, after deprotection of the orthogonal protecting groups phenylisopropyl (Pip) for aspartate and glutamate, and methyltrityl (Mtt) for lysine (Figure S1). The peptides were purified by semi‐preparative HPLC and characterized by mass spectroscopy, CD, NMR and analytical HPLC, demonstrating a purity greater than 99 %.

#### CD showed that unbound bicyclic peptides 1 c, 2 c and 3 c were α‐helically folded

The helicity of short α‐helical peptides is characterized by a positive maximum of molar ellipticity ([*θ*]) at 190 nm and a minimum at 207 nm which is similar to longer helices in proteins with maximum at 190 nm and minimum at 208 nm. A shift is also observed in the wavelength of the additional minimum at 215 nm, versus 222 nm for longer helices.^[11]^ In the current series, the spectra of bicyclic peptides **1 c**, **2 c** and **3 c** showed such a characteristic shape of α‐helix: they had a positive maximum shifted to 192 nm, a negative band with two minima at 209 nm and 215 nm, and a negative shoulder around 228 nm (Figure 1b). On the contrary, the monocyclic peptides **1**, **2**, **3** and **4**, and the peptide **4 c** had little secondary structure: they displayed a weak maximum at 190 nm, a negative minimum at 204–205 nm followed by a positive slope reaching zero at around 225 nm and forming a small positive peak around 228 nm. The α‐helicity was estimated by the values of [*θ*]_215_ and the 215 nm/207 nm ellipticity ratio (Table S1).^[11]^ Bicyclic peptides **1 c**, **2 c** and **3 c** had higher [*θ*]_215_ than the other peptides and ratios closer to 1, indicating that their C‐terminal α‐helices were mostly structured in unbound form.

#### NMR data evidenced the helical folding of C‐terminal helix of 1 c, 2 c and 3 c peptides

The complete peptide series was analyzed by 1D and 2D ^1^H NMR spectroscopy in aqueous solutions. ^1^H NMR signals in all spectra were sharp and well‐defined, indicating the absence of slowly interconverting conformers. However, fast exchanging conformers were expected, because small peptides, even cyclic ones, are usually present as multiple conformations. All backbone protons and most relevant side chains protons were assigned with Topspin and CCP NMR programs based on TOCSY and NOESY spectra.

The negative chemical shift index (CSI) values of the Hα protons of the bicyclic peptides **1 c**, **2 c**, and **3 c** confirmed the presence of α‐helical structure in the C‐terminal segment (residues P10‐15, Figure 1c), whereas CSI of the monocyclic and **4 c** peptides indicated random structures (the P labeling of amino acids refers to the peptide chain, and V and W to the VEGF chains interacting with the peptide).^[12]^ However, the CSI values of the Hα protons in the N‐terminal part (residues P1‐8) of all 8 peptides were close, with negative values for Cys P1, His P4 and ^n^Leu P6. This indicated that their secondary structures in the N‐terminal part were similar and unaffected by the differences in the C‐terminal part.

Variation of NH chemical shifts signals as a function of temperature showed that the bicyclic peptides **1 c**, **2 c**, and **3 c** had overall more hydrogen atoms involved in stable H‐bonds than the other peptides, as demonstrated by smaller |*Δ*δ/°C| values (Table S2).^[13]^ In contrast, the bicyclic peptide **4 c** appeared very similar to the monocyclic peptide **4**. Some values of *Δ*δ/°C close to 0 or even positive were observed for **1 c** and **3 c**, indicating the existence of very strong H‐bonds at the N‐terminal β‐turn (Val P5) and for some residues of the C‐terminal α‐helix (Cys P11 and Leu P15). No H‐bond was detected for the Lys P10/P14 NHζ of the lactam bridge of the bicyclic peptides, indicating that it did not interact with the backbone of the peptides. The flexibility of the lactam linkers was evaluated by measuring the differences in chemical shift (*Δ*δ in ppm) between the diastereotopic protons of the side chains involved in position P10 and P14 (Table S3). In the bicyclic peptides **1 c**, **2 c** and **3 c**, significant chemical shift differences were observed in Asp and Glu CH_2_β and Lys CH_2_ϵ, which evidenced inhomogeneity in the environment of these protons resulting from hindered rotation: for **1 c** and **3 c**, large *Δ*δ values up to 0.8 to 1.0 ppm were observed at position P10 and P14 whereas for **4 c**, smaller values of 0.2 ppm were measured. Peptide **2 c** had intermediate *Δ*δ values, which could be interpreted as intermediate flexibility of the lactam Asp P10 – Lys P14 linker. In the NMR spectra of peptide **1 c**, the linker amide NH resonance was also split into a broad doublet of doublets, whereas for **2 c** it was a broad triplet, in agreement with the observation by Hoang et al., who proposed specific n→π* stabilizing interaction in a KxxxD lactam‐bridged peptide.^[14]^ The corresponding NH resonances were not observable in **3 c** and **4 c** because of signal overlap.

Finally, the structures of the unbound peptides were determined by simulated annealing with XPLOR‐NIH with NOE constraints. The 2D NMR spectra of bicyclic peptides showed spectral dispersion suggesting that the peptides were partially ordered in solution. However, the NMR structure calculations did not converge into a single conformation. The backbone RMSD of the 20 lowest energy NMR structures, compared to the co‐crystal structures of bicyclic peptides and VEGF (see below), indicated that the bicyclic **2 c** and **3 c** were significantly closer to the folded structures than were the other peptides (Table S4). In particular, the RMSD values in the α‐helix segment P10‐14 were extremely low for these bicyclic peptides (1.1 and 0.6 Å), showing their similarity with the crystal structure (see the supplementary NMR discussion for a more detailed description, including the β‐turn P2‐5 and extended fragment P6‐9). The clustering of the 20 lowest energy NMR structures obtained for each peptide showed that helices (amino acids P10‐14) of bicyclic peptides **1 c**, **2 c** and **3 c** were stabilized, compared to monocyclic control peptides (Table S5). Helical folds were observed in most structures for **2 c** and **3 c**, resulting in a single α‐helical cluster for a RMSD cutoff of 1.2 Å. At this value, **1 c** had 6 clusters, showing a larger flexibility in this segment than **2 c** and **3 c**, whereas **4 c** and the monocyclic peptides had between 9 and 16 clusters. The β‐turn conformation (amino acids P2‐5) was overall well conserved among all peptides, with the presence of conformations compatible with the type I β‐turn present in the crystal structure. The fragment in‐between (amino acids P6‐9) was in extended conformation, with the indole of Trp P7 consistently oriented toward the solvent, in a remarkably different orientation than in the VEGF‐bound structures (Figure 1d, see the supplementary NMR discussion for a more detailed description). Peptide v107 was studied by Fairbrother et al. by NMR spectroscopy under similar conditions of concentration, pH and temperature as our experiments, and can be compared with the monocyclic peptides.^[8]^ The peptide v107 has a limited spectral dispersion, which is a first indication of its disordered nature. Indeed, its structure could not be determined because NOE cross‐peaks gave rise to serious spectral overlaps and significant restraints violations in distance geometry calculations. In addition, according to spectra recorded at 10 °C and 20 °C, the region corresponding to P8‐10 is involved in a conformational exchange process. The authors concluded that v107 is poorly structured, although some α‐helical character is suspected at the C‐terminal part, and that it populates multiple exchanging conformations in solution.[^7a^, ^8^, ^15^]

Overall, the NMR data of the free peptides in solution indicated that they did not exist as a single conformation. The monocyclic peptides were similar to the peptide v107. A β‐turn at the N‐terminal end was observed in all the peptides with small variability. Inversely, the α‐helix at the C‐terminal end was stabilized by the presence of the lactam bridge in the bicyclic peptides **1 c**, **2 c** and **3 c** and not in the five other peptides, in agreement with CD data. The extended conformation of the P6‐9 residues was flexible for all the peptides, as in v107, and exposed the indole side chain of Trp P7 toward the solvent.

#### Crystal structures of bicyclic peptides‐VEGF complexes

The VEGF_11‐109_ homodimer was crystallized in the presence of peptide **1 c**, **2 c**, **3 c** or **4 c**, and the structures were refined at 1.8 Å, 2.1 Å, 1.8 Å and 1.6 Å respectively (PDB ID: 6ZCD, 6Z3F, 6Z13, 6ZBR) (Table S6). The asymmetric units contained a VEGF protein in which one of the two symetrical VEGF binding sites was partially occupied by a peptide molecule, the other site being inaccessible by steric hindrance. VEGF‐peptide **2 c** mixture crystallized also in a tetragonal form that contained no bound peptide. Its structure was refined to 1.6 Å to explore the effect of MPD used for crystallization (PDB ID 6ZFL). All the refined VEGF structures were close to the crystal structures previously solved in the presence of the VEGF receptor 1 domain 2 (PDB ID 1FLT)^[16]^ or in its absence (PDB ID 2VPF)^[17]^ (rmsd(Cα)=1.1 to 1.4 Å). The main backbone displacements occurred in the 83–89 and 35–46 loops, that are flexible in molecular modelling studies as well.^[9]^ Consequently, the MPD used for crystallizing did not result in any significant structural change, but probably weakened the affinity of peptides for VEGF.

#### The four peptides adopted conformations close to the monocyclic v107 peptide:VEGF NMR structure (PDB ID 1KAT)^[8]^

**Figure 2:**
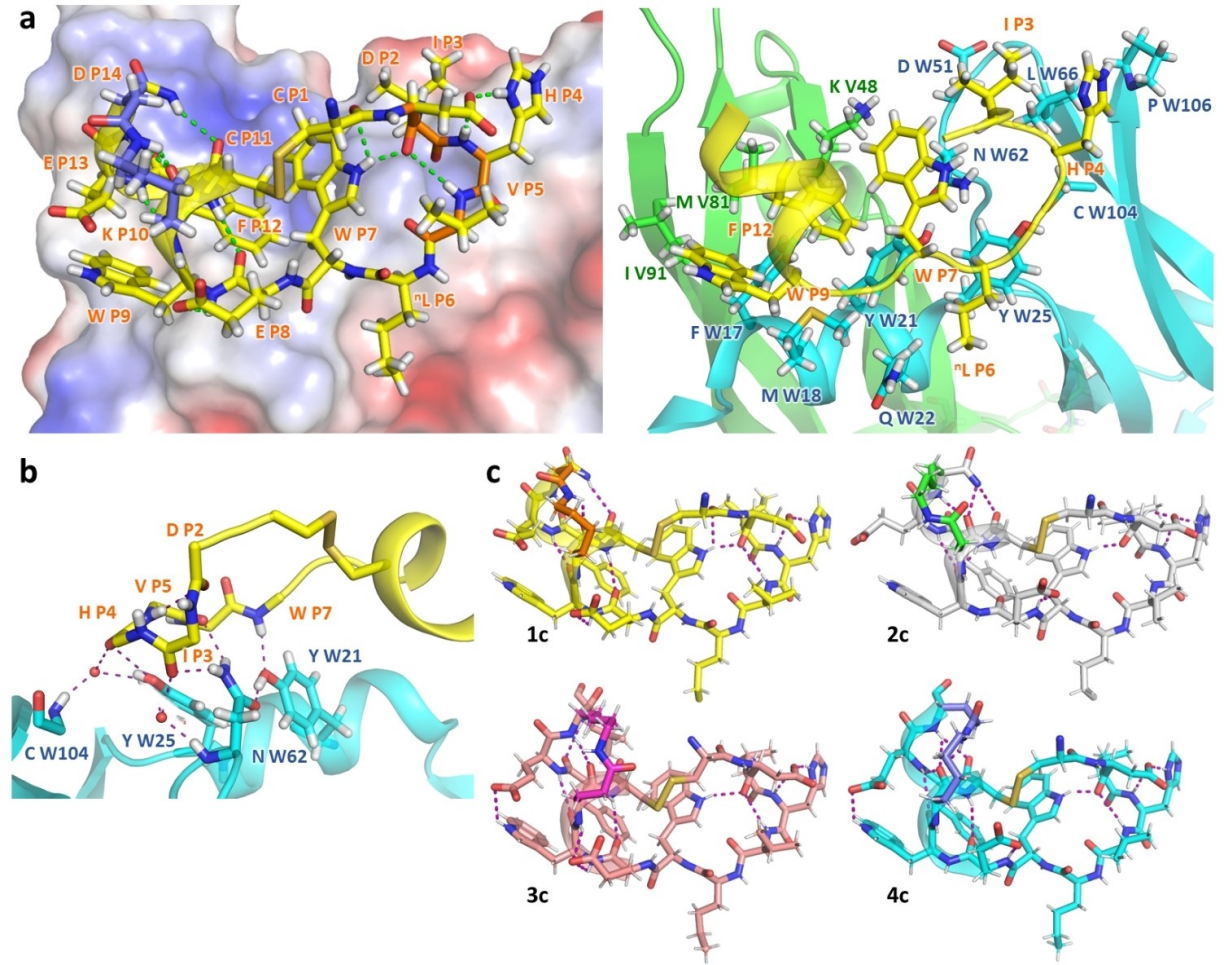
X‐ray structural characterization of VEGF‐bound bicyclic peptides. The labeling of amino acids P, V and W refers to the peptide and to the VEGF V and W chains, respectively. a) left: structure of **1 c** on the surface of VEGF colored as a function of the electrostatic potential. The β‐turn is drawn in orange, the two amino acids involved in the lactam bridge in blue and the intramolecular H‐bonds in green. Right: peptide binding site on the VEGF surface. Note the T‐shape π‐stacking of the Phe P12 with Trp P7, Phe W17 and Tyr W21. b) H‐bonding network between VEGF chain W and peptide **1 c**. The W61‐W62 peptide bond flips upon binding, allowing the Asn W62 amide group to orient toward the peptide backbone. Two strongly H‐bound water molecules are indicated as red spheres. c), Refined crystal structures of the four bicyclic peptides bound to VEGF. The lactam bridges are in bright colors. The H‐bond network is noted in purple dashes.

The residues P2‐5 formed a distorted type‐I β‐turn, followed by an extended fragment (P6‐8). The residues P9‐14 folded as a helix and the C‐terminus Leu P15 was not defined in the electronic density. The Cys P11 was disulfide bound with Cys P1. The amino acids P1 to P11 wrapped around the indole side chain of Trp P7 that was tightly packed against Ile P3 and Phe P12 side chains and against the disulfide bridge (Figure 2a). Moreover, O Asp P2 formed a further H‐bond with HNϵ Trp P7 contributing to the stability of the indole ring orientation.

#### The peptides:VEGF binding site was composed of two distinct surfaces, apolar and polar

Around 950 Å^2^ of accessible surface area (ASA) was buried at the peptide‐VEGF interface, that overlapped with the VEGF receptor 1 domain 2 (VEGFR1‐d2)‐binding site (Figure S2).^[16]^ On the apolar surface, the VEGF residues Met W18, Ile V91, Met V81, Phe W17, Tyr W21, Tyr W25, Leu W66 and a part of the Lys V48 side chain formed a large and relatively flat hydrophobic binding site (buried ASA: 700 Å^2^), in which the peptides residues Trp P9, Phe P12, Trp P7 and Ile P3 were bound. A remarkable feature at the heart of the site was the orthogonal T‐shaped π‐stacking of Phe P12 with Trp P7, Phe W17 and Tyr W21, the Phe W17 and Tyr W21 themselves being in a parallel‐displaced π‐stacking (Figures 2a and S3). The ^n^Leu P6 contributed to binding by apolar contacts with the side chain of Gln W22 and Tyr W25. In the complementary polar surface three short H‐bonds between VEGF residues and the peptide backbone near the β‐turn contributed to the peptide specificity for the site: Asn ND2 W62 H‐bound to O Ile P3 (d=2.2 Å) and to O Val P5 (d=2.3 Å), and OH Tyr W21 bound to NH Trp P7 (d=2.2 Å). Two conserved bound water molecules contributed also to the peptide binding by relaying H‐bonds from O Ile P3 carbonyl (d=2.8 Å) and O His P4 (d=2.8 Å) to VEGF (Figure 2b). They were also present in the unbound VEGF structures (PDB ID 6ZFL) and in the VEGF:VEGFR1‐d2 interface. The amino acids of the VEGF backbone that were involved in the interaction with the four peptide molecules remained unchanged as compared to unbound VEGF (rmsd_main chain_=0.45±0.09 Å), with the notable exception of W61‐62 peptide‐bond, which flipped with respect to the apo‐structures of VEGF (PDB ID 6ZFL, 2VFP) in the presence of the peptide.

The Asn W62 side chain moved 4.6 Å to pack against Trp P7 and H‐bind to OH Tyr W21, forming the above‐mentioned tight H‐bond network with the peptide backbone.

In all four bicyclic peptide structures the C‐terminus backbones folded in helices with higher B‐factor than the rest of the main chains, reflecting greater thermal motion (Figures 2c and S4). The real space correlation coefficient (RSCC, see Table S6) of the α‐helix was higher than 0.83 on VEGF:**1 c**, VEGF:**2 c** and VEGF:**3 c** structures, which supported these models. In contrast, the helix of peptide **4 c** was poorly defined in the electronic density (RSCC=0.76). Helix residues Trp P9 and Phe P12 were buried in the binding site and Cys P11 was linked to the N‐term Cys P1. The solvent‐facing side chains (Glu P8, Glu P13 and Leu P15) had high thermal factors, as did the lactam bridges.

In particular, the Cγ and Cδ of the Lys P10 or P14 were agitated. This suggested that, even in crystal state, lactam bridges can adopt several conformations in dynamical exchange. Interestingly the lactam bridge cyclization caused the side chains of residues P10 and P14 for peptide **1 c** and **3 c** to be in a non standard rotamer conformation,^[18]^ the consequence of which was moderate steric clashes involving hydrogen atoms. In the **4 c** peptide, the lactam bridge cyclization forced Lys P14 to rotate along phi angle (ϕ_Lys,P14_=−47°), which prevented any folding of the P15 residue into the helix. No steric conflict was observed on peptide **2 c**. Constraints caused by the lactam bridges and by the binding of the macro‐cyclized peptide to the VEGF site also made the α‐helices non‐canonical: in each of the four peptides, the residues P8‐11 folded into a 3_10_‐helix characterized by a H‐bond between Glu P8 O and Cys P11 HN. For the peptides **1 c** and **2 c** (KxxxD and DxxxK), the DSSP software^[19]^ identified residues P9‐14 as an α‐helix, although Trp P9 O could bind to both Phe P12 HN and Glu P13 HN. In the peptide **3 c** (ExxxK) the α‐helix extended from residues P9 to P12 followed by a 3_10_ turn where Phe P12 O was H‐bound to Leu P15 HN. The helix of peptide **4 c** (KxxxE) was longer (residues P8 to P14) with a H‐bond network similar to peptide **2 c** (Figure S4). In peptide **1 c**, we also observed that the conformation of the lactam was compatible with a n→π* stabilizing interaction involving the linker amide carbonyl π* orbital and the Lys P10 main chain carbonyl n orbital, which was mentioned above (Figure S5).^[14]^

The monocyclic peptides were not co‐crystallized, but the v107 peptide:VEGF NMR structure can be used as a model.^[8]^ Despite some sequence differences, all the peptide‐protein contacts are equivalent (see Supporting Information for details), except for ^n^Leu 6, which is substituted by a Met in v107. The sequence of the four residues involved in the β‐turn, ‐DIHV‐ in the current bicyclic series, is ‐DIAR‐ in the v107 peptide, and the residues corresponding to the lactam bridge in α‐helix are Glu (i) and Arg (i+4). The main RMSD values between the amino acids homologous to the bicyclic‐peptides and to the 24 best NMR structures of v107 were calculated. It showed that the v107 backbone was close to all bicyclic structures (< RMSD_MainChain1‐14_>=1.01±0.07 Å) (Figure S6). However, unlike bicyclic peptides helices that began by a 3_10_ turn, the v107 peptide helix formed a regular α‐helix terminated by a 3_10_ turn relaying atoms equivalent to O Phe P12 and HN Leu P15. This resulted in the offset of the α‐helix axis of approximately 10° relative to the bicyclic peptides.

Overall, the crystal structures of bicyclic peptides bound to VEGF brought out that all four bicyclic peptides adopted structures close to the v107 peptide. In all the peptides, the amino acids in positions 10 and 14 were located on the peptide face opposite to the binding site at least 9 Å from the nearest VEGF residue side chains, ruling out any short‐range intermolecular interaction. Although only lactam‐bridged ExxxK, DxxxK and KxxxD induced the formation of stable helix in the unbound state, all peptides of the series were helically folded when bound to VEGF. For comparison, structural references of lactam‐bridged helices are scarce (Figure S7).

#### Analytical isothermal titration calorimetry

Thermodynamic analysis of related protein‐ligands series requires specific design of isothermal titrations and realistic estimation of data uncertainties. ITC is the golden standard of thermodynamics binding measurement, as it is the only method capable of independently recording the change in enthalpy *ΔH* and the association constant *K*_a_ during binding. From these derive the Gibbs free energy ($\Delta G=-R.T.\ln K_{a}$ Equation (1)), the entropy variation *ΔS* (ΔG=ΔH-TΔS Equation (2)), and the variation of heat capacity upon binding ($\Delta Cp={Cp}_{\;bound\;}-{Cp}_{\;unbound\;}\;\approx \frac{\left(\Delta H\left(T\right)-\Delta H\left(T_{ref}\right)\right)}{T-T_{ref}}$ Equation (3)^[20]^). However, conclusions are often based on experiments that overlook systematic biases and overly optimistically assess uncertainties. In this study four points were optimized: first, the quality of each individual titration was improved by analytical methods of measuring titrant and titrated molecules concentrations, by the choice of optimal “c‐values” (the Wiseman parameter^[21]^), and low numbers of injections that increased signal/noise ratio (see supplemental). Second and importantly, because *ΔH* is proportional to the titrant concentration in the syringe, the enthalpies values must be determined on reverse titrations (protein injected into the peptide solution) to use the same reference for all peptides titrations. Third, each set of replicated titrations was analyzed globally, and the uncertainties “δ” were calculated at confidence level *P*=95 % with F‐statistics based contours of the error surface implemented in SEDPHAT.^[22]^ Fourth, all the titration series were duplicated at 20 °C and 37 °C.

The method resulted in the following mean relative errors: <δ*K*_d_/*K*_d_>=15 %, <δ(*ΔH*)/*ΔH*>=2.5 %, that gave <δ(*ΔG*)/*ΔG*>=0.9 %, and <δ(*TΔS*)/*TΔS*>=5.9 %. It can be compared to the <1 S.D. (*K*_d_)/*K*_d_>=22 % and <1 S.D. (*ΔH*)/*ΔH*>=24 %, previously reported for an inter‐laboratory comparison of the interaction between the bovine carbonic anhydrase II and the 4‐carboxybenzenesulfonamide.^[23]^

#### Thermodynamics and structure analysis

##### Upon binding, the entropy cost of folding outweighed the favorable entropy of desolvation

**Figure 3:**
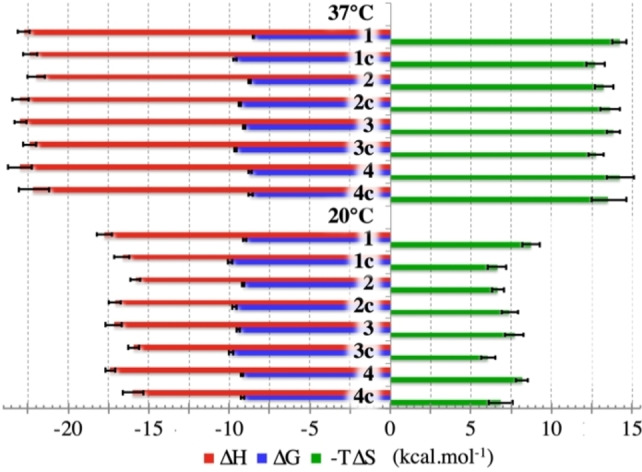
Graph summarizing macroscopic thermodynamic parameters of peptide−VEGF interactions on the first binding site.

The thermograms displayed one‐phase patterns that fitted well with the “two symmetrical and equivalent sites binding model” (see above, crystal structure section) of SEDPHAT (Figures S8a and S8b). Global analysis of direct and reverse titrations evidenced the absence of cooperativity between the two symetrical binding sites.^[10]^ All peptides had favorable enthalpies of binding counterbalancing unfavorable entropy contributions, which resulted in a narrow *ΔG* range corresponding to 6.5‐fold maximum variation of affinity toward VEGF (Figure 3, Tables 1, S7a and S7b). Raising the temperature from 20 °C to 37 °C resulted in the increase of both enthalpy and entropy intensities. Consequently, *ΔC*p were negative and close to −0.34 kcal⋅mol^−1^⋅K^−1^. As evidenced by Privalov and Makhatadze^[24]^ the *ΔC*p reflects mainly the dehydration of the protein‐peptide interface but also a weaker non‐hydration term associated with internal protein‐protein or ‐peptide interactions.^[20]^ Cooper emphasized that any macromolecular process involving a multiplicity of cooperative weak interactions can result in significant *ΔC*p effects.^[25]^ Therefore, the observed negative *ΔC*p can be related to co‐crystal and NMR structures that showed 950 Å^2^ of accessible surface area buried at the peptide‐protein interface, including roughly 700 Å^2^ formed by aliphatic and aromatic chains. Burying of apolar surface results in negative *ΔC*p, whereas the effect of the polar surface desolvation is the subject of debate.[^25^, ^26^] Particularly, we identified two trapped water molecules, that could also contribute to the negative *ΔC*p (Figure 2b).

**Table 1:** Thermodynamic parameters of cyclic and bicyclic peptides binding on VEGF, determined by ITC at 20 °C and 37 °C.

		K_d_ [nM]	*ΔG* [kcal⋅mol^−1^]	*ΔH* [kcal⋅mol^−1^]	−*TΔS* [kcal⋅mol^−1^]	nd:nr	*ΔC*p [kcal⋅mol^−1^.K^−1^]
20 °C	**1**	178 [151; 206]	−9.05 [−9.15; −8.97]	−17.8 [−18.2; −17.3]	8.7 [8.2; 9.3]	4 : 6	−0.29 [−0.32; −0.26]
	**1 c**	38 [32; 46]	−9.95 [−10.06; −9.84]	−16.6 [−17.0; −16.1]	6.7 [6.0; 7.2]	4 : 5	−0.34 [−0.38; −0.30]
	**2**	155 [135; 179]	−9.13 [−9.21; −9.05]	−15.8 [−16.1; −15.5]	6.7 [6.3; 7.1]	7 : 7	−0.36 [−0.40; −0.32]
	**2 c**	59 [48; 72]	−9.70 [−9.82; −9.58]	−17.1 [−17.5; −16.7]	7.4 [6.9; 7.9]	9 : 8	−0.35 [−0.39; −0.31]
	**3**	88 [74; 100]	−9.47 [−9.56; −9.39]	−17.2 [−17.7; −16.7]	7.7 [7.1; 8.3]	5 : 4	−0.34 [−0.38; −0.30]
	**3 c**	42 [34; 51]	−.89 [−10.01; −9.78]	−16.0 [−16.3; −15.6]	6.1 [5.6; 6.5]	3 : 6	−0.38 [−0.41; −0.35]
	**4**	134 [122; 148]	−9.22 [−9.27; −9.16]	−17.4 [−17.7; −17.1]	8.2 [7.8; 8.5]	4 : 5	−0.33 [−0.38; −0.28]
	**4 c**	149 [123; 180]	−9.16 [−9.27; −9.05]	−16.0 [−16.6; −15.4]	6.8 [6.1; 7.6]	3 : 5	−0.37 [−0.43; −0.31]
37 °C	**1**	1010 [930; 1089]	−8.51 [−8.56; −8.46]	−22.8 [−23.1; −22.4]	14.2 [13.8; 14.7]	4 : 4	
	**1 c**	156 [135; 183]	−9.66 [−9.75; −9.56]	−22.4 [−22.9; −21.9]	12.7 [12.2; 13.3]	4 : 4	
	**2**	669 [608; 737]	−8.76 [−8.82; −8.70]	−22.0 [−22.6; −21.5]	13.3 [12.7; 13.9]	4 : 5	
	**2 c**	258 [224; 295]	−9.35 [−9.44; −9.27]	−23.0 [−23.5; −22.5]	13.6 [13.0; 14.2]	3 : 6	
	**3**	386 [348; 421]	−9.10 [−9.17; −9.05]	−23.0 [−23.3; −22.6]	13.9 [13.4; 14.3]	3 : 4	
	**3 c**	168 [145; 194]	−9.61 [−9.71; −9.52]	−22.4 [−22.8; −22.0]	12.8 [12.3; 13.2]	4 : 5	
	**4**	725 [629; 828]	−8.71 [−8.80; −8.63]	−23.0 [−23.7; −22.3]	14.3 [13.5; 15.1]	2 : 3	
	**4 c**	770 [639; 932]	−8.68 [−8.79; −8.56]	−22.2 [−23.2; −21.3]	13.5 [12.5; 14.7]	1 : 4

95 % confidence intervals are given in brackets. Parameters are those of the first binding site. “nd:nr” refers to the number of direct and reverse titrations of VEGF by peptide used for the global analysis of ITC assays.

Considering the mostly hydrophobic surface at the interface, a favorable entropy of desolvation due to a significant hydrophobic effect was expected. Counterintuitively, we measured unfavorable entropies of binding, which we attributed to the configurational change of protein and peptide. Indeed, statistical thermodynamics may parse the entropy into a sum of marginal terms including the change of solvent entropy (${\Delta S}_{solv}$ ), the loss of rotation/translation degrees of freedom following the transformation of two molecular species in a single $\left({\Delta S}_{r/t}\right)$ , and the change of configurational entropy of protein and ligand $({\Delta S}_{config}^{protein-ligand}$ ).^[27]^ In a general case, solvent, protein, and ligand motions are partially correlated, resulting in $\Delta S^{binding}$ ≲${\Delta S}_{solv}+{\;\Delta S}_{r/t}+{\Delta S}_{config}^{protein-ligand}$ .^[5b]^ Therefore, the overall negative $\Delta S^{binding}$ observed in peptides series resulted from ${\Delta S}_{config}^{protein-ligand}<{-\;\Delta S}_{solv}-{\Delta S}_{r/t}$ . In this relationship, ${\Delta S}_{solv}\;>0$ as evidenced by hydrophobic VEGF‐peptide interfaces, and ${\Delta S}_{r/t}<0$ because of the loss of rotation/translation degrees of freedom. Moreover, because the loss of entropy of a single rotatable bond is estimated to 0.4‐1.2 kcal⋅mol^−1^ at 25 °C and the global loss of ${\Delta S}_{r/t}$ for small molecule binding to protein to 3.6–4.8 kcal⋅mol^−1^, the amplitude of ${\Delta S}_{r/t}$ is also supposed to be low compared to the intensity of the higher terms ${\Delta S}_{solv}$ and ${\Delta S}_{config}^{protein-ligand}$ upon protein ligand binding.^[28]^

In conclusion, the overall entropy penalty may be interpreted as the consequence of the counterbalancing of two main effects: a favorable ${\Delta S}_{solv}$ and unfavorable ${\Delta S}_{config}^{protein-ligand}$ .

#### Thermodynamics and structural analysis converged on a mutual adjustment of protein and peptide

The unfavorable ${\Delta S}_{config}^{protein-ligand}$ was supported by structural results: in the current series of peptides, NMR analysis has shown that unbound peptides adopted fast exchanging conformations and partially unfolded structures in solution. In contrast, in the bound state, a nearly identical fold was observed in NMR and crystal structures. A double structural adjustment was evidenced upon binding: on VEGF, the Cys W61‐Asn W62 peptide‐bond flipped to bind the type I β‐turn of the peptide, which allowed Asn W62 to pack against the Trp P7 indole and establish several stabilizing H‐bonds. On the peptides, the amino acids P1‐11 wrapped around the side chain of the Trp P7 to form an apolar cluster and a very specific π‐stacking of Trp P7, Phe P12, Phe W17 and Tyr W21. Therefore, the bound structures did not correspond to the lowest energy states of the peptides and protein observed in the unbound state.

In the literature, two conceptual models were consistent with the protein‐ligand binding mechanisms and thermodynamics that have been observed experimentally. These are “conformational selection” and “induced fit” models.^[29]^ The current data do not allow a conclusion as it would require a kinetic characterization.^[30]^ However, the first model seemed unlikely because it postulates that the dynamic equilibrium conformations of unbound ligands and proteins could reach simultaneously the complementary conformations, which we did not observe. In particular, the NMR data evidenced that the Trp P7 side chain was systematically oriented toward the solvent in the unbound state (Figure 1d). Conversely, the induced fit model seemed more plausible since the β‐turn of the peptide was often present in solution in our NMR analysis. Moreover, the conformational exchange involving the flipping of the ϕ dihedral angle of VEGF Asn W62 was already noticed in several crystal structures of apo‐VEGF,^[8]^ which assumes that the two conformations are in dynamical equilibrium in solution. In addition, in a molecular dynamic simulation of the interaction of peptide v107 with VEGF, Horta et al. observe that both Tyr W21 and Asn W62 residues contribute significantly to the electrostatic binding energy.^[9]^ Therefore, the binding of the peptide β‐turn to VEGF could be an initial binding event, strong enough to allow multiple tentative collisions to achieve the conformation shift of the peptide residues P1‐11 toward the bound conformation.

**Figure 4:**
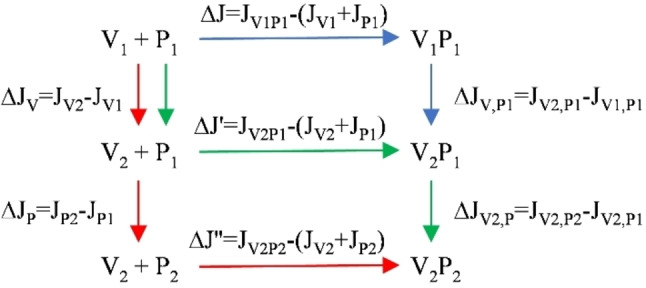
Scheme illustrating thermodynamically equivalent paths from the unbound and unfolded species V1+P1 to the folded complex V2P2. *J* can represent a state function *G*, *S* or *H* at constant (*p*,*T*), and the P1/P2 and V1/V2 are unbound and bound conformations of the peptide and VEGF, respectively. The green arrows correspond to the induced fit hypothesis: the folding of the protein, followed by the binding of the peptide and its rearrangement. The alternative hypothesis of the conformation selection model corresponds to red arrows.

The conformational selection model in Figure 4 allows a thermodynamic separation of the folding of unbound peptide and of the binding process of folded species: as NMR, MD and crystal structures have shown that the monocyclic and bicyclic peptides bound in a similar manner to the VEGF, and since the conformations of the side chains and water molecules involved in the binding site were identical throughout the series, the differences in thermodynamics observed between molecules seemed mainly related to the process of unbound peptide folding ($\Delta J_{P}=J_{P2}-J_{P1}$ ).

#### Sequence effect in the monocyclic peptide series thermodynamics

Although the residues in position P10 or P14 were not involved in the binding site, the sequence differences impacted the thermodynamics of binding to VEGF (Table 2a). The position of K in P14 (**2** and **3**), exposed to the solvent near the C‐terminus of α‐helix, was entropically favored over the P10 position which was more conformationally constrained (**1** and **4**) after the peptide folding. Differences in binding enthalpies and entropies up to 2.0 kcal⋅mol^−1^ were measured, but they were largely compensating each other, resulting in similar Gibbs free energies of binding. (See the supplementary discussion for detailed analysis and comparison with literature data).

**Table 2:** a) Differences of thermodynamics between monocyclic peptides b) Differences of thermodynamics between bicyclic and monocyclic counterparts.

	*Δ*(*ΔG* ^20 °C^)	*Δ*(*ΔG* ^37 °C^)	*Δ*(*ΔH* ^20 °C^)	*Δ*(*ΔH* ^37 °C^)	*Δ*(‐*TΔS*)^20 °C^	*Δ*(‐*TΔS*)^37 °C^	*Δ*(*ΔC*p)
a)	[kcal⋅mol^−1^]		[kcal⋅mol^−1^]		[kcal⋅mol^−1^]		[kcal⋅mol^−1^.K^−1^]
**1**–**4**	0.16±0.10	0.20±0.09	−0.4±0.6	0.2±0.8	0.6±0.7	0.0±1.0	0.04±0.06
**2**–**3**	0.33±0.12	0.34±0.08	1.4±0.6	1.0±0.7	−1.0±0.8	−0.6±0.8	−0.02±0.05
**2**–**1**	−0.08±0.12	−0.25±0.08	2.0±0.6	0.7±0.7	−2.0±0.7	−1.0±0.8	−0.07±0.05
**3**–**4**	−0.25±0.10	−0.39±0.10	0.2±0.6	0.0±0.9	−0.5±0.7	−0.4±1.0	−0.01±0.06
b)							
**1 c**–**1**	−0.89±0.14	−1.15±0.11	1.2±0.7	0.4±0.6	−2.1±0.8	−1.5±0.7	−0.05±0.05
**2 c**–**2**	−0.56±0.14	−0.59±0.10	−1.3±0.5	−1.0±0.8	0.7±0.7	0.4±0.9	0.02±0.05
**3 c**–**3**	−0.51±0.10	−0.51±0.10	1.2±0.7	0.6±0.6	−1.6±0.8	−1.1±0.7	−0.04±0.05
**4 c**–**4**	0.06±0.12	0.04±0.14	1.4±0.7	0.8±1.2	−1.3±0.9	−0.8±1.3	−0.04±0.08

Confidence intervals are calculated at *P*=95 %. Note that *Δ*(*ΔC*p) varied accordingly to entropy variation *Δ*(*ΔS*) and followed the relation $Cp-T\frac{dS}{dT}\;$ at p constant.^[20]^

#### Configurational entropy and affinity changes cannot be interpreted as pure conformational effects

The lactam bridges reduce the number of conformations reached by the unfolded peptides. The conformational entropy variation of bicyclic peptides upon binding should then be reduced compared to the monocyclic analogus, especially for **1 c**, **2 c** and **3 c** whose α‐helix was pre‐organized. Consequently, neglecting the differences of *ΔS*_solv_ between bicyclic and monocyclic peptides, positive values of T(*ΔS*_bicycl._–*ΔS*_monocycl._) were expected, even to a lower extent for peptide **4 c** whose unbound peptide α‐helix was not stabilized. However, in our data, the stabilization of α‐helix by the lactam bridge directly improved the affinity but did not correlate with an entropy gain (Table 2b): the affinity of **4 c** was slightly decreased despite an entropy gain, and the binding of peptide **2 c** to VEGF exhibited a less favorable entropy variation than peptide **2**, despite the high helicity of unbound peptide **2 c** and improved affinity. An explanation could be a difference in *ΔS*°_solv_ between peptides **2 c** and **2**, but the *ΔC*p of both peptides did not evidence any significant variation in the desolvation process upon binding to VEGF. Therefore, our data showed that the entropy differences between closely related peptides are a complex phenomenon that cannot be interpreted solely as effects of conformational restriction.

#### Unfavorable vibrational entropy changes counterbalance the conformational entropy gain due to preorganization

In the following section, we proposed that these anomalies resulted from the constraints associated with the lactam cyclization, which would alter the vibrational motion of individual conformations. Indeed, in statistical thermodynamic the configurational entropy may be then formally decomposed in two parts, the conformational entropy and the vibrational entropy.[^1a^, ^5b^, ^27b^, ^31^]

(4) $${\Delta S}_{config}={\Delta S}_{confor}+{\Delta S}_{vib}$$

The free energy function that describes the system from conformational space comprises multiple energy wells. The conformational entropy part reflects its distribution across the different wells:

$S_{confor}^{0}=-R\sum_{j}^{n}{(p}_{j}lnp_{j}$ ) (Boltzmann equation *p*_j_ is the probability of occupying the energy well j).

**Figure 5:**
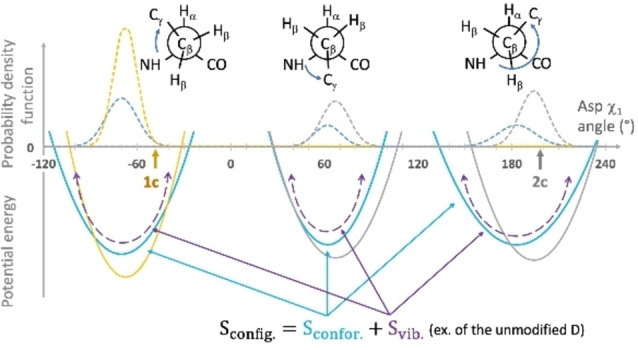
Configurational entropy decomposition in conformational and vibrational terms. Example of an Asp residue involved in lactam bridge. Dashed lines: probability density function of χ_1_. Blue: distribution observed in the PDB for unmodified Asp residue (D),^[18]^ or from molecular simulation for D_i_‐K_i+4_ (grey) and K_i_‐D_i+4_ (orange) lactam‐bridged Asp residues in a short α‐helix.^[14]^ The lactam bridges and the α‐helix fold imposed additional constraints to Asp χ_1_ and shifted the χ_1_ distribution of D_i_‐K_i+4_ and K_i_‐D_i+4_, prohibiting certain conformations. Arrows indicate values of X‐ray structures of **1 c** and **2 c** VEGF‐bound peptides. Solid lines: corresponding potential energies calculated from Maxwell‐Boltzmann statistics. Only the negative potentials are populated. The conformational entropy refers to the number of energy wells and their probability across the conformation space (χ_1_). In this example, *S*_confor_(D) > *S*_confor_ (D_i_‐K_i+4_) > *S*_confor_ (K_i_‐D_i+4_). The vibrational entropy is the Boltzmann‐averaged entropy of the individual energy wells, reflecting oscillation around well's equilibrium. In this example the individual energy well for *χ*_1_= −67° is deeper and narrower for K_i_‐D_i+4_ according to the corresponding energy well of unmodified Asp: $S_{Vib,Ki-Di+4}^{\chi_{1}=-67^{\circ }}<S_{Vib,D}^{\chi_{1}=-67^{\circ }}$ .

The vibrational entropy is the weighted mean of $S_{j}^{0}$ , that describes the local fluctuation of the molecule within a single well in the vicinity of a defined conformation. This term is proportional to the average width of the energy wells (Figure 5).

$$S_{vib}^{0}=\sum_{j}^{n}p_{j}S_{j}^{0}$$

Although the amplitude of vibrational entropy is nearly an order of magnitude larger than conformational entropy, its variation on a protein folding process is generally estimated^[31]^ or calculated^[32]^ to be small relative to the conformational term. However, the molecular dynamic analysis of a small molecule (Amprenavir) binding to the HIV protease evidenced that most of the computed configurational entropy loss can result from the reduction in vibrational entropy due to a narrower well of free energy in the bound state than in the unbound state.^[27b]^

In the structures of bicyclic peptides bound to VEGF, we observed that constraints due to lactam cyclization were tightened compared to unbound state. Indeed, as discussed above, the C‐terminus of peptides **1 c**, **2 c**, and **3 c**, that displayed α‐helix signature in CD, were partially distorted toward 3_10_ helix and agitated in bound state. Although each covalent bond forming the lactam bridge had adopted a conformation which minimized geometric constraints, such as steric contacts, rotation angles and angle bends, the α‐helix and side chains geometry on residues P10 and P14 deviated from ideal values. Therefore, lactam cyclization can result in sub‐optimal geometry with remaining steric constraints, where the side chains did not adopt the standard conformers (**1 c** and **3 c** peptides). This would lead to a large and unsteady configurational space. In an opposite way, the equilibrium for idealized geometry can be restricted to a narrower configurational space, which reduced the movement of the bound peptide (**2 c**). The free energy well of a bicyclic peptide, corresponding to a conformation, can therefore be enlarged, or narrowed in the VEGF‐bound forms.

Consequently, these structural data let us hypothesize that the unexplained entropy variations described above could be the consequence of significant changes in vibrational entropy of the bicyclic peptides between the free and the VEGF‐bound states. Figure 4 described equivalent thermodynamics paths from the unbound and unfolded state (V_1_+P_1_) to the folded and bound state (V_2_P_2_). Considering the “conformation selection model” the energy was decomposed in three terms: $\Delta J=\Delta J_{V}+\Delta J_{P}+\Delta J{}^{'}{}^{'}$ . $\Delta J_{V}$ corresponded to the configurational change of VEGF from V_1_ to V_2_ and was independent of the peptide. The $\Delta J_{P}$ term described the process of peptide folding and differed from one peptide to another. The last term $\Delta J{}^{'}{}^{'}$ described the binding process of pre‐folded peptide and pre‐folded VEGF. We evidenced above, from structural analysis, that the variation of this term must be neglectable throughout the series of mono and bicyclic peptides. For example, considering the entropy term $\Delta S{}^{'}{}^{'}$ ≲${\Delta S{}^{'}{}^{'}}_{solv}+{\;\Delta S{}^{'}{}^{'}}_{r/t}+{\Delta S{}^{'}{}^{'}}_{config}^{protein-ligand}$ , ${\Delta S{}^{'}{}^{'}}_{solv}$ corresponded to the dehydration of binding surfaces that were identical in the peptide series, ${\Delta S{}^{'}{}^{'}}_{r/t}$ was constant, and because we defined V_2_ and P_2_ as the bound configurations, ${\Delta S{}^{'}{}^{'}}_{config}^{protein-ligand}$ was equal to zero. Consequently, the differences in thermodynamics observed between related peptides can be related mainly to the differences in the process of peptide folding, thus $\Delta \left(\Delta J\right)\approx \Delta \left(\Delta J_{p}\right)$ . Considering the entropy terms, one can write: $\Delta \left(\Delta S\right)\approx \Delta \left({\Delta S}_{config}\right)={\Delta (\Delta S}_{confor}+{\Delta S}_{vib})$ , where ${\Delta S}_{config}\;$ was the entropy of configuration of the system, including the solvent, during the peptide folding process.

Numbering **i** and **ic** the monocyclic and bicyclic analogues, it resulted:

$\Delta \left({\Delta S}_{vib}\right)\approx \Delta \left(\Delta S\right){-\Delta (\Delta S}_{confor})\;$ that is

(Eq. 5) $${\Delta S}_{i,vib}-{\Delta S}_{ic,vib}\approx {(\Delta S}_{i}-{\Delta S}_{ic})-{(\Delta S}_{i,confor}-{\Delta S}_{ic,confor})$$

Moreover, (i) (*ΔS*_**i**,confor_–*ΔS*_**ic**,confor_) <0 because the number of degrees of freedom upon binding decreased more for monocyclic than for bicyclic peptides, especially if compared to unbound **1 c**, **2 c** and **3 c**, which were partially preorganized, (ii) For peptides i=**2** we measured (*ΔS*_**2**_–*ΔS*_**2c**_) >0, and for peptides i=**1**, **3** and **4** (*ΔS*_**i**_–*ΔS*_**ic**_) <0 (iii) according to the literature and for monocyclic peptides $\Delta S_{i,vib}\approx 0$ , because the vibrational entropy variation is negligible for proteins during folding compared to the changes in conformational entropy.[^31^, ^32^] However, for bicyclic peptides we assumed no hypothesis on $\Delta S_{ic,vib}$ value because additional constraints may modify the width of the energy well upon binding.

It will follow from Equation (5) several qualitative relationships:

For i=**2**, ${\Delta S_{2c,vib}=S}_{2c,vib}^{bound}-S_{2c,vib}^{unbound}<0$ . The vibrational energy of the protein‐peptide complex would be lower than unbound species. This unfavorable difference meant that the width of the free energy well of the bound state would be reduced compared to the unbound state. This can be linked experimentally to the fact that, in the lactam bridge of peptide **2 c**, the residues P10 and P14 adopted standard rotamers and therefore a stable and constrained conformation. It was not the case for **1 c** and **3 c** peptides, which did not adopt standard rotamers. The overall analysis by spectroscopic methods also evidenced that **2 c** was the most structured peptide in solution with **3 c**.

For i ≠ **2**, we cannot conclude qualitatively on the sign of$\;\Delta S_{ic,vib}$ . It is plausible that same behavior existed, with $S_{ic,vib}^{bound}-S_{ic,vib}^{unbound}<0$ , but its amplitude would be modulated depending on the peptide. For *i*=**4**, because the free peptide was unfolded, the negative difference (*ΔS*_**4**,confor_–*ΔS*_**4c**,confor_) would be of smaller amplitude than for **1**/**1 c**, **2**/**2 c** and **3**/**3 c**, consequently, (*ΔS*_**4**,vib_–*ΔS*_**4c**,vib_) should also be decreased. Indeed, crystal structure and NMR data evidenced that the C‐terminus part of the **4 c** helix was poorly structured, making it the most dynamic among the bicyclic peptides. Although P10 and P14 residues were in standard rotamer conformation, the constraints due to the lactam bridge destabilized the helix fold and acted as helix breaker at the P15 residue.

Overall, the experimental structural and ITC data supported the theoretical proposal that the vibrational entropy decreased when the free energy well of a conformation was narrowed, i. e. when the rigidity of the bound bicyclic peptide increased. This unfavorable vibrational entropy counterbalanced all or part of the favorable conformational entropy gain.

#### Preorganized bicyclic peptides have partially escaped the Entropy‐Enthalpy compensation

**Figure 6:**
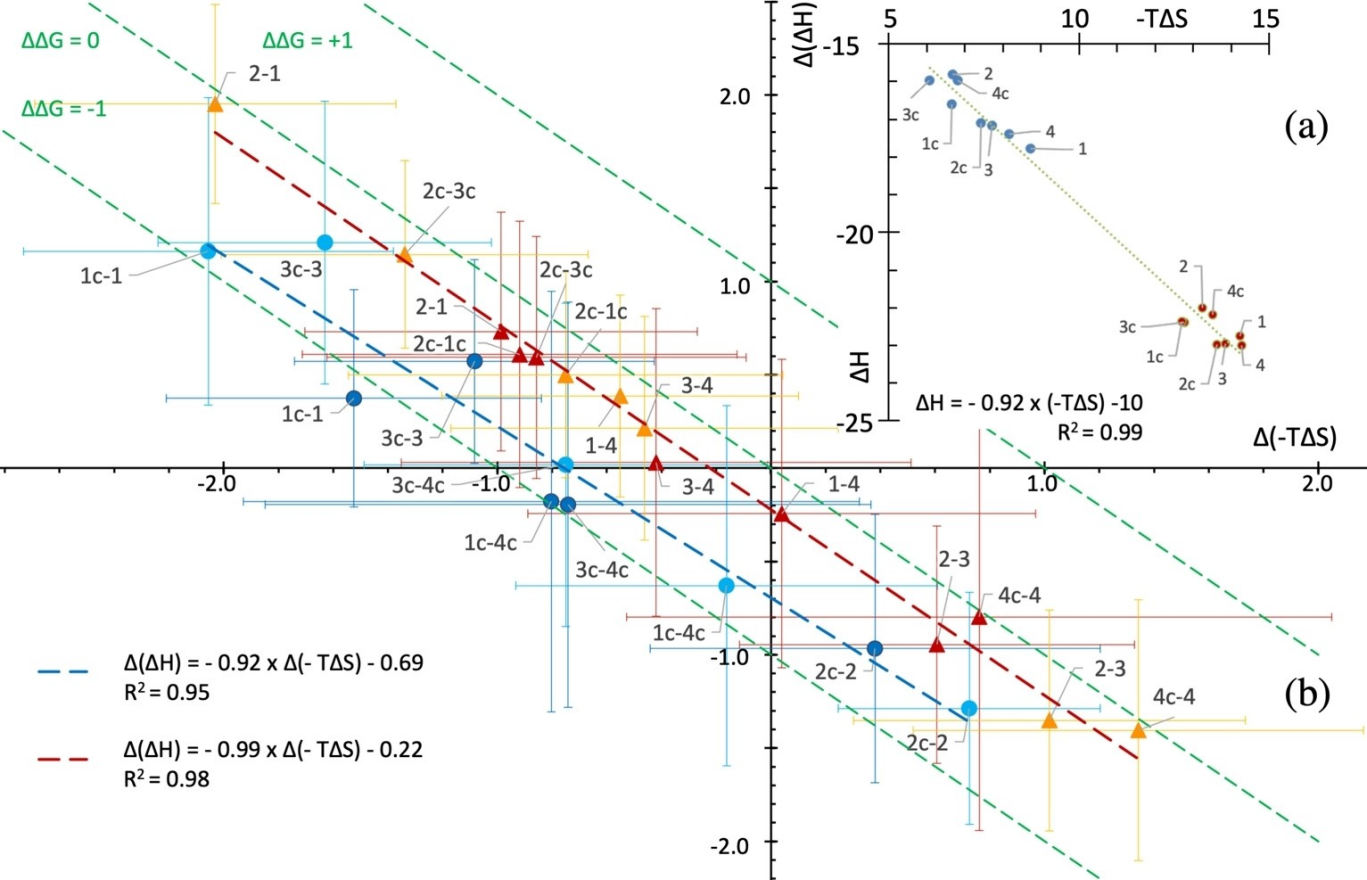
a) (top right graph) Plot of *ΔH* as a function of ‐*TΔS* for the whole set of titrations at 20 °C (blue dots) and 37 °C (red dots). b) (main graph) Plot of *Δ*(*ΔH*) as a function of *Δ*(‐*TΔS*) for a selected pair of peptides. Blue circles: helically structured bicyclic peptides and their monocyclic counterparts (**1 c**/**1**, **2 c**/**2** and **3 c**/**3** or inversion of lactam bridge orientation **3 c**/**4 c**). ‐ Red triangles: related peptides whose both structures in unbound state were similar, either unfolded or helical. The pairs, calculated in the direction *ΔΔG* <0, correspond to inversion of Asp/Glu and Lys at positions P10 and P15, or substitutions of Asp/Glu at position P10 or P15, or effect of the lactam bridge on **4 c**/**4** peptides, or inversion of lactam bridge orientation (**2 c** and **1 c**). The points at the top left of the scheme correspond to favorable entropy and unfavorable enthalpy differences, and the points in the lower right part characterize unfavorable entropy and favorable enthalpy differences. Dark blue/red and light blue/orange points correspond to measurements at 37 °C and 20 °C respectively. Error bars confidence level was *P*=0.95. Iso‐ Gibbs free energies were displayed in green dashed lines. Energy units are kcal⋅mol^−1^.

For the series of peptides, the changes in entropy were partially compensated by changes in enthalpy following the so‐called enthalpy/entropy compensation. The top panel (a) of the Figure 6 showed a linear relation between *ΔH* and *TΔS* in all the titrations of peptides binding to VEGF. The regression line gave *ΔH*=0.92 *TΔS* –10 kcal⋅mol^−1^, which was close to Equation (2) (*ΔH*=*TΔS*+*ΔG*). The location at lower *ΔH* and ‐*TΔS* of the preorganized peptides **1 c**, **2 c** and **3 c** at 20 °C and 37 °C directly reflected the lower *ΔG* values in comparison to the other unfolded peptides. Figure 6b specifically compared the thermodynamics of related peptides, which showed the energetic changes resulting from local chemical modification. This graphical representation had the advantage of subtracting some thermodynamical phenomena that were almost identical for the two compared peptides binding processes, such as *ΔS*_solv_ or *ΔS*_r/t_, and highlighted the specific effect of helical pre‐organization of the peptides in unbound state. Indeed, a remarkable feature of this figure was the obvious possibility to separate the data into two sets as a function of the differences of folding between the peptides that were compared: on one side, peptides with high helicity content in solution compared to monocyclic counterparts (“light and dark blue set”), and on the other side, peptides compared to analogues having no helical fold in unbound state (“orange and red set”). Each kind of transformation was roughly aligned, which resulted in two distinct regression lines with similar slope but different Y‐intercept. The enthalpy change was always positively correlated to entropy change, however the helical pre‐folding specifically resulted in a net gain in affinity: the regression line of the “blue set” was *Δ*(*ΔH*)=−0.92 *Δ*(‐*TΔS*)–0.69. As *Δ*(*ΔH*)=−*Δ*(*TΔS*)+*Δ*(*ΔG*), the equation meant that any favorable or unfavorable change in entropy due to the lactam bridge cyclization resulted in a gain in *ΔG* close to −0.69 kcal⋅mol^−1^. It corresponded to the mean Gibbs free energy gain due to the pre‐organization of bicyclic peptides **1 c**, **2 c** and **3 c** compared to monocyclic analogs. This 3.3‐fold equivalent increase in affinity agreed with the ratio generally reported in the literature (2 to 6 fold), provided that the introduction of a single lactam bridge is the sole modification.^[33]^ The significance of such an observation was that the helical pre‐folding of peptides **1 c**, **2 c** and **3 c** resulted in an affinity gain due to the systematic reduction in conformational entropy – but not in configurational entropy – of unbound peptides, which partially escaped the EEC.

## Conclusion

A comprehensive series of closely related ligand peptides was compared to decipher the effect of sequence and lactam bridges constraints on their affinity towards VEGF. Lactam bridges were designed on the side of an α‐helix far enough from the binding site to isolate the entropic effect of the cyclization from the consequence of the direct binding to the protein. The thermodynamics of binding has been analytically measured to reduce ITC uncertainties. Next, crystal structures of constrained peptides bound to VEGF were determined, and unbound peptide structures were examined by electronic circular dichroism and NMR. This work provided a unique set of precise thermodynamic data for a series of structurally well characterized and closely related protein‐ligand complexes, which can be used for the future prediction of binding enthalpies and entropies.

Our results evidenced that the KxxxD, DxxxK and ExxxK lactam bridges were able to stabilize the α‐helix. Their positive effects on the affinity towards VEGF were particularly significant at higher temperature, up to 6.5 folds at 37 °C. Conversely, the lactam bridge KxxxE did not stabilize the α‐helix in the unbound state. The resulting energetic schemes of the peptides binding to the protein were complex and cannot be understood by the mere description of the binding interface. Comparisons of bound and unbound structures allowed to rationalize binding thermodynamics. We have evidenced that peptide molecules bind to VEGF in conformations higher in energy than their minima in the unbound state, following either kinetic models of induced fit or conformational selection. Among the current series of peptide molecules, the differences in Gibbs free energy originated mainly from the structural behavior of the peptides in the unbound state and resulted from the fine opposing balance between entropy and enthalpy of folding. We observed that the favorable conformational entropy gains due to the cyclization constraints, although of low amplitude, partially escaped the EEC when the pre‐folded peptides bound to VEGF. Moreover, the apparent inconsistency of entropy changes in peptides **2**/**2 c** likely results from differences in vibrational entropy due to the cyclization constraints. We propose that the vibrational entropy decreases when the flexibility of the conformation of the bound bicyclic peptide decreases, i. e. when the free energy well is narrowed. This effect could also be present for peptide linkers other than lactams, like hydrocarbon chains, because we evidenced the absence of interactions between the linker and the target protein. As a rule, our work suggests that an untargeted reduction in the degree of freedom of a ligand may be useless due to the near total entropy/enthalpy compensation. An ideal linker should shift the conformational equilibrium of the unbound ligand toward a folded bioactive‐like conformation without excessive constraints. Chemical linkers used to cyclize the ligands must be chosen by selecting those which are close to a stable conformational energy state when folded. However, some flexibility in the linker should be kept to allow a better adjustment of the ligand toward the target, which can improve the enthalpy of binding and avoid unfavorable vibrational entropy change. Similarly, an unfavorable change of entropy can result from a linker that is too stiff, that suggests that it could be chemically modified to increase the vibrational entropy of the bound form and to improve affinity.

## Experimental Section

Experimental Procedures and data are provided in Supporting Information.

## Conflict of interest

The authors declare no conflict of interest.

1

## Supporting information

As a service to our authors and readers, this journal provides supporting information supplied by the authors. Such materials are peer reviewed and may be re‐organized for online delivery, but are not copy‐edited or typeset. Technical support issues arising from supporting information (other than missing files) should be addressed to the authors.

Supporting Information

## Data Availability

The data that support the findings of this study are available from the corresponding author upon reasonable request.
