# Supplementary material for: Structural and ITC Characterization of Peptide‐Protein Binding: Thermodynamic Consequences of Cyclization Constraints, a Case Study on Vascular Endothelial Growth Factor Ligands
Source: Chemistry. 2022 Jul 7;28(48):e202200465. doi: 10.1002/chem.202200465 (PMC9543606; doi:10.1002/chem.202200465)

Chemistry–A European Journal

Supporting Information

Structural and ITC Characterization of Peptide-Protein Binding: Thermodynamic Consequences of Cyclization Constraints, a Case Study on Vascular Endothelial Growth Factor Ligands

Jean-François Gaucher,* Marie Reille-Seroussi, and Sylvain Broussy*

Table of content

1 MATERIAL AND METHODS 2

1.1 Peptide synthesis 2
1.2 Protein expression and purification 2
1.3 Crystallization, data collection, structure resolution 2
1.4 Spectrometric methods 3
1.5 ITC assays 3

2 ADDITIONAL DISCUSSIONS 4

2.1 Circular dichroism spectra of unbound peptides 4
2.2 NMR structures of unbound peptides 4
2.3 Analytical requirements in ITC analysis 5
2.4 Sequence effects in monocyclic peptides 6

3 REFERENCES 7

4 SUPPLEMENTAL FIGURES 8

4.1 Figure S1. Synthesis of bicyclic peptides. 8
4.2 Figure S2. Superposition of the structures of VEGF:peptide **1c** and VEGF in complex with VEGFR1. 9
4.3 Figure S3. Orthogonal T-shaped π -stacking. 9
4.4 Figure S4. Structures of the bicyclic peptide helices. 10
4.5 Figure S5. Detail of **1c** helix structure 10
4.6 Figure S6. Representation of the backbones of bicyclic peptides and v107. 11
4.7 Figure S7. Superposition of available crystal structures of helices stabilized by lactam bridge. 11
4.8 Figure S8a. Examples of reverse and direct ITC of VEGF by peptides at 20° C. 12
4.9 Figure S8b. Examples of reverse and direct ITC of VEGF by peptides at 37° C. 13
4.10 Figure S9. Examples of reverse and direct titration of $BaBr_2$ and $BaCl_2$ by 18-crown-ether-6 at 20°C. 14

5 SUPPLEMENTAL TABLES 14

5.1 Table S1 Mean peptides residue molar ellipticities. 14
5.2 Table S2 Proton amide chemical shift variation with temperature. 15
5.3 Table S3 NMR chemical shift differences of diastereotopic protons of the lactam bridge 15
5.4 Table S4 RMSD values of NMR peptides structures *v.s.* crystal structure. 16
5.5 Table S5 Clustering of NMR structures. 16
5.6 Table S6 Diffraction data and crystal structures refinement 17
5.8 Table S7a ITC data. 18
5.9 Table S7b ITC data. 19
5.10 Table S7c ITC data 20
5.11 Table S8 ITC data. 20

6 ANALYTICAL HPLC AND MASS SPECTROMETRY 21

6.1 Peptide **1** 21
6.2 Peptide **1c** 22
6.3 Peptide **2** 23
6.4 Peptide **2c** 24
6.5 Peptide **3** 25
6.6 Peptide **3c** 26
6.7 Peptide **4** 27
6.8 Peptide **4c** 28

7 NMR DATA 29

7.1 Peptide **1** 29
7.2 Peptide **1c** 29
7.3 Peptide **2** 30
7.4 Peptide **2c** 30
7.5 Peptide **3** 30
7.6 Peptide **3c** 31
7.7 Peptide **4** 31
7.8 Peptide **4c** 31
7.9 NOE restraints used in XPLOR-NIH simulated annealing 32
7.10 XPLOR patches nle, lald and lale and peptide bond file top19_bicycle.pep 43
7.11 NMR spectra 45

1 Material and methods

1.1 Peptide synthesis

General remarks

N^{α}-Fmoc amino acids with standard side chains protections and coupling reagents (HBTU, DIC, HOBt, OxymaPure and DIEA) were purchased from SDS Carlo-Erba. The NovaSyn® TGR resin (loading 0.25 mmol / g) and PyBOP were obtained from Novabiochem. All other reagents were purchased from Aldrich. All solvents were purchased from SDS Carlo-Erba, except NMR solvents, which were obtained from Eurisotop. Peptides were synthesized on solid phase and under microwave irradiation using a CEM Liberty 1 synthesizer. Microwave irradiation was employed following the synthesizer provider recommendations for both coupling and deprotection steps. HPLC analysis was realized using a Phenomenex Luna C18 column (5 µm, 2.5 x 250 mm). Peptides were purified by semi-preparative HPLC on a Grace Alltima C18 column (5 µm, 10 x 250 mm) using the indicated gradient program at a flow rate of 2 mL/min. For HPLC analysis, the following solvents were used: solvent A was water with 0.1% TFA and solvent B was a 70% acetonitrile in water solution with 0.09% TFA. The products were detected by UV at 220 and 254 nm.

Monocyclic peptides synthesis

Peptides were synthesized at a 0.1 mmol scale with NovaSyn® TGR resin (0.25 mmol / g). Coupling reactions were performed using N^{α}-Fmoc amino acids (5 eq. related to the resin) activated with DIC (4.9 eq.) and Oxyma Pure (5 eq.) in DMF. After each coupling step, Fmoc removal was achieved by treating the resin with 20% piperidine in DMF. The deprotection and cleavage of the peptide from the resin was done by treatment with 10 mL of TFA/H_2O/EDT/TIPS (94:2.5:2.5:1.0) for 3 h at room temperature. After removal of the resin, the filtrate was concentrated under a stream of argon and precipitated with cold diethyl ether. The precipitate was isolated by centrifugation and washed with diethyl ether, affording the crude peptide. The cyclization was carried out at 1 mM peptide concentration by addition of a 100 mM aqueous solution of $NH_4.HCO_3$. The reaction was monitored by HPLC and Ellman analysis. After completion of the reaction, the solvent was evaporated, and the crude peptides were purified by semi-preparative HPLC using the indicated gradient program. The products were collected and analyzed by HPLC and mass spectrometry. Pure fractions were pooled and lyophilized to yield the peptides as white solids in 15-25% yields.

Bicyclic peptides synthesis

The first six amino acids at the C-terminal extremity of the peptides were synthesized on solid phase and under microwave irradiation at a 0.1 mmol scale with NovaSyn® TGR resin (0.25 mmol / g). Coupling reactions were performed using N^{α}-Fmoc amino acids (5 eq. related to the resin) activated with DIC (4.9 eq.) and Oxyma Pure (5 eq.) in DMF. Excepted for the last N-terminal amino acid, the Fmoc groups were removed under standard conditions after each coupling reaction. Orthogonal protecting groups (phenylisopropyl ester, O-Pip, of the aspartic or glutamic acid and the methyltrityl, NH-Mtt, of the lysine) were removed by treating the peptide resin with 3% TFA in DCM (5 × 2 min). Then, the resin was washed with DMF (2 × 2 min) and DCM (2 × 2 min). The resin was neutralized by washing with 5% DIPEA in DMF (2 × 3 min) and washed again with DMF (2 × 2 min) and DCM (2 × 2 min). The cyclization was achieved on-resin using 4 eq. of PyBOP and 10 eq. of DIEA in DMF. The reaction mixture was stirred at room temperature for 14 hours and was monitored by cleavage of 10 mg resin and HPLC analysis of the peptide. After complete disappearance of starting material, the resin was washed with DMF and the cyclic peptide was elongated under standard conditions. After elongation and removal of the last Fmoc, the peptide was simultaneously deprotected and cleaved from the resin by treatment with TFA/H_2O/EDT/TIPS (94:2.5:2.5:1.0) for 2 hours. The solution was then filtered and concentrated under a stream of argon, and the crude peptide was precipitated with cold diethyl ether. The precipitate was isolated by centrifugation and washed with diethyl ether. The crude peptide was then engaged in the formation of the disulfide bridge. The cyclization reaction was carried out at 100 µM peptide concentration in a solution mixture of H_2O/CH_3CN/NH_4HCO_3 (200 mM) (25 %:25 %:50 %). The reaction was monitored by HPLC and Ellman analysis. After completion, the solvent was evaporated, and the crude peptide was purified by semi-preparative HPLC using the indicated gradient. The products were collected and analyzed by HPLC and mass spectrometry. Pure fractions were pooled and lyophilized to obtain the desired bicyclic peptide as white solids in 5-10% yields. Mass spectrometry MALDI spectra of peptides were recorded on an Applied Biosystems 4700 proteomic analyzer spectrometer at UPMC (Paris, France).

1.2 Protein expression and purification

The VEGF protein was prepared as previously described.[1] Briefly, a synthetic gene encoding the human VEGF protein (residues 13-107) was cloned into pET22b(+) and expressed as an insoluble protein in *E.coli* Rosetta™2(DE3) bacterial strain (Novagen). The inclusion bodies were harvested, solubilized in urea, and refolded by dialysis. The soluble protein was purified by cation exchange chromatography and size exclusion chromatography. Subsequently, it was extensively dialyzed against water, cryo-frozen, lyophilized and kept at -20°C.

1.3 Crystallization, data collection, structure resolution

Lyophilized protein and peptides were dissolved in HEPES/NaOH 50 mM pH 7.5 NaCl 150 mM and mixed at a molar ratio 1:4 (VEGF:peptide) (60 µM final). The mixtures were concentrated and purified by size exclusion chromatography (Superdex 75HR 10/300). For each mixture of protein and peptide the main peaks were eluted at a volume corresponding to the expected size of VEGF:2 peptide molecules complex. The complexes were concentrated on ultrafiltration unit (3 kDa) and the buffer was exchanged for Tris/HCl 10 mM pH 8.5 by three successive steps of five-fold dilution followed by concentration. VEGF:**1c,** VEGF:**2c**, VEGF:**3c**, and VEGF:**4c** were respectively concentrated at 13.5 mg/ml, 17.0 mg/ml, 9.8 mg/ml and 17.0 mg/ml, and then crystalized by vapor diffusion method: 1 µl of complex solution was mixed with 1 µl of reservoir at 18°C. The complexes VEGF:**1c**, VEGF:**2c**, and VEGF:**4c** were crystallized against reservoirs composed of NaOAc/HCl 100 mM pH 4.5, $(NH_4)_2PO_4$ 150 mM, 2-Methyl-2,4-pentanediol (MPD) 35% (v/v). VEGF:**3c** was crystallized against NaOAc/HCl 100 mM pH 4.5, $CaCl_2$ 20 mM, MPD 36% (v/v). Note that VEGF:**1c**, VEGF:**2c**, and VEGF:**4c** were also crystallized against Tris/HCl 100mM pH 8.5, $(NH_4)_2PO_4$ 200 to 300 mM, MPD 36 to 43% (v/v), or against Hepes/NaOH 100mM pH 6.5, $(NH_4)_2PO_4$ 150 mM, MPD 35 to 40%. Attempts to crystallize complexes at MPD concentrations below 32% (v/v) have not yield crystals in our hands.

X-ray diffraction data were collected at 100K at the European Synchrotron Radiation Facility (Grenoble, France) on ID23-1 beamline (complexes containing peptides **1c**, **2c**, and **4c**) and on ID23-2 (VEGF:**3c** complex). Diffraction data were integrated using XDS, scaled and merged using Scala (CCP4i).[2] The structures were solved by molecular replacement from the VEGF structure model (PDB id: 1FLT) and then refined using Phenix suite (version 1.18_3855).[3]

1.4 Spectrometric methods

Circular Dichroism

CD experiments were performed on a Jasco Model spectropolarimeter at 25°C. Spectra were recorded in a 0.1 cm cell between 190 and 260 nm with a bandwidth of 2.0 nm, response time of 2 seconds and resolution step width of 0.5 nm. Each spectrum represents the mean residue ellipticity (deg.cm^{-2}.dmol^{-1}) averaged on five scans and calculated over 15 residues. Peptide samples were dissolved in distilled water (stock solution: 500 µM). Each stock solution was then diluted five times with 10 mM phosphate buffer pH 7.4 to obtain a final concentration of 100 µM. The accurate final concentrations were determined by UV with molar extinction coefficients calculated using literature data ($\varepsilon_{280\ nm}$ = 11 225 L.mol^{-1}.cm^{-1}).[4]

NMR

NMR samples were prepared by dissolving 2 to 3 mg of each lyophilized peptide in 500 µL of a mixture of $H_2O/D_2O/CD_3CN$ in a molar ratio 8:1:1. Deuterated acetonitrile was used to ensure complete solubility of the bicyclic peptides and for convenient external calibration of chemical shifts. The resulting pH was between 3.5 and 4.5 for all samples. NMR spectra were recorded on Bruker spectrometers operating at 400 or 600 MHz (^{1}H frequencies) and were internally referenced to the residual acetonitrile co-solvent signal at 2.06 ppm. Spectra were recorded at four different temperatures (298, 303, 308 and 313 K). For all experiments, the temperature was controlled externally using a temperature control system. Water peak suppression was achieved by a 1D “excitation sculpting” pulse sequence (zgesgp in the Bruker pulse sequence library) using 180° water-selective pulses. The transmitter frequency was set to the water signal. Two-dimensional spectra TOCSY (total correlated spectroscopy) and NOESY (nuclear Overhauser effect spectroscopy) were recorded with mixing times of 90 ms and 120 ms, respectively. These spectra were acquired with 2048 real points in t2, a spectral width of 7246 Hz, and 256 t1 increments. All spectra were recorded with 128 scans. All data were processed using the software Topspin from Bruker and analyzed with Topspin and CcpNmr Analysis.[5]

Chemical shift index

The chemical shift index (CSI) was calculated as the deviation from random coil values: (measured chemical shift of Hα) minus (chemical shift of Hα in a random coil conformation). The random-coil value of leucine has been used also for norleucine CSI.[6] The CSI can be an indication of the presence of secondary structures in a polypeptide chain, in particular α-helices and β-strands.[7], [8], [9] For Hα protons, an upfield shift relative to that of the random-coil conformation reports α-helix fold, whereas a downfield shift shows β structures.

NMR structure calculations

Peptide structures were calculated based on NOE distance restrains with XPLOR-NIH.[10], [11] A conservative approach was chosen to allow for the maximum number of possible conformations to be identified: pseudo-atom corrections were introduced on all diastereotopic protons and methyl groups[12] a short mixing time of 120 msec was chosen, and upper-bound limits of violated NOE constraints were increased after visual inspection of some correlation spots if signal overlapping was suspected. XPLOR patches were made in-house to describe the modified amino acids, and the lactam bridge between residues P10 and P14 (lald, lale, and nle are available in the supporting information). For each peptide, 200 structures were calculated with the refine.py python protocol,[13] which implements a simulated annealing calculation with the CHARMM forcefield using the NOE restraints in a .tbl file format. Mostly default parameters were chosen. Twenty structures with the lowest energies were selected for statistical analysis. Final structures had no NOE violation greater than 0.2 Å and less than 5% geometry violations. The conformers and NOE constraints were visualized with VMD-XPLOR, and figures were produced with PyMol. The 20 lowest energy NMR structures obtained for each peptide were clustered with the XPLOR-NIH helper program *findClusters*.

1.5 ITC assays

ITC assays were carried out on a Microcal™ ITC200 system at 20° C or 37° C. The cell volume was measured with two direct and two reverse titrations of aqueous solutions of $BaBr_2$ and 18-crown ether-6 (CAS 17455-13-9) at 20°C (Figure S9 and table S8).[14]

For peptide-VEGF titration, the two lyophilized components were dissolved in the following buffer: 50 mM HEPES/NaOH, 150 mM NaCl, pH adjusted to 7.5. Stock solutions were centrifuged at 16,000 rcf during 10’ then filtered through 0.1 µm filters. The concentrations of peptide and protein, and the design of titration were best adjusted to meet the following three requirements: i) optimal ‘c value’ close to 30 – 500 ii) inflection curve centered on half-term of titration iii) a second titration peak whose absolute value was close to 0.5-1.5 µcal.s^{-1} in order to achieve a high signal/noise ratio. Both protein and peptides solutions were degassed 10 min, and their concentrations were measured by UV absorption without further dilution (triplicate measures in 1 cm length path crystal cuvette or 0.5 mm nanodrop cell depending on the concentration). The dried Microcal™ cell was rinsed and filled with the titrated solution. After the filling of the syringe and the cell, the concentrations were measured again on the rest of both solutions. The average of UV measurements was kept as a best estimate of the concentration values. ITCs with relative concentration differences on the injected compound greater than 2.5% were rejected.

To avoid the first injection anomaly, 0.2 µl of titrant was withdraw from syringe before plugging the syringe into the cell reactor.[15] A typical measurement was made of 0.2 µl first injection followed by 15 × 2.54 µl successive microinjections of titrant, with a spacing of 200 s between injections, stirring of 800 r.p.m., and reference power of 5 µcal.s^{-1}. Titrations with a baseline signal (DP) outside of 5.0 ± 0.2 µcal.s^{-1} were discarded. The heat of ligand dilution was corrected from the base line measured after the cell molecule saturation at the end of titration. To explore the potential cooperativity of the two symmetrical binding sites of VEGF, and record thermodynamic data with reliable statistics, both direct and reverse titration of the protein molecule by the ligand were repeated.

ITC data were integrated into NITPIC (version 1.1.15).[16] Analysis of single or pooled titrations were done with SEDPHAT,[17] using the “two symmetrical sites” model of binding (the binding model was described in ref. [1]). The fixed (2:1) stoichiometry implied to refine a concentration correction factor for each titration. We normalized the enthalpy of binding by fixing the correction of protein concentration for reverse titration to 1.000. The confidence

intervals were estimated at confidence level P = 95% with F-statistics based contours of the error surface implemented in SEDPHAT, with titrant molecule correction factor set, base line and correction factor of the titrated molecule being floating. Gibbs free energy of binding was calculated from Van't Hoff equation (eq. 1 in the main manuscript). The resulting entropic contribution was calculated from Gibbs equation (eq. 2 in the main manuscript). As ΔG and ΔH may be correlated, the uncertainty of TΔS was calculated as the result of the propagation of the measurement uncertainties of ΔG and ΔH, *i.e.* Tδ(ΔS) = δ(ΔG) + δ(ΔH). For thermodynamic comparison of independent peptides, uncertainty was estimated with a 95% confidence level from variance formula: $s_f^2 = \sqrt{\sum_i \left(\left(\frac{\partial f}{\partial x_i}\right)^2 . s_{x_i}^2\right)}$

2 Additional discussions

2.1 Circular dichroism spectra of unbound peptides

The CD spectra of peptides differ from those of proteins. Except under conditions where particular secondary structures are predominant, algorithms of CD spectra deconvolution are not usable for peptides.[18] Indeed, the chiroptic properties of the short α-helices differ from the longer ones present in proteins, the great flexibility of peptides also results in a rapid equilibrium of conformations, and aromatic residues can significantly contribute to CD spectra in far UV.[19] In the present case, folded structures of the VEGF-bound peptides comprised six residues structured in helix, four in β-turn and five forming an extended structure. If such a conformation was present for the unbound peptides, and considering that the β-turn and the extended part had low intensity CD patterns, we expected the spectra to be dominated by the helix signature.

The CD spectra of **1c**, **2c** and **3c** clearly displayed characteristic helix signatures, unlike the monocyclic and **4c** peptides. However, the accurate interpretation of monocyclic and **4c** peptides spectra was more ambiguous: although some groups initially observed that CD signature of 3_{10} helix was indistinguishable from α-helix,[20], [21] Toniolo *et al.* proposed that right-handed 3_{10} helical peptides were characterized by a strong negative minimum in the 202-206 nm region[22], [23] accompanied by a pronounced negative shoulder centered at 222 nm. More recently, others have only observed an increase in the 208 nm / 220 nm ratio, without shifting the spectrum toward the short wavelengths.[24] Therefore, from qualitative and quantitative elements, it can be proposed that a short 3_{10} helical structure and random coil contributed to monocyclic and **4c** peptides spectra.

2.2 NMR structures of unbound peptides

Proton amide chemical shift variation with temperature.

Stable H-bonds were identified by proton amide chemical shift variation with temperature. In aqueous solutions, hydrogen atoms involved in hydrogen bonds are usually less sensitive to temperature variations than solvent exposed ones. They are therefore identified if their |Δδ/°C| is < 4 ppb/°C, whereas non H-bonded protons have |Δδ/°C| values > 6 ppb/°C.[25] Variation of NH signals chemical shifts as a function of temperature for the eight peptides is given in Table S2.

More hydrogen atoms involved in stable H-bonds were measured for peptides **1c**, **2c**, and **3c** than for the corresponding monocyclic ones. In contrast, the bicyclic peptide **4c** appeared very similar to the monocyclic peptide **4**. Values of Δδ/°C ≥ 0 were observed for **1c** and **3c**, indicating the existence of very strong H-bonds at the N-terminal β-turn (Val P5) and for some residues of the C-terminal helix (Cys P11 and Leu P15). The H-bond network seemed to extend to at least the NH of Cys P11, which could therefore be H-bonded to the carbonyl or carboxylic group of Glu P8 (i to i+3) or the carbonyl of Trp P7 (i to i+4). The H-bonding of amide protons of P13 to P15 was expected in stable α-helix. However, the amino acids Phe P12 / Glu P13 / Asp P14 of the helix segment in **1c** did not seem to be involved in strong H-bonds. We also noted that the NH of Leu P15 appeared here to be strongly H-bonded for the bicyclic peptides **1c**-**3c**, unlike the NOE-based structure determination and X-ray analysis, which showed that this Leu P15 was agitated. The monocyclic peptides and the bicyclic peptide **4c** had only a tendency to form H-bonds in this C-terminal part (Δδ ~ -4 to -6 ppb/K).

Some hydrogen bonds were rather stable and temperature resistant in monocyclic peptides, implying Cys P11, Lys/ Glu or Asp P10 and Val P5. The latter may imply the existence of a β-turn, similar to the bicyclic peptides, with a H-bond between NH Val P5 and CO Asp P2. The Glu P8 NH bond was also found to be particularly stable in mono and bicyclic peptides, except for peptide **3**. In the crystal and NMR structures of the linked peptides, it can only be linked to Cys P11 sulphur atom at 3.2 Å with a weak electrostatic interaction.

NMR structures determination

The structures of the peptides in solution were determined by simulated annealing with XPLOR-NIH with NOE constraints. However, attempt to solve their structures resulted either in significant restraints violations with unmodified restraints lists, or in an enlarged conformational landscape compatible with NOE constraints after loosening the constraints with extensive pseudo-atom corrections and conservatively taking into account potential overlaps of NOE cross-peaks (see experimental section). Results obtained without any significant restraint violations are presented below.

The backbone RMSD of the 20 lowest energy NMR structures compared to the crystal structure were calculated for each peptide (Table S4), indicating that the bicyclic **2c** and **3c** were significantly closer to the folded structures than the other peptide molecules. However, the segmentation of the structure of the peptides bound to VEGF in short well-defined secondary elements made the comparison to unbound peptides more meaningful. Indeed, it allowed the evaluation of the presence of these short secondary structures without taking into account their relative orientations. The β-turn P2-5 was the most conserved element with RMSD values between 1.2 and 1.6 Å. The RMSD values of the P6-9 fragment were also in a narrow range for all the peptides, but slightly higher (between 1.4 and 2.0 Å). The largest variability was observed in the α-helix P10-14. The values of 1.1 and 0.6 Å for **2c** and **3c** indicated that the helix was similar to the crystal structure. For the bicyclic peptide **1c** significantly higher value of 1.8 Å was obtained, possibly because some key NOE correlations between NH protons of the helix were invisible, being too close to the diagonal. Large values for the monocyclic peptides and **4c** indicated that they were less structured in α-helices than the bicyclic peptides.

The 20 lowest energy NMR structures obtained for each peptide were clustered with the XPLOR-NIH helper program *findClusters*. Backbone atoms N, CA, C and O were selected for the RMSD calculations with a range of cut-off values from 0.8 to 1.8 Å (Table S5). Clustering over the whole sequence did not allow the identification of major clusters, because of the flexibility of the

central P6-9 loop. Therefore, a clustering according to each of the three secondary structures was performed.

In the β-Turn (amino acids P2-5), several clusters were obtained for each peptide. Further examination of the conformations revealed some flexibility of this sequence, with the presence of different types of turns and extended conformations (Fig. 1d). We noted here that the presence of extended conformations in the bicyclic peptide **1c** and **3c** seemed in contradiction with variable NMR temperature measurements showing a strong hydrogen bond involving the NH of Val P5. However, we did not include hydrogen bond restraints in these calculations, which were only based on NOE restraints. Conformations matching a type I β-turn, corresponding to the turn observed in the crystal structure, were present among the solution structures of all the peptides. This observation agreed with the NOE observed between NH His P4 and NH Val P5 in the range 2.4 - 3.1 Å for all the peptides (2.5 Å in a canonical type I β-turn), and NH Ile P3 and NH His P4 observed in most peptides in the range 2.6 - 3.4 Å (2.9 Å in a type I β-turn).

The extended fragment (amino acids P6-9) had a similar range of RMSD values versus the crystal structure for all the peptides (between 1.4 - 2.0 Å, mostly 1.5 - 1.6 Å, Table S4). There was no noticeable difference or characteristics among the peptides. However, a remarkable change in the orientation of this fragment was observed between structures of unbound and bound peptide. In the NMR and crystal structures of VEGF-bound peptides, amino acids P1-11 were wrapped around the side chain of Trp P7, that formed the nucleus of an apolar cluster, with the indole being packed against Ile P3 and Phe P12 side chains and against the disulfide bridge. In the NMR structures of unbound peptides, superposition over the more structured fragments P2-5 and P10-14 demonstrated that interacting residues of the extended fragment (^{n}Leu P6 and Trp P7) were always in very different orientations. An example is given for Trp P7 of peptide **3c**, which shows the side chain folded in the hydrophobic core of the peptide in the crystal structure, whereas it was completely in reverse orientation in almost all the NMR structures (Figure 1d). Concurrently, in all the 20 lowest energy NMR structures of the peptides, the indole side chains were oriented out of the P1-11 macrocycle and were exposed to the solvent. Among all the peptides, a NOE correlation was observed between Trp P7 NHε3 and Hα (except peptide **3** for which the expected correlation was apparently overlapped with lysine NHζ signals), corresponding to calculated interproton distances between 2.8 and 3.3 Å. The distance was approximately 5.2 Å in the crystal structures in complex with VEGF, indicating that the conformations of the Trp P7 indole side chain of the unbound peptides were not compatible with the folded VEGF-bound structures. Moreover, all the NH(i) - NH(i+1) distances measurable by NOE on this extended fragment residues P6-9 were in the range 2.7 - 3.3 Å, whereas in the crystal structures these distances were larger than 4.3 Å. Therefore, the binding of the peptide to the VEGF molecule required a reorganization of the backbone and corresponding side chains involving at least residues P6 to 8.

As expected from the design, the helices (amino acids P10-14) of bicyclic peptides **1c**, **2c** and **3c** were stabilized, compared to monocyclic control peptides. In particular, helical folds were observed in most structures for **2c** and **3c**, resulting in a single α-helical cluster for a RMSD value of 1.2 Å. At this cutoff, **1c** had 6 clusters, showing a larger flexibility in this segment than **2c** and **3c**, whereas **4c** and the monocyclic peptides had between 9 and 16 clusters (Table S5).

2.3 Analytical requirements in ITC analysis

The attempt to rationalize the thermodynamic data for a series of related protein-ligand complexes required careful design and measurement of isothermal titrations, followed by estimation of data uncertainties. It was particularly important to minimize the enthalpy errors to obtain reliable entropy values: as ΔG is less prone to errors than ΔH, due the logarithm term in eq. 1, the error on TΔS mainly propagates from ΔH errors according to the Gibbs equation (eq. 2). In ITC analysis, if the binding stoichiometry or the cell concentration can float, the calculated enthalpy varies almost proportionally to the concentration in the syringe for high Wiseman parameter (*c*-value = n.[M_0]/K_d, with n the number of sites per receptor molecule M_0 in the cell) and ΔH precision is directly related to the uncertainty of the injected molecule concentration.[26], [27], [28] Consequently, the measurement of titrant molecules concentrations by UV absorption without further dilution made it possible to achieve a relative precision on the concentrations (1 S.D. (C)/ C) of less than 3%. Additionally, to obtain a robust statistic, we replicated the ITC measurements by comparing direct and reverse titration, which measured peptide binding to VEGF and, conversely, VEGF binding to peptide molecules. Enthalpies of binding per mole of peptide or per mole of VEGF binding site should theoretically be equal. This was not strictly the case and we observed in the preliminary data that the ΔH per mole may also vary slightly from batch to batch, despite any obvious sign of impurity in the analysis (HPLC, SEC, SDS page or mass spectrometry). We then decided to further purify batches of peptides by HPLC, and to normalize the data according to the molar concentration of VEGF binding sites and not the peptide site, because VEGF was the only reagent common in all the titrations.

For each peptide, the titrations were globally analyzed in SEDPHAT,[17] using a fixed stoichiometry but floating cell concentration correction factors and baselines. However, due to material constraints, we had to use three different batches of lyophilized protein, which resulted in three sets of ΔH in reverse titration at 20°C, when the titrations were analyzed individually. This was due to differences in the proportion of fully functional protein, the so-called "competent fractions" of protein. The batch n°3 was the most active and its competent fraction was fixed to 1.000. The competent fraction of the two other batches were determined from the average value of the ratio of <ΔHbatch n°i> / <ΔHbatch n°3> calculated over 10 mean values of independent protein-peptide pairs. This resulted in competent fraction of 0.976 (S.D. 0.033) for batch n°1, and 0.879 (S.D. 0.052) for batch n°2 (Tables S7.a-c). The titrations measured at 37°C were almost completely done with the batch n°3 and confirmed the data recorded at 20°C.

The analysis of each peptide/protein pair was done using the model of "two symmetrical sites" of SEDPHAT, without cooperativity effect. To compare the different peptides, the enthalpy of binding was normalized by setting the correction factor of protein concentration for reverse titration to 1.000. Under these conditions, the resulting concentration correction factor of each peptide for direct injection (peptide injected in protein solution) was refined close to 0.997 (S.D. 0.078). This value indicated that the mean ΔH per binding site measured in the direct and reverse titrations was the same but that the correction factor was significantly scattered across the peptides. It gave a high confidence in the measurements of ΔH and a first estimate of the uncertainties. However, small deviations can result from imprecision in the molar absorption coefficients of peptides due to their folding differences and / or from a poor estimate of the

competent local peptide fraction, which has not been corrected. The scatter of results from direct titrations also demonstrated that reliable enthalpies cannot be determined by direct titration (peptide injected into protein) and that reverse titration (protein to peptide) was necessary to compare small differences in thermodynamics. However, whatever the orientation of the titration (direct or reverse), the average of the titrate correction factor in the cell was equal to 0.885 (S.D. 0.056) instead of 1.000. This remains an open question but may reflect small differences in heat capacity between the titration cell and the reference cell or an inaccurate cell volume that has not significant impact on the precision of ΔH and Kd.[14]

Finally, confidence intervals were estimated at confidence level P = 95%. Note that the densities of probability of Kd and ΔH were not Gaussian and that the mean relative errors at P = 0.95 or P = 0.653 were relatively close: for P = 0.95 the mean uncertainties were $<\delta K_d / K_d>$ = 15%, $<\delta(\Delta H)/\Delta H>$ = 2.5%, that gave $<\delta(\Delta G)/\Delta G>$ = 0.9%, and $<\delta(T\Delta S)/T\Delta S>$ = 5.9%, and for P= 0.653, the mean uncertainties were $<\delta K_d / K_d>$ = 12%, $<\delta(\Delta H)/\Delta H>$ = 2.0%, that gave $<\delta(\Delta G)/\Delta G>$ = 0.8%, and $<\delta(T\Delta S)/T\Delta S>$ = 4.9%.

2.4 Sequence effects in monocyclic peptides

Peptides **1** and **4** had a Lys residue in position P10 but differed in residues Asp and Glu in position P14, respectively. Their Gibbs free energies of binding to VEGF were equal ($\Delta G_1^{20°C} - \Delta G_4^{20°C}$ = 0.2 ± 0.1 and $\Delta G_1^{37°C} - \Delta G_4^{37°C}$ = 0.2 ± 0.1 kcal.mol^{-1}, confidence level P = 0.95), but resulted from slight differences of enthalpy and entropy of binding ($\Delta H_1^{20°C} - \Delta H_4^{20°C}$ = -0.4 ± 0.6 and $\Delta H_1^{37°C} - \Delta H_4^{37°C}$ = 0.2 ± 0.8 kcal.mol^{-1}, $-T(\Delta S_1^{20°C} - \Delta S_4^{20°C})$ = 0.6 ± 0.7 and $-T(\Delta S_1^{37°C} - \Delta S_4^{37°C})$ = 0.0 ± 1.0 kcal.mol^{-1}).

Peptides **2** and **3** had Lys residue in position P14, and Asp or Glu at position P10, respectively. Their Gibbs free energy of binding to VEGF were equal ($\Delta G_2^{20°C} - \Delta G_3^{20°C}$ = 0.3 ± 0.2 and $\Delta G_2^{37°C} - \Delta G_3^{37°C}$ = 0.3 ± 0.1 kcal.mol^{-1}), but resulted from differences of enthalpy and entropy of binding ($\Delta H_2^{20°C} - \Delta H_3^{20°C}$ = 1.4 ± 0.6 and $\Delta H_2^{37°C} - \Delta H_3^{37°C}$ = 1.0 ± 0.7 kcal.mol^{-1}, $-T(\Delta S_2^{20°C} - \Delta S_3^{20°C})$ = -1.0 ± 0.8 and $-T(\Delta S_2^{37°C} - \Delta S_3^{37°C})$ = -0.6 ± 0.8 kcal.mol^{-1}). Overall, the sequence inversion KxxxD/E (peptides **1** and **4**) to D/ExxxK (**2** and **3**) led to differences in binding thermodynamics between peptides, which resulted in $\Delta S_1 \approx \Delta S_4 < \Delta S_3 < \Delta S_2$ and $\Delta H_1 \approx \Delta H_4 \approx \Delta H_3 < \Delta H_2$. When it was significant, the entropy variation was more favorable for inversion **1** to **2** than **4** to **3** ($-T.(\Delta S_2^{20°C} - \Delta S_1^{20°C})$ = -2.0 ± 0.7 and $T.(\Delta S_2^{37°C} - \Delta S_1^{37°C})$ = -1.0 ± 0.8 kcal.mol^{-1}) and ($-T.(\Delta S_3^{20°C} - \Delta S_4^{20°C})$ = -0.5 ± 0.7 and $T.(\Delta S_3^{37°C} - \Delta S_4^{37°C})$ = -0.4 ± 1.0 kcal.mol^{-1}), but it was partially compensated by unfavorable enthalpy variation ($\Delta H_2^{20°C} - \Delta H_1^{20°C}$ = 2.0 ± 0.6 and $\Delta H_2^{37°C} - \Delta H_1^{37°C}$ = 0.7 ± 0.7 kcal.mol^{-1}) and ($\Delta H_3^{20°C} - \Delta H_4^{20°C}$ = 0.2 ± 0.6 and $\Delta H_3^{37°C} - \Delta H_4^{37°C}$ = 0.0 ± 0.9 kcal.mol^{-1}).

In the VEGF-bound state, residues P10 and P14 of monocyclic peptides were part of the C-terminus helix, which had formed upon binding. They were located on the peptide face opposite to the binding site to at least 9 Å of the nearest VEGF residue side chains, ruling out any short-range electrostatic interaction. Therefore, the binding interface was unchanged throughout the monocyclic peptide series, that suggest the thermodynamics differences must primarily by related to the folding energy of the peptide that corresponds to the $\Delta J_P = J_{P2} - J_{P1}$ term in the Figure 4.

In contrast, NMR data and CD spectra measured across the monocyclic peptide molecules suggested that the four peptides had few secondary structures in solution. In particular, the helicity estimated from CD spectra showed that the α-helix was not folded in solution (Table S1). NMR data confirmed CD measurement: the Hα CSI indicated that P9 to P15 were in random-coil conformation (Figure 1c). Although fast exchange conformers were expected, some strong H-bonds were noted from the chemical shift of proton amide with temperature (Table S3), which suggested however some preferential folds of the P8-15 backbone in solution which differed from the bound structures. In particular, the amide protons of Glu P8, K/E/D P10 and Cys P11 were H-bonded. The strength of the NH-bonds was higher for Asp or Glu in position P10 (peptides **2** and **3**) than Lys in the same position (peptides **1** and **4**). Likewise, the strength of the P14 NH-bond was higher for Asp or Glu at P14 position than Lys at the same position. Such observations showed that the sequence changes affected the conformation equilibriums of unbound monocyclic peptides.

A few leads can be suggested in an attempt to rationalize thermodynamics from these structures: it is known that D/ExxxK is capable of stabilizing α-helical peptides regardless of their orientation in a manner dependent of pH and salt concentration, which leads to the conclusion of an electrostatic effect of the charged residues capable of making a salt-bridge between side chains or/and interacting with the helix macrodipole.[29], [30] In this study, we first observed that peptide **2** and **3** bound VEGF with a higher affinity than **1** and **4** respectively, suggesting that the D/ExxxK orientation was more favorable than the reverse orientation. This result agreed with the observations of others in two independent studies on helix folding: at low ionic strength and neutral pH, the helix fold is favored by E_i/K_{i+4} conformation compared to K_i/E_{i+4}, in17 amino acids-long peptides series with three E/K pairs.[31], [30] A similar result is recorded from 15-mer with a single E/K pair,[29] but in this study K_i/D_{i+4} is more stabilizing that D_i/K_{i+4}. Interestingly, a compilation of salt bridges observed in the Protein Data Bank (PDB) shows that K_i/D_{i+4} on helices has a frequency significantly higher than any other combination of charged residues.[32]

The D/ExxxK orientation (**2** and **3**) was entropically more favorable than KxxxD/E (**1** and **4**). In the peptide series, the conformational spaces accessible by the P10 and P14 side chains were reduced by the peptide folding upon binding. The number of degrees of freedom of the residue in P10 position was strongly constrained by close van der Walls contacts with neighboring residues Glu P13, Glu P8, Cys P11, Trp P9 and the residue P14. In contrast, position P14 was located at the C-terminus of the folded α-helix, away from VEGF and from the peptide core. Therefore, the conformational space accessible to the P14 residue side chain was larger than at P10 position. As the number of degrees of rotation decreased from Lys to Glu and Asp side chains (4, 3 and 2 rotamers respectively), it was plausible that burying of Lys in position P10 had a more negative consequence on conformational entropy compared to the effect of Asp and Glu in the same position. In addition, since Glu differed from Asp by a single rotamer, the entropy of folding was less unfavorable for peptides **2** than **3** (Asp P10 and Glu P10 respectively). Note that the number of rotamers observed in the PDB for Lys, Glu and Asp are 27, 8 and 5 respectively, and when restricted to helix, 94% of Asp residues correspond to only two rotamers.[33] Our experimental observation was consistent with the order of magnitude of the loss of entropy of a single rotatable bond or with the change in side-chain conformational entropy upon protein folding reviewed by Doig *et al*.[34], [35]

The enthalpy difference was not significant for ExxxK (**3**) compared to the opposite orientation (**4**). Similarly, Meuzelarr *et al.* show that the folding enthalpy of Glu_i/Lys_{i+4} in Ac-A(EaaaK)$_3$A-NH_2 α-helical peptides are not significantly different compared to the opposite orientation.[31] In contrast, DxxxK (**2**) enthalpy of

binding was less favorable that the opposite orientation (**1**). To our knowledge, this interaction had not been previously quantified in helical peptides. It is possibly an example of EEC, if the conformational restraints on KxxxD conformation increased the probability of the salt bridge formation as compared to the opposite orientation.

3 References

[1] M. Reille-Seroussi, J. F. Gaucher, C. Desole, N. Gagey-Eilstein, F. Brachet, I. Broutin, M. Vidal, S. Broussy, *Biochemistry* **2015**, *54*, 5147–56.

[2] E. Potterton, P. Briggs, M. Turkenburg, E. Dodson, *Acta Crystallogr. D Biol. Crystallogr.* **2003**, *59*, 1131–7.

[3] D. Liebschner, P. V. Afonine, M. L. Baker, G. Bunkoczi, V. B. Chen, T. I. Croll, B. Hintze, L. W. Hung, S. Jain, A. J. McCoy, N. W. Moriarty, R. D. Oeffner, B. K. Poon, M. G. Prisant, R. J. Read, J. S. Richardson, D. C. Richardson, M. D. Sammito, O. V. Sobolev, D. H. Stockwell, T. C. Terwilliger, A. G. Urzhumtsev, L. L. Videau, C. J. Williams, P. D. Adams, *Acta Crystallogr D Struct Biol* **2019**, *75*, 861–877.

[4] C. N. Pace, F. Vajdos, L. Fee, G. Grimsley, T. Gray, *Protein Sci.* **1995**, *4*, 2411–23.

[5] W. F. Vranken, W. Boucher, T. J. Stevens, R. H. Fogh, A. Pajon, M. Llinas, E. L. Ulrich, J. L. Markley, J. Ionides, E. D. Laue, *Proteins* **2005**, *59*, 687–696.

[6] S. Olofsson, L. Baltzer, *Folding and Design* **1996**, *1*, 347–356.

[7] D. S. Wishart, B. D. Sykes, F. M. Richards, *J. Mol. Biol.* **1991**, *222*, 311–333.

[8] D. S. Wishart, B. D. Sykes, in *Methods in Enzymology*, Elsevier, **1994**, pp. 363–392.

[9] S. Schwarzinger, G. J. A. Kroon, T. R. Foss, J. Chung, P. E. Wright, H. J. Dyson, *J. Am. Chem. Soc.* **2001**, *123*, 2970–2978.

[10] C. D. Schwieters, J. J. Kuszewski, N. Tjandra, G. Marius Clore, *Journal of Magnetic Resonance* **2003**, *160*, 65–73.

[11] C. D. Schwieters, G. A. Bermejo, G. M. Clore, *Protein Sci.* **2018**, *27*, 26–40.

[12] K. Wüthrich, M. Billeter, W. Braun, *J. Mol. Biol.* **1983**, *169*, 949–961.

[13] G. A. Bermejo, C. D. Schwieters, *Methods Mol. Biol.* **2018**, *1688*, 311–340.

[14] J. Tellinghuisen, *Anal. Biochem.* **2004**, *333*, 405–6.

[15] L. S. Mizoue, J. Tellinghuisen, *Anal. Biochem.* **2004**, *326*, 125–7.

[16] T. H. Scheuermann, C. A. Brautigam, *Methods* **2015**, *76*, 87–98.

[17] C. A. Brautigam, H. Zhao, C. Vargas, S. Keller, P. Schuck, *Nat. Protoc.* **2016**, *11*, 882–94.

[18] S. M. Kelly, T. J. Jess, N. C. Price, *Biochim. Biophys. Acta* **2005**, *1751*, 119–39.

[19] N. Sreerama, M. C. Manning, M. E. Powers, J.-X. Zhang, D. P. Goldenberg, R. W. Woody, *Biochemistry* **1999**, *38*, 10814–10822.

[20] T. S. Sudha, E. K. S. Vijayakumar, P. Balaram, *Inter. J. Pept. Prot. Res.* **1983**, *22*, 464–468.

[21] N. H. Andersen, Z. Liu, K. S. Prickett, *FEBS Letters* **1996**, *399*, 47–52.

[22] C. Toniolo, A. Polese, F. Formaggio, M. Crisma, J. Kamphuis, *J. Am. Chem. Soc.* **1996**, *118*, 2744–2745.

[23] C. Toniolo, F. Formaggio, S. Tognon, Q. B. Broxterman, B. Kaptein, R. Huang, V. Setnicka, T. A. Keiderling, I. H. McColl, L. Hecht, L. D. Barron, *Biopolymers* **2004**, *75*, 32–45.

[24] L. Bottorf, S. Rafferty, I. D. Sahu, R. M. McCarrick, G. A. Lorigan, *J. Phys. Chem. B* **2017**, *121*, 2961–2967.

[25] M. Iqbal, P. Balaram, *Biochemistry* **1981**, *20*, 7278–7284.

[26] J. Tellinghuisen, *Anal. Biochem.* **2007**, *360*, 47–55.

[27] J. Tellinghuisen, *J. Phys. Chem. B* **2005**, *109*, 20027–35.

[28] S. A. Kantonen, N. M. Henriksen, M. K. Gilson, *Biochim. Biophys. Acta Gen. Subj.* **2017**, *1861*, 485–498.

[29] J. S. Smith, J. M. Scholtz, *Biochemistry* **1998**, *37*, 33–40.

[30] S. Marqusee, R. L. Baldwin, *Proc. Nat. Acad. Sci. U. S. A.* **1987**, *84*, 8898–8902.

[31] H. Meuzelaar, J. Vreede, S. Woutersen, *Biophys. J.* **2016**, *110*, 2328–2341.

[32] J. E. Donald, D. W. Kulp, W. F. DeGrado, *Proteins: Structure, Function, and Bioinformatics* **2011**, *79*, 898–915.

[33] S. C. Lovell, J. M. Word, J. S. Richardson, D. C. Richardson, *Proteins* **2000**, *40*, 389–408.

[34] C. W. Murray, M. L. Verdonk, *J. Comput. Aided Mol. Des.* **2002**, *16*, 741–753.

[35] A. J. Doig, M. J. E. Sternberg, *Protein Sci.* **1995**, *4*, 2247–2251.

[36] H. N. Hoang, C. Wu, T. A. Hill, A. Dantas de Araujo, P. V. Bernhardt, L. Liu, D. P. Fairlie, *Angew. Chem. Int. Ed.* **2019**, *58*, 18873–18877.

[37] T. E. Speltz, C. G. Mayne, S. W. Fanning, Z. Siddiqui, E. Tajkhorshid, G. L. Greene, T. W. Moore, *Org. Biomol. Chem.* **2018**, *16*, 3702–3706.

[38] T. Okamoto, K. Zobel, A. Fedorova, C. Quan, H. Yang, W. J. Fairbrother, D. C. Huang, B. J. Smith, K. Deshayes, P. E. Czabotar, *ACS Chem. Biol.* **2013**, *8*, 297–302.

[39] I. Wadsö, R. N. Goldberg, *Pure and Applied Chemistry* **2001**, *73*, 1625–1639.

4 Supplemental Figures

4.1 Figure S1. Synthesis of bicyclic peptides.

The detailed scheme is given for **3c**, and similar procedures are used for the synthesis of **1c**, **2c**, and **4c**.

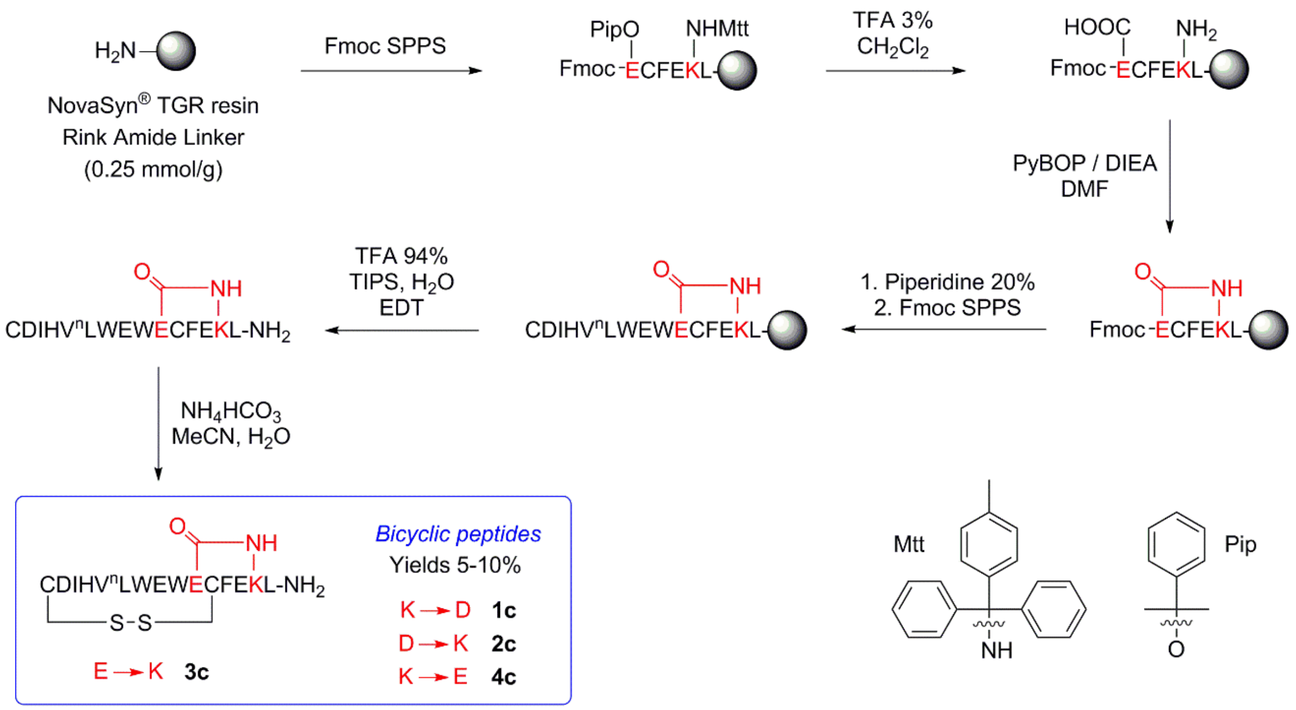

	Sequence	Molecular formula	Molecular weight
**1**	cCDIHVnLWEWKCFEDL-NH2	C90H125N21O23S2	1933.23
**1c**	ccCDIHVnLWEWKCFEDL-NH2	C90H123N21O22S2	1915.22
**2**	cCDIHVnLWEWDCFEKL-NH2	C90H125N21O23S2	1933.23
**2c**	ccCDIHVnLWEWDCFEKL-NH2	C90H123N21O22S2	1915.22
**3**	cCDIHVnLWEWECFEKL-NH2	C91H127N21O23S2	1947.26
**3c**	ccCDIHVnLWEWECFEKL-NH2	C91H125N21O22S2	1929.25
**4**	cCDIHVnLWEWKCFEEL-NH2	C91H127N21O23S2	1947.26
**4c**	ccCDIHVnLWEWKCFEEL-NH2	C91H125N21O22S2	1929.25

4.2 Figure S2. Superposition of the structures of VEGF:peptide **1c** and VEGF in complex with VEGFR1.

Superposition of the structure of peptide **1c** (yellow) and the crystal structure of VEGF-A in complex with VEGFR1-domains 1 to 6 (magenta) (PDB id 5T89). The peptide-binding site overlapped with the binding site of VEGF receptor 1 domain 2. However, most of the **1c** residues in contacts with VEGF showed no homology with VEGFR1-d2.

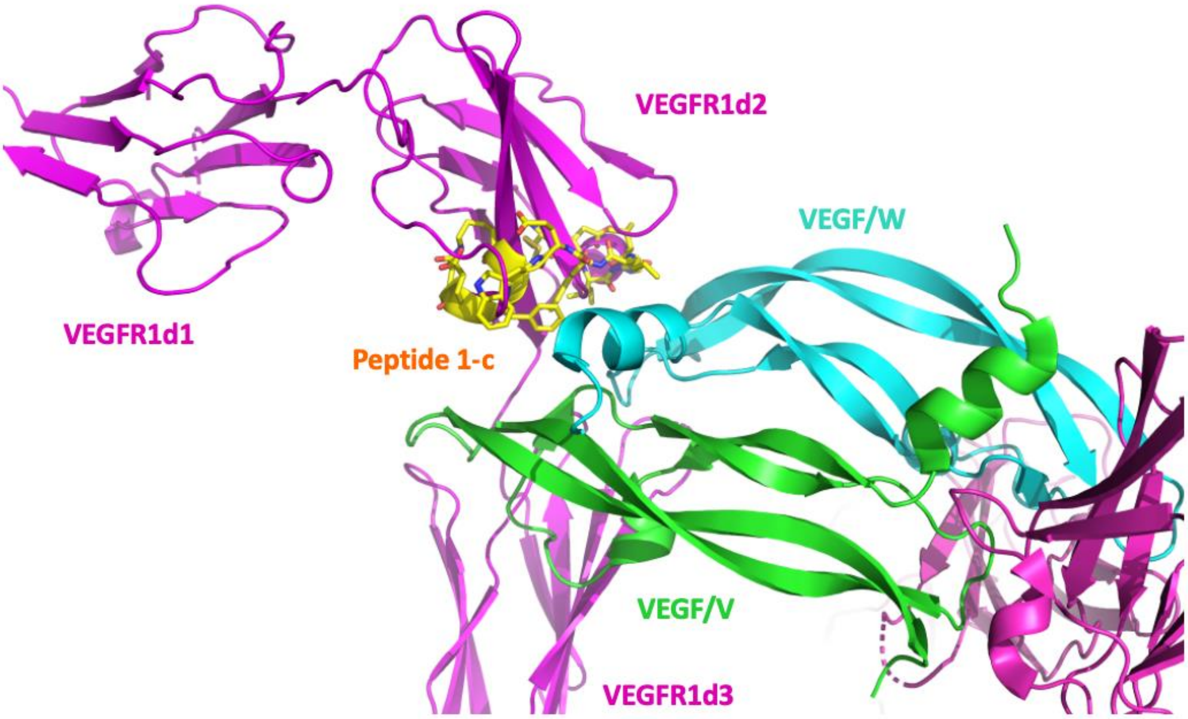

4.3 Figure S3. Orthogonal T-shaped π -stacking.

Orthogonal T-shaped π-stacking of Phe P12 with Trp P7, Phe W17 and Tyr W21, the Phe W17 and Tyr W21 themselves being in a parallel-displaced π-stacking.

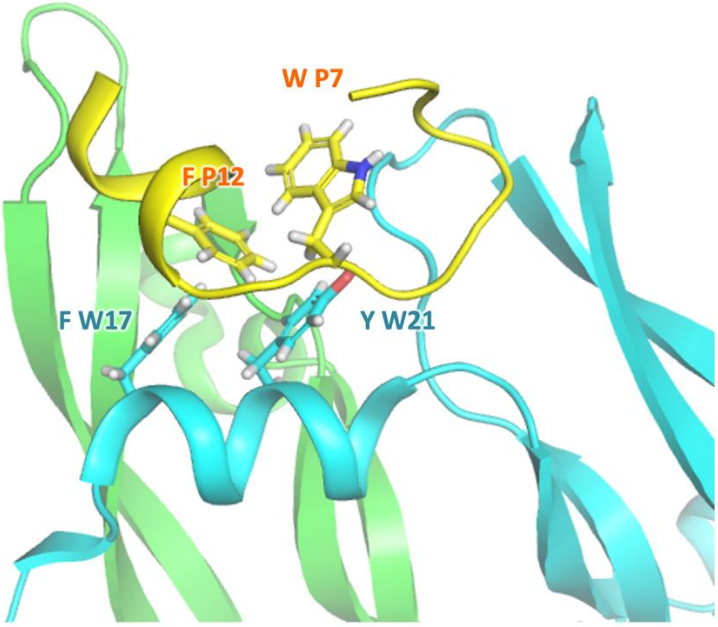

4.4 Figure S4. Structures of the bicyclic peptide helices.

The lactam bridges are in bright colors. The H-bond network is noted in purple dashes.

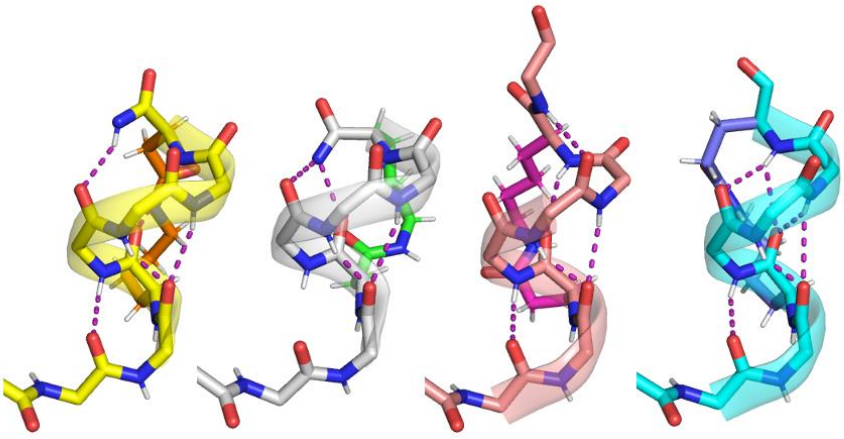

1c 2c 3c 4c

4.5 Figure S5. Detail of **1c** helix structure.

The refined geometry of peptide helix was compatible with the hypothesis of a stabilization by a long-range n $\rightarrow$ π* interaction between the O Lys P10 (n orbital) and CO Asp P14 (π* orbital), which is proposed by Hoang et al.[36]
d = 3.08 Å, θ = 135°

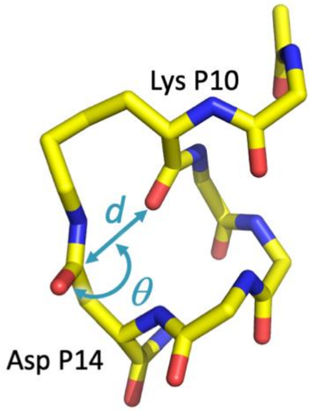

4.6 Figure S6. Representation of the backbones of bicyclic peptides and v107.

On the right, superposition of rotated structures of v107, **1c** and **2c** (up) and v107, **3c** and **4c** (down).

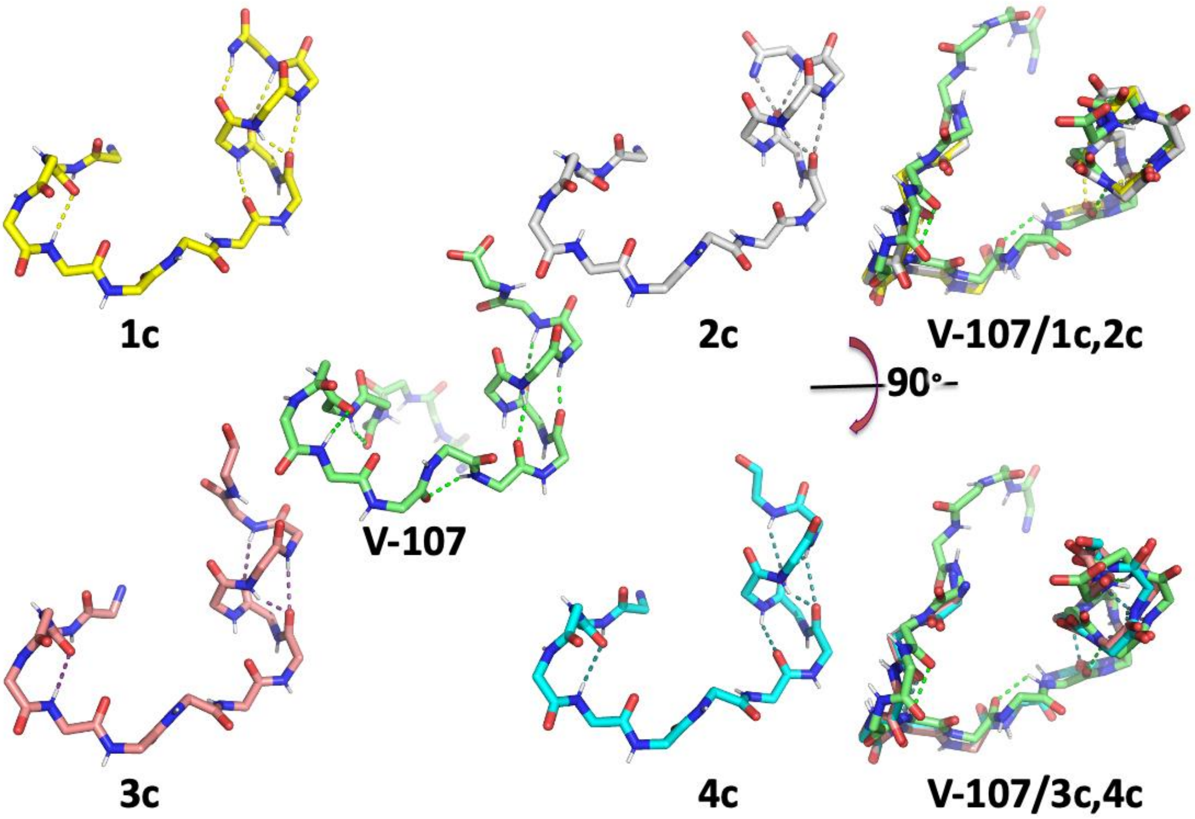

4.7 Figure S7. Superposition of available crystal structures of helices stabilized by lactam bridge.

- (left) KxxxD: peptide **1c** (yellow), PDB id 5WGD[37] (deep purple) and CCDC deposition number: 1941068[36]. (grey). **1c** differed from the two other structures on the χ_3 angle of Lys P10. - (right) ExxxK: peptide **3c** (salmon) and PDB id 2YQ7[38] (violet). **3c** differed from 2YQ7 structures on the χ_1 angle of Glu P10.

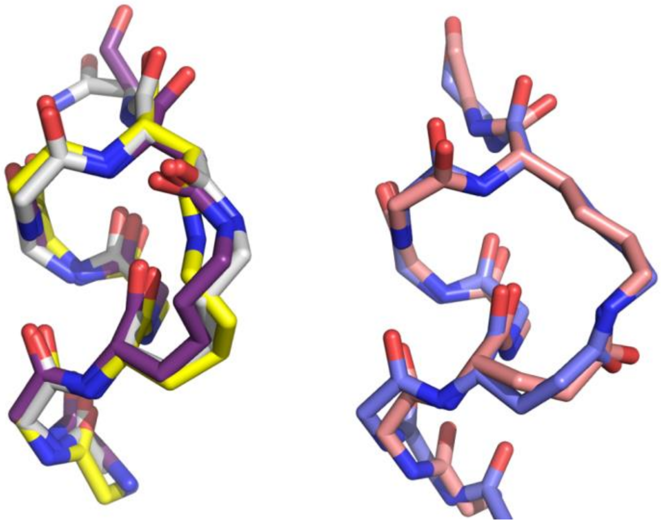

4.8 Figure S8a. Examples of reverse and direct ITC of VEGF by peptides at 20° C.

The data fit curves and the residuals were calculated with the parameters resulting from the overall refinement.

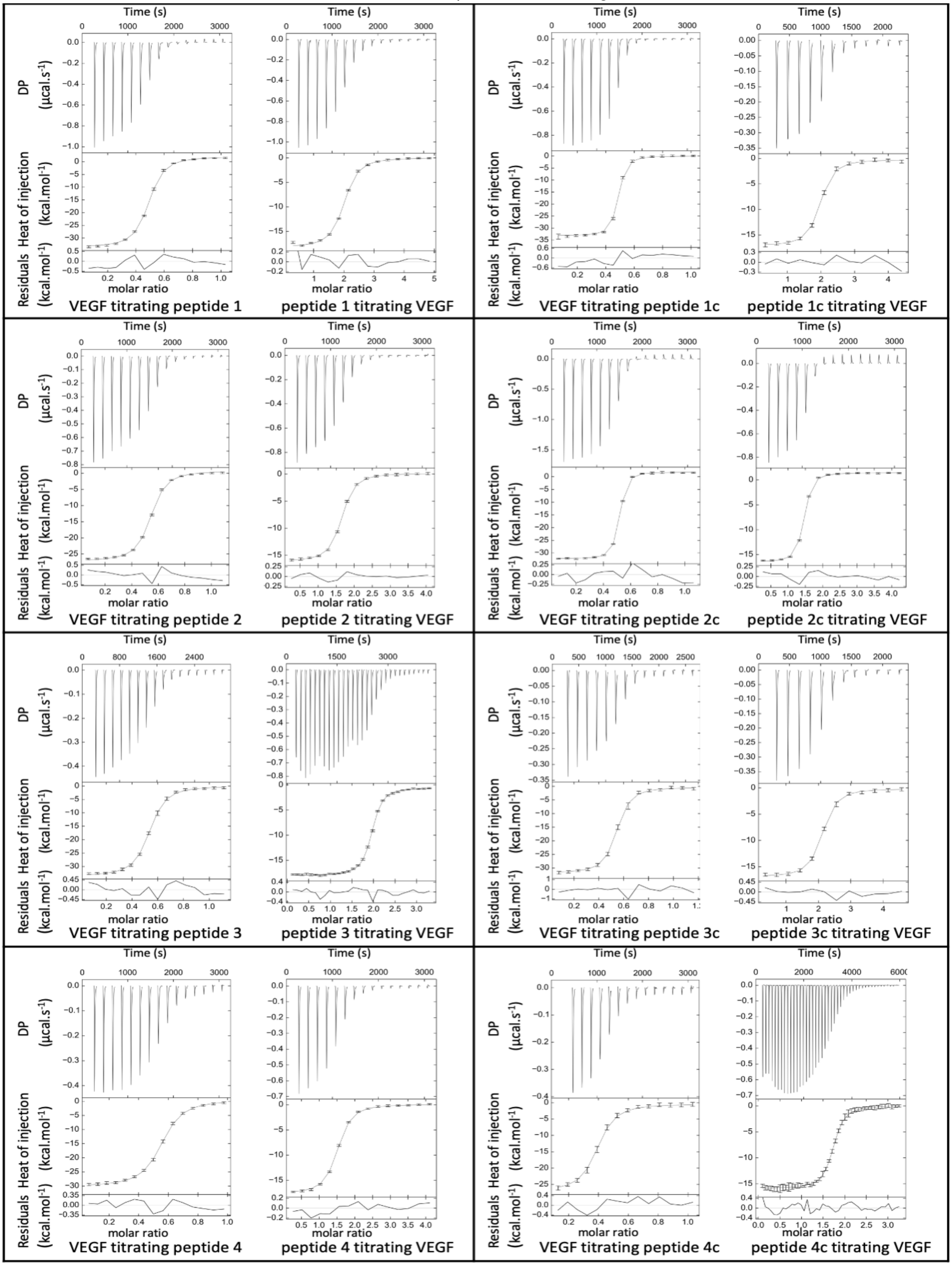

4.9 Figure S8b. Examples of reverse and direct ITC of VEGF by peptides at 37° C.

The data fit curves and the residuals were calculated with the parameters resulting from the overall refinement.

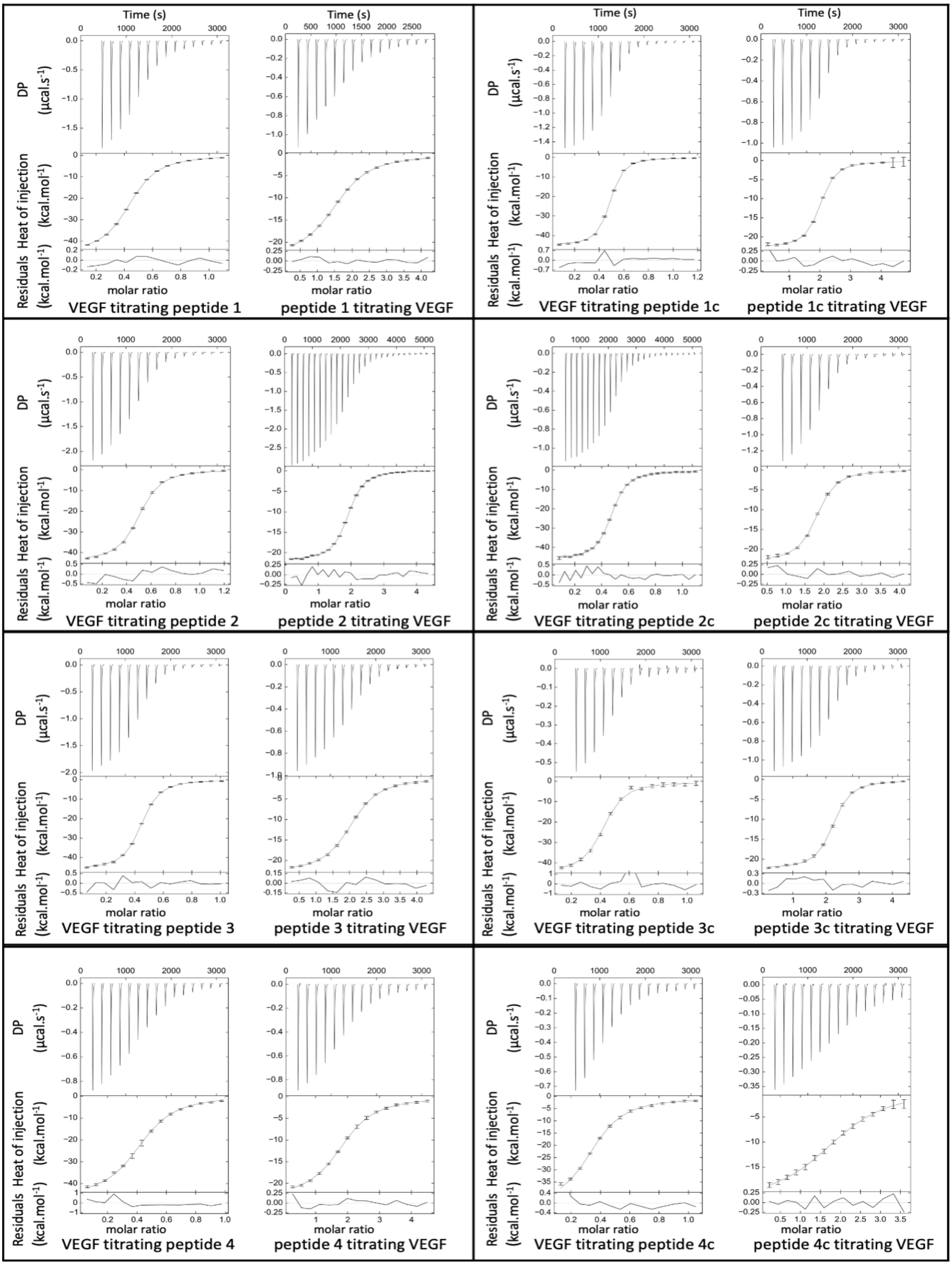

4.10 Figure S9. Examples of reverse and direct titration of $BaBr_2$ and $BaCl_2$ by 18-crown-ether-6 at 20°C.

Microcal™ITC200 system calibration was checked with Ba2+/18-crown ether-6 direct and reverse titrations. Data curves were refined with the global SEDPHAT analysis with "A + B <-> A-B Hetero-Association" model and titrant correction factors set to one.

	ΔH° (kcal.mol^{-1})	Kd (M)
$BaBr_2$	-7.36 ± 0.15	$1.41.10^{-4} \pm 0.08.10^{-4}$
$BaCl_2$	-7.31 ± 0.40	$1.51.10^{-4} \pm 0.30.10^{-4}$

The results were close to the reference data determined for $BaCl_2$ at 20°C: ΔH = - 7.66 ± 0.05 kcal.mol^{-1} and K_d = $1.36.10^{-4}$ ± 0.04 M^{-1} (IUPAC standard).[39]

The method makes it possible to precisely measure the volume of the cell (v_{cell} = 201.8 µl) in order to respect the strict 1:1 stoichiometry.[14] The corrected volume value was used in NITPIC and SEDPHAT calculations.

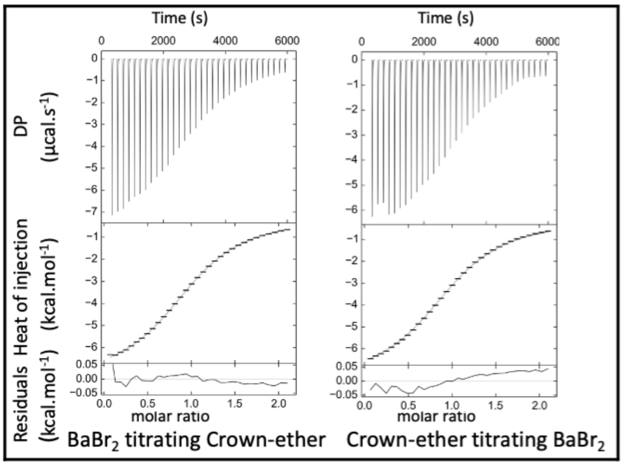

5 Supplemental tables

5.1 Table S1 Mean peptides residue molar ellipticities.

Mean residue molar ellipticities ([θ] deg.cm^2.dmol^{-1}) at λ = 215 and 207 nm and ratios of ellipticities at 215 / 207 nm for monocyclic peptides **1**-**4** and bicyclic peptides **1c**-**4c**, measured by CD in 10 mM phosphate buffer pH 7.4 at 25°C.

Peptide	$[\theta]_{215}$	$[\theta]_{207}$	$[\theta]_{215}$ / $[\theta]_{207}$
**1**	-4329	-7573	0.57
**1c**	-8133	-8297	0.98
**2**	-3770	-6220	0.61
**2c**	-8292	-7971	1.04
**3**	-3791	-6219	0.61
**3c**	-8884	-7903	1.12
**4**	-3790	-6634	0.57
**4c**	-4312	-6721	0.64

5.2 Table S2 Proton amide chemical shift variation with temperature.

Proton amide chemical shift variation with temperature Δδ/°C (ppb/°C). Values representative of strong hydrogen bonds, characterized by |Δδ/°C| < 4 ppb/°C, are colored in orange. Values for very strong hydrogen bonds, characterized by |Δδ/°C| < 1 ppb/°C or Δδ/°C > 0, are colored in red.

	1	1c	2	2c	3	3c	4	4c
C1	-	-	-	-	-	-	-	-
D2	-4.1	-3.1	-5.1	-3.5	-4.9	-3.5	-4.6	-5.0
I3	-8.9	-12.1	-9.1	-9.5	-7.7	-10.6	-9.5	-9.2
H4	-4.5	-3.7	-3.9	-4.1	-4.3	-2.7	-3.1	-3.9
V5	-3.0	-0.1	-2.8	-1.4	-2.3	0.0	-2.4	-3.2
^{n}L6	-6.9	-8.9	-8.5	-8.9	-7.2	-8.5	-8.1	-8.1
W7	-5.6	-6.8	-6.6	-5.1	-4.9	-5.0	-5.9	-6.4
E8	-1.9	1.8	-2.3	-1.7	-4.4	0.1	-1.5	-1.4
W9	-4.8	-7.2	-5.9	-4.3	-5.9	-5.9	-5.5	-5.4
K/E/D10	-3.4	-5.2	-2.7	-3.0	-2.7	-3.5	-4.1	-3.3
C11	-3.1	-0.9	-2.5	-1.5	-2.6	2.1	-2.4	-4.1
F12	-4.2	-5.8	-5.6	-3.7	-4.3	-3.5	-5.0	-4.6
E13	-4.2	-7.2	-5.5	-4.1	-4.7	-4.7	-5.2	-6.3
K/E/D14	-3.4	-5.5	-6.3	-5.5	-5.9	-0.9	-3.7	-3.3
L15	-4.5	-0.1	-7.0	-2.2	-6.4	0.3	-4.5	-5.1
K10/14 NHζ		-9.3		-8.3		-8.6		-7.5

5.3 Table S3 NMR chemical shift differences of diastereotopic protons of the lactam bridge

NMR chemical shift differences Δδ (ppm) of diastereotopic protons of the side chains of amino acids P10 and P14 involved in the lactam bridge. Chemical shift differences between methylene protons characterize inhomogeneity in their environment and evidence restraint in rotation of carbon-carbon bonds.

Peptide	1	1c	2	2c	3	3c	4	4c
Lys P10 HE1 / HE2	< 0.10	1.00					< 0.10	0.22
Asp P10 HB1 / HB2			< 0.10	0.64				
Glu P10 HB1 / HB2					< 0.10	0.80		
Asp P14 HB1 / HB2	0.16	0.23						
Lys P14 HE1 / HE2			< 0.10	0.36	< 0.10	0.73		
Glu P14 HB1 / HB2							< 0.10	0.17

5.4 Table S4 RMSD values of NMR peptides structures *vs.* crystal structure.

Backbone RMSD values (in Å, considering atoms C, CA, N and O) versus crystal structure of the 20 lowest energy structures calculated by NOE NMR restrained simulated annealing with XPLOR-NIH. Crystal structures of the bicyclic peptides **1c**-**4c** were also used as RMSD references for the corresponding monocyclic peptides **1**-**4**.

RMSD	aa P1-15	aa P2-5	aa P6-9	aa P10-14	number of NOE restraints
**1**	3.58	1.25	1.54	1.99	69
**1c**	3.64	1.62	1.52	1.84	82
**2**	4.00	1.46	1.95	2.43	80
**2c**	3.03	1.30	1.41	1.09	95
**3**	3.29	1.18	1.51	2.32	85
**3c**	2.53	1.18	1.63	0.60	103
**4**	3.86	1.63	1.64	2.75	73
**4c**	3.63	1.50	1.58	1.98	88

5.5 Table S5 Clustering of NMR structures.

Clustering with the program findClusters at the indicated value (in Å) of the 20 lowest energy structures calculated by NOE NMR restrained simulated annealing with XPLOR-NIH.
The number of clusters is indicated.

		cutoff (Å)					
Segment	Peptide	0.8	1.0	1.2	1.4	1.6	1.8
P2-5 Turn	**1**	7	5	3	1	1	1
	**1c**	16	14	6	1	1	1
	**2**	15	10	6	4	1	1
	**2c**	8	3	1	1	1	1
	**3**	8	5	3	1	1	1
	**3c**	8	5	1	1	1	1
	**4**	15	12	4	2	1	1
	**4c**	15	11	3	1	1	1
P6-9 Extended	**1**	14	11	7	5	2	1
	**1c**	14	11	6	2	1	1
	**2**	17	11	8	2	1	1
	**2c**	17	8	3	1	1	1
	**3**	15	11	4	1	1	1
	**3c**	17	13	4	2	1	1
	**4**	11	10	6	2	1	1
	**4c**	19	15	11	3	1	1
P10-14 Helix	**1**	18	11	9	4	2	1
	**1c**	11	10	6	3	1	1
	**2**	20	18	16	14	3	1
	**2c**	3	2	1	1	1	1
	**3**	20	18	14	11	5	2
	**3c**	3	2	1	1	1	1
	**4**	17	16	14	7	2	1
	**4c**	17	15	10	4	1	1

5.6 Table S6 Diffraction data and crystal structures refinement.

Data set	$VEGF_2$: peptide **1c**	$VEGF_2$: peptide **2c**	$VEGF_2$: peptide **3c**	$VEGF_2$: peptide **4c**	$VEGF_2$
PDB ID	6ZCD	6Z3F	6Z13	6ZBR	6ZFL
Resolution range (outer shell) (Å)	45.17-1.80 (1.84-1.80)	44.55–2.10 (2.16–2.10)	45.12–1.80 (1.89–1.80)	55.60–1.60 (1.69-1.60)	44.17–1.60 (1.69-1.60)
Unique reflections (outer shell)	22094 (1271)	14341 (1151)	22364 (4517)	31383 (4517)	41413 (6017)
Data redundancy (outer shell)	9.8 (10.0)	7.5 (7.7)	6.0 (5.8)	8.8 (8.6)	3.3 (3.1)
Completeness, % (outer shell)	100 (100)	99.9 (99.8)	99.8 (97.2)	100 (100)	99.7 (99.7)
R_{merge} (outer shell) (%)	9.8 (90.2)	13.2 (70.1)	6.8 (70.4)	7.7 (68.2)	9.2 (83.8)
$<I/\sigma(I)>$	11.5 (2.3)	8.10 (2.2)	14.6 (2.4)	11.3 (2.4)	7.9 (1.6)
R_{cryst}/R_{free} (%)	19.0/24.2	20.9/26.5	20.4/24.1	20.4/24.2	16.5/18.6
CC(1/2) (outer shell)	0.998 (0.957)	0.998 (0.910)	0.999 (0.881)	0.999 (0.966)	0.996 (0.707)
Crystal					
Space group	$P2_12_12_1$	$P2_12_12_1$	$P2_12_12_1$	$P2_12_12_1$	$P4_1$
Cell dimensions					
a, b, c (Å)	53.64, 55.70, 77.21	54.42, 55.82, 77.59	54.08, 55.60, 77.07	54.25, 55.69, 76.98	88.34, 88.34, 40.49
α, β, ϒ (deg.)	90, 90, 90	90, 90, 90	90, 90, 90	90, 90, 90	90, 90, 90

The real space correlation coefficient reflects the goodness of fit of partial models, from 0 to 1 (best fit), with ρ the partial model electronic density:

$$RSCC = \frac{\sum(\rho_{obs} - <\rho_{obs}>)(\rho_{calc} - <\rho_{calc}>)}{[\sum(\rho_{obs} - <\rho_{obs}>)^2 \sum(\rho_{calc} - <\rho_{calc}>)^2]^{1/2}}$$

5.8 Table S7a ITC data.

Thermodynamic parameter of cyclic and bicyclic peptides binding on VEGF, determined by ITC at 20°C and 37°C.

			Nitpic/SEDPHAT individual thermogram analysis			Nitpic/SEDPHAT global analysis of thermograms									
	[VEGF] (µM)	[Peptide] (µM)	corr. Factor (Cell)	Kd1 (nM)	ΔH_1°/ mole of binding site (kcal.mol^{-1})	corr. Factor [VEGF]	corr. Factor [peptide]	base line (cal.mol^{-1})	*c* value	Kd_1 (nM)	ΔG_1° (kcal.mol^{-1})	ΔH_1° (kcal.mol^{-1})	ΔS_1° (cal.mol^{-1}K^{-1})	$-T\Delta S_1^\circ$ (kcal.mol^{-1})	incompet. fraction [VEGF]
20°C	22.8	310.1	0.882	183	-16.91	0.864	0.952	-426	257						0.024
peptide **1**	20.3	356.0	0.821	153	-18.48	0.882	1.046	-132	228						0.024
titrating VEGF	20.2	280.5	0.806	122	-19.08	0.898	1.044	481	227						0.024
	13.1	302.6	0.838	184	-18.78	0.988	1.045	20	148						0.000
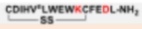			SEDPHAT average corr. factor:			0.908	1.022								
	62.4	12.8	0.971	195	-18.26	1.000	0.961	-1293	36						0.024
VEGF titrating	61.8	13.3	0.927	201	-18.34	1.000	0.901	-1204	37						0.024
peptide **1**	149.0	30.3	0.929	170	-17.84	1.000	0.931	1950	85						0.000
	161.9	29.5	0.922	152	-17.32	1.000	0.917	3398	83						0.000
	138.7	27.3	0.911	175	-18.22	1.000	0.913	338	77						0.000
	135.9	27.6	0.909	178	-18.10	1.000	0.890	441	78						0.000
			SEDPHAT average corr. factor:			1.000	0.919		results:	177.9	-9.05	-17.77	-29.73	8.72	
20°C	24.2	473.0	0.826	45	-17.62	0.894	1.056	-190	1 263						0.024
peptide **1c**	28.3	391.0	0.752	30	-17.20	0.820	1.057	-295	1 476						0.024
titrating VEGF	28.3	384.0	0.758	36	-17.62	0.805	1.041	-76	1 476						0.024
	4.3	89.4	0.915	37	-16.40	0.935	0.993	-273	226						0.024
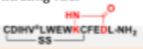			SEDPHAT average corr. factor:			0.864	1.037								
	41.5	8.7	0.857	36	-16.40	1.000	0.836	-990	114						0.024
VEGF titrating	88.8	17.4	0.936	54	-14.96	1.000	0.819	-413	227						0.121
peptide **1c**	68.7	13.5	0.920	44	-13.99	1.000	0.801	1832	176						0.121
	139.1	28.7	0.917	35	-16.75	1.000	0.918	8	374						0.000
	102.5	19.7	0.936	36	-16.57	1.000	0.936	620	257						0.000
			SEDPHAT average corr. factor:			1.000	0.862		results:	38.3	-9.95	-16.61	-22.72	6.66	
20°C	22.8	319.5	0.773	142	-17.28	0.864	1.140	-95	294						0.024
peptide **2**	22.8	319.5	0.737	123	-17.65	0.849	1.125	-57	294						0.024
titrating VEGF	31.9	487.3	0.740	170	-17.42	0.828	1.090	12	411						0.024
	15.6	301.5	0.775	134	-16.84	0.944	1.071	152	201						0.121
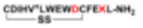	11.8	231.0	0.903	138	-16.62	0.954	1.056	678	152						0.000
	12.3	252.3	0.877	115	-16.52	0.936	1.064	1010	159						0.000
	10.4	207.6	0.873	125	-16.82	0.933	1.076	892	134						0.000
			SEDPHAT average corr. factor:			0.901	1.089								
	62.2	14.6	0.887	158	-15.57	1.000	0.865	-594	47						0.024
VEGF titrating	62.3	14.6	0.891	122	-14.97	1.000	0.867	-1132	47						0.024
peptide **2**	153.1	30.6	1.089	131	-13.47	1.000	0.957	651	98						0.121
	156.6	30.3	1.033	160	-13.68	1.000	0.908	616	98						0.121
	85.1	14.7	0.847	101	-16.05	1.000	0.848	1082	47						0.000
	235.4	46.9	0.978	154	-15.86	1.000	0.978	434	151						0.000
	132.9	26.0	0.876	166	-16.21	1.000	0.885	371	84						0.000
			SEDPHAT average corr. factor:			1.000	0.901		results:	155.2	-9.13	-15.82	-22.80	6.68	
20°C	24.1	390.0	0.812	47	-17.02	0.833	1.004	830	817						0.024
peptide **2c**	21.8	340.9	0.739	84	-19.05	0.822	1.086	496	737						0.024
titrating VEGF	21.3	349.0	0.828	65	-16.20	0.802	0.945	409	721						0.024
	15.3	238.0	0.821	86	-18.07	0.853	0.997	57	517						0.024
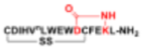	15.6	314.0	0.777	29	-17.75	0.998	1.146	990	528						0.121
	13.9	280.5	0.674	48	-18.74	0.844	1.102	1359	472						0.121
	11.2	236.7	0.922	43	-18.23	0.997	1.083	938	379						0.000
	8.0	161.2	1.005	36	-17.81	1.080	1.076	639	271						0.000
	8.5	162.6	0.900	48	-18.99	1.005	1.117	1156	286						0.000
			SEDPHAT average corr. factor:			0.915	1.062								
	43.0	12.0	0.840	34	-15.81	1.000	0.826	-2958	102						0.024
VEGF titrating	42.3	10.5	0.878	46	-16.85	1.000	0.846	-534	89						0.024
peptide **2c**	88.6	16.3	1.121	57	-15.05	1.000	0.984	63	138						0.121
	66.4	13.5	1.050	46	-15.32	1.000	0.916	2327	115						0.121
	135.4	27.5	1.059	61	-15.16	1.000	0.931	556	233						0.121
	152.2	27.5	1.066	61	-14.43	1.000	0.936	1920	232						0.121
	138.8	28.0	0.939	48	-17.50	1.000	0.941	225	237						0.000
	262.8	51.9	0.976	57	-17.05	1.000	0.976	1848	439						0.000
			SEDPHAT average corr. factor:			1.000	0.919		results:	59.1	-9.70	-17.11	-25.28	7.41	

VEGF purification batchs	n° 1	n° 2	n° 3

5.9 Table S7b ITC data.

Thermodynamic parameters of cyclic and bicyclic peptides binding on VEGF, determined by ITC at 20°C and 37°C.

	Nitpic/SEDPHAT individual thermogram analysis					Nitpic/SEDPHAT global analysis of thermograms									
	[VEGF] (µM)	[Peptide] (µM)	corr. Factor (Cell)	Kd1 (nM)	ΔH_1°/ mole of binding site (kcal.mol^{-1})	corr. Factor [VEGF]	corr. Factor [peptide]	base line (cal.mol^{-1})	*c* value	Kd_1 (nM)	ΔG_1° (kcal.mol^{-1})	ΔH_1° (kcal.mol^{-1})	ΔS_1° (cal.mol^{-1}K^{-1})	-TΔS_1° (kcal.mol^{-1})	incompet. fraction [VEGF]
**20°C**	24.8	470.3	0.902	81	-15.90	0.872	0.940	-268	566						0.024
peptide **3**	21.8	331.0	0.810	101	-16.59	0.803	0.963	-10	499						0.024
titrating VEGF	24.7	364.0	0.733	131	-20.71	0.851	1.163	-4923	564						0.024
CDIHV*LWEWECFEKL-NH$_2$ (SS)	24.2	389.0	0.959	91	-16.83	0.966	0.983	-529	553						0.024
	8.9	182.3	0.764	60	-16.72	0.873	1.004	234	204						0.121
			SEDPHAT average corr. factor:			0.873	1.011								
	62.8	15.4	0.880	68	-16.21	1.000	0.859	419	88						0.024
VEGF titrating	63.2	14.7	0.847	81	-16.67	1.000	0.827	-50	84						0.024
peptide **3**	85.1	17.0	1.049	64	-14.73	1.000	0.925	813	97						0.121
	99.9	17.0	0.847	81	-16.67	1.000	0.814	1833	97						0.121
			SEDPHAT average corr. factor:			1.000	0.856		results:	87.6	-9.47	-17.17	-26.28	7.70	
**20°C**	28.3	383.0	0.821	41	-15.93	0.840	0.999	-104	1 335						0.024
peptide **3c**	15.2	260.7	0.828	37	-15.78	0.841	0.993	-94	719						0.024
titrating VEGF	4.3	92.9	0.987	45	-14.93	0.943	0.934	-268	205						0.024
O—NH			SEDPHAT average corr. factor:			0.875	0.975								
CDIHV*LWEWECFEKL-NH$_2$ (SS)	43.0	9.2	0.886	41	-15.82	1.000	0.868	-479	108						0.024
VEGF titrating	88.3	16.3	1.160	57	-15.68	1.000	1.096	1091	192						0.121
peptide **3c**	103.7	17.3	0.850	30	-15.34	1.000	0.829	4174	204						0.000
	185.3	36.4	0.778	62	-15.90	1.000	0.780	255	429						0.000
	107.2	22.9	0.876	26	-16.00	1.000	0.711	1121	270						0.000
	111.3	22.5	0.915	47	-16.19	1.000	0.879	-59	265						0.000
			SEDPHAT average corr. factor:			1.000	0.861		results:	42.4	-9.89	-15.96	-20.71	6.07	
**20°C**	22.8	317.0	0.765	135	-18.85	0.840	1.073	-230	340						0.024
peptide **4**	22.8	317.0	0.800	137	-18.71	0.874	1.067	-208	340						0.024
titrating VEGF	31.9	497.0	0.807	142	-17.78	0.839	1.017	-77	475						0.024
	10.8	211.0	0.711	128	-17.59	0.819	1.012	103	161						0.121
CDIHV*LWEWKCFEEL-NH$_2$ (SS)			SEDPHAT average corr. factor:			0.843	1.042								
	63.5	15.1	0.955	129	-17.04	1.000	0.933	-929	56						0.024
VEGF titrating	62.5	14.3	0.839	116	-16.09	1.000	0.821	-508	53						0.024
peptide **4**	119.4	19.8	1.014	136	-15.21	1.000	0.892	84	74						0.121
	82.8	17.1	1.070	147	-15.12	1.000	0.942	483	64						0.121
	88.7	17.1	1.022	158	-14.54	1.000	0.828	1540	64						0.121
			SEDPHAT average corr. factor:			1.000	0.883		results:	134.2	-9.22	-17.38	-27.85	8.16	
**20°C**	21.3	352.0	0.857	143	-15.36	0.846	0.964	43	286						0.024
peptide **4c**	32.4	526.0	0.829	168	-15.74	0.832	0.979	-317	434						0.024
titrating VEGF	31.9	496.0	0.857	152	-16.03	0.879	1.003	43	428						0.024
HN—O			SEDPHAT average corr. factor:			0.852	0.982								
CDIHV*LWEWKCFEEL-NH$_2$ (SS)	63.7	15.5	0.825	118	-15.64	1.000	0.809	-251	52						0.024
VEGF titrating	130.8	23.1	0.825	102	-15.72	1.000	0.830	-221	77						0.024
VEGF titrating	130.8	23.1	0.805	256	-13.53	1.000	0.694	-175	77						0.121
peptide **4c**	109.5	20.8	0.821	234	-13.38	1.000	0.715	116	70						0.121
	97.1	19.8	0.745	184	-13.57	1.000	0.645	-122	66						0.121
			SEDPHAT average corr. factor:			1.000	0.762		results:	149.1	-9.16	-15.98	-23.27	6.82	
**37°C**	12.2	254.0	0.788	1 031	-22.72	0.892	0.995	-266	24						0.121
peptide **1**	11.8	253.5	0.792	973	-22.89	0.918	1.015	-213	23						0.121
titrating VEGF	11.7	220.5	0.899	972	-22.35	0.889	0.991	99	23						0.000
	7.3	153.3	0.976	1 067	-22.10	1.046	0.966	-781	14						0.000
CDIHV*LWEWKCFEDL-NH$_2$ (SS)			SEDPHAT average corr. factor:			0.936	0.992								
	202.8	42.0	0.894	1 124	-20.12	1.000	0.789	-305	21						0.121
VEGF titrating	223.0	42.9	0.860	929	-22.32	1.000	0.875	-82	21						0.000
peptide **1**	225.8	43.3	0.879	1 035	-22.66	1.000	0.859	-63	21						0.000
	229.7	45.2	0.951	985	-22.67	1.000	0.949	-30	22						0.000
			SEDPHAT average corr. factor:			1.000	0.868		results:	1 010	-8.51	-22.75	-47.44	14.24	
**37°C**	13.2	223.5	0.698	159	-25.36	0.889	1.121	-540	169						0.121
peptide **1c**	4.6	104.0	0.711	139	-24.93	0.793	1.116	-597	59						0.121
titrating VEGF	9.9	223.2	0.954	139	-22.31	0.973	1.010	-159	127						0.000
HN—O	10.2	238.8	0.811	138	-22.99	0.511	1.048	50	130						0.000
CDIHV*LWEWKCFEDL-NH$_2$ (SS)			SEDPHAT average corr. factor:			0.791	1.074								
	107.2	18.8	1.179	176	-19.85	1.000	1.039	-547	60						0.121
VEGF titrating	116.0	18.8	0.915	142	-18.68	1.000	0.805	-344	60						0.121
peptide **1c**	153.1	27.4	0.916	169	-22.67	1.000	0.917	-123	88						0.000
	121.0	23.5	0.906	128	-21.87	1.000	0.896	-194	75						0.000
			SEDPHAT average corr. factor:			1.000	0.914		results:	156	-9.66	-22.37	-42.36	12.71	
										VEGF purification batchs		n° 1	n° 2	n° 3	

5.10 Table S7c ITC data.

Thermodynamic parameters of cyclic and bicyclic peptides binding on VEGF, determined by ITC at 20°C and 37°C.

	Nitpic/SEDPHAT individual thermogram analysis					Nitpic/SEDPHAT global analysis of thermograms									
	[VEGF] (µM)	[Peptide] (µM)	corr. Factor (Cell)	Kd1 (nM)	ΔH_1°/ mole of binding site (kcal.mol^{-1})	corr. Factor [VEGF]	corr. Factor [peptide]	base line (cal.mol^{-1})	*c* value	Kd_1 (nM)	ΔG_1° (kcal.mol^{-1})	ΔH_1° (kcal.mol^{-1})	ΔS_1° (cal.mol^{-1}K^{-1})	-TΔS_1° (kcal.mol^{-1})	incompet. fraction [VEGF]
37°C	19.9	453.9	1.020	545	-22.26	1.064	1.039	245	59						0.000
peptide **2**	42.4	881.2	1.020	669	-22.59	0.951	1.025	146	127						0.000
titrating VEGF	19.2	391.9	0.938	740	-23.05	0.951	1.024	160	57						0.000
	12.2	245.7	0.911	588	-21.71	0.919	1.011	121	36						0.000
CDIHV*LWEWDCFEKL-NH_2 (SS)			**SEDPHAT average corr. factor:**			**0.971**	**1.025**								
	239.0	42.0	0.964	631	-22.10	1.000	0.967	32	31						0.000
VEGF titrating	213.8	41.1	1.007	587	-21.92	1.000	1.012	-350	31						0.000
peptide **2**	316.4	52.9	0.887	734	-22.04	1.000	0.886	-1149	40						0.000
	61.6	12.6	0.905	653	-21.53	1.000	0.903	-474	9						0.000
	126.2	26.1	0.936	661	-21.90	1.000	0.933	24	19						0.000
			**SEDPHAT average corr. factor:**			**1.000**	**0.940**		**results:**	**669**	**-8.76**	**-22.01**	**-44.15**	**13.25**	
37°C	13.4	264.0	0.864	236	-24.19	0.913	1.058	-17	104						0.000
peptide **2c**	9.7	176.2	0.842	288	-25.18	0.894	1.070	-194	75						0.000
titrating VEGF	14.2	263.7	0.802	239	-23.87	0.839	1.048	-160	110						0.000
O–NH			**SEDPHAT average corr. factor:**			**0.882**	**1.059**								
CDIHV*LWEWDCFEKL-NH_2 (SS)	99.6	18.3	0.931	233	-23.66	1.000	0.938	-360	35						0.000
	194.0	39.7	0.961	285	-23.16	1.000	0.960	-798	77						0.000
VEGF titrating	137.6	29.1	0.959	347	-22.99	1.000	0.954	1382	56						0.000
peptide **2c**	52.4	11.0	0.917	223	-22.66	1.000	0.911	-918	21						0.000
	90.9	18.3	0.931	232	-23.02	1.000	0.937	-956	35						0.000
	179.7	34.3	0.919	269	-22.97	1.000	0.919	-263	66						0.000
			**SEDPHAT average corr. factor:**			**1.000**	**0.937**		**results:**	**258**	**-9.35**	**-22.01**	**-42.20**	**12.66**	
37°C	10.4	192.4	0.906	382	-21.56	0.861	0.949	125	54						0.000
peptide **3**	9.7	188.2	0.946	314	-20.82	0.903	0.950	123	50						0.000
titrating VEGF	7.0	129.4	1.003	374	-20.54	0.920	0.915	429	36						0.000
CDIHV*LWEWECFEKL-NH_2 (SS)			**SEDPHAT average corr. factor:**			**0.895**	**0.938**								
	205.1	42.5	0.848	397	-23.02	1.000	0.849	0	55						0.000
VEGF titrating	165.9	32.2	0.847	372	-22.84	1.000	0.847	-143	42						0.000
peptide **3**	186.8	38.9	0.867	393	-23.02	1.000	0.867	-125	50						0.000
	137.5	25.1	0.879	415	-22.83	1.000	0.868	-80	33						0.000
			**SEDPHAT average corr. factor:**			**1.000**	**0.858**		**results:**	**386**	**-9.10**	**-22.96**	**-46.17**	**13.86**	
37°C	7.6	135.1	0.879	257	-19.81	0.757	0.855	-185	91						0.000
peptide **3c**	12.4	257.0	1.063	214	-19.42	0.910	0.860	-213	148						0.000
titrating VEGF	8.8	165.8	0.990	164	-20.53	0.916	0.927	40	105						0.000
	12.8	237.9	0.804	153	-23.40	0.845	1.052	-3	153						0.000
O–NH			**SEDPHAT average corr. factor:**			**0.857**	**0.924**								
CDIHV*LWEWECFEKL-NH_2 (SS)	81.1	15.9	0.769	168	-22.84	1.000	0.775	-1066	47						0.000
VEGF titrating	190.0	37.8	0.796	185	-21.80	1.000	0.794	472	112						0.000
peptide **3c**	167.0	33.4	0.805	182	-22.33	1.000	0.805	-519	99						0.000
	66.0	13.0	0.806	143	-21.82	1.000	0.808	-162	39						0.000
	151.4	29.8	0.917	154	-22.37	1.000	0.918	-79	89						0.000
			**SEDPHAT average corr. factor:**			**1.000**	**0.820**		**results:**	**168**	**-9.61**	**-22.39**	**-42.56**	**12.77**	
peptide **4** 37°C	9.7	208.2	0.913	690	-21.89	0.888	0.968	-159	27						0.000
titrating VEGF	15.5	282.2	0.965	830	-22.17	0.912	0.950	-190	43						0.000
CDIHV*LWEWKCFEEL-NH_2 (SS)			**SEDPHAT average corr. factor:**			**0.900**	**0.959**								
	148.1	29.0	0.892	715	-23.10	1.000	0.892	-77	20						0.000
VEGF titrating	96.7	20.7	0.851	764	-22.86	1.000	0.848	35	14						0.000
peptide **4**	129.6	25.9	0.832	912	-23.12	1.000	0.821	-305	18						0.000
			**SEDPHAT average corr. factor:**			**1.000**	**0.854**		**results:**	**725**	**-8.71**	**-22.99**	**-47.57**	**14.28**	
peptide **4C** 37°C	6.1	120.6	1.111	899	-16.33	0.870	0.767	462	16						0.000
titrating VEGF			**SEDPHAT average corr. factor:**			**0.870**	**0.767**								
HN–O<br>CDIHV*LWEWKCFEEL-NH_2 (SS)	149.4	28.3	0.637	673	-21.20	1.000	0.635	26	18						0.000
	136.9	23.6	0.720	956	-22.28	1.000	0.713	-471	15						0.000
VEGF titrating	136.0	27.4	0.686	925	-23.59	1.000	0.684	-907	18						0.000
peptide **4c**	101.6	20.3	0.701	727	-21.36	1.000	0.689	-549	13						0.000
			**SEDPHAT average corr. factor:**			**1.000**	**0.680**		**results:**	**769**	**-8.68**	**-22.19**	**-45.03**	**13.52**	

5.11 Table S8 ITC data.

Thermodynamic parameters of reverse and direct titration of $BaBr_2$ and $BaCl_2$ by 18-crown-ether-6.

	Nitpic/Sedphat individual analysis					Nitpic/SEDPHAT global thermograms analysis								
	[$BaBl_2$] (mM)	[CrownEther] (mM)	Corr fact cell	Kd (µM)	ΔH	corr. Factor [Br2]	corr. Factor [CrownEt]	base line (cal.mol^{-1})	*c* value	Kd (µM)	ΔG° (kcal.mol^{-1})	ΔH° (kcal.mol^{-1})	ΔS° (cal.mol^{-1}K^{-1})	-TΔS° (kcal.mol^{-1})
**$BaBr_2$ 20°C**	10.0	1.00	0.988	139.9	-7.38	1.000	0.992	-58	9					
titrating	10.0	1.00	0.990	145.1	-7.37	1.000	0.987	-39	9					
Crown Ether			**SEDPHAT average corr. factor:**			**1.000**	**0.989**							
**CrownEther**	1.00	10.0	0.995	144.5	-7.44	0.990	1.000	-130	7					
titrating $BaBr_2$	1.00	10.0	0.987	145.4	-7.46	0.994	1.000	-92	7					
			**SEDPHAT average corr. factor:**			**0.992**	**1.000**		**results:**	**141.0**	**-5.16**	**-7.36**	**-7.5**	**2.19**
**$BaCl_2$ 20°C**	12.5	1.25	1.000	166.1	-7.52	1.000	0.960	-73	8					
titrating	12.5	1.25	0.970	163.2	-7.62	1.000	0.922	-19	8					
Crown ether			**SEDPHAT average corr. factor:**			**1.000**	**0.941**							
**Crown Ether**	1.25	12.5	0.998	165.5	-7.52	0.973	1.000	-6	8					
titrating	1.25	12.5	0.948	150.8	-7.51	1.024	1.000	5	8					
$BaCl_2$	1.25	12.5	0.953	163.9	-7.76	1.009	1.000	0	8					
			**SEDPHAT average corr. factor:**			**1.002**	**1.000**		**results:**	**151.4**	**-5.21**	**-7.50**	**-7.8**	**2.29**

6 Analytical HPLC and mass spectrometry

6.1 Peptide **1**

CDIHV^{n}LWEWKCFEDL-NH$_2$
SS

MS (MALDI): $[M+H]^+$ calculated 1932.88; found 1933.03
HPLC: Rt = 19.56 min (20 - 100 % of solvent B in 30 min)

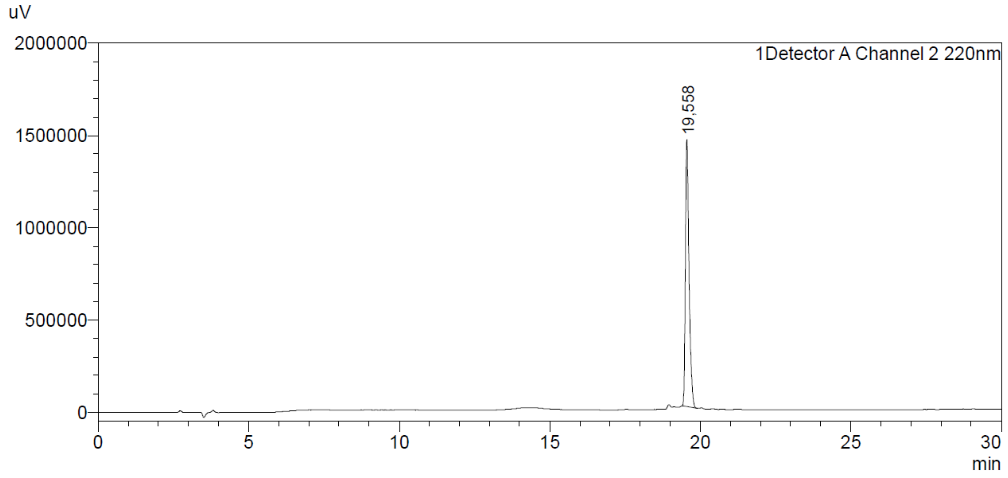

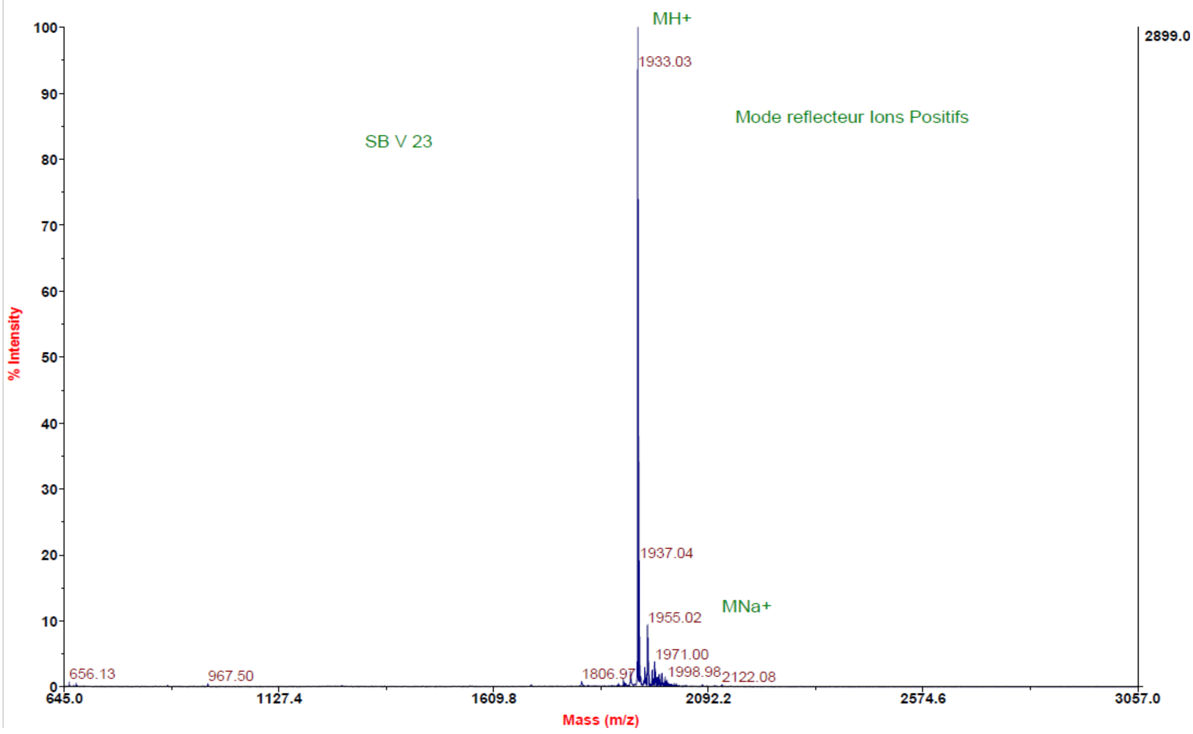

6.2 Peptide **1c**

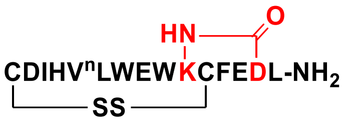

MS (MALDI): $[M+H]^+$ calculated 1914.89; found 1914.92
HPLC: Rt = 20.68 min (20 - 100 % of solvent B in 30 min)

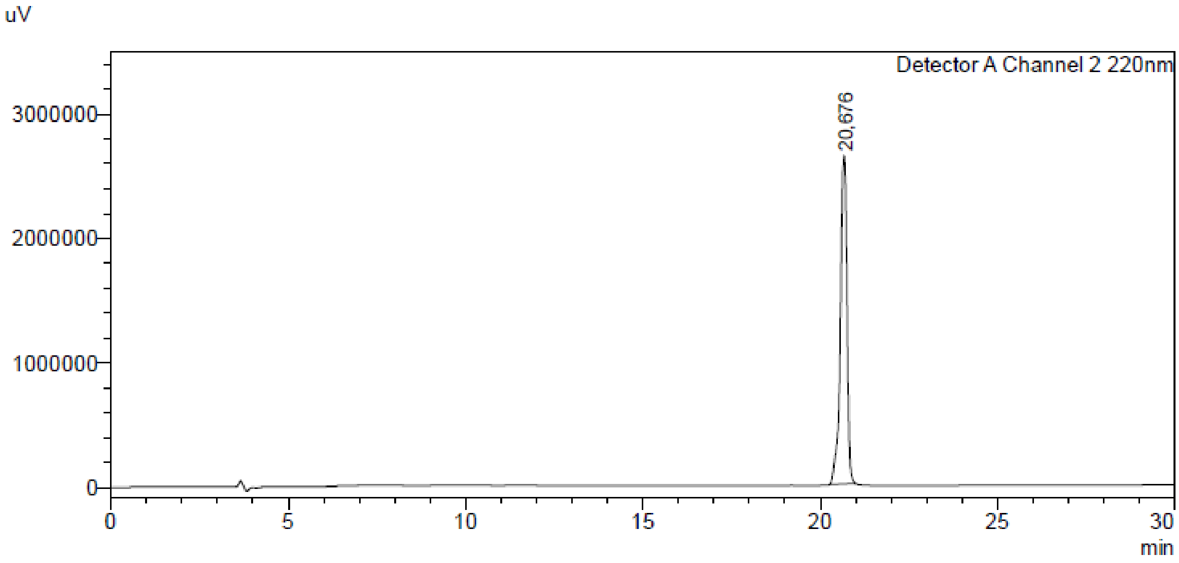

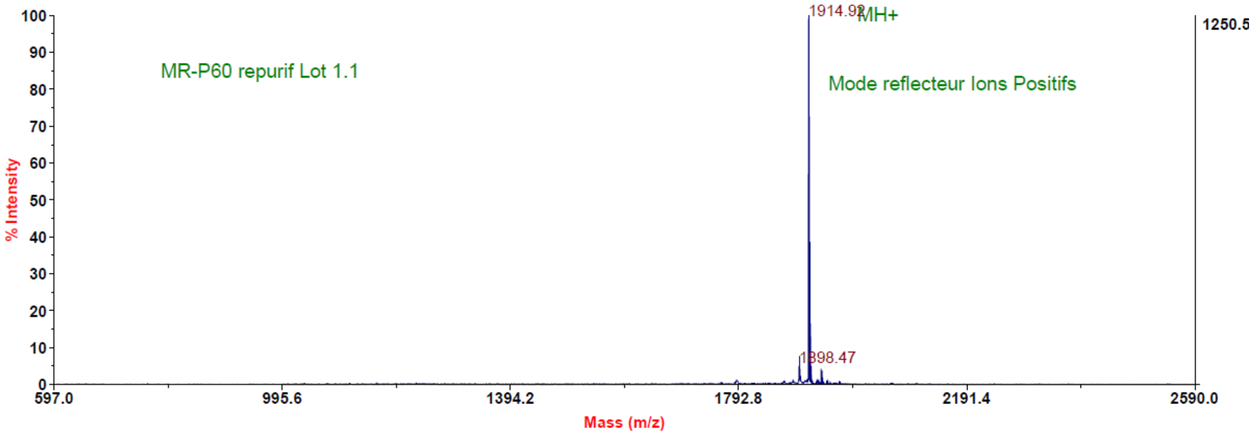

6.3 Peptide **2**

CDIHV^{n}LWEWDCFEKL-NH$_2$

SS

MS (MALDI): $[M+H]^+$ calculated 1932.88; found 1932.92

HPLC: Rt = 20.77 min (20 - 100 % of solvent B in 30 min), Uptisphere C18 5 µm Column, Interchim.

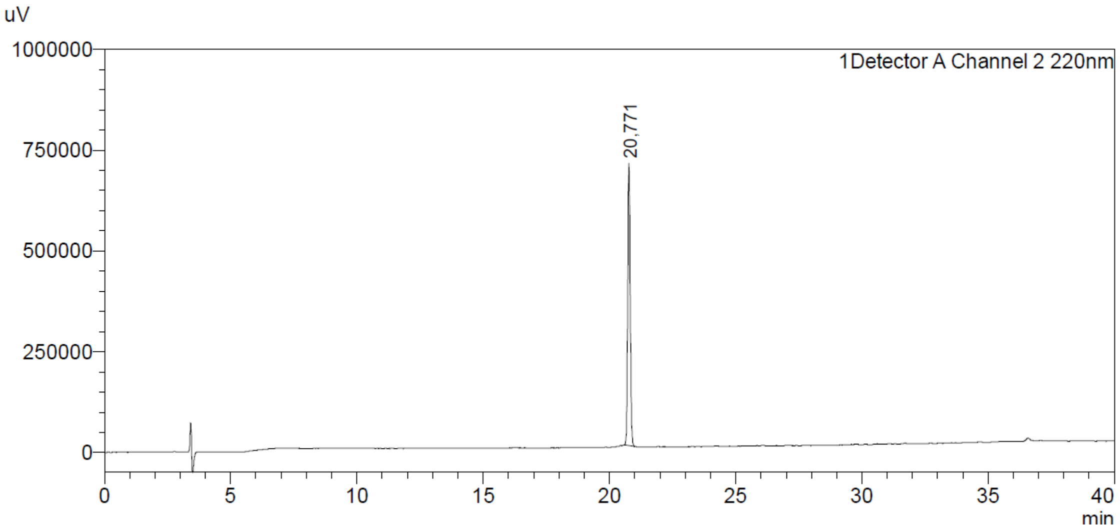

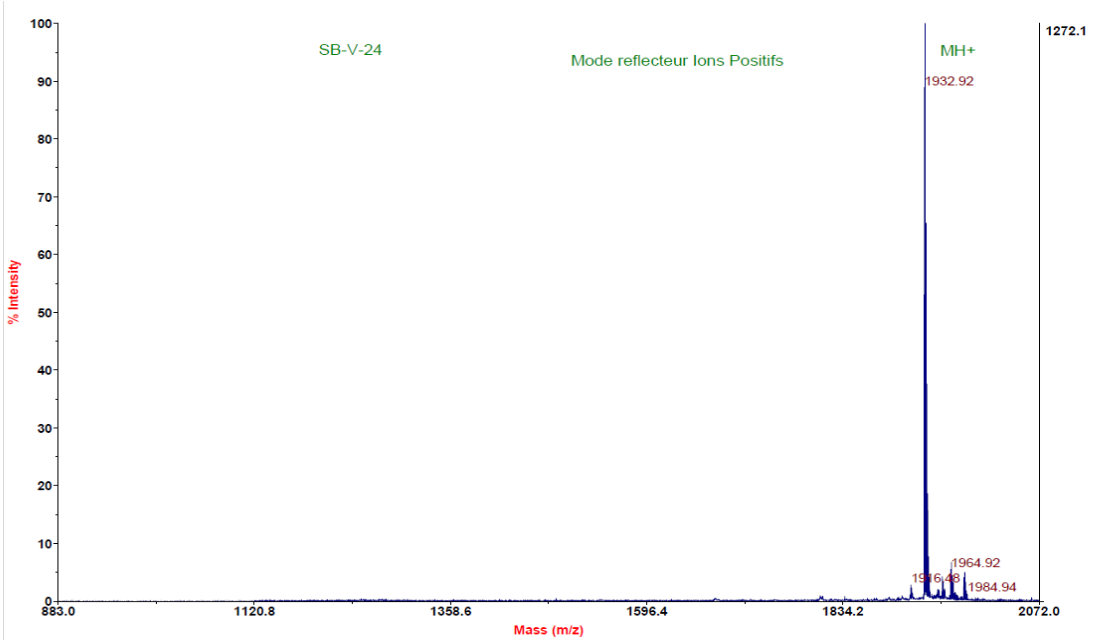

6.4 Peptide **2c**

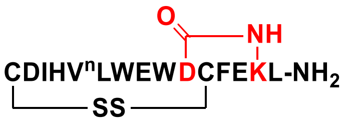

MS (MALDI): $[M+H]^+$ calculated 1914.89; found 1914.90
HPLC: Rt = 20.31 min (20 - 100 % of solvent B in 30 min)

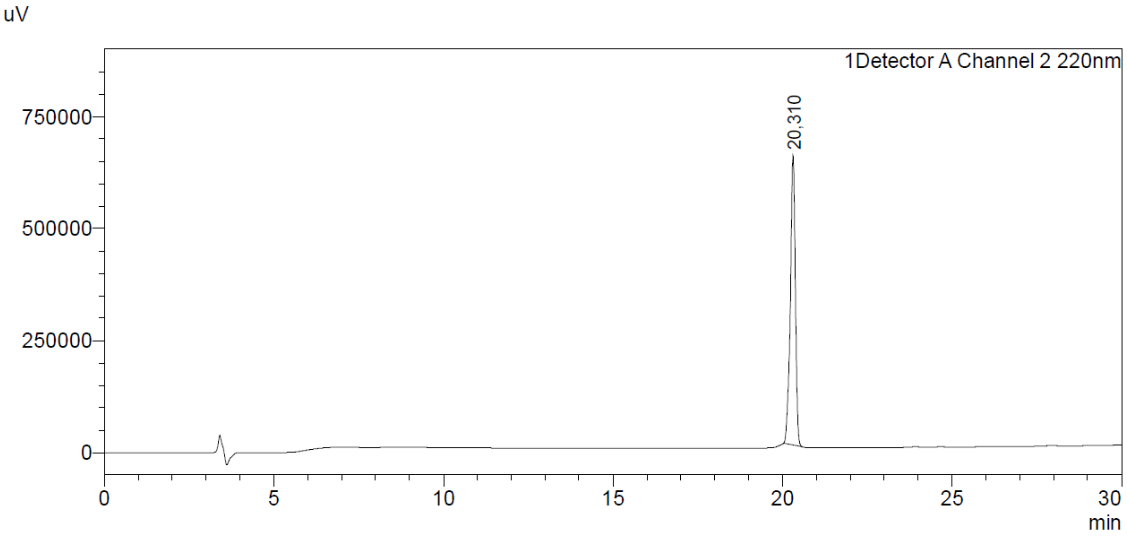

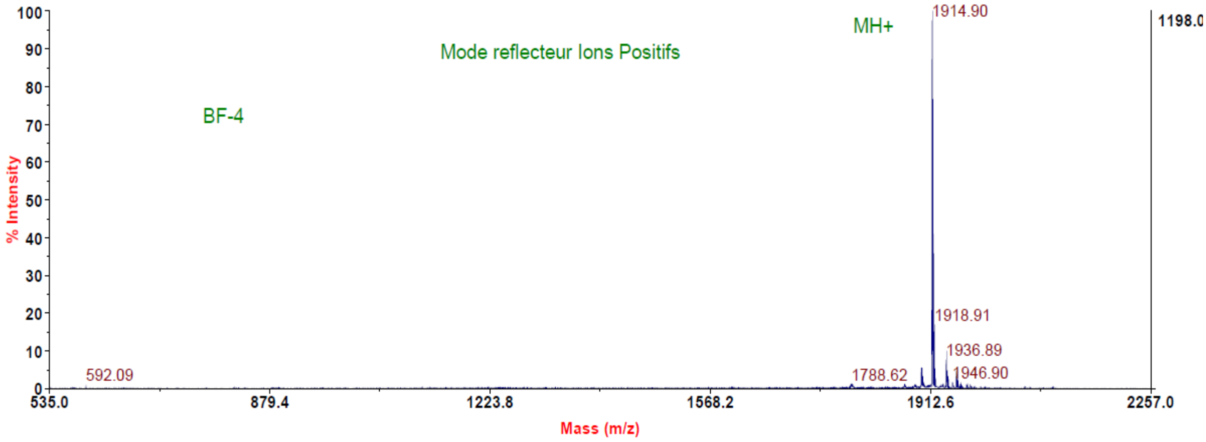

6.5 Peptide **3**

CDIHV^{n}LWEWECFEKL-NH$_2$
SS

MS (MALDI): $[M+H]^+$ calculate[4]d 1946.89; found 1946.89
HPLC: Rt = 19.67 min (20 - 100 % of solvent B in 30 min)

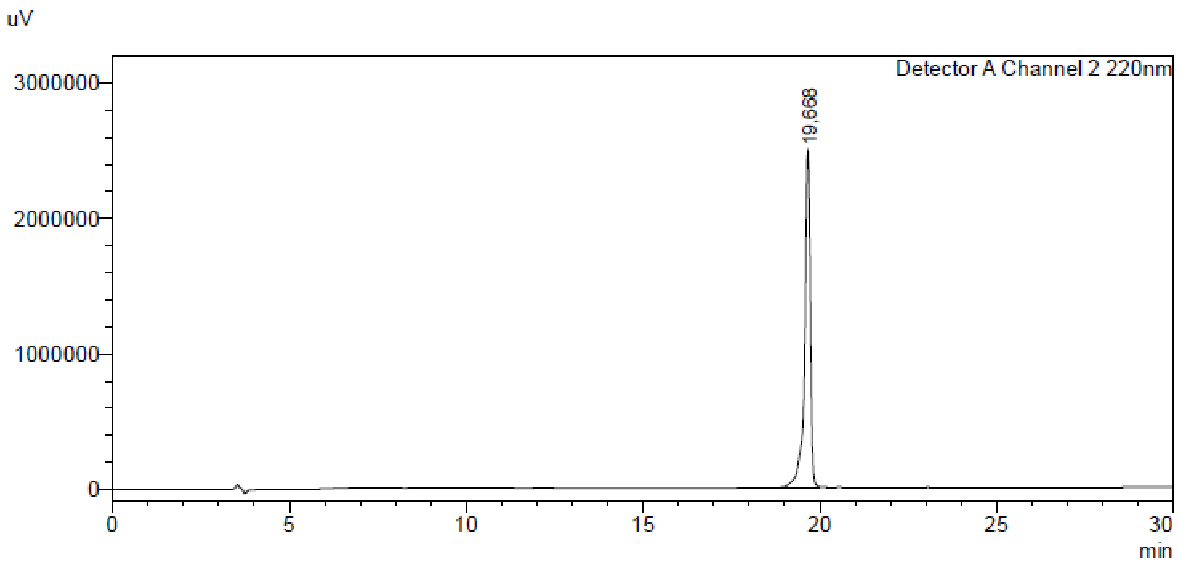

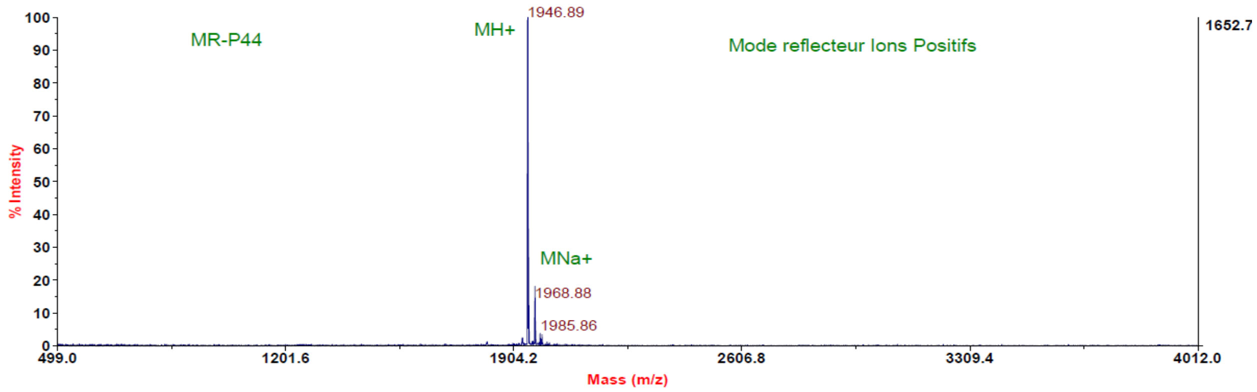

6.6 Peptide **3c**

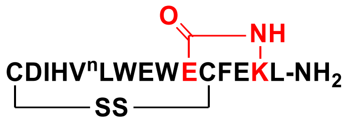

MS (MALDI): [M+H]+ calculated 1928.89; found 1928.98
HPLC: Rt = 21.78 min (20 - 100 % of solvent B in 30 min)

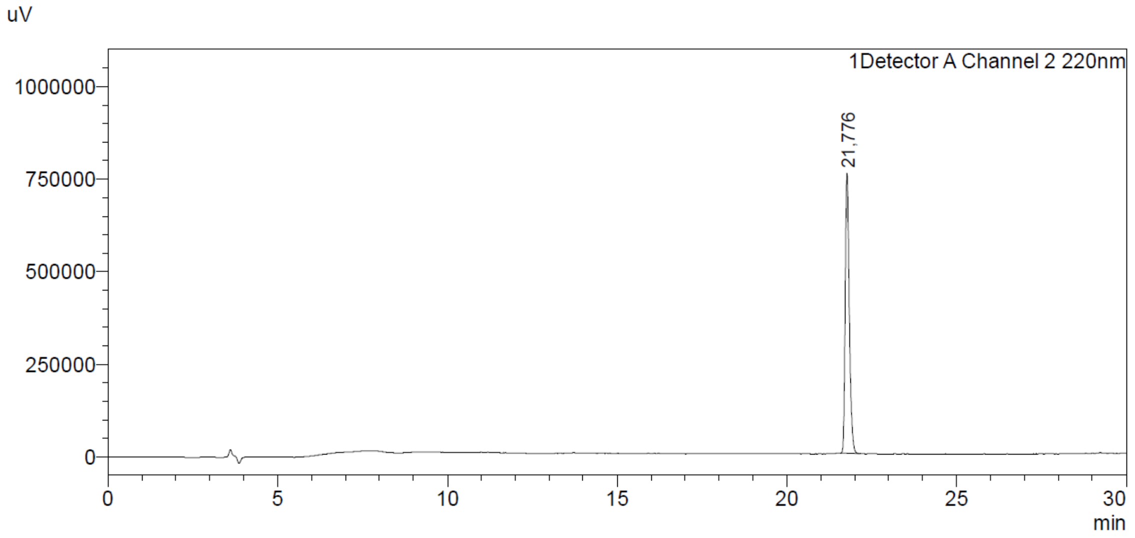

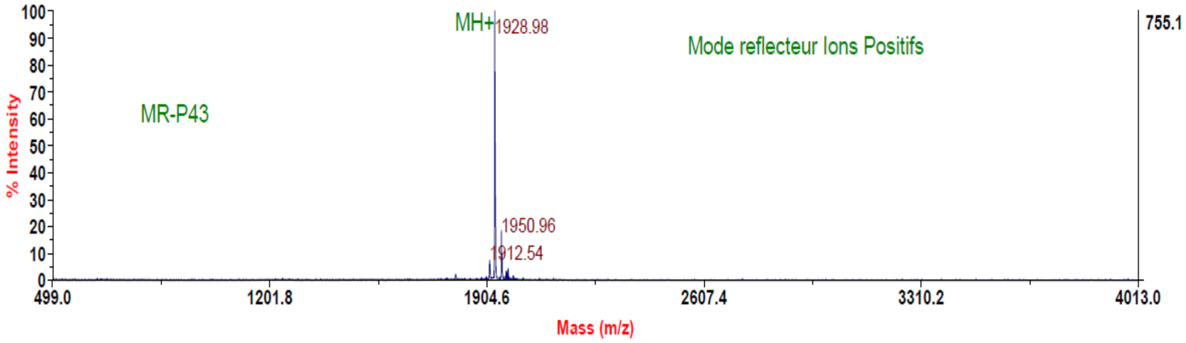

6.7 Peptide **4**

CDIHV^{n}LWEWKCFEEL-NH$_2$

SS (disulfide bridge between the two C residues)

MS (MALDI): [M+H]+ calculated 1946.89; found 1946.41
HPLC: Rt = 18.38 min (20 - 100 % of solvent B in 30 min)

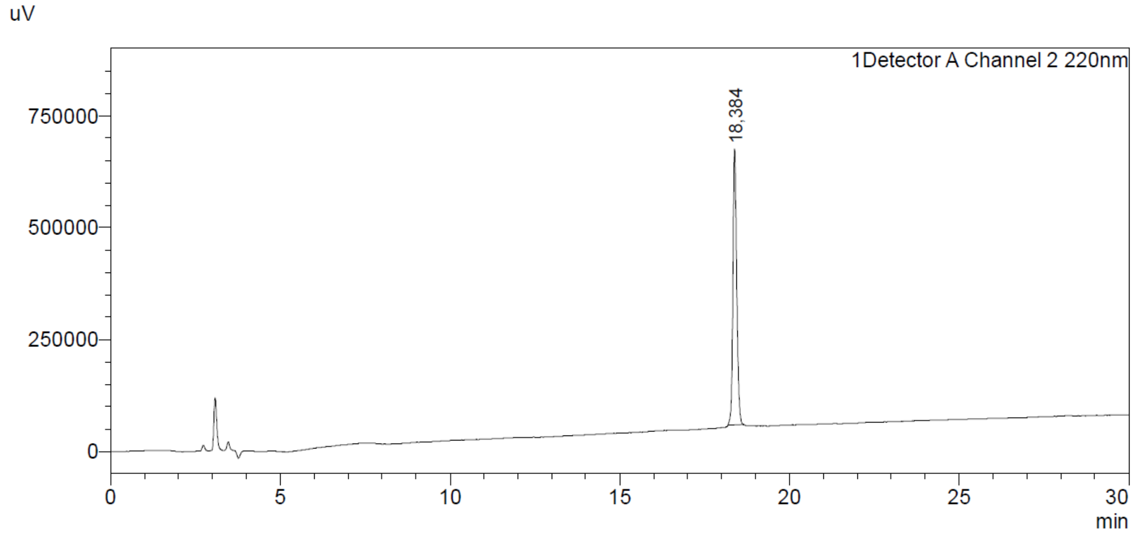

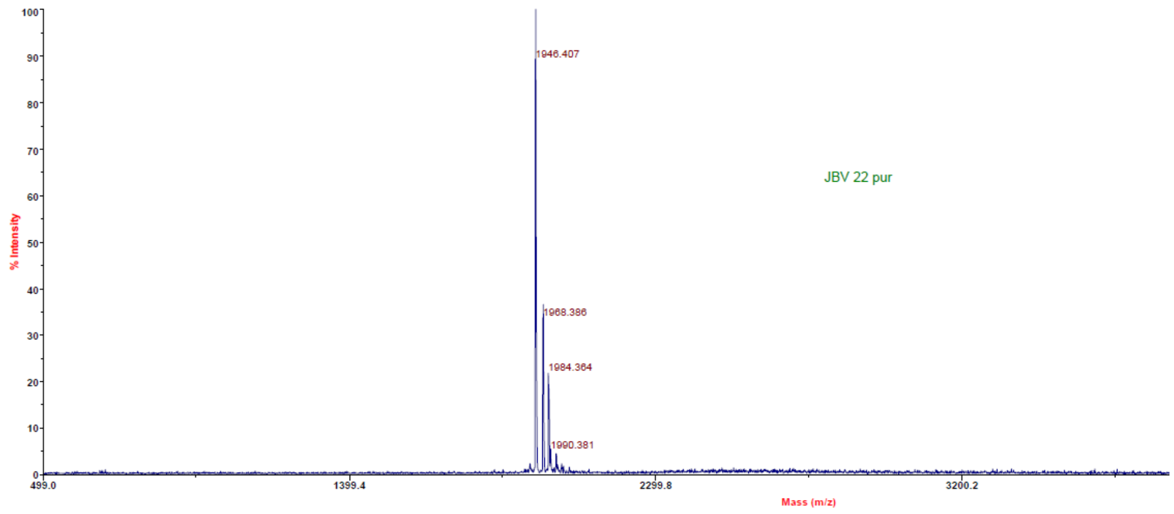

6.8 Peptide **4c**

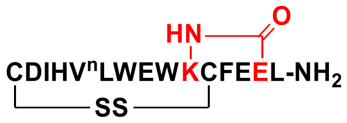

MS (MALDI): [M+H]+ calculated 1928.89; found 1928.97
HPLC: Rt = 20.98 min (20 - 100 % of solvent B in 30 min)

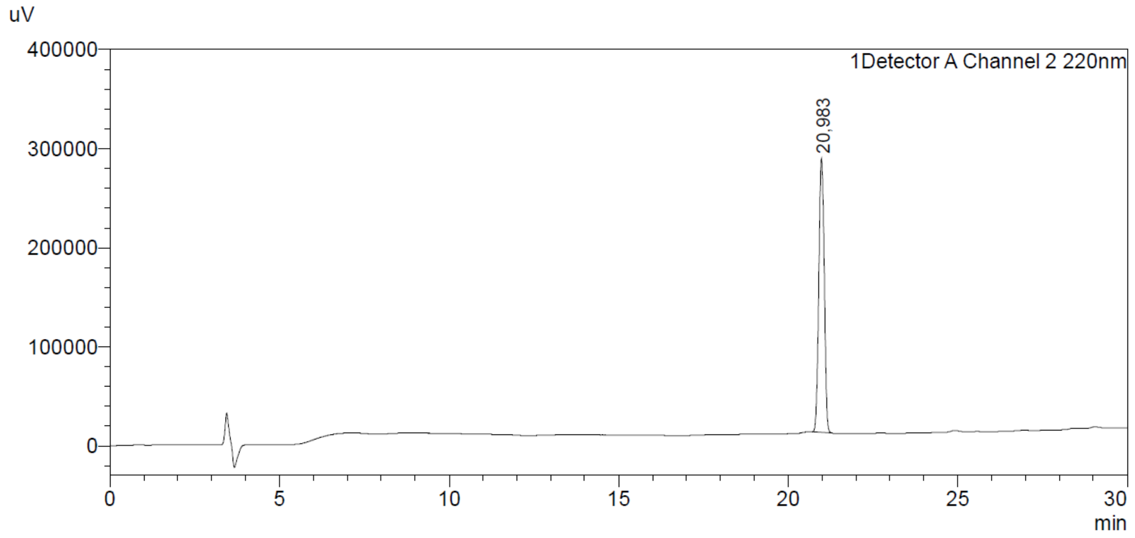

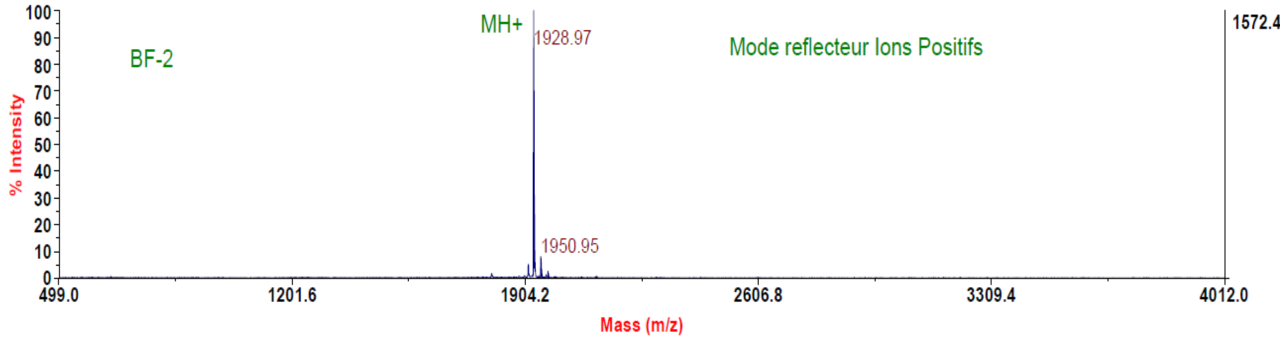

7 NMR data

Chemical shifts δ are given in ppm. Ha: Hα; Hb: Hβ; Hc: Hγ; Hd: Hδ; He: Hε; Hz: Hζ.
For methylene protons, only one chemical shift is given (for example Hba) when the two protons are found at the same chemical shift. If two chemical shifts are given (for example Hba and Hbb), the Hba is systematically attributed as the more upfield proton.

7.1 Peptide **1**

298K

		H	HA	Others			
1	Cys	-	4.214				
2	Asp	8.660	4.798	2.824:Hba			
3	Ile	8.133	4.072	1.810:Hb	1.078:Hg1a	0.751:Hd1*	
4	His	8.458	4.623	3.151:Hba	3.329:Hbb		
5	Val	7.605	4.167	1.935:Hb	0.754:Hga*		
6	Nle	8.035	4.037				
7	Trp	7.511	4.608	3.219:Hba	7.224:Hd1	10.135:He1	7.548:He3
8	Glu	7.681	4.155	1.819:Hba	2.046:Hga		
9	Trp	7.818	4.449	3.321:Hba	7.279:Hd1	10.128:He1	7.554:He3
10	Lys	7.715	4.058	1.654:Hba	1.050:Hga	1.526:Hda	2.870:Hea
11	Cys	7.957	4.472	2.999:Hba			
12	Phe	7.991	4.552	3.004:Hba	3.168:Hbb		
13	Glu	8.054	4.263	1.931:Hba	2.035:Hbb	2.378:Hga	
14	Asp	8.225	4.676	2.803:Hba	2.959:Hbb		
15	Leu	8.024	4.294				

7.2 Peptide **1c**

298K

		H	HA	Others				
1	Cys	-	4.140					
2	Asp	8.566	4.852	2.742:Hba	2.857:Hbb			
3	Ile	8.264	4.116	1.835:Hb	0.738:Hd1*			
4	His	8.500	4.608	3.201:Hba	3.405:Hbb			
5	Val	7.454	4.223	1.871:Hb	0.716:Hga*			
6	Nle	8.153	4.006	1.641:Hba	1.184:Hg			
7	Trp	7.435	4.690	3.221:Hba	3.329:Hbb	7.224:Hd1	10.128:He1	7.531:He3
8	Glu	7.597	4.237	2.004:Hba	2.203:Hga			
9	Trp	7.938	4.405	3.334:Hba	7.329:Hd1	10.061:He1	7.503:He3	
10	Lys	7.614	4.007	1.158:Hga	1.411:Hda	2.617:Hea	3.615:Heb	7.826:Hz*
11	Cys	7.716	4.179	3.012:Hba				
12	Phe	7.690	4.260	2.921:Hba	3.108:Hbb			
13	Glu	8.247	3.990	1.927:Hba	2.383:Hga			
14	Asp	8.133	4.732	2.665:Hba	2.896:Hbb			
15	Leu	7.616	4.237					

7.3 Peptide **2**

298K

		H	HA	Others				
1	Cys	-	4.202					
2	Asp	8.709	4.811	2.807:Hba	2.890:Hbb			
3	Ile	8.143	4.092	1.824:Hb	0.762:Hd1*			
4	His	8.432	4.629	3.170:Hba	3.354:Hbb			
5	Val	7.633	4.159	1.954:Hb	0.766:Hga*			
6	Nle	8.072	4.012	1.631:Hba	1.148:Hga			
7	Trp	7.510	4.601	3.120:Hba	3.243:Hbb	7.214:Hd1	10.086:He1	7.541:He3
8	Glu	7.623	4.245	1.811:Hba	2.120:Hga			
9	Trp	7.869	4.475	3.302:Hba	7.276:Hd1	10.109:He1	7.591:He3	
10	Asp	7.970	4.448	2.552:Hba				
11	Cys	7.860	4.407	2.918:Hba				
12	Phe	7.966	4.559	2.997:Hba	3.164:Hbb			
13	Glu	7.984	4.260	1.914:Hba	2.020:Hbb	2.352:Hga		
14	Lys	8.122	4.283	1.424:Hga	1.691:Hda	2.986:Hea		
15	Leu	8.086	4.321	0.900:Hda*				

7.4 Peptide **2c**

298K

		H	HA	Others					
1	Cys	-	4.190	2.759:Hba	2.904:Hbb				
2	Asp	8.465	4.796	2.739:Hba	2.867:Hbb				
3	Ile	8.302	4.048	1.842:Hb	1.114:Hg1a	0.746:Hd1*			
4	His	8.523	4.596	3.227:Hba	3.410:Hbb				
5	Val	7.569	4.156	1.951:Hb	0.675:Hga*	0.791:Hgb*			
6	Nle	8.009	3.920	1.660:Hba	1.140:Hga				
7	Trp	7.415	4.681	3.314:Hba	3.373:Hbb	7.238:Hd1	10.133:He1	7.549:He3	
8	Glu	7.518	4.349	1.816:Hba	2.012:Hga				
9	Trp	8.021	4.454	3.410:Hba	7.380:Hd1	10.137:He1	7.604:He3		
10	Asp	8.077	4.502	1.974:Hba	2.614:Hbb				
11	Cys	7.979	4.081	2.906:Hba	3.111:Hbb				
12	Phe	7.947	4.253	2.984:Hba	3.085:Hbb				
13	Glu	7.802	4.123	2.014:Hba	2.343:Hga				
14	Lys	7.797	4.202	1.877:Hba	1.213:Hga	1.520:Hda	2.919:Hea	3.281:Heb	7.931:Hz*
15	Leu	7.681	4.211	1.733:Hba	1.551:Hg	0.856:Hda*			

7.5 Peptide **3**

298K

		H	HA	Others				
1	Cys	-	4.219	3.127:Hba				
2	Asp	8.698	4.778	2.852:Hba				
3	Ile	7.983	4.143	1.818:Hb	1.078:Hg1a	0.766:Hd1*		
4	His	8.435	4.621	3.145:Hba	3.315:Hbb			
5	Val	7.583	4.177	1.944:Hb	0.734:Hga*			
6	Nle	8.012	3.941	1.500:Hba				
7	Trp	7.310	4.668	2.947:Hba	3.009:Hbb	7.187:Hd1	10.161:He1	7.556:He3
8	Glu	8.005	4.151	2.012:Hba	2.243:Hga			
9	Trp	7.766	4.431	3.294:Hba	3.402:Hbb	7.352:Hd1	10.041:He1	7.508:He3
10	Glu	7.765	4.016	1.780:Hba	2.001:Hga			
11	Cys	7.892	4.413	2.950:Hba				
12	Phe	7.882	4.601	3.025:Hba	3.227:Hbb	7.271:Hd*		
13	Glu	7.983	4.303	2.048:Hba	2.439:Hga			
14	Lys	8.137	4.297	1.858:Hba	1.450:Hga	1.699:Hda	2.999:Hea	7.524:Hz*
15	Leu	8.074	4.319	1.728:Hba	1.605:Hg	0.910:Hda*		

7.6 Peptide **3c**

```
298K

              H     HA   Others
1    Cys      -  4.162
2    Asp  8.663  4.853  2.796:Hba   2.919:Hbb
3    Ile  8.308  4.070  1.813:Hb    0.977:Hg1a  1.091:Hg1b  0.702:Hg2*
4    His  8.300  4.612  3.215:Hba   3.406:Hbb
5    Val  7.490  4.195  1.892:Hb    0.744:Hga*
6    Nle  8.093  4.012  1.900:Hba   1.659:Hga   1.178:Hda   0.836:He*
7    Trp  7.568  4.626  3.195:Hba   3.318:Hbb   7.194:Hd1   9.921:He1   7.461:He3
8    Glu  7.389  4.350  1.962:Hba   2.213:Hga
9    Trp  8.089  4.362  3.325:Hba   7.346:Hd1  10.078:He1
10   Glu  7.858  3.717  1.363:Hba   2.162:Hbb   2.219:Hga   2.374:Hgb
11   Cys  7.462  4.003  2.943:Hba
12   Phe  7.698  4.152  2.937:Hba   3.063:Hbb
13   Glu  8.051  3.960  2.120:Hba   2.408:Hga
14   Lys  7.446  4.067  1.685:Hba   1.923:Hbb   1.243:Hga   1.403:Hda   2.698:Hea   3.428:Heb
7.723:Hz*
15   Leu  7.516  4.159  1.779:Hba   1.556:Hg    0.847:Hda*
```

7.7 Peptide **4**

```
298K

              H     HA   Others
1    Cys      -  4.214
2    Asp  8.650  4.810  2.849:Hba
3    Ile  8.155  4.079  1.815:Hb    1.076:Hg1a  0.754:Hd1*
4    His  8.403  4.630  3.157:Hba   3.343:Hbb
5    Val  7.584  4.176  1.920:Hb    0.749:Hga*
6    Nle  8.061  4.032  1.626:Hba   1.177:Hga   0.827:He*
7    Trp  7.510  4.617  3.189:Hba   3.264:Hbb   7.237:Hd1  10.142:He1   7.531:He3
8    Glu  7.674  4.168  1.837:Hba   2.065:Hga
9    Trp  7.872  4.448  3.333:Hba   7.311:Hd1  10.138:He1   7.554:He3
10   Lys  7.737  4.035  1.664:Hba   1.053:Hga   1.525:Hda   2.873:Hea   7.475:Hz*
11   Cys  7.910  4.450  3.007:Hba
12   Phe  7.998  4.496  3.023:Hba   3.156:Hbb
13   Glu  8.048  4.219
14   Glu  8.051  4.294
15   Leu  7.994  4.293  1.695:Hba   1.587:Hg    0.901:Hda*
```

7.8 Peptide **4c**

```
298K

              H     HA
1    Cys      -  4.218
2    Asp  8.714  4.800  2.810:Hba
3    Ile  8.079  4.126  1.811:Hb    0.766:Hd1*
4    His  8.506  4.639  3.122:Hba   3.299:Hbb
5    Val  7.641  4.161  1.929:Hb    0.740:Hga*
6    Nle  8.068  3.992  1.576:Hba
7    Trp  7.439  4.654  3.127:Hba   7.194:Hd1  10.089:He1   7.501:He3
8    Glu  7.753  4.154  1.887:Hba   2.142:Hga
9    Trp  7.807  4.470  3.315:Hba   7.305:Hd1  10.088:He1   7.523:He3
10   Lys  7.573  4.080  2.966:Hea   3.183:Heb   7.632:Hz*
11   Cys  7.984  4.353  2.968:Hba
12   Phe  7.770  4.478  3.001:Hba   3.174:Hbb
13   Glu  8.216  4.146  2.001:Hba   2.111:Hbb   2.404:Hga
14   Glu  7.947  4.303  1.989:Hba   2.154:Hbb   2.301:Hga   2.407:Hgb
15   Leu  7.997  4.292  1.709:Hba   1.583:Hg    0.900:Hda*
```

7.9 NOE restraints used in XPLOR-NIH simulated annealing

Distance restraints are given in Å in .tbl file format followed by lower and upper accepted limits (Δd/d = 20% by default). Comments regarding signal overlap and pseudo-atom corrections are given.

7.9.1 Peptide **1**

```
(2  HN  )  (1  HA  )    2.899    0.580    0.580
(2  HN  )  (2  HA  )    2.609    0.522    0.522
(2  HN  )  (2  HB# )    3.252    1.250    1.250 ! HB2 to HB# +/- 0.6 Å
(2  HA  )  (3  HN  )    2.773    0.555    0.555
(3  HN  )  (3  HA  )    3.291    0.658    0.658
(3  HN  )  (3  HD1#)    3.291    0.658    1.658 ! + 1.0 Å
(3  HN  )  (3  HG1#)    3.271    1.654    1.654 ! HG12 to HG1# +/- 1.0 Å
(3  HN  )  (3  HB  )    3.255    0.651    0.651
(3  HA  )  (4  HN  )    3.166    0.633    0.633 !
(3  HN  )  (4  HN  )    3.368    1.174    1.174 ! +/- 0.5 Å
(4  HN  )  (4  HA  )    3.253    0.651    0.651 !
(4  HN  )  (4  HB# )    3.206    1.241    1.241 ! HB2 to HB# +/- 0.6 Å
(4  HN  )  (4  HB# )    3.692    1.238    1.238 ! HB1 to HB# +/- 0.6 Å
(4  HB2 )  (4  HB1 )    2.048    0.328    0.410
(4  HA  )  (5  HN  )    3.290    0.658    1.658 ! overlap + 1.0 Å
(4  HN  )  (5  HN  )    3.105    0.621    0.621
(5  HN  )  (5  HA  )    3.374    0.675    0.675 !
(5  HN  )  (5  HB  )    3.494    1.199    0.699 ! - 0.5 Å
(5  HN  )  (5  HG1#)    3.169    0.634    0.634
(5  HA  )  (6  HN  )    2.874    0.575    0.575
(5  HN  )  (6  HN  )    3.171    0.634    1.634 ! overlap + 1.0 Å
(6  HN  )  (6  HA  )    3.110    0.622    0.622
(6  HA  )  (7  HN  )    3.121    0.624    0.624 !
(6  HN  )  (7  HN  )    3.306    0.661    1.161 ! small overlap + 0.5 Å
(7  HN  )  (7  HA  )    3.200    1.640    1.640 ! overlap +/- 1.0 Å
(7  HN  )  (7  HB2 )    3.070    0.614    0.614
(7  HB# )  (7  HE3 )    3.253    0.651    1.251 ! HB2 to HB# + 0.6 Å
(7  HA  )  (7  HE3 )    3.246    0.649    0.649
(7  HE1 )  (7  HD1 )    2.881    0.576    0.576
(7  HB# )  (7  HD1 )    3.207    0.641    0.641 ! HB2 to HB#
(7  HA  )  (8  HN  )    3.053    1.111    1.111 ! small overlap +/- 0.5 Å
(8  HN  )  (8  HA  )    3.148    1.130    1.130 ! small overlap +/- 0.5 Å
(8  HN  )  (8  HB2 )    3.319    0.664    0.664
(8  HN  )  (8  HG# )    3.421    0.684    1.684 ! HG2 to HG# + 1.0 Å
(8  HA  )  (9  HN  )    2.957    0.591    0.591
(8  HN  )  (9  HN  )    2.835    1.000    1.567 ! overlap +/- 1.0 Å
(8  HG2 )  (13 HN  )    3.740    0.748    0.748
(9  HN  )  (9  HA  )    3.075    0.615    0.615
(9  HN  )  (9  HB2 )    2.894    0.579    0.579
(9  HB# )  (9  HE3 )    3.073    0.615    1.215 ! HB2 to HB# + 0.6 Å
(9  HA  )  (9  HE3 )    3.507    0.701    0.701
(9  HE1 )  (9  HD1 )    2.816    0.563    0.563
(9  HB# )  (9  HD1 )    3.152    0.630    0.630 ! HB2 to HB#
(9  HA  )  (10 HN  )    3.264    0.653    0.653 !
(9  HB# )  (10 HN  )    3.394    1.279    1.279 ! HB2 to HB# +/- 0.6 Å
(10 HN  )  (10 HA  )    3.083    0.617    0.617
(10 HN  )  (10 HB# )    3.578    1.316    1.316 ! HB2 to HB# +/- 0.6 Å
(10 HN  )  (10 HD# )    3.235    0.647    1.647 ! HD2 to HD# + 1.0 Å
(10 HN  )  (10 HG# )    3.596    1.719    1.719 ! HG2 to HG# +/- 1.0 Å
(10 HA  )  (11 HN  )    3.101    0.620    0.620
(10 HN  )  (11 HN  )    3.175    0.635    0.635 !
(11 HN  )  (11 HA  )    3.123    0.625    0.625
(11 HN  )  (11 HB# )    2.850    0.570    0.570 ! HB2 to HB#
(11 HA  )  (12 HN  )    2.997    1.599    1.599 ! overlap +/- 1 Å
(12 HN  )  (12 HA  )    3.049    0.610    0.610
(12 HN  )  (12 HB# )    3.349    1.270    1.270 ! HB2 to HB# +/- 0.6 Å
(12 HN  )  (12 HB# )    2.869    1.174    1.174 ! HB1 to HB# +/- 0.6 Å
(13 HN  )  (12 HA  )    3.120    0.624    0.624 !
(13 HN  )  (13 HA  )    3.273    1.655    1.655 ! overlap +/- 1.0 Å
```

```
(13 HN  )   (13 HG# )      3.928     1.786     1.786 ! HG2 to HG# +/- 1.0 Å
(13 HN  )   (13 HB# )      3.296     1.259     1.259 ! HB2 to HB# +/- 0.6 Å
(13 HA  )   (14 HN  )      3.263     0.653     0.653
(13 HN  )   (14 HN  )      2.952     0.590     0.590
(14 HN  )   (14 HB# )      3.860     2.000     1.372 ! HB2 to HB# +/- 0.6 Å
(14 HN  )   (14 HB# )      3.718     2.000     1.344 ! HB1 to HB# +/- 0.6 Å
(14 HB2 )   (14 HB1 )      1.800     0.080     0.360
(14 HA  )   (15 HN  )      3.718     0.744     0.744
(14 HN  )   (15 HN  )      3.187     0.637     0.637
(15 HN  )   (15 HA  )      3.371     1.674     1.674 ! overlap +/- 1.0 Å
```

7.9.2 Peptide **1c**

```
(2  HN  )   (1  HA  )      2.508     0.502     0.502
(2  HN  )   (2  HA  )      2.789     0.558     0.558
(2  HN  )   (2  HB2 )      2.631     0.526     0.526
(2  HN  )   (2  HB1 )      2.887     0.577     0.577
(2  HA  )   (3  HN  )      2.308     0.462     0.462
(3  HN  )   (2  HB# )      3.100     0.620     1.220 ! HB2 to HB# + 0.6 Å
(3  HN  )   (2  HB# )      3.307     0.661     1.261 ! HB1 to HB# + 0.6 Å
(3  HN  )   (3  HA  )      2.764     0.553     0.553
(3  HN  )   (3  HB  )      2.676     0.535     1.535 ! overlap + 1.0 Å
(3  HN  )   (3  HD1#)      2.665     0.533     2.233 ! + 1.7 Å
(4  HN  )   (3  HA  )      2.751     0.550     0.550 !
(4  HN  )   (3  HN  )      2.776     0.555     0.555
(4  HN  )   (4  HA  )      2.744     0.549     0.549
(4  HN  )   (4  HB# )      3.054     0.611     1.211 ! HB2 to HB# + 0.6 Å
(4  HN  )   (4  HB# )      2.657     0.531     1.131 ! HB1 to HB# + 0.6 Å
(4  HB2 )   (4  HB1 )      1.800     0.080     0.360
(4  HN  )   (5  HN  )      2.643     0.529     0.529
(4  HA  )   (5  HN  )      2.828     0.566     0.566
(5  HN  )   (5  HA  )      2.787     0.557     0.557
(5  HN  )   (5  HG1#)      2.445     0.489     0.489
(5  HA  )   (6  HN  )      2.485     0.497     0.497
(6  HN  )   (6  HA  )      2.735     0.547     0.547
(6  HN  )   (6  HG# )      3.149     0.630     1.630 ! + 1.0 Å
(6  HN  )   (6  HB2 )      2.721     0.544     0.544
(7  HN  )   (6  HN  )      2.698     0.540     0.540
(7  HN  )   (6  HA  )      2.626     0.525     1.025 ! small overlap + 0.5 Å
(7  HN  )   (7  HA  )      2.608     0.522     0.522
(7  HE1 )   (7  HD1 )      2.432     0.486     0.486
(7  HN  )   (7  HB# )      2.821     0.564     1.164 ! HB2 to HB# + 0.6 Å
(7  HN  )   (7  HB# )      2.821     0.564     1.164 ! HB1 to HB# + 0.6 Å
(7  HD1 )   (7  HB# )      2.719     0.544     1.144 ! HB2 to HB# + 0.6 Å
(7  HD1 )   (7  HB# )      2.859     0.572     1.172 ! HB1 to HB# + 0.6 Å
(7  HA  )   (7  HE3 )      2.840     0.568     0.568
(7  HB# )   (7  HE3 )      2.889     0.578     1.178 ! HB1 to HB# + 0.6 Å
(7  HB# )   (7  HE3 )      2.606     0.521     1.121 ! HB2 to HB# + 0.6 Å
(7  HA  )   (8  HN  )      2.622     0.524     1.024 ! small overlap + 0.5 Å
(8  HA  )   (8  HN  )      2.583     0.517     0.517
(8  HN  )   (8  HB# )      2.836     0.567     1.167 ! HB2 to HB# + 0.6 Å
(8  HN  )   (8  HG# )      2.806     0.561     1.561 ! HG2 to HG# + 1.0 Å
(9  HN  )   (8  HA  )      2.638     0.528     0.528
(9  HN  )   (9  HA  )      2.701     0.540     0.540
(9  HN  )   (9  HB2 )      2.432     0.486     0.486
(9  HE1 )   (9  HD1 )      2.438     0.488     0.488
(9  HB2 )   (9  HD1 )      2.547     0.509     0.509
(9  HA  )   (9  HE3 )      2.853     0.571     0.571
(9  HB# )   (9  HE3 )      2.755     0.551     1.151 ! HB2 to HB# + 0.6 Å
(9  HA  )   (9  HD1 )      2.964     0.593     0.593
(9  HN  )   (9  HD1 )      2.994     0.599     0.599
(9  HN  )   (10 HN  )      2.542     0.508     0.508
(9  HA  )   (10 HN  )      3.154     0.631     0.631
(10 HN  )   (9  HB2 )      2.836     0.567     0.567
(10 HN  )   (10 HA  )      2.828     0.566     0.566
(10 HZ# )   (10 HG2 )      2.793     0.559     0.559
```

(10 HZ#)	(10 HD2)	2.956	0.591	0.591	
(10 HE2)	(10 HE1)	2.172	0.434	0.434	
(10 HZ#)	(10 HE2)	3.064	0.613	0.613	
(11 HN)	(10 HA)	3.028	0.606	0.606	
(11 HN)	(11 HA)	2.741	0.548	0.548	
(11 HN)	(11 HB#)	2.372	0.474	1.074	! HB2 to HB# + 0.6 Å
(12 HN)	(11 HA)	2.868	0.574	0.574	
(12 HN)	(12 HA)	2.720	0.544	0.544	
(12 HN)	(12 HB#)	2.541	0.508	1.108	! HB2 to HB# + 0.6 Å
(12 HN)	(12 HB#)	2.708	0.542	1.142	! HB1 to HB# + 0.6 Å
(12 HB2)	(12 HB1)	1.874	0.154	0.375	
(13 HN)	(12 HN)	2.622	0.524	0.524	
(13 HN)	(12 HA)	3.083	0.617	0.617	
(13 HN)	(12 HB#)	3.031	0.606	1.206	! HB1 to HB# + 0.6 Å
(13 HN)	(12 HB#)	3.030	0.606	1.206	! HB2 to HB# + 0.6 Å
(13 HN)	(13 HA)	2.656	0.531	0.531	
(13 HN)	(13 HG#)	2.922	0.584	1.584	! HG2 to HG# + 1.0 Å
(13 HN)	(13 HB#)	2.651	0.530	1.130	! HB2 to HB# + 0.6 Å
(13 HA)	(13 HG2)	2.913	0.583	0.583	
(14 HN)	(13 HA)	2.751	0.550	1.550	! overlap + 1.0 Å
(14 HN)	(13 HN)	2.438	0.488	0.488	
(14 HN)	(13 HB#)	2.912	0.582	1.182	! HB2 to HB# + 0.6 Å
(14 HN)	(14 HB#)	2.600	0.520	1.120	! HB2 to HB# + 0.6 Å
(14 HN)	(14 HB#)	2.948	0.590	1.190	! HB1 to HB# + 0.6 Å
(14 HN)	(14 HA)	2.760	0.552	0.552	
(14 HB2)	(14 HB1)	1.805	0.085	0.361	
(14 HN)	(15 HN)	2.743	0.549	0.549	
(14 HA)	(15 HN)	2.755	0.551	1.551	! overlap + 1.0 Å
(15 HN)	(15 HA)	2.541	0.508	0.508	

7.9.3 Peptide **2**

(2 HN)	(1 HA)	2.629	0.526	0.526	!
(2 HN)	(2 HA)	2.734	0.547	0.547	
(2 HN)	(2 HB#)	3.114	1.223	1.223	! HB2 to HB# +/- 0.6 Å
(2 HN)	(2 HB#)	3.127	1.225	1.225	! HB2 to HB# +/- 0.6 Å
(2 HA)	(3 HN)	2.580	0.516	0.516	!
(3 HN)	(3 HA)	3.105	0.621	0.621	
(3 HN)	(3 HD1#)	3.216	0.643	2.343	! + 1.7 Å
(3 HN)	(3 HB)	2.931	0.586	0.586	
(3 HA)	(3 HB)	2.741	0.548	0.548	
(3 HA)	(3 HD1#)	2.742	0.548	2.248	! + 1.7 Å
(3 HA)	(4 HN)	2.893	0.579	0.579	
(3 HN)	(4 HN)	3.172	0.634	1.134	! + 0.5 Å
(4 HN)	(4 HA)	3.042	0.608	0.608	
(4 HN)	(4 HB#)	2.882	1.176	1.176	! HB2 to HB# +/- 0.6 Å
(4 HN)	(4 HB#)	3.378	1.176	1.176	! HB1 to HB# +/- 0.6 Å
(4 HB2)	(4 HB1)	1.800	0.080	0.360	!
(4 HA)	(5 HN)	2.795	0.559	0.559	
(4 HN)	(5 HN)	2.927	0.585	0.585	
(5 HN)	(5 HA)	2.992	0.598	0.598	
(5 HN)	(5 HG1#)	3.071	0.614	0.614	
(5 HN)	(5 HB)	2.978	0.596	0.596	
(5 HA)	(5 HB)	2.930	0.586	0.586	
(5 HA)	(5 HG1#)	2.747	0.549	0.549	
(5 HA)	(6 HN)	2.597	0.519	1.519	! overlap + 1.0 Å
(5 HN)	(6 HN)	3.072	0.614	1.114	! + 0.5 Å
(6 HN)	(6 HA)	3.011	0.602	0.602	
(6 HN)	(6 HG2)	3.468	0.694	0.694	
(6 HN)	(6 HB#)	2.721	0.544	1.144	! HB2 to HB# + 0.6 Å
(6 HA)	(6 HB2)	2.882	0.576	0.576	
(6 HA)	(7 HN)	2.836	0.567	0.567	
(6 HB#)	(7 HN)	3.366	0.673	1.673	! HB2 to HB# + 1.0 Å
(6 HN)	(7 HN)	3.249	0.650	0.650	
(7 HN)	(7 HA)	2.880	0.576	0.576	

```
(7  HN  )   (7  HB# )     3.096    0.619    1.219 ! HB2 to HB# + 0.6 Å
(7  HN  )   (7  HB# )     3.211    0.642    1.242 ! HB1 to HB# + 0.6 Å
(7  HB2 )   (7  HD1 )     3.001    0.600    0.600
(7  HB1 )   (7  HD1 )     3.167    0.633    0.633
(7  HD1 )   (7  HE1 )     2.719    0.544    0.544
(7  HA  )   (7  HE3 )     3.110    0.622    0.622
(7  HA  )   (7  HD1 )     3.226    0.645    0.645
(7  HB# )   (7  HE3 )     3.263    0.653    1.253 ! HB1 to HB# + 0.6 Å
(7  HB# )   (7  HE3 )     3.280    0.656    1.256 ! HB2 to HB# + 0.6 Å
(7  HA  )   (8  HN  )     2.711    0.542    1.542 ! overlap + 1.0 Å
(8  HN  )   (8  HA  )     3.317    1.663    0.663 ! - 1.0 Å
(8  HN  )   (8  HB2 )     3.105    0.621    0.621
(8  HN  )   (8  HG# )     3.383    1.677    1.677 ! HG2 to HG# +/- 1.0 Å
(8  HA  )   (8  HG# )     3.155    0.631    0.631 ! HG2 to HG#
(8  HA  )   (9  HN  )     2.689    1.538    0.538 ! - 1.0 Å
(8  HN  )   (9  HN  )     3.014    0.603    0.603 !
(9  HN  )   (9  HA  )     2.673    0.535    0.535
(9  HN  )   (9  HB2 )     2.626    0.525    0.525
(9  HB# )   (9  HD1 )     2.755    0.551    1.151 ! HB2 to HB# + 0.6 Å
(9  HD1 )   (9  HE1 )     2.715    0.543    0.543
(9  HA  )   (9  HE3 )     3.143    0.629    0.629
(9  HA  )   (9  HD1 )     3.246    0.649    0.649
(9  HB# )   (9  HE3 )     2.926    0.585    1.185 ! HB2 to HB# + 0.6 Å
(9  HA  )   (9  HB2 )     3.044    0.609    0.609
(10 HN  )   (9  HB# )     3.085    0.617    1.617 ! HB2 to HB# + 1.0 Å
(10 HN  )   (9  HA  )     2.525    0.505    1.505 ! overlap + 1.0 Å
(10 HN  )   (10 HB# )     2.872    0.574    1.174 ! HB2 to HB# + 0.6 Å
(10 HN  )   (10 HA  )     2.524    0.505    0.505
(11 HN  )   (10 HA  )     2.629    0.526    1.526 ! overlap + 1.0 Å
(11 HN  )   (11 HB# )     2.770    0.554    1.154 ! HB2 to HB# + 0.6 Å
(11 HN  )   (11 HA  )     2.944    0.589    0.589
(11 HA  )   (12 HN  )     2.952    0.590    0.590
(12 HN  )   (12 HA  )     3.132    0.626    1.626 ! overlap + 1.0 Å
(12 HN  )   (12 HB# )     2.789    1.558    1.158 ! HB2 to HB# +/- 0.6 Å
(12 HN  )   (12 HB# )     3.115    1.623    1.223 ! HB1 to HB# +/- 0.6 Å
(12 HB2 )   (12 HB1 )     1.806    0.086    0.361
(13 HN  )   (12 HA  )     2.713    0.543    1.543 ! overlap + 1.0 Å
(13 HN  )   (12 HB# )     2.856    0.571    1.171 ! HB2 to HB# + 0.6 Å
(13 HN  )   (12 HB# )     3.115    0.623    1.223 ! HB1 to HB# + 0.6 Å
(13 HN  )   (13 HB2 )     3.019    0.604    0.604
(13 HN  )   (13 HB1 )     3.415    0.683    0.683
(13 HN  )   (13 HG# )     3.448    0.690    1.690 ! HG2 to HG# + 1.0 Å
(13 HN  )   (13 HA  )     3.174    1.635    0.635 ! - 1.0 Å
(14 HN  )   (13 HA  )     2.808    0.562    0.562
(14 HN  )   (14 HA  )     2.827    0.565    0.565
(14 HA  )   (15 HN  )     3.124    1.625    1.625 ! overlap +/- 1.0 Å
(15 HN  )   (15 HA  )     3.225    1.645    0.645 ! - 1.0 Å
```

7.9.4 Peptide **2c**

```
(2  HN  )   (1  HA  )     2.430    0.486    0.486
(1  HA  )   (1  HB2 )     2.350    0.470    0.470
(1  HA  )   (1  HB1 )     2.547    0.509    0.509
(2  HN  )   (1  HA  )     2.282    0.456    0.456
(2  HN  )   (2  HA  )     2.342    0.468    0.468
(2  HN  )   (2  HB2 )     2.477    0.495    0.495 !
(2  HN  )   (2  HB1 )     2.506    0.501    0.501 !
(3  HN  )   (2  HA  )     2.099    0.379    1.420 ! overlap water + 1.0 Å
(3  HN  )   (3  HA  )     2.548    0.510    0.510
(3  HN  )   (3  HB  )     2.475    0.495    0.495 !
(3  HN  )   (3  HD1#)     2.352    0.470    2.170 ! + 1.7 Å
(3  HN  )   (3  HG1#)     2.612    0.522    1.522 ! HG12 to HG1# + 1.0 Å
(4  HN  )   (3  HA  )     2.637    0.527    1.027 ! + 0.5 Å
(4  HN  )   (3  HN  )     2.602    0.520    0.520
(4  HN  )   (4  HA  )     2.511    0.502    0.502
(4  HN  )   (4  HB# )     2.609    0.522    1.122 ! HB2 to HB# + 0.6 Å
```

(4 HN)	(4 HB#)	2.431	0.486	1.086	! HB1 to HB# + 0.6 Å
(4 HB2)	(4 HB1)	1.800	0.080	0.360	
(4 HA)	(5 HN)	2.751	0.550	1.550	! overlap + 1.0 Å
(4 HN)	(5 HN)	2.400	0.480	0.480	
(5 HN)	(5 HA)	2.740	0.548	0.548	
(5 HN)	(5 HB)	2.612	0.522	0.522	!
(5 HN)	(5 HG1#)	2.446	0.489	1.489	! + 1.0 Å
(5 HA)	(6 HN)	2.270	0.454	0.454	
(6 HN)	(6 HA)	2.558	0.512	0.512	
(6 HN)	(6 HG#)	2.678	0.536	1.536	! HG2 to HG# + 1.0 Å
(6 HN)	(6 HB#)	2.468	0.494	1.094	! HB2 to HB# + 0.6 Å
(6 HA)	(6 HG2)	2.634	0.527	0.527	
(6 HA)	(6 HB2)	2.452	0.490	0.490	
(6 HA)	(7 HN)	2.554	0.511	0.511	
(7 HN)	(7 HA)	2.586	0.517	0.517	
(7 HN)	(7 HB#)	2.605	0.521	1.121	! HB2 to HB# + 0.6 Å
(7 HN)	(7 HB#)	2.645	0.529	1.129	! HB1 to HB# + 0.6 Å
(7 HB#)	(7 HD1)	2.387	0.477	1.477	! HB2 to HB# + 1.0 Å
(7 HB#)	(7 HD1)	2.437	0.487	1.487	! HB1 to HB# + 1.0 Å
(7 HD1)	(7 HE1)	2.130	0.410	0.426	
(7 HB#)	(7 HE3)	2.558	0.512	1.512	! HB1 to HB# + 1.0 Å
(7 HB#)	(7 HE3)	2.538	0.508	1.508	! HB2 to HB# + 1.0 Å
(7 HA)	(7 HE3)	2.508	0.502	0.502	
(7 HA)	(8 HN)	2.469	0.494	0.494	
(8 HN)	(8 HA)	2.630	0.526	0.526	
(8 HN)	(8 HG#)	2.432	0.486	1.486	! HG2 to HG# + 1.0 Å
(8 HN)	(8 HB#)	2.583	0.517	1.117	! HB2 to HB# + 0.6 Å
(8 HA)	(9 HN)	2.313	0.463	1.463	! overlap + 1.0 Å
(9 HN)	(9 HA)	2.414	0.483	0.483	
(9 HN)	(9 HB#)	2.380	0.476	1.076	! HB2 to HB# + 0.6 Å
(9 HB#)	(9 HD1)	2.276	0.455	1.455	! HB2 to HB# + 1.0 Å
(9 HD1)	(9 HE1)	2.101	0.381	0.420	
(9 HB#)	(9 HE3)	2.526	0.505	1.505	! HB2 to HB# + 1.0 Å
(9 HA)	(9 HE3)	2.541	0.508	1.108	! + 0.6 Å
(9 HA)	(9 HD1)	2.612	0.522	1.122	! + 0.6 Å
(9 HN)	(9 HD1)	2.544	0.509	0.509	
(9 HA)	(10 HN)	2.642	0.528	1.528	! overlap + 1.0 Å
(10 HN)	(9 HB#)	2.592	0.518	1.518	! HB2 to HB# + 1.0 Å
(10 HN)	(10 HA)	2.513	0.503	0.503	
(10 HN)	(10 HB#)	2.440	0.488	1.088	! HB2 to HB# + 0.6 Å
(10 HN)	(10 HB#)	2.585	0.517	1.117	! HB1 to HB# + 0.6 Å
(10 HB2)	(10 HB1)	2.164	0.433	0.433	
(10 HA)	(11 HN)	2.745	0.549	0.549	
(10 HA)	(12 HN)	2.649	0.530	1.530	! + 1.0 Å
(11 HN)	(11 HA)	2.584	0.517	0.517	
(11 HN)	(11 HB#)	2.500	0.500	1.100	! HB2 to HB# + 0.6 Å
(11 HN)	(11 HB#)	2.420	0.484	1.084	! HB1 to HB# + 0.6 Å
(11 HB2)	(11 HB1)	1.902	0.182	0.380	
(11 HA)	(11 HB1)	2.486	0.497	0.497	
(11 HA)	(11 HB2)	2.360	0.472	0.472	
(11 HA)	(14 HD2)	2.632	0.526	0.526	
(12 HN)	(11 HA)	2.736	0.547	1.547	! overlap + 1.0 Å
(12 HN)	(12 HA)	2.563	0.513	0.513	
(12 HN)	(12 HB#)	2.325	0.465	1.065	! HB2 to HB# + 0.6 Å
(12 HN)	(12 HB#)	2.330	0.466	1.066	! HB1 to HB# + 0.6 Å
(12 HA)	(13 HN)	2.553	0.511	1.511	! overlap + 1.0 Å
(12 HN)	(13 HN)	2.285	0.457	0.457	
(13 HN)	(12 HB#)	2.691	0.538	1.538	! HB2 to HB# + 1.0 Å
(13 HN)	(12 HB#)	2.718	0.544	1.544	! HB1 to HB# + 1.0 Å
(13 HN)	(13 HA)	2.372	0.474	0.474	
(13 HN)	(13 HG#)	2.490	0.498	1.498	! HG2 to HG# + 1.0 Å
(13 HN)	(13 HB#)	2.209	0.442	1.042	! HB2 to HB# + 0.6 Å
(13 HA)	(13 HG2)	2.541	0.508	0.508	
(13 HA)	(14 HN)	2.481	0.496	1.496	! overlap + 1.0 Å
(14 HN)	(14 HA)	2.446	0.489	0.489	
(14 HZ#)	(14 HE#)	2.427	0.485	0.485	! HE2 to HE#
(14 HZ#)	(14 HE#)	2.551	0.510	0.510	! HE1 to HE#
(14 HZ#)	(14 HD#)	2.554	0.511	1.111	! HD2 to HD# + 0.6 Å
(14 HN)	(14 HD#)	2.236	0.447	2.147	! HD2 to HD# + 1.7 Å
(14 HZ#)	(14 HG#)	2.609	0.522	1.522	! HG2 to HG# + 1 Å
(14 HN)	(14 HG#)	2.564	0.513	1.513	! HG2 to HG# + 1 Å

(14 HN)	(14 HB#)	2.689	0.538	1.138	! HB2 to HB# + 0.6 Å
(14 HE2)	(14 HE1)	2.090	0.370	0.418	
(15 HN)	(14 HA)	2.388	0.478	1.478	! overlap + 1.0 Å
(15 HN)	(14 HN)	2.429	0.486	0.486	
(15 HN)	(15 HA)	2.381	0.476	0.476	
(15 HN)	(15 HD1#)	2.705	0.541	2.541	! + 2.0 Å
(15 HN)	(15 HB#)	2.252	0.450	1.050	! HB2 to HB# + 0.6 Å
(15 HN)	(15 HG)	2.499	0.500	0.500	

7.9.5 Peptide **3**

(2 HN)	(1 HA)	2.534	0.507	0.507	!
(2 HN)	(1 HB#)	3.218	0.644	1.644	! HB2 to HB# + 1.0 Å
(2 HN)	(2 HA)	2.578	0.516	0.516	
(2 HN)	(2 HB#)	2.859	0.572	1.172	! HB2 to HB# + 0.6 Å
(2 HA)	(3 HN)	2.604	0.521	0.521	!
(2 HN)	(5 HG1#)	3.398	0.680	2.680	! + 2.0 Å
(3 HN)	(3 HA)	3.038	0.608	0.608	
(3 HN)	(3 HB)	2.946	0.589	0.589	
(3 HN)	(3 HG1#)	2.883	0.577	1.577	! HG12 to HG1# + 1.0 Å
(3 HN)	(3 HD1#)	2.934	0.587	1.587	! + 1.0 Å
(3 HN)	(4 HN)	3.026	0.605	0.605	
(4 HN)	(3 HA)	2.758	0.552	0.552	
(4 HN)	(3 HD1#)	3.141	0.628	3.028	! + 2.4 Å
(4 HN)	(3 HG1#)	3.536	0.707	1.707	! HG12 to HG1# + 1.0 Å
(4 HN)	(3 HB)	3.446	0.689	0.689	
(4 HN)	(4 HA)	2.978	0.596	0.596	
(4 HN)	(4 HB#)	3.197	1.639	1.239	! HB2 to HB# +/- 0.6 Å
(4 HN)	(4 HB#)	2.768	1.554	1.154	! HB1 to HB# +/- 0.6 Å
(4 HB2)	(4 HB1)	1.800	0.080	0.360	
(4 HA)	(5 HN)	2.928	0.586	0.586	
(4 HN)	(5 HN)	2.812	0.562	0.562	
(5 HA)	(5 HN)	3.015	0.603	0.603	
(5 HN)	(5 HG1#)	2.775	1.555	2.555	! + 2.0 Å
(5 HN)	(5 HB)	3.065	0.613	0.613	
(6 HN)	(5 HA)	2.444	0.489	1.489	! overlap + 1.0 Å
(6 HN)	(5 HN)	2.931	0.586	1.586	! overlap + 1.0 Å
(6 HN)	(5 HG1#)	2.995	0.599	1.599	! + 1.0 Å
(6 HN)	(6 HA)	2.844	0.569	0.569	
(6 HN)	(6 HB#)	2.735	0.547	1.147	! HB2 to HB# + 0.6 Å
(6 HA)	(7 HN)	2.872	0.574	0.574	
(6 HN)	(7 HN)	2.841	0.568	1.068	! overlap + 0.5 Å
(7 HD1)	(5 HG1#)	3.475	0.695	0.695	
(7 HN)	(5 HG1#)	3.278	0.656	1.656	! + 1.0 Å
(7 HN)	(6 HB#)	3.373	0.675	1.675	! HB2 to HB# + 1.0 Å
(7 HN)	(7 HB#)	3.032	0.606	1.206	! HB2 to HB# + 0.6 Å
(7 HN)	(7 HA)	2.984	0.597	0.597	
(7 HB#)	(7 HE3)	3.088	0.618	1.218	! HB2 to HB# + 0.6 Å
(7 HB#)	(7 HE3)	2.977	0.595	1.195	! HB1 to HB# + 0.6 Å
(7 HB#)	(7 HD1)	2.909	0.582	1.582	! HB1 to HB# + 1.0 Å
(7 HB#)	(7 HD1)	2.912	0.582	1.582	! HB2 to HB# + 1.0 Å
(7 HD1)	(7 HE1)	2.569	0.514	0.514	
(7 HN)	(7 HB#)	3.132	0.626	1.226	! HB1 to HB# + 0.6 Å
(7 HA)	(8 HN)	2.812	0.562	0.562	
(8 HN)	(8 HA)	2.601	0.520	0.520	
(8 HN)	(8 HG#)	3.340	0.668	1.668	! HG2 to HG# + 1.0 Å
(8 HN)	(8 HB#)	2.967	0.593	1.193	! HB2 to HB# + 0.6 Å
(8 HA)	(9 HN)	2.606	0.521	0.521	!
(9 HN)	(9 HA)	2.658	0.532	0.532	
(9 HN)	(9 HB#)	2.801	0.560	1.160	! HB2 to HB# + 0.6 Å
(9 HN)	(9 HB#)	2.719	0.544	1.144	! HB1 to HB# + 0.6 Å
(9 HB#)	(9 HD1)	3.072	0.614	1.614	! HB2 to HB# + 1.0 Å
(9 HB#)	(9 HD1)	2.866	0.573	1.573	! HB1 to HB# + 1.0 Å
(9 HB#)	(9 HE3)	3.130	0.626	1.626	! HB2 to HB# + 1.0 Å
(9 HB#)	(9 HE3)	3.256	0.651	1.651	! HB1 to HB# + 1.0 Å
(9 HD1)	(9 HE1)	2.472	0.494	0.494	

(9 HN)	(9 HD1)	2.988	0.598	0.598	
(9 HA)	(10 HN)	2.704	0.541	1.541	! overlap + 1.0 Å
(10 HN)	(10 HA)	3.001	0.600	0.600	
(10 HN)	(10 HB#)	2.745	0.549	1.149	! HB2 to HB# + 0.6 Å
(10 HN)	(10 HG#)	3.034	0.607	1.607	! HG2 to HG# + 1.0 Å
(10 HA)	(11 HN)	2.973	0.595	0.595	
(10 HN)	(11 HN)	2.606	0.521	0.521	!
(11 HN)	(11 HA)	2.755	0.551	0.551	
(11 HN)	(11 HB2)	2.486	0.497	0.497	
(11 HA)	(12 HN)	2.715	0.543	1.543	! overlap + 1.0 Å
(12 HN)	(12 HA)	2.947	0.589	0.589	
(12 HN)	(12 HB#)	2.979	0.596	1.196	! HB2 to HB# + 0.6 Å
(12 HN)	(12 HB#)	2.492	0.498	1.098	! HB1 to HB# + 0.6 Å
(12 HB2)	(12 HB1)	1.874	0.154	0.375	
(12 HB2)	(12 HD#)	2.934	0.587	0.587	
(12 HB1)	(12 HD#)	2.837	0.567	0.567	
(13 HN)	(12 HA)	2.788	0.558	1.058	! overlap + 0.5 Å
(13 HN)	(12 HB#)	3.299	0.660	1.260	! HB2 to HB# + 0.6 Å
(13 HN)	(12 HB#)	3.265	0.653	1.253	! HB1 to HB# + 0.6 Å
(13 HN)	(13 HA)	3.125	0.625	0.625	
(13 HN)	(13 HG2)	3.160	0.632	0.632	
(13 HN)	(13 HB2)	2.929	0.586	0.586	
(13 HA)	(14 HN)	2.793	0.559	0.559	
(13 HN)	(14 HN)	2.683	0.537	0.537	
(14 HN)	(14 HA)	2.807	0.561	0.561	
(14 HN)	(14 HD#)	3.789	0.758	1.758	! HD2 to HD# + 1.0 Å
(14 HN)	(14 HB#)	3.225	1.645	1.245	! HB2 to HB# +/- 0.6 Å
(14 HN)	(14 HG#)	3.559	0.712	1.712	! HG2 to HG# + 1.0 Å
(15 HN)	(14 HA)	3.098	1.620	1.620	! overlap +/- 1.0 Å
(15 HN)	(15 HA)	3.148	1.630	1.630	! overlap +/- 1.0 Å

7.9.6 Peptide **3c**

(2 HN)	(1 HA)	2.430	0.486	0.486	
(2 HN)	(2 HA)	2.940	0.588	0.588	
(2 HN)	(2 HB#)	2.684	0.537	1.137	! HB2 to HB# + 0.6 Å
(2 HN)	(2 HB#)	2.893	0.579	1.179	! HB1 to HB# + 0.6 Å
(2 HN)	(5 HG1#)	3.364	0.673	3.073	! + 2.4 Å
(3 HN)	(2 HA)	2.257	0.451	0.451	
(3 HN)	(2 HB2)	3.310	0.662	0.662	!
(3 HN)	(2 HB1)	3.161	0.632	0.632	!
(3 HN)	(3 HA)	2.584	0.517	1.517	! overlap + 1.0 Å
(3 HN)	(3 HB)	2.615	0.523	1.523	! + 1.0 Å
(3 HN)	(3 HG1#)	2.692	0.538	1.538	! HG12 to HG1# + 1.0 Å
(3 HN)	(3 HG1#)	3.005	0.601	1.601	! HG11 to HG1# + 1.0 Å
(3 HN)	(3 HG2#)	2.490	0.498	1.498	! + 1.0 Å
(3 HA)	(4 HN)	2.584	0.517	1.517	! overlap + 1.0 Å
(4 HN)	(4 HA)	2.843	0.569	0.569	
(4 HN)	(4 HB#)	3.128	1.226	1.226	! HB2 to HB# +/- 0.6 Å
(4 HN)	(4 HB#)	2.635	1.127	1.127	! HB1 to HB# +/- 0.6 Å
(4 HB2)	(4 HB1)	1.809	0.089	0.362	
(4 HA)	(5 HN)	3.089	0.618	0.618	
(4 HN)	(5 HN)	2.627	0.525	0.525	
(5 HN)	(5 HG1#)	2.733	0.547	2.247	! + 1.7 Å
(5 HN)	(5 HB)	2.926	0.585	0.585	
(5 HN)	(5 HA)	3.037	0.607	0.607	
(5 HA)	(6 HN)	2.439	0.488	0.488	
(5 HG1#)	(6 HN)	2.994	0.599	2.999	! + 2.4 Å
(5 HN)	(6 HN)	2.700	0.540	1.540	! overlap + 1.0 Å
(5 HG1#)	(7 HN)	3.415	0.683	3.083	! + 2.4 Å
(6 HN)	(6 HA)	2.702	0.540	0.540	
(6 HN)	(6 HB#)	2.786	0.557	0.557	! HB2 to HB#
(6 HN)	(6 HG#)	2.552	0.510	0.510	! HG2 to HG#
(6 HN)	(6 HD#)	3.043	0.609	2.309	! HD2 to HD# + 1.7 Å
(6 HA)	(7 HN)	2.777	0.555	1.555	! overlap + 1.0 Å
(7 HN)	(7 HA)	3.062	0.612	0.612	

```
(7  HN  )   (7  HB# )      3.093     0.619     1.219 ! HB2 to HB# + 0.6 Å
(7  HN  )   (7  HB# )      3.159     0.632     1.232 ! HB1 to HB# + 0.6 Å
(7  HE1 )   (7  HD1 )      2.710     0.542     0.542
(7  HA  )   (7  HD1 )      2.974     0.595     0.595
(7  HB# )   (7  HD1 )      2.750     0.550     0.550 ! HB2 to HB#
(7  HB# )   (7  HD1 )      2.808     0.562     0.562 ! HB1 to HB#
(7  HA  )   (7  HE3 )      2.965     0.593     0.593
(7  HA  )   (8  HN  )      2.753     0.551     1.551 ! overlap + 1.0 Å
(8  HN  )   (8  HA  )      2.980     0.596     0.596
(8  HN  )   (8  HB# )      2.929     0.586     1.186 ! HB2 to HB# + 0.6 Å
(8  HN  )   (8  HG# )      2.910     0.582     1.582 ! HG2 to HG# + 1.0 Å
(8  HA  )   (9  HN  )      2.464     0.493     1.493 ! overlap + 1.0 Å
(8  HN  )   (9  HN  )      2.927     0.585     0.585
(9  HN  )   (8  HG# )      3.124     0.625     1.625 ! HG2 to HG# + 1.0 Å
(9  HN  )   (9  HA  )      2.451     0.490     1.490 ! overlap + 1.0 Å
(9  HN  )   (9  HB# )      2.593     0.519     1.119 ! HB2 to HB# + 0.6 Å
(9  HE1 )   (9  HD1 )      2.428     0.486     0.486
(9  HB# )   (9  HD1 )      2.641     0.528     0.528 ! HB2 to HB#
(9  HA  )   (10 HN  )      3.421     1.684     1.684 ! +/- 1.0 Å
(9  HN  )   (10 HN  )      2.728     0.546     0.546
(9  HA  )   (11 HN  )      2.736     0.547     1.547 ! overlap + 1.0 Å
(9  HA  )   (12 HN  )      3.376     0.675     0.675
(10 HN  )   (10 HA  )      3.015     0.603     0.603
(10 HA  )   (10 HB2 )      2.886     0.577     0.577 !
(10 HN  )   (10 HB2 )      2.662     0.532     0.532 !
(10 HN  )   (10 HB1 )      2.711     0.542     0.542 !
(10 HA  )   (11 HN  )      3.128     0.626     0.626
(10 HN  )   (11 HN  )      2.878     0.576     0.576
(10 HB1 )   (11 HN  )      2.774     0.555     0.555
(10 HN  )   (12 HN  )      2.782     0.556     1.556 ! near diagonal + 1.0 Å
(10 HA  )   (13 HN  )      3.292     0.658     0.658
(11 HN  )   (11 HA  )      2.950     0.590     0.590
(11 HN  )   (11 HB# )      2.780     0.556     1.156 ! HB2 to HB# + 0.6 Å
(11 HA  )   (12 HN  )      3.344     0.669     0.669
(11 HN  )   (12 HN  )      2.556     0.511     0.511
(11 HA  )   (14 HD# )      2.784     0.557     1.557 ! HD2 to HD# + 1.0 Å
(12 HN  )   (12 HA  )      2.901     0.580     0.580
(12 HN  )   (12 HB# )      2.375     0.475     1.075 ! HB2 to HB# + 0.6 Å
(12 HN  )   (12 HB# )      2.752     0.550     1.150 ! HB1 to HB# + 0.6 Å
(12 HB2 )   (12 HB1 )      1.800     0.080     0.360
(12 HA  )   (13 HN  )      3.250     0.650     0.650
(12 HB1 )   (13 HN  )      3.042     0.608     0.608 !
(12 HB2 )   (13 HN  )      3.067     0.613     0.613 !
(12 HN  )   (13 HN  )      2.631     0.526     0.526
(13 HN  )   (13 HA  )      2.784     0.557     0.557
(13 HN  )   (13 HB# )      2.486     0.497     1.097 ! HB2 to HB# + 0.6 Å
(13 HN  )   (13 HG# )      2.903     0.581     1.581 ! HG2 to HG# + 1.0 Å
(13 HA  )   (14 HN  )      3.029     0.606     0.606
(13 HN  )   (14 HN  )      2.679     0.536     0.536
(13 HB# )   (14 HN  )      2.918     0.584     1.584 ! HB2 to HB# + 1.0 Å
(14 HZ# )   (10 HG1 )      2.816     0.563     0.563 ! HG2
(14 HZ# )   (10 HG2 )      2.428     0.486     0.486 ! HG1
(14 HN  )   (14 HA  )      2.964     0.593     0.593
(14 HZ# )   (14 HE2 )      2.894     0.579     0.579
(14 HZ# )   (14 HE1 )      2.522     0.504     0.504
(14 HE2 )   (14 HE1 )      2.161     0.432     0.432
(14 HZ# )   (14 HG# )      3.239     0.648     1.648 ! HG2 to HG# + 1.0 Å
(14 HZ# )   (14 HD# )      3.022     0.604     1.204 ! HD2 to HD# + 0.6 Å
(14 HN  )   (14 HG# )      3.114     0.623     1.623 ! HG2 to HG# + 1.0 Å
(14 HN  )   (14 HD# )      3.228     0.646     2.346 ! HD2 to HD# + 1.7 Å
(14 HN  )   (14 HB# )      2.393     1.079     1.079 ! HB2 to HB# +/- 0.6 Å
(14 HZ# )   (14 HB# )      2.832     0.566     1.566 ! HB2 to HB# + 1.0 Å
(14 HN  )   (14 HB# )      3.024     1.205     1.205 ! HB1 to HB# +/- 0.6 Å
(14 HE2 )   (14 HD# )      2.638     0.528     0.528 ! HD2 to HD#
(14 HE1 )   (14 HD# )      2.760     0.552     0.552 ! HD2 to HD#
(14 HA  )   (15 HN  )      3.146     1.629     1.629 ! overlap +/- 1.0 Å
(15 HN  )   (15 HA  )      2.800     0.560     0.560
(15 HN  )   (15 HD1#)      3.523     0.705     2.705 ! + 2.0 Å
(15 HN  )   (15 HG  )      3.233     0.647     0.647
(15 HN  )   (15 HB# )      2.417     0.483     1.083 ! HB2 to HB# + 0.6 Å
```

7.9.7 Peptide **4**

(2 HN)	(1 HA)	2.446	0.489	0.489	
(2 HN)	(1 HB#)	3.126	1.225	1.225	! HB2 to HB# +/- 0.6 Å
(2 HN)	(1 HB#)	3.128	1.226	1.226	! HB1 to HB# +/- 0.6 Å
(2 HN)	(2 HA)	2.654	0.531	0.531	
(2 HN)	(2 HB#)	2.785	0.557	1.157	! HB2 to HB# + 0.6 Å
(2 HA)	(3 HN)	2.392	0.478	1.078	! water overlap + 0.6 Å
(3 HN)	(2 HB#)	3.043	0.609	1.209	! HB2 to HB# + 0.6 Å
(3 HN)	(3 HA)	2.819	0.564	0.564	
(3 HN)	(3 HB)	2.739	0.548	0.548	
(3 HN)	(3 HD1#)	2.710	0.542	2.242	! + 1.7 Å
(3 HN)	(3 HG1#)	2.786	0.557	1.557	! HG12 to HG1# + 1.0 Å
(3 HA)	(4 HN)	2.756	0.551	0.551	
(4 HN)	(4 HA)	2.812	0.562	0.562	
(4 HN)	(4 HB#)	3.003	0.601	1.201	! HB2 to HB# + 0.6 Å
(4 HN)	(4 HB#)	2.676	0.535	1.135	! HB1 to HB# + 0.6 Å
(4 HB2)	(4 HB1)	1.800	0.080	0.360	
(4 HA)	(5 HN)	2.956	0.591	0.591	
(4 HN)	(5 HN)	2.703	0.541	0.541	
(5 HN)	(5 HA)	3.078	0.616	0.616	
(5 HN)	(5 HG1#)	2.618	0.524	2.524	! + 2.0 Å
(5 HN)	(5 HB)	3.022	1.604	0.604	! − 1.0 Å
(5 HA)	(6 HN)	2.454	0.491	0.491	
(6 HN)	(5 HG1#)	2.893	0.579	0.579	
(6 HN)	(6 HA)	2.702	0.540	0.540	
(6 HN)	(6 HB#)	2.563	0.513	1.113	! HB2 to HB# + 0.6 Å
(6 HA)	(7 HN)	2.756	0.551	0.551	
(7 HN)	(6 HB#)	3.108	0.622	1.222	! HB2 to HB# + 0.6 Å
(7 HN)	(7 HA)	2.695	0.539	0.539	
(7 HN)	(7 HB#)	2.873	0.575	1.175	! HB2 to HB# + 0.6 Å
(7 HN)	(7 HB#)	2.953	0.591	1.191	! HB1 to HB# + 0.6 Å
(7 HE1)	(7 HD1)	2.429	0.486	0.486	
(7 HB#)	(7 HD1)	2.792	0.558	1.158	! HB2 to HB# + 0.6 Å
(7 HB#)	(7 HD1)	2.540	0.508	1.108	! HB1 to HB# + 0.6 Å
(7 HA)	(7 HE3)	2.780	0.556	0.556	
(7 HB#)	(7 HE3)	2.925	0.585	1.185	! HB1 to HB# + 0.6 Å
(7 HB#)	(7 HE3)	2.906	0.581	1.181	! HB2 to HB# + 0.6 Å
(7 HA)	(8 HN)	2.744	0.549	0.549	
(7 HN)	(8 HN)	2.650	0.530	0.530	
(8 HN)	(8 HA)	2.901	0.580	0.580	
(8 HN)	(8 HG#)	2.931	0.586	1.586	! HG2 to HG# + 1.0 Å
(8 HN)	(8 HB#)	2.860	0.572	1.172	! HB2 to HB# + 0.6 Å
(8 HA)	(9 HN)	2.511	0.502	0.502	
(9 HN)	(9 HA)	2.715	0.543	0.543	
(9 HN)	(9 HB#)	2.452	0.490	1.090	! HB2 to HB# + 0.6 Å
(9 HE1)	(9 HD1)	2.408	0.482	0.482	!
(9 HB#)	(9 HD1)	2.449	0.490	1.090	! HB2 to HB# + 0.6 Å
(9 HA)	(9 HE3)	2.858	0.572	0.572	
(9 HB#)	(9 HE3)	2.644	0.529	1.129	! HB2 to HB# + 0.6 Å
(9 HA)	(10 HN)	2.925	0.585	0.585	
(9 HN)	(10 HN)	2.600	0.520	0.520	
(10 HN)	(9 HB#)	2.905	0.581	1.181	! HB2 to HB# + 0.6 Å
(10 HN)	(10 HA)	2.857	0.571	0.571	
(10 HN)	(10 HG#)	3.000	0.600	1.600	! HG2 to HG# + 1.0 Å
(10 HN)	(10 HB#)	2.971	0.594	1.194	! HB2 to HB# + 0.6 Å
(10 HN)	(10 HD2)	2.726	0.545	2.245	! HD2 to HD# + 1.7 Å
(10 HA)	(11 HN)	2.809	0.562	0.562	
(10 HN)	(11 HN)	2.690	0.538	2.038	! overlap near diag. + 1.5 Å
(11 HN)	(11 HA)	2.798	0.560	0.560	
(11 HN)	(11 HB#)	2.505	0.501	1.101	! HB2 to HB# + 0.6 Å
(11 HA)	(12 HN)	2.560	0.512	1.512	! overlap + 1.0 Å
(12 HN)	(12 HA)	2.607	0.521	0.521	
(12 HN)	(12 HB#)	2.767	1.553	1.153	! HB2 to HB# +/- 0.6 Å
(12 HN)	(12 HB#)	2.434	1.087	1.087	! HB1 to HB# +/- 0.6 Å
(12 HB2)	(12 HB1)	1.820	0.100	0.364	
(12 HA)	(13 HN)	2.641	0.528	0.528	
(13 HN)	(12 HB#)	2.930	0.586	1.186	! HB2 to HB# + 0.6 Å
(13 HN)	(12 HB#)	2.963	0.593	1.193	! HB1 to HB# + 0.6 Å

```
(13 HN  )   (13 HA  )      2.449     0.490     0.490
(13 HA  )   (14 HN  )      2.449     0.490     1.490 ! overlap + 1.0 Å
(14 HN  )   (14 HA  )      2.796     0.559     0.559
(14 HA  )   (15 HN  )      2.848     0.570     0.570
(15 HN  )   (15 HA  )      2.848     0.570     0.570
(15 HN  )   (15 HB# )      2.646     0.529     1.129 ! HB2 to HB# + 0.6 Å
```

7.9.8 Peptide **4c**

```
(1  HA  )   (1  HB# )      2.776     0.555     0.555 ! HB2 to HB#
(1  HA  )   (1  HB# )      2.872     0.574     0.574 ! HB1 to HB#
(2  HN  )   (1  HA  )      2.494     0.499     0.499
(2  HN  )   (1  HB# )      3.127     0.625     1.225 ! HB2 to HB# + 0.6 Å
(2  HN  )   (2  HA  )      2.590     0.518     0.518
(2  HN  )   (2  HB# )      2.799     0.560     1.160 ! HB2 to HB# + 0.6 Å
(2  HA  )   (3  HN  )      2.478     0.496     0.496
(3  HN  )   (3  HA  )      2.685     0.537     0.537
(3  HN  )   (3  HB  )      2.873     1.075     1.075 ! small overlap +/- 0.5 Å
(4  HN  )   (3  HA  )      2.674     0.535     0.535 !
(4  HN  )   (3  HN  )      2.983     0.597     0.597 !
(4  HN  )   (3  HD1#)      2.993     0.599     2.599 ! + 2.0 Å
(4  HN  )   (3  HB  )      3.350     1.170     1.170 ! +/- 0.5 Å
(4  HN  )   (4  HA  )      2.849     0.570     0.570
(4  HN  )   (4  HB# )      3.044     1.609     1.209 ! HB2 to HB# +/- 0.6 Å
(4  HN  )   (4  HB# )      2.741     1.548     1.148 ! HB1 to HB# +/- 0.6 Å
(4  HB2 )   (4  HB1 )      1.953     0.233     0.391
(4  HA  )   (5  HN  )      2.919     0.584     0.584
(4  HN  )   (5  HN  )      2.810     0.562     0.562 !
(5  HA  )   (5  HN  )      3.044     0.609     0.609
(5  HN  )   (5  HB  )      3.030     0.606     0.606
(5  HN  )   (5  HG1#)      2.762     0.552     1.552 ! + 1.0 Å
(6  HN  )   (5  HA  )      2.478     0.496     1.496 ! overlap + 1.0 Å
(6  HN  )   (5  HN  )      2.891     0.578     1.078 ! small overlap + 0.5 Å
(6  HN  )   (5  HB  )      2.935     1.087     1.087 ! small overlap +/- 0.5 Å
(6  HN  )   (6  HA  )      2.782     0.556     0.556
(6  HN  )   (6  HB# )      2.629     0.526     1.126 ! HB2 to HB# + 0.6 Å
(6  HA  )   (7  HN  )      2.849     0.570     0.570
(6  HN  )   (7  HN  )      3.102     0.620     0.620 !
(7  HN  )   (6  HB# )      3.105     0.621     1.221 ! HB2 to HB# + 0.6 Å
(7  HN  )   (7  HA  )      2.920     0.584     0.584
(7  HN  )   (7  HB# )      2.965     0.593     1.193 ! HB2 to HB# + 0.6 Å
(7  HB# )   (7  HE3 )      2.912     0.582     1.182 ! HB2 to HB# + 0.6 Å
(7  HA  )   (7  HE3 )      3.029     0.606     0.606
(7  HE1 )   (7  HD1 )      2.457     0.491     0.491
(7  HB# )   (7  HD1 )      2.719     0.544     1.144 ! HB2 to HB# + 0.6 Å
(7  HA  )   (7  HD1 )      2.833     1.167     1.167 ! +/- 0.6 Å
(7  HA  )   (8  HN  )      2.798     0.560     0.560
(7  HN  )   (8  HN  )      3.041     0.608     1.608 ! overlap + 1.0 Å
(8  HN  )   (8  HA  )      2.962     0.592     0.592
(8  HN  )   (8  HB# )      2.977     0.595     1.195 ! HB2 to HB# + 0.6 Å
(8  HN  )   (8  HG# )      3.108     0.622     1.622 ! HG2 to HG# + 1.0 Å
(8  HA  )   (9  HN  )      2.712     0.542     1.042 ! small overlap + 0.5 Å
(9  HN  )   (9  HA  )      2.865     0.573     0.573
(9  HN  )   (9  HB# )      2.672     0.534     1.134 ! HB2 to HB# + 0.6 Å
(9  HB# )   (9  HE3 )      2.741     0.548     1.148 ! HB2 to HB# + 0.6 Å
(9  HA  )   (9  HE3 )      2.958     0.592     0.592
(9  HE1 )   (9  HD1 )      2.382     0.476     0.476
(9  HB# )   (9  HD1 )      2.589     0.518     1.118 ! HB2 to HB# + 0.6 Å
(9  HA  )   (9  HD1 )      2.862     1.172     1.172 ! +/- 0.6 Å
(9  HA  )   (10 HN  )      3.127     0.625     0.625 !
(9  HN  )   (10 HN  )      2.814     0.563     1.563 ! overlap + 1.0 Å
(10 HN  )   (9  HB# )      3.085     0.617     1.217 ! HB2 to HB# + 0.6 Å
(10 HN  )   (10 HA  )      2.988     0.598     0.598
(10 HZ# )   (10 HE# )      2.836     0.567     0.567 ! HE2 to HE#
(10 HZ# )   (10 HE# )      2.789     0.558     0.558 ! HE1 to HE#
(10 HE2 )   (10 HE1 )      1.867     0.147     0.373
(10 HZ# )   (14 HG1 )      2.748     0.550     1.050 ! HG2 to HG1 overlap + 0.5 Å
```

```
(10 HZ# )   (14 HG2 )      2.718     0.544     1.044 ! HG1 to HG2 overlap + 0.5 Å
(11 HN  )   (10 HA  )      2.939     0.588     0.588
(11 HN  )   (10 HN  )      3.105     0.621     0.621 !
(11 HN  )   (11 HA  )      2.920     0.584     0.584
(11 HN  )   (11 HB# )      2.778     0.556     1.156 ! HB2 to HB# + 0.6 Å
(11 HA  )   (12 HN  )      2.970     0.594     0.594
(11 HN  )   (12 HN  )      2.857     0.571     1.571 ! overlap + 1.0 Å
(12 HN  )   (12 HA  )      2.807     0.561     0.561
(12 HN  )   (12 HB# )      2.863     1.173     1.173 ! HB2 to HB# +/- 0.6 Å
(12 HN  )   (12 HB# )      2.550     1.110     1.110 ! HB1 to HB# +/- 0.6 Å
(12 HB2 )   (12 HB1 )      1.800     0.080     0.360
(13 HN  )   (12 HA  )      2.918     0.584     0.584
(13 HN  )   (12 HN  )      2.961     0.592     0.592
(13 HN  )   (12 HB# )      3.234     0.647     0.647 ! HB2 to HB#
(13 HN  )   (12 HB# )      3.127     0.625     0.625 ! HB1 to HB#
(13 HN  )   (13 HA  )      2.930     0.586     0.586
(13 HN  )   (13 HG# )      2.975     0.595     1.595 ! HG2 to HG# + 1.0 Å
(13 HN  )   (13 HB# )      3.116     1.223     1.223 ! HB2 to HB# +/- 0.6 Å
(13 HN  )   (13 HB# )      2.853     1.171     1.171 ! HB1 to HB# +/- 0.6 Å
(13 HA  )   (14 HN  )      2.744     0.549     1.049 ! overlap + 0.5 Å
(13 HN  )   (14 HN  )      2.899     0.580     1.580 ! overlap + 1.0 Å
(14 HN  )   (14 HA  )      2.941     0.588     0.588
(14 HN  )   (14 HG# )      3.001     0.600     1.600 ! HG2 to HG# + 1.0 Å
(14 HN  )   (14 HG# )      3.052     0.610     1.610 ! HG1 to HG# + 1.0 Å
(14 HN  )   (14 HB# )      2.905     0.581     1.181 ! HB2 to HB# + 0.6 Å
(14 HN  )   (14 HB# )      2.617     0.523     1.123 ! HB1 to HB# + 0.6 Å
(14 HA  )   (15 HN  )      2.616     0.523     1.523 ! overlap + 1.0 Å
(15 HN  )   (15 HA  )      2.626     0.525     0.525
(15 HN  )   (15 HB# )      2.595     0.519     1.119 ! HB2 to HB# + 0.6 Å
(15 HN  )   (15 HG  )      3.003     0.601     0.601
```

7.10 XPLOR patches nle, lald and lale and peptide bond file top19_bicycle.pep

These patches were made in-house to describe the modified amino acids, the lactam bridge between residues P10 and P14 and the amide function at the C-terminal position.

```
residue NLE
! patch designed to create a norleucine residue JF Gaucher 2020
group
atom N   type=NH1 charge=-0.360 end
atom HN  type=H   charge= 0.260 end
group
atom CA  type=CT  charge= 0.000 end
atom HA  type=HA  charge= 0.100 end
group
atom CB  type=CT  charge=-0.200 end
atom HB1 type=HA  charge= 0.100 end
atom HB2 type=HA  charge= 0.100 end
group
atom CG  type=CT  charge=-0.200 end
atom HG1 type=HA  charge= 0.100 end
atom HG2 type=HA  charge= 0.100 end
group
atom CD  type=CT  charge=-0.200 end
atom HD1 type=HA  charge= 0.100 end
atom HD2 type=HA  charge= 0.100 end
group
atom CE  type=CT  charge=-0.200 end
atom HE1 type=HA  charge= 0.100 end
atom HE2 type=HA  charge= 0.100 end
group
atom CZ  type=CT  charge=-0.300 end
atom HZ1 type=HA  charge= 0.100 end
atom HZ2 type=HA  charge= 0.100 end
atom HZ3 type=HA  charge= 0.100 end
group
atom C   type=C   charge= 0.480 end
atom O   type=O   charge=-0.480 end

bond N  HN
bond N  CA     bond CA HA
bond CA CB     bond CB HB1     bond CB HB2
bond CB CG     bond CG HG1     bond CG HG2
bond CG CD     bond CD HD1     bond CD HD2
bond CD CE     bond CE HE1     bond CE HE2
bond CE CZ     bond CZ HZ1     bond CZ HZ2     bond CZ HZ3
bond CA C
bond C  O

improper HA N C CB      !stereo CA
improper HB1 HB2 CA CG  !stereo CB
improper HG1 HG2 CB CD  !stereo CG
improper HD1 HD2 CG CE  !stereo CD
improper HE1 HE2 CD CZ  !stereo CE
improper HZ1 HZ2 CE HZ3 !stereo CZ

dihedral CG  CB  CA  N
dihedral CD  CG  CB  CA
dihedral CE  CD  CG  CB
dihedral CZ  CE  CD  CG

end

presidue LALD
! patch designed to create a lactam bridge between the NZ of Lysine in position 1 and
! CD of Aspartate in position 2. Importantly lactam bridge conformation is trans.
! topology and parameters derive from peptide link template
! residue 1 refers to Lys and residue 2 to ASP - JF Gaucher 2020
group
modify atom 1CE           charge=-0.10  END
```

```
modify atom 1NZ  type=NH1 charge=-0.36  END
modify atom 1HZ1 type=H   charge= 0.26  END
delete atom 1HZ2                        END
delete atom 1HZ3                        END
group
modify atom 2CB           charge=-0.20  END
modify atom 2CG  type=C   charge= 0.48  END
modify atom 2OD1 type=O   charge=-0.48  END
delete atom 2OD2                        END

add bond 1NZ 2CG

add angle 2CB 2CG 1NZ                     ! OK
add angle 1NZ 2CG 2OD1                    ! OK
add angle 2CG 1NZ 1CE                     ! OK
add angle 1HZ1 1NZ 2CG                    ! OK

add improper 2OD1 2CG 1NZ 1CE             ! planar -C    fixed for DG by JK
add improper 1HZ1 1NZ 2CG 2CB             ! planar +N
add improper 2CB 2CG 1NZ 1CE              ! planar peptide        "
end

presidue LALE
! patch designed to create a lactam bridge between the NZ of Lysine in position 1 and
! CD of Glutamate in position 2. Importantly lactam bridge conformation is trans.
! topology and parameters derive from peptide link template
! residue 1 refers to Lys and residue 2 to GLU - JF Gaucher 2020

group
modify atom 1CE           charge=-0.10  END
modify atom 1NZ  type=NH1 charge=-0.36  END
modify atom 1HZ1 type=H   charge= 0.26  END
delete atom 1HZ2                        END
delete atom 1HZ3                        END
group
modify atom 2CG           charge=-0.20  END
modify atom 2CD  type=C   charge= 0.48  END
modify atom 2OE1 type=O   charge=-0.48  END
delete atom 2OE2                        END

add bond 1NZ 2CD

add angle 2CG 2CD 1NZ
add angle 1NZ 2CD 2OE1
add angle 2CD 1NZ 1CE
add angle 1HZ1 1NZ 2CD

add improper 2OE1 2CD 1NZ 1CE                     ! planar -C    fixed for DG by JK
add improper 1HZ1 1NZ 2CD 2CG                     ! planar +N
add improper 2CG 2CD 1NZ 1CE                   ! planar peptide        "
end
```

7.11 NMR spectra

7.11.1 Peptide **1**

CDIHV^{n}LWEWKCFEDL-NH$_2$
SS

^{1}H 400 MHz

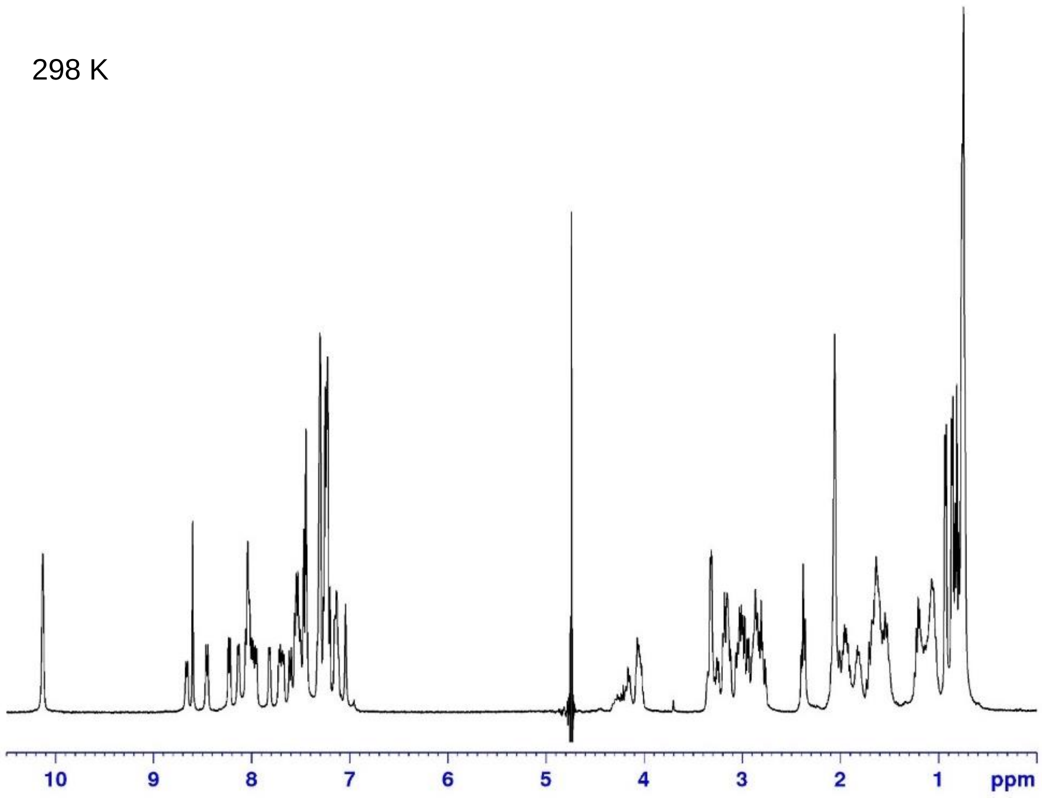

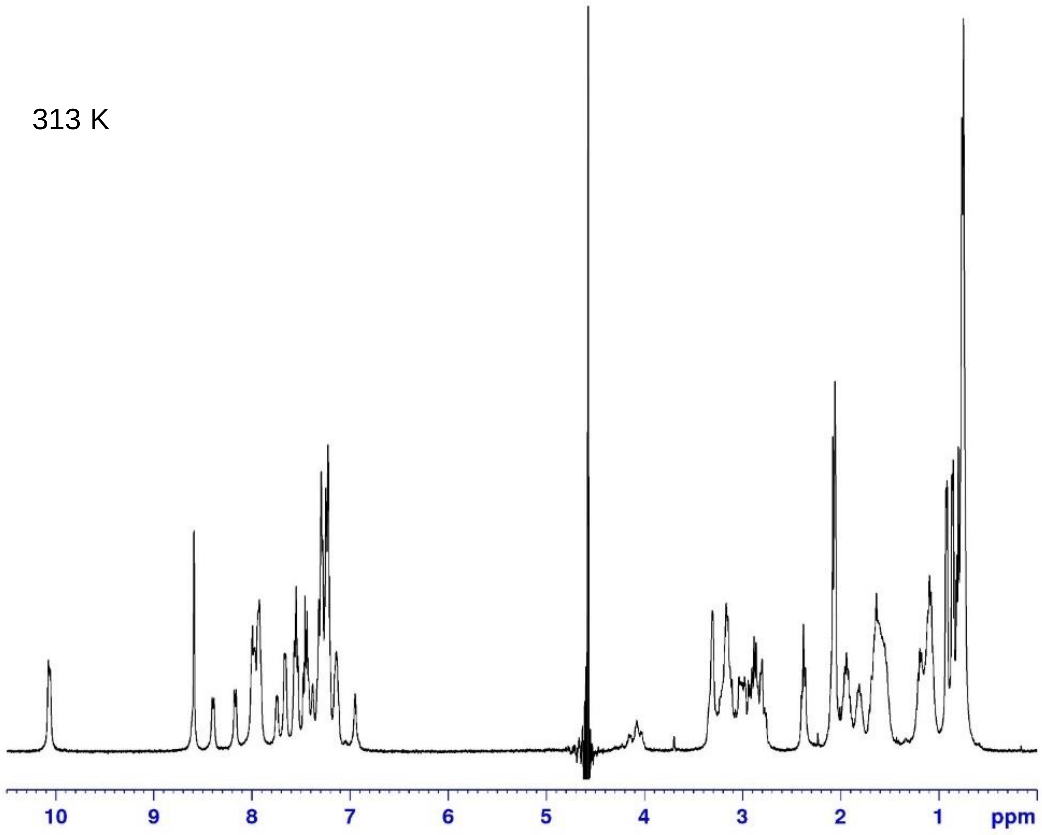

Peptide **1** – TOCSY 400 MHz

298 K

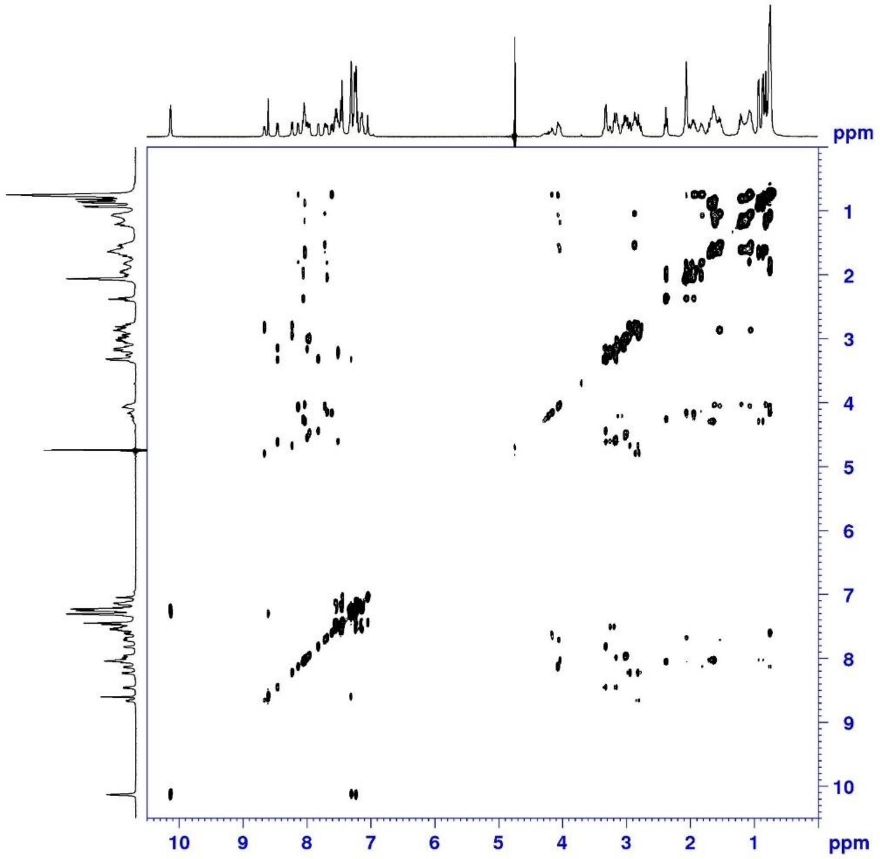

313K

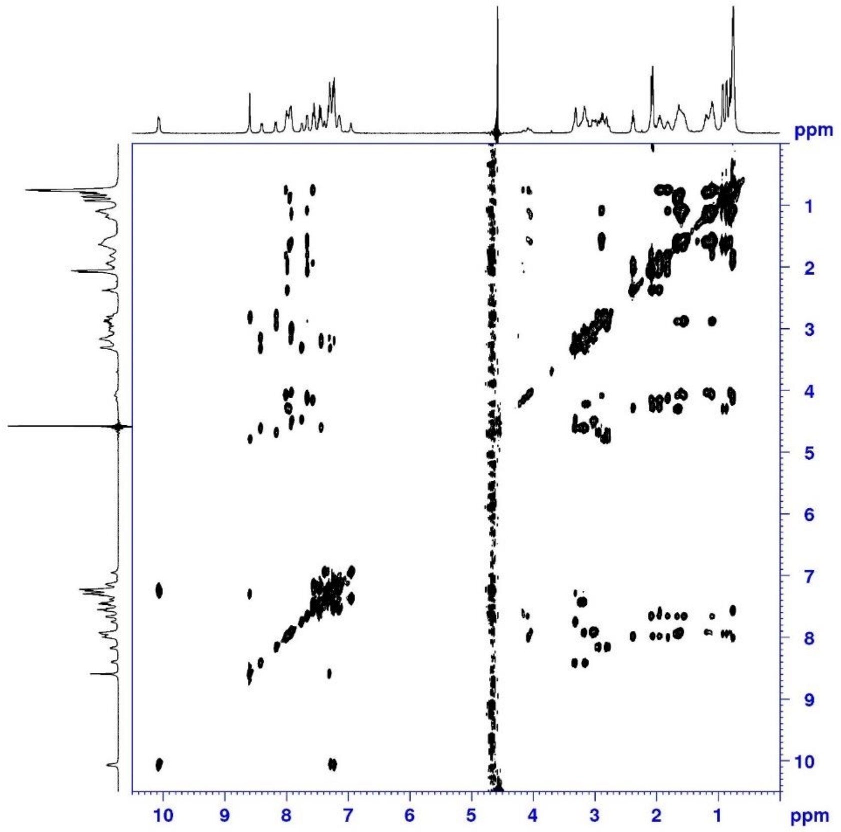

Peptide **1** – TOCSY 400 MHz

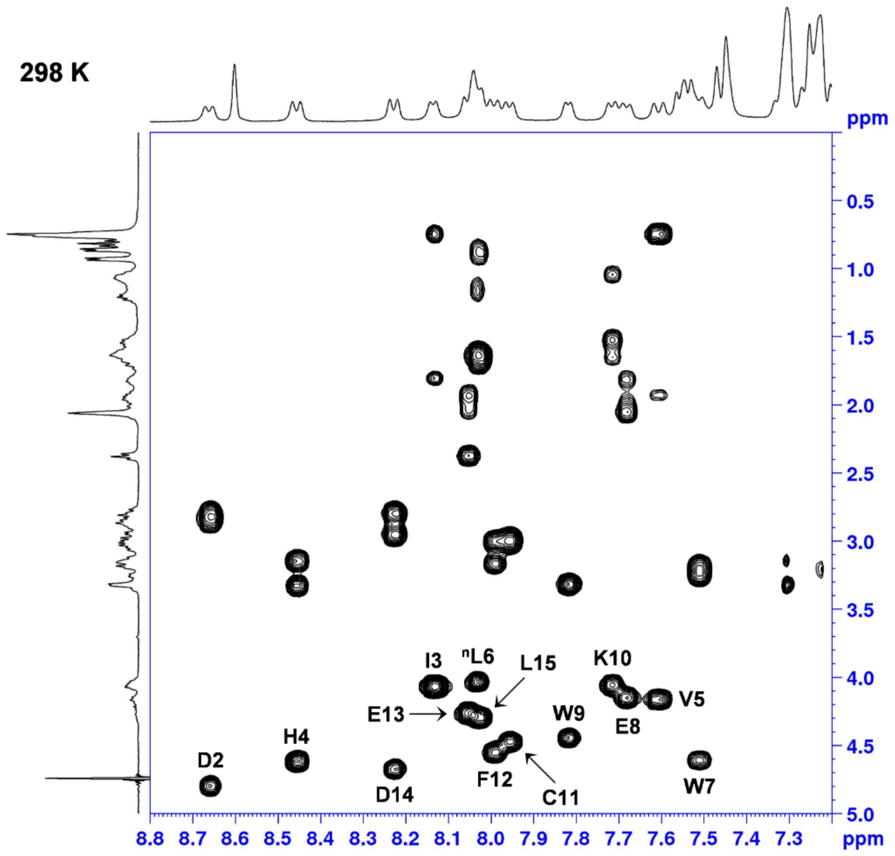

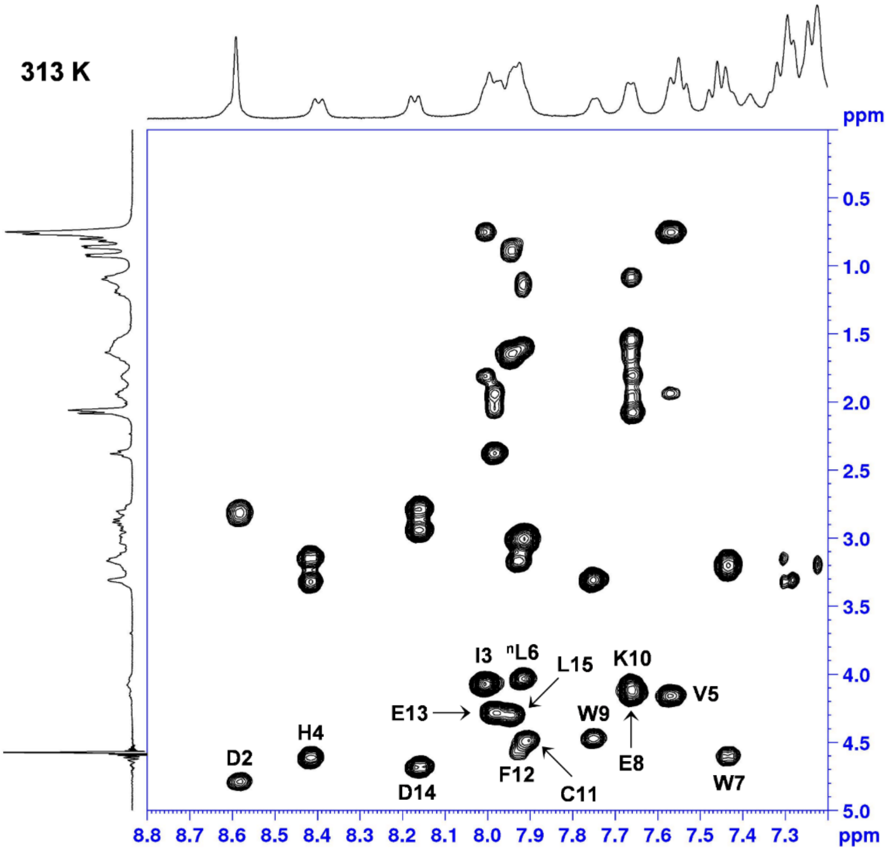

Peptide **1** - NOESY 400 MHz

298 K

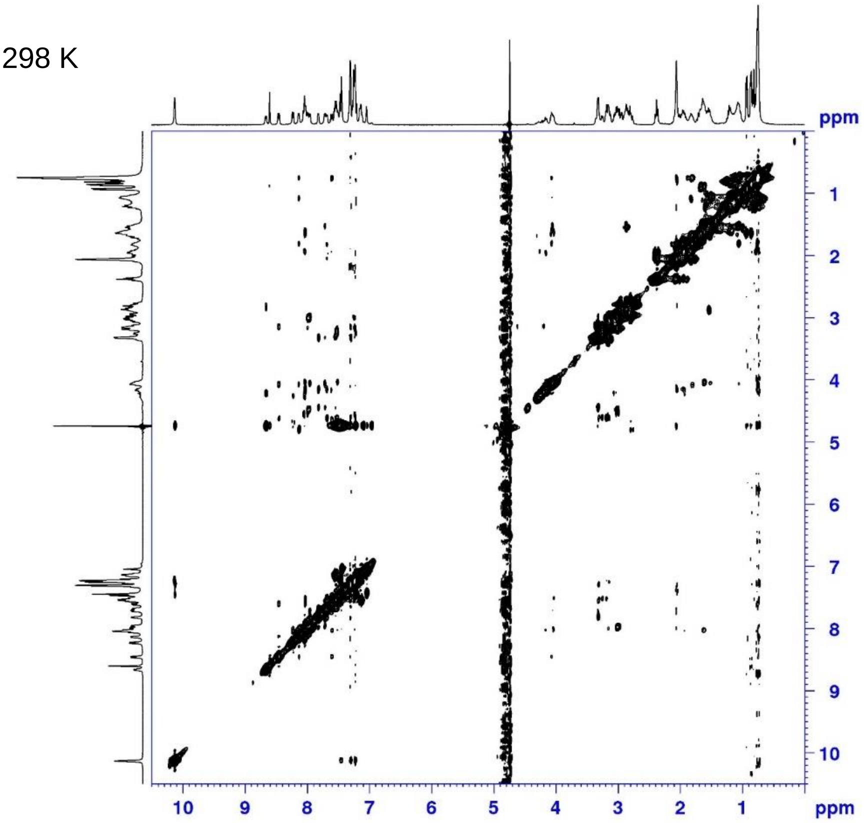

313K

313 K

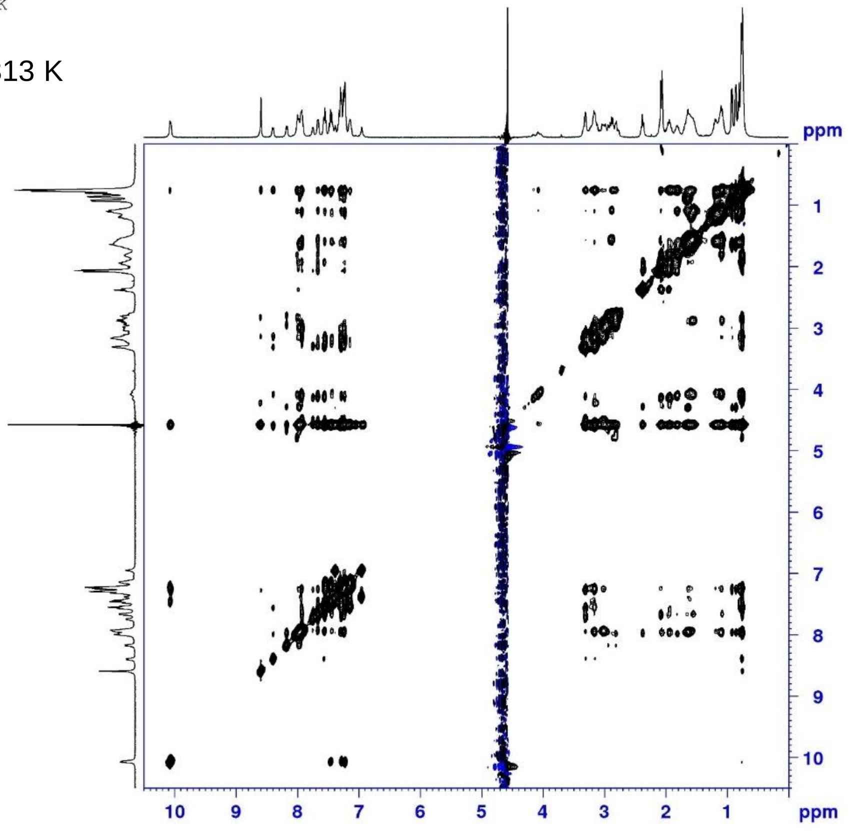

7.11.2 Peptide **1c**

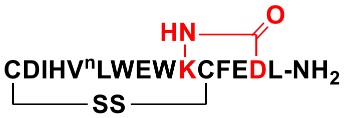

^{1}H 400 MHz

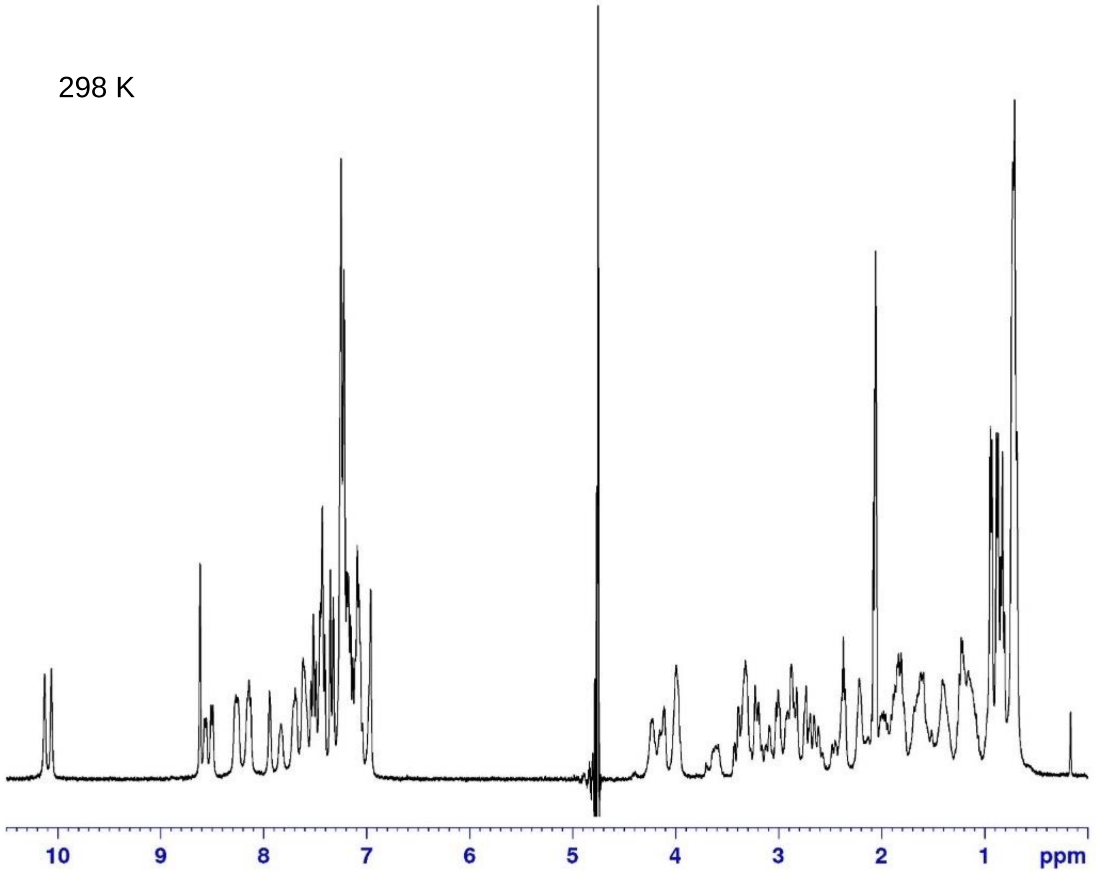

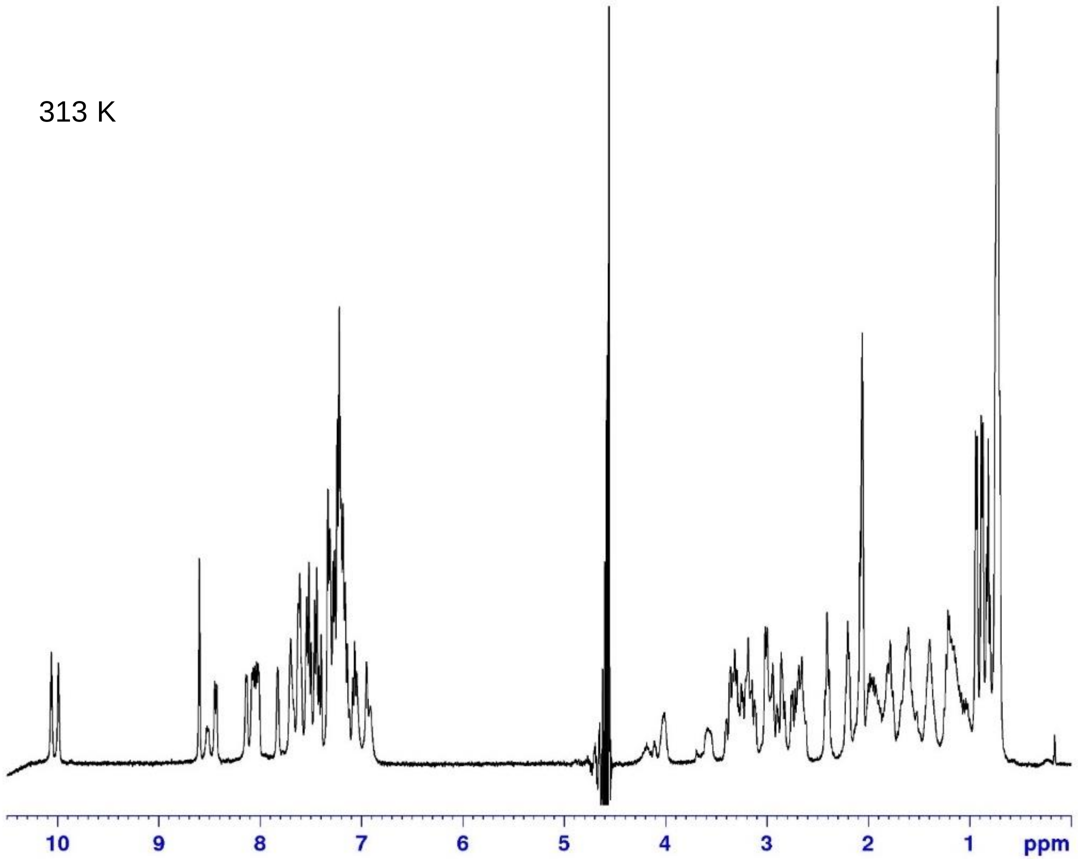

Peptide **1c** TOCSY 400 MHz

298K

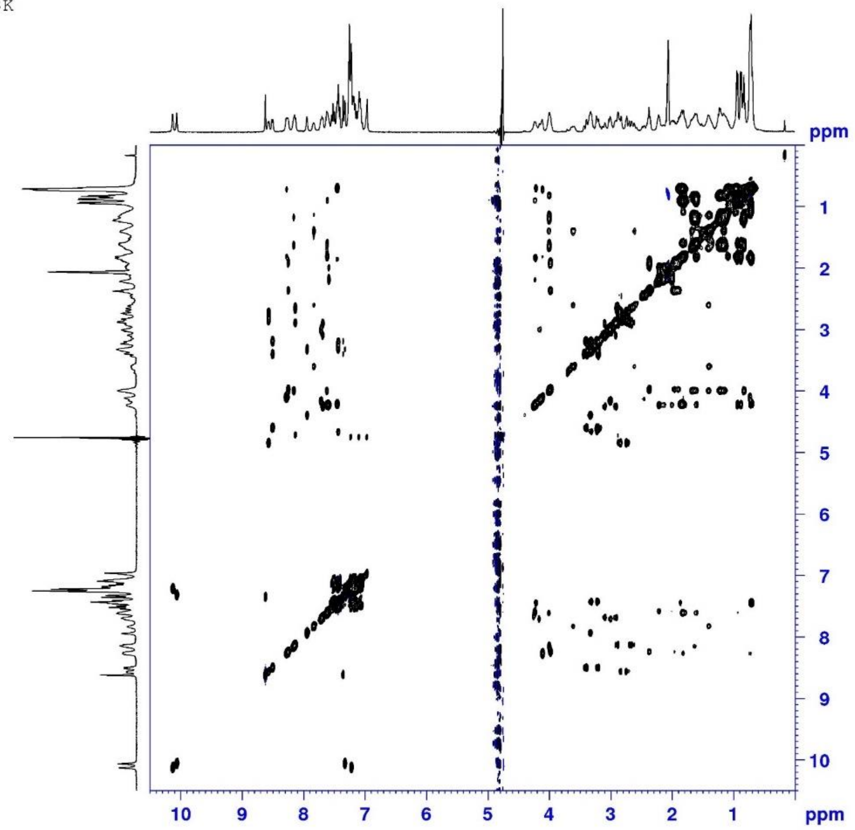

313K

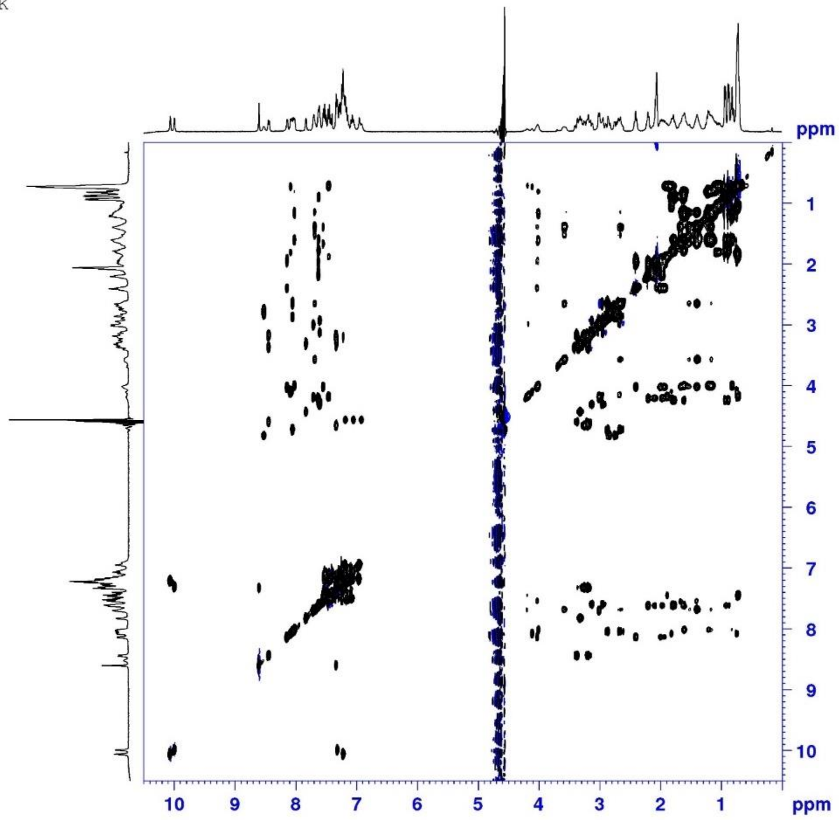

Peptide **1c** - TOCSY 400 MHz

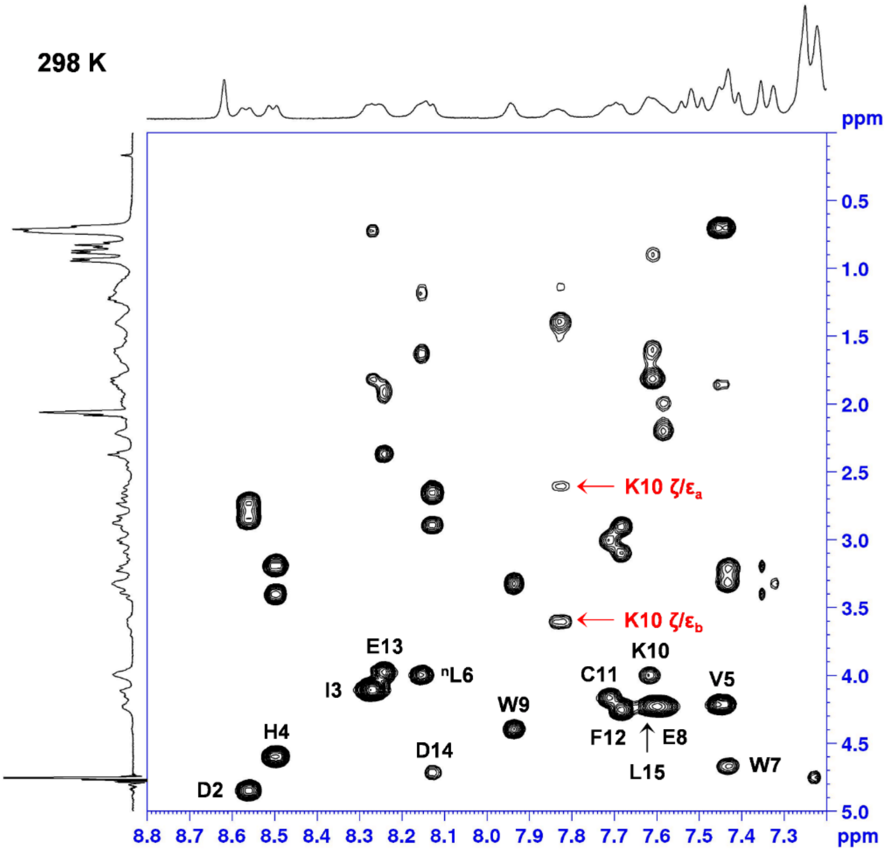

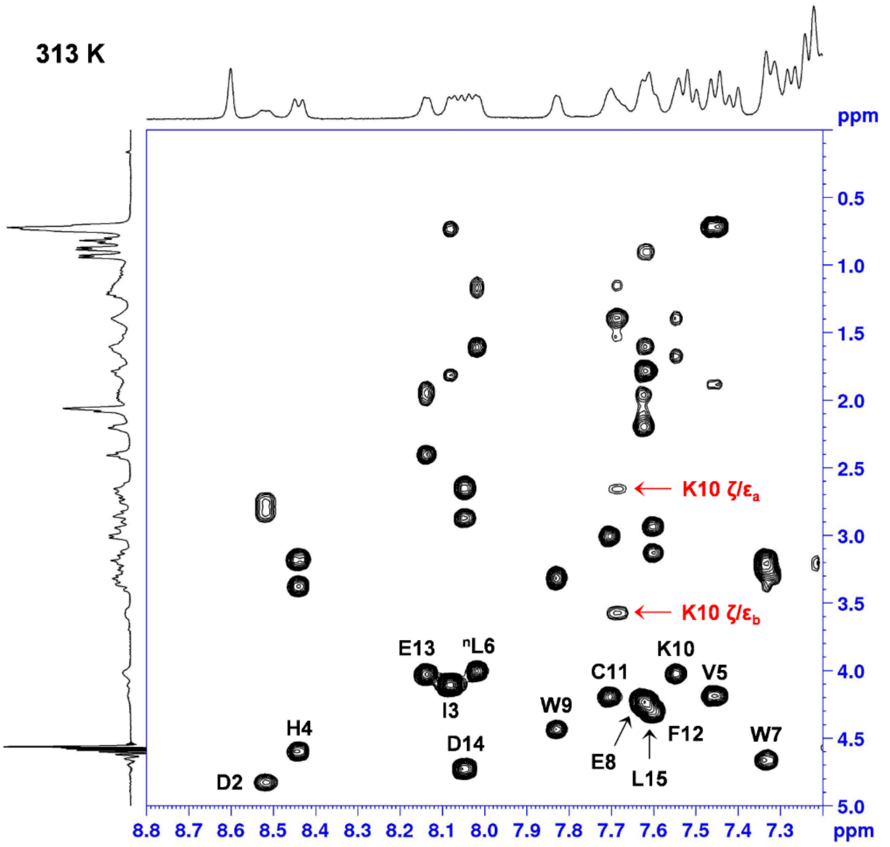

Peptide **1c** - NOESY 400 MHz

298 K

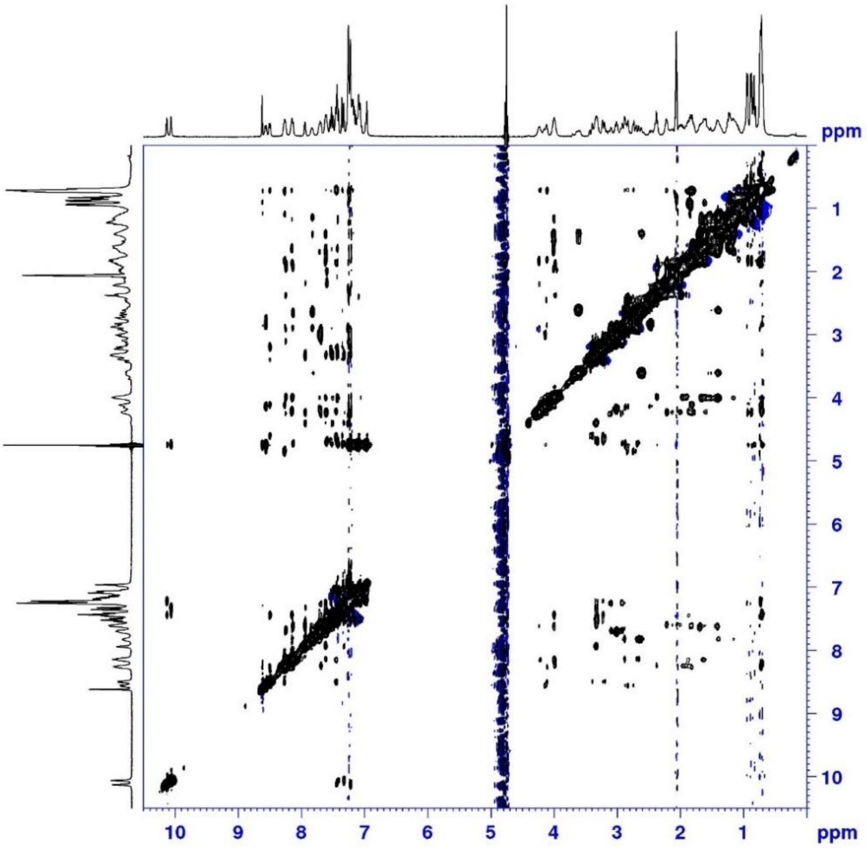

313 K

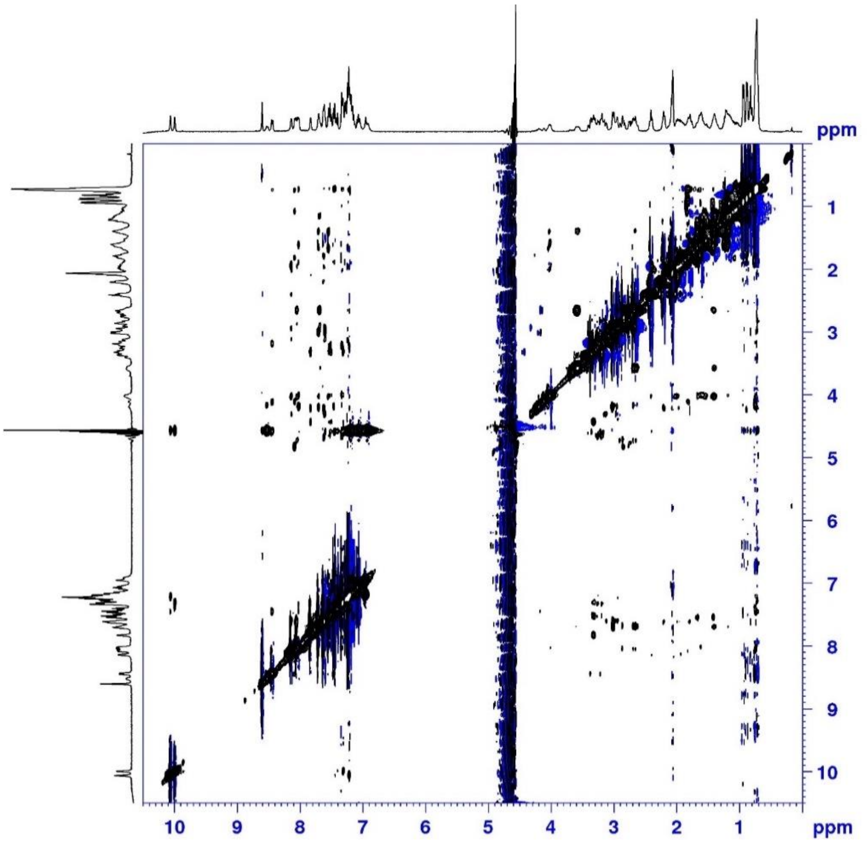

7.11.3 Peptide **2**

CDIHV^{n}LWEWDCFEKL-NH$_2$ (SS bridge between the two C residues)

^{1}H 600 MHz

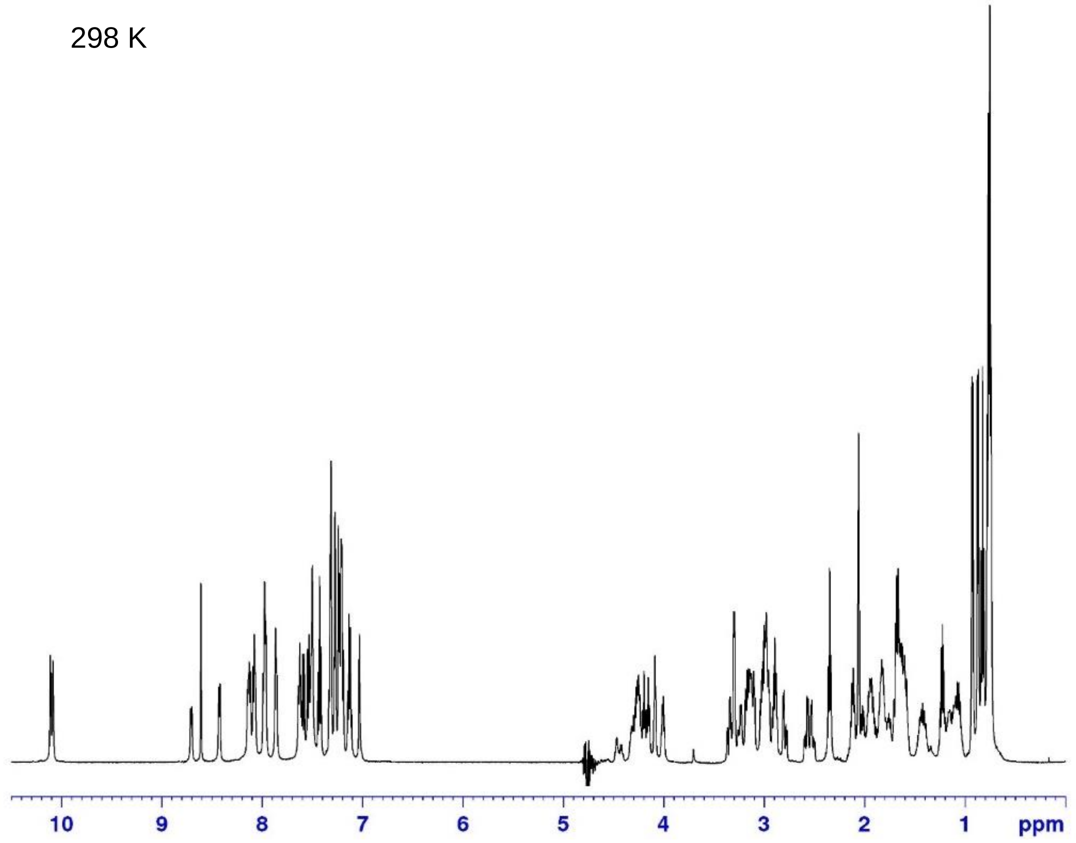

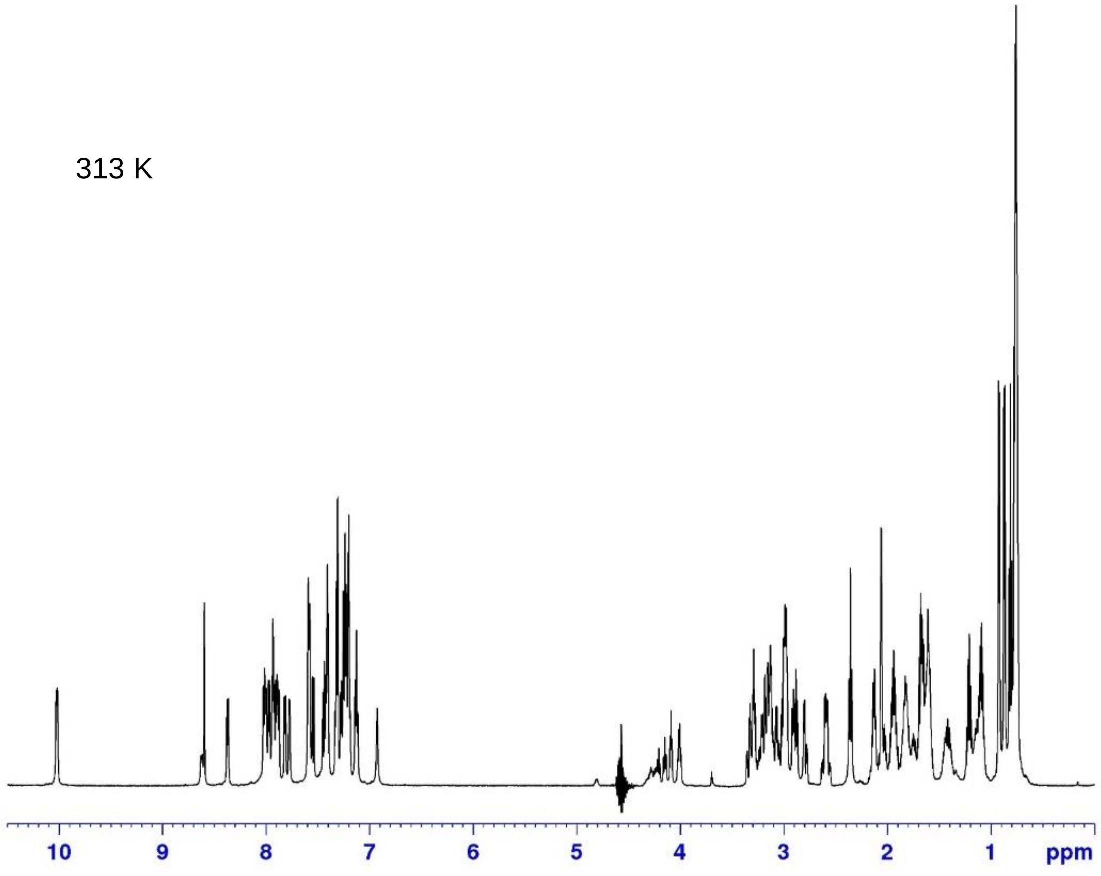

Peptide **2** TOCSY 600 MHz

SB-V-24 298K 2.3mg/500ul H20/D20/CD3CN 80/10/10 tocsyesgp

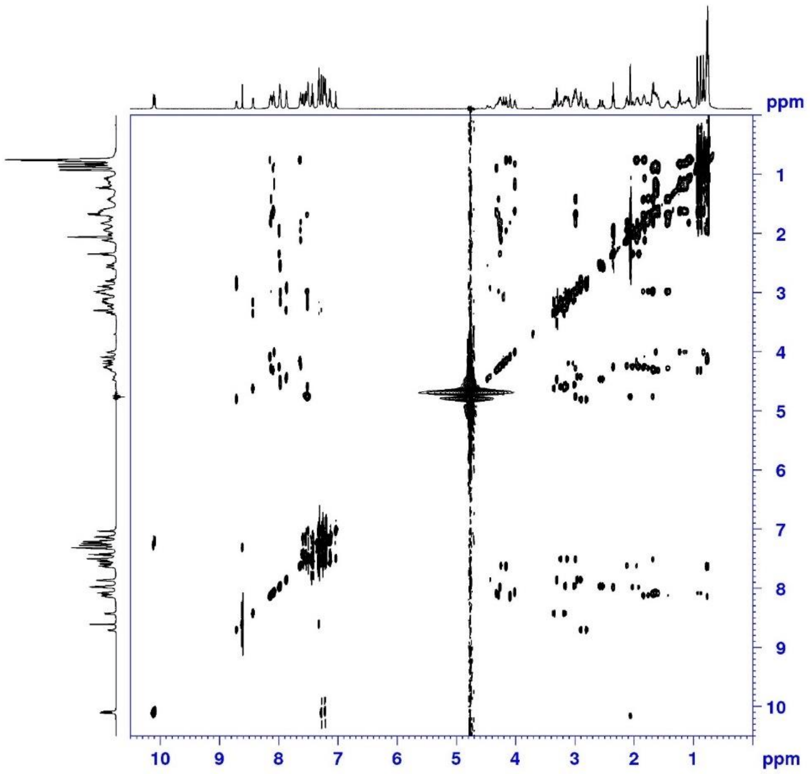

SB-V-24 313K 2.3mg/500ul H20/D20/CD3CN 80/10/10 tocsyesgp

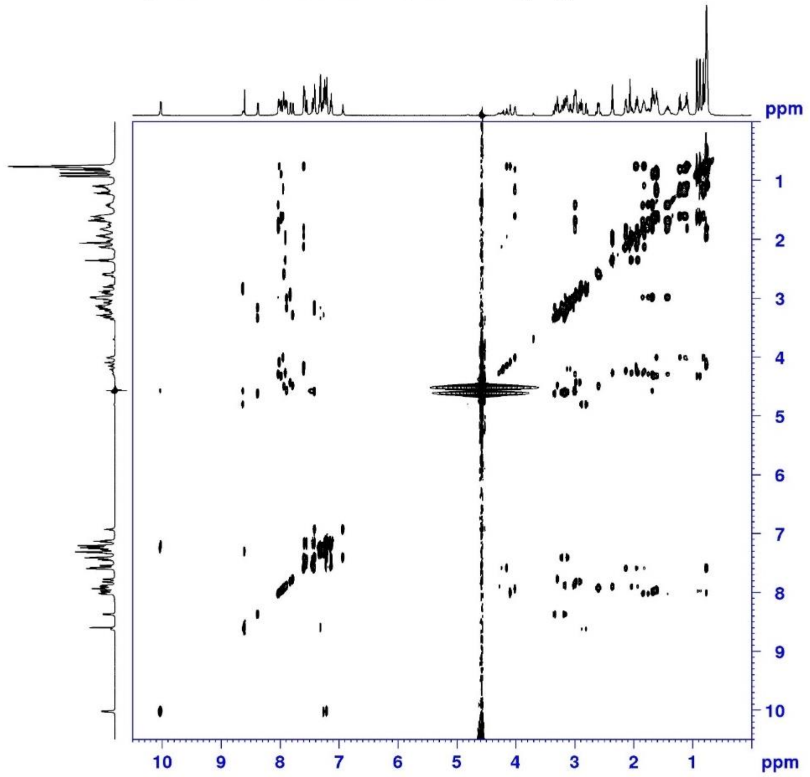

Peptide **2** - TOCSY 600 MHz

298 K

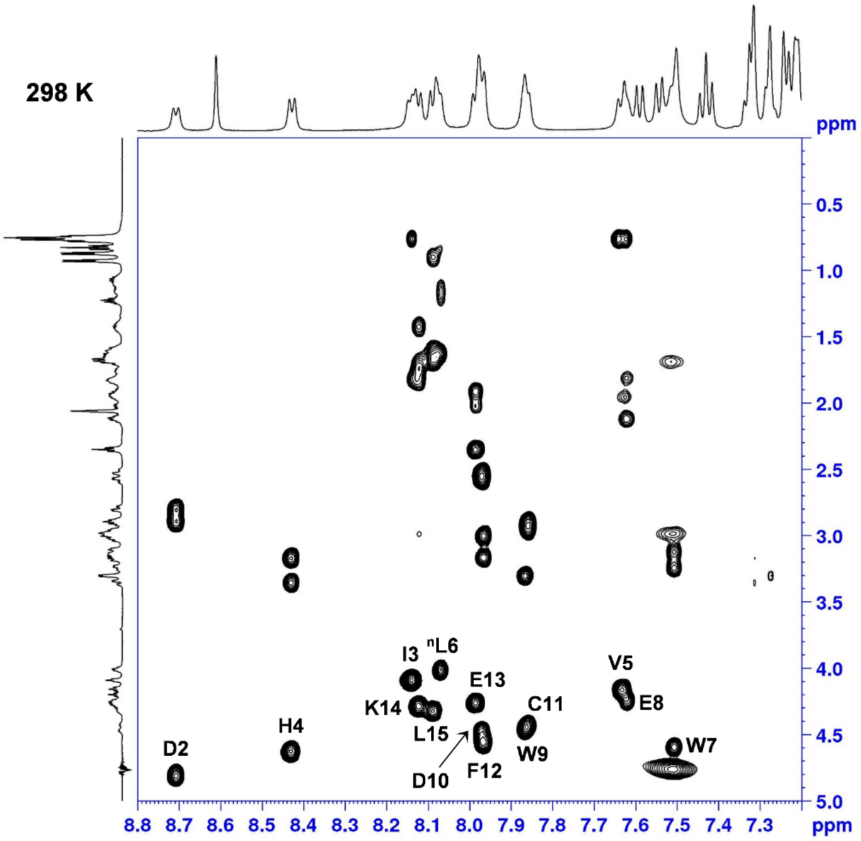

313 K

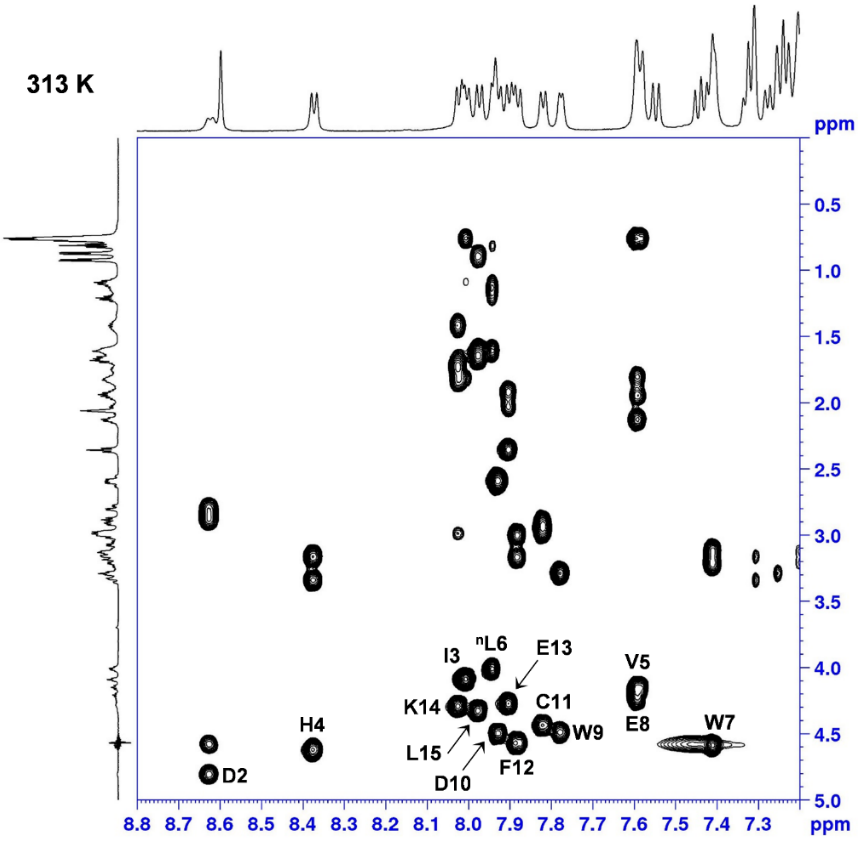

Peptide **2** NOESY 600 MHz

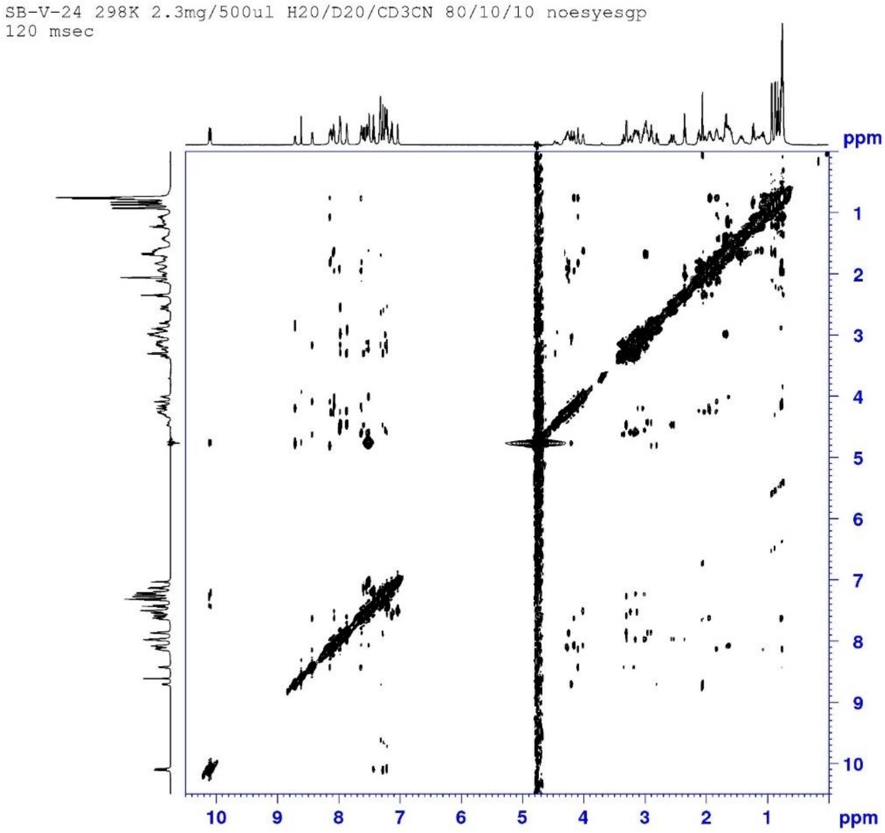

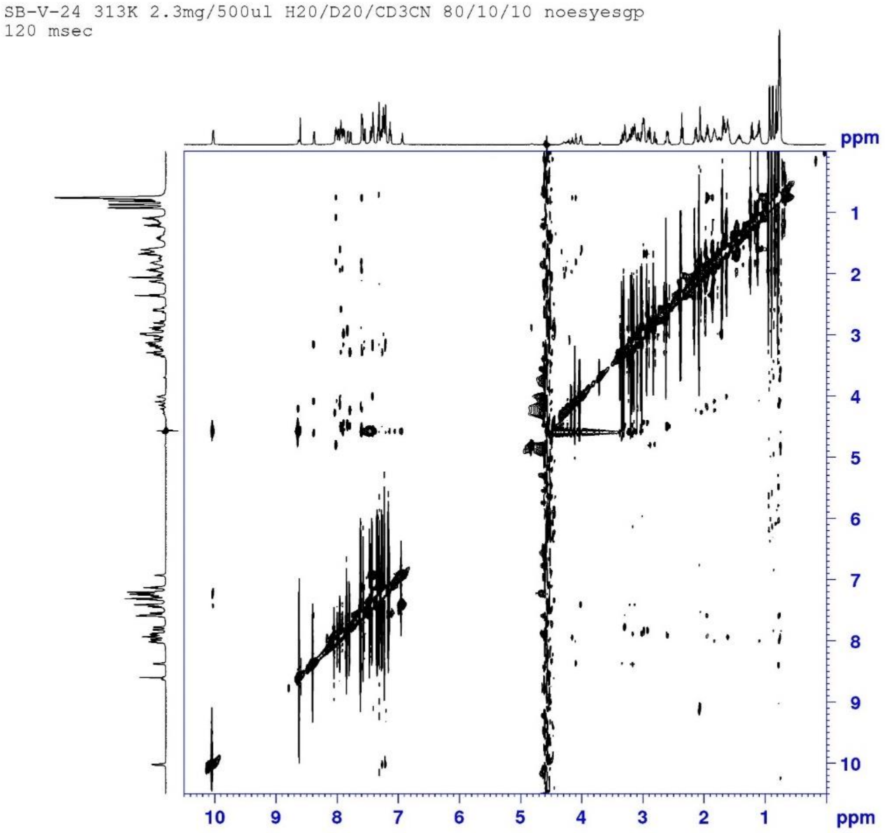

7.11.4 Peptide **2c**

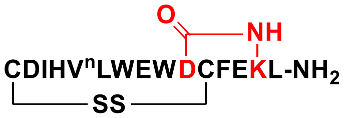

^{1}H 600 MHz

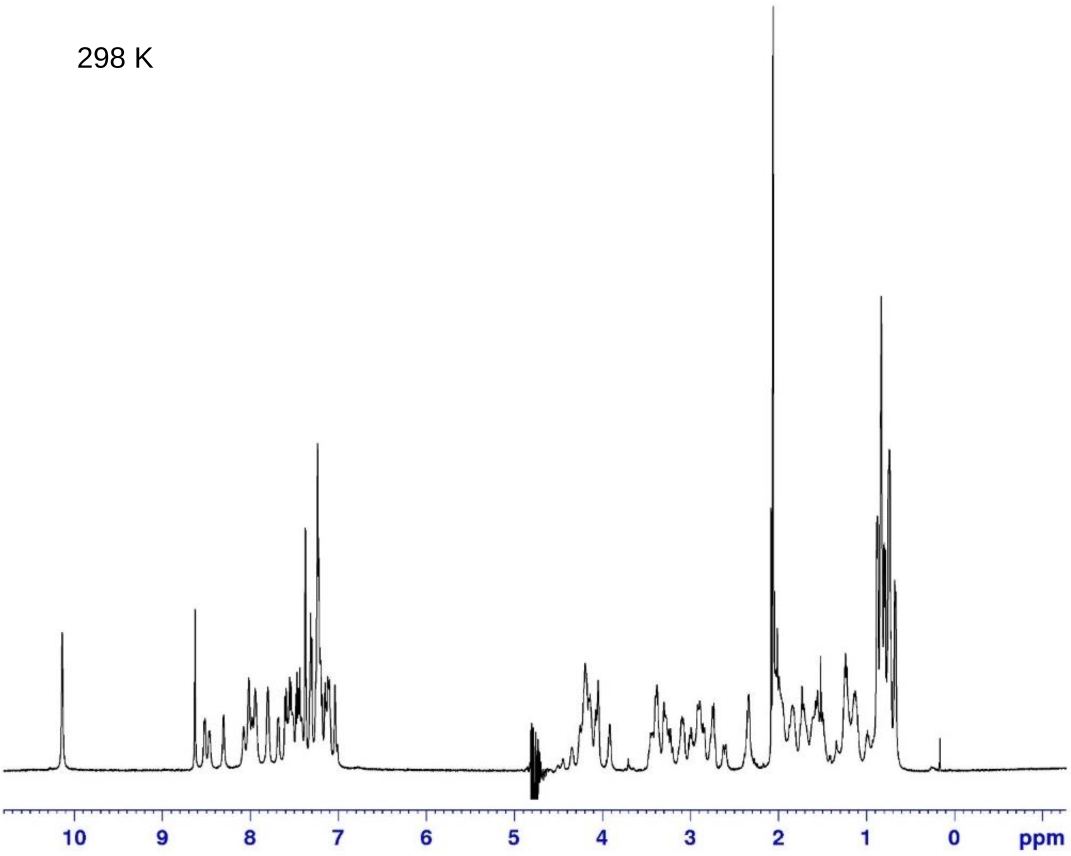

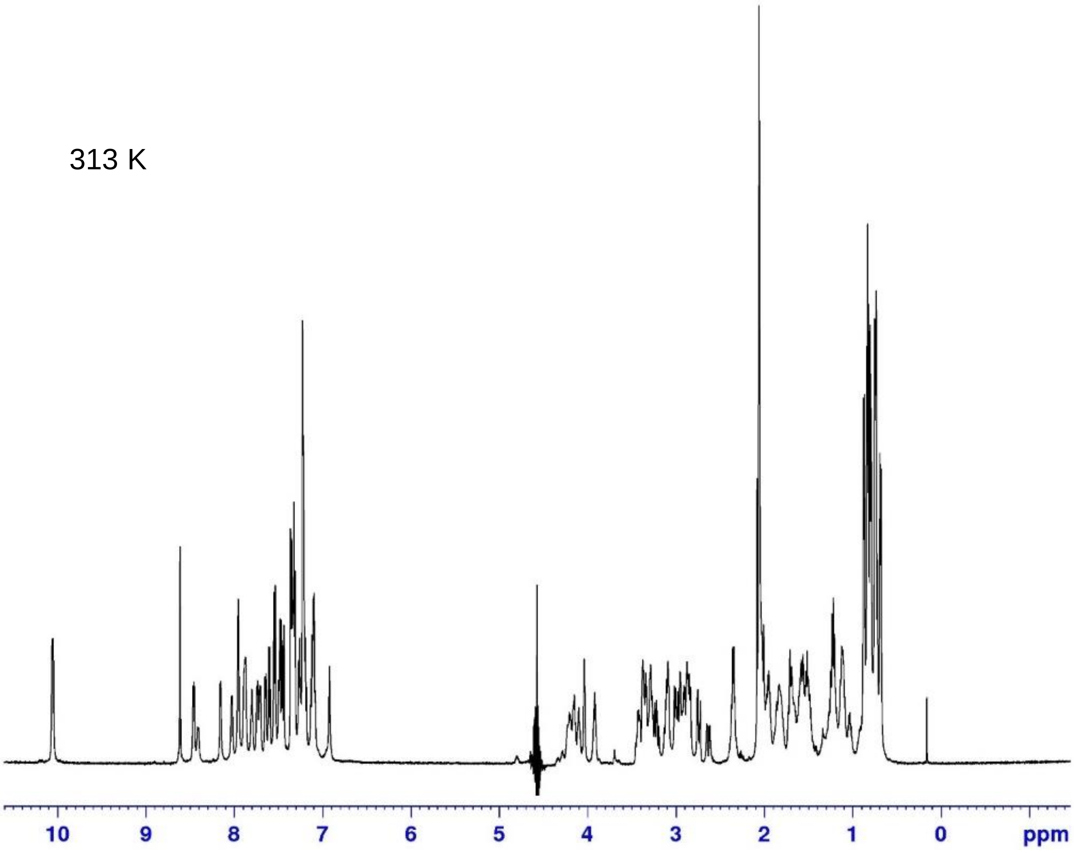

Peptide **2c** TOCSY 600 MHz

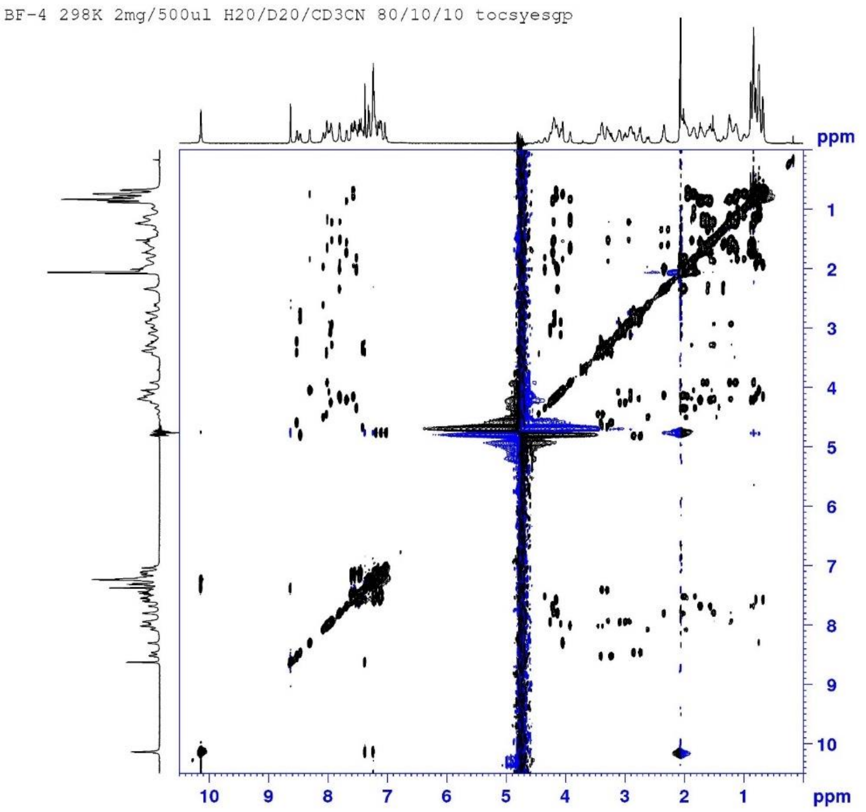

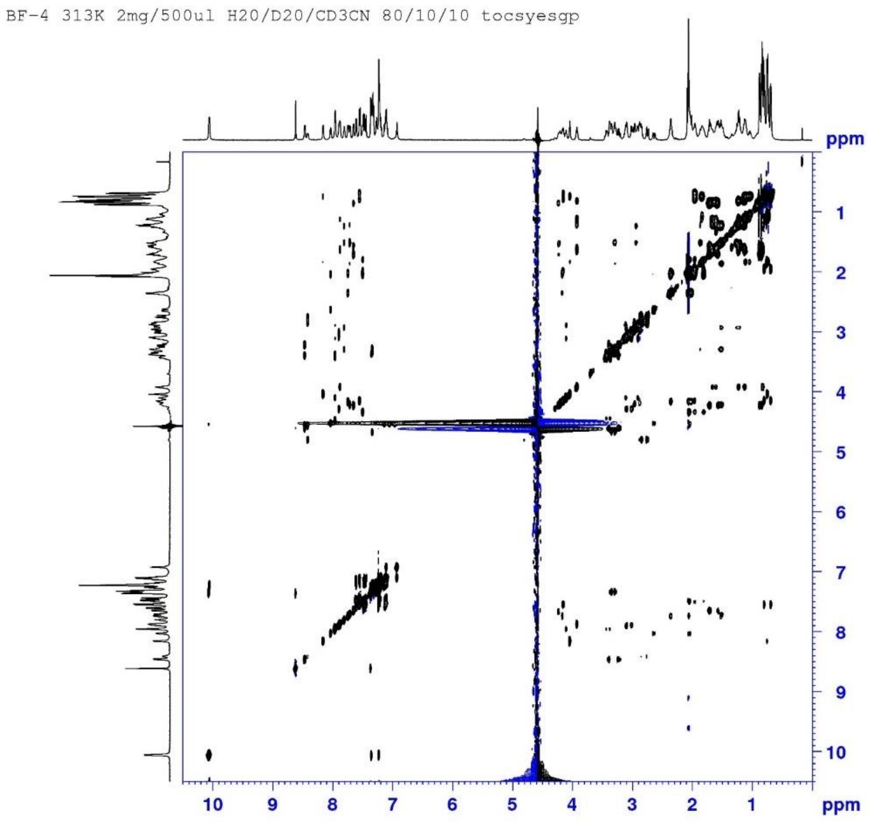

Peptide **2c** - TOCSY 600 MHz

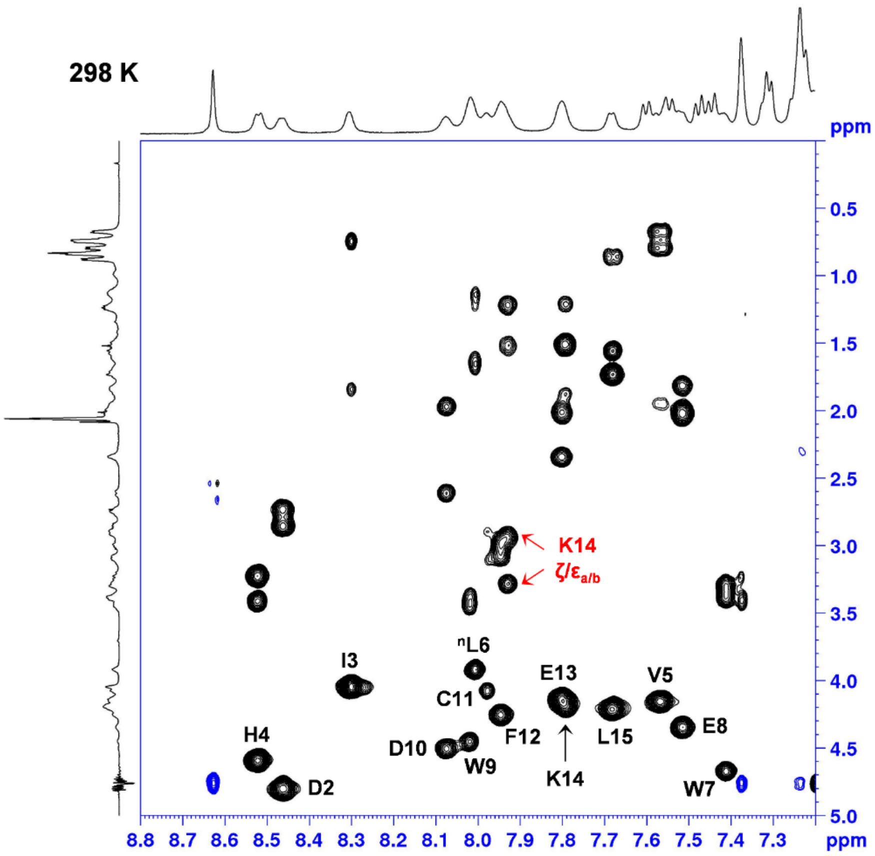

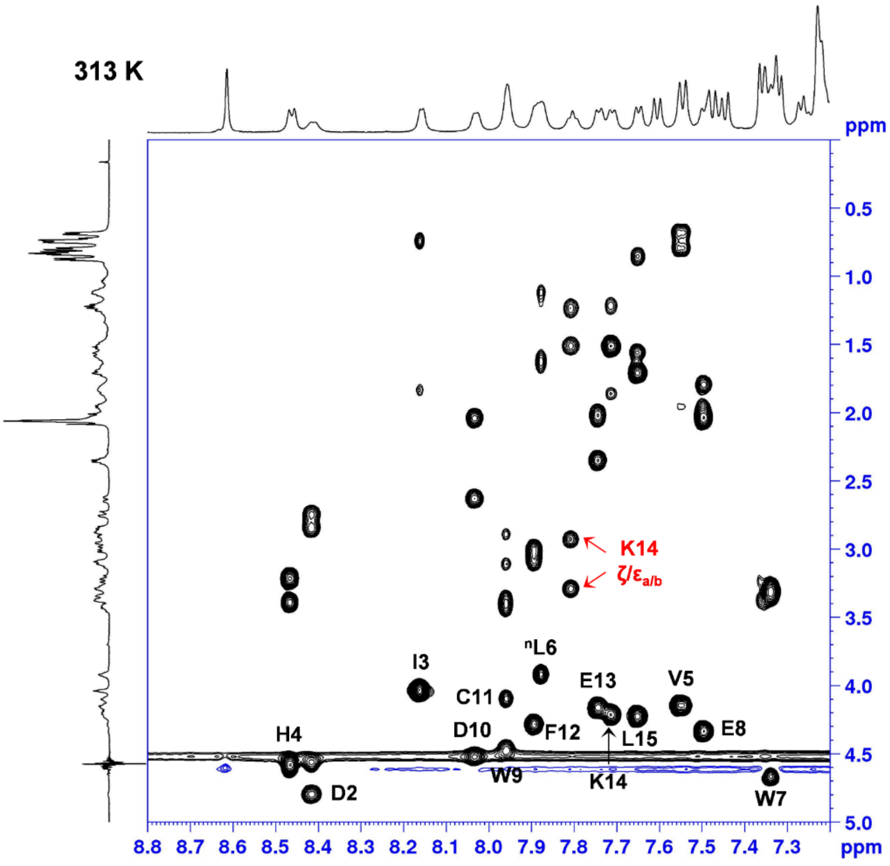

Peptide **2c** NOESY 600 MHz

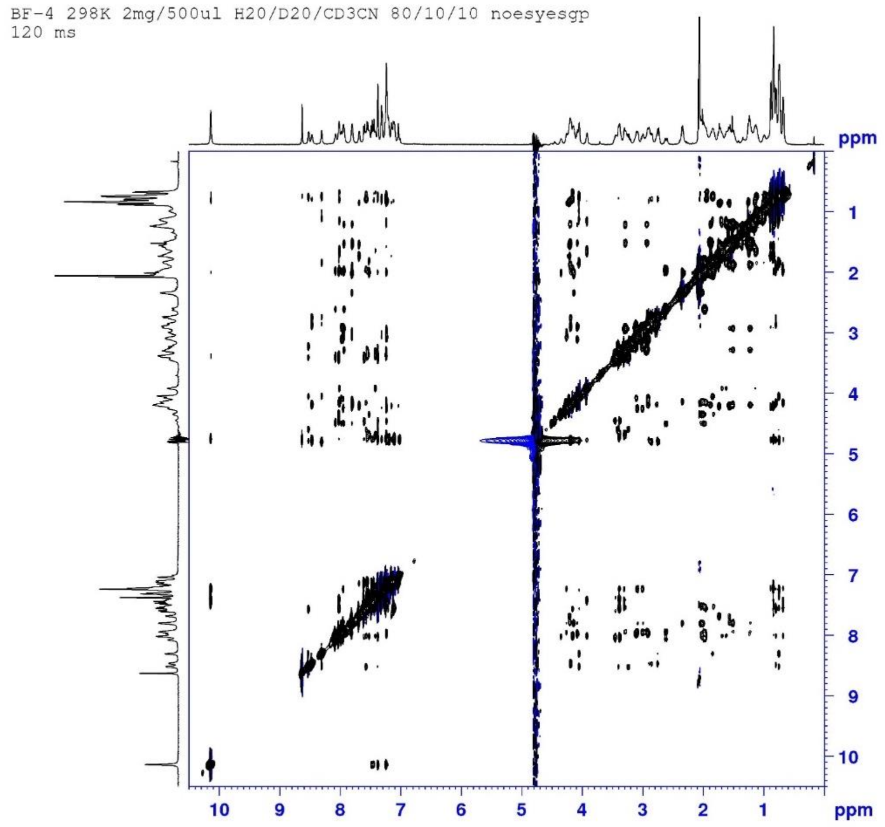

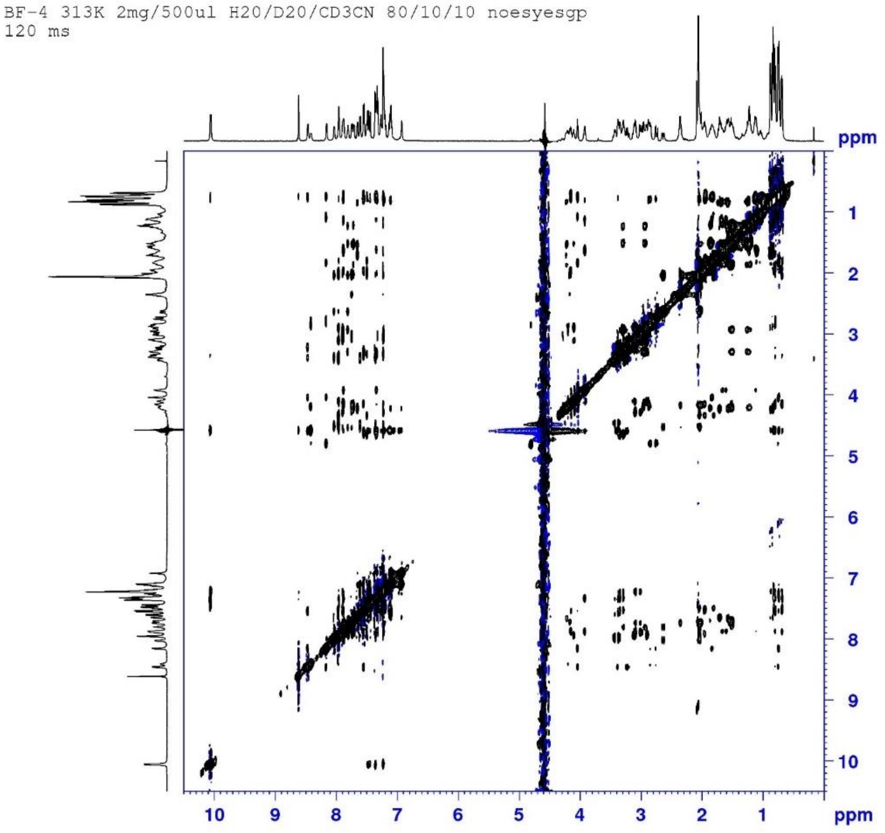

7.11.5 Peptide **3**

CDIHV^{n}LWEWECFEKL-NH$_2$

SS

^{1}H 600 MHz

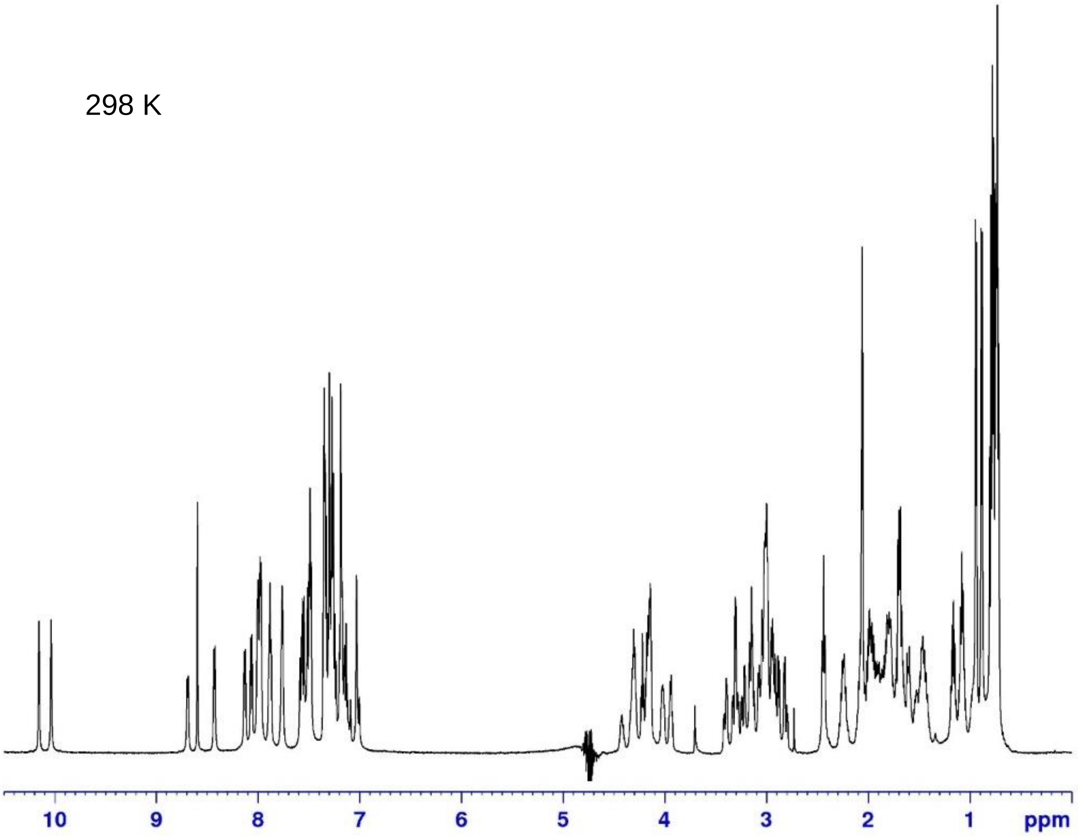

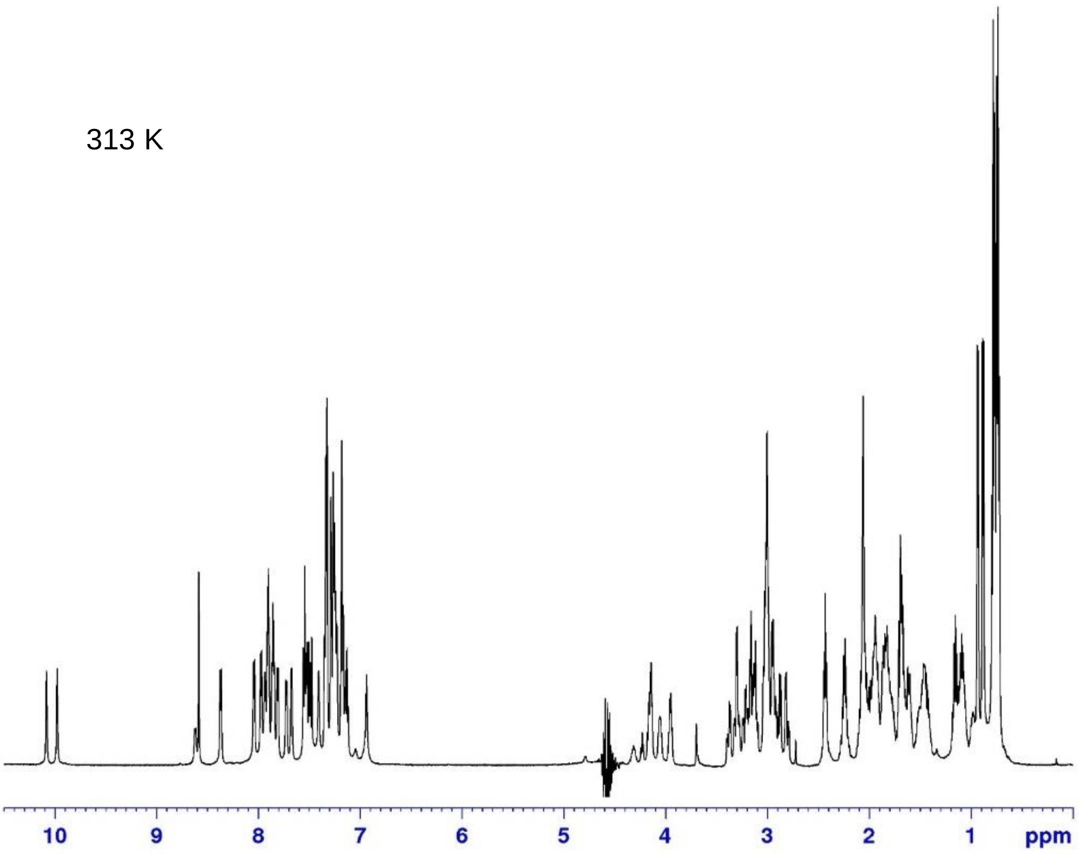

Peptide **3** TOCSY 600 MHz

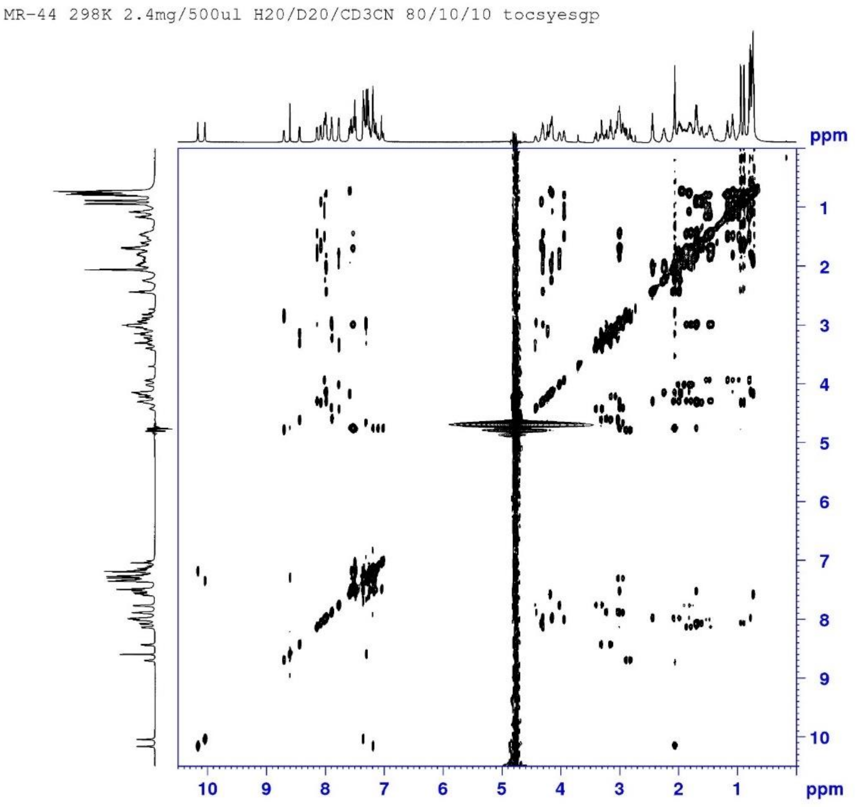

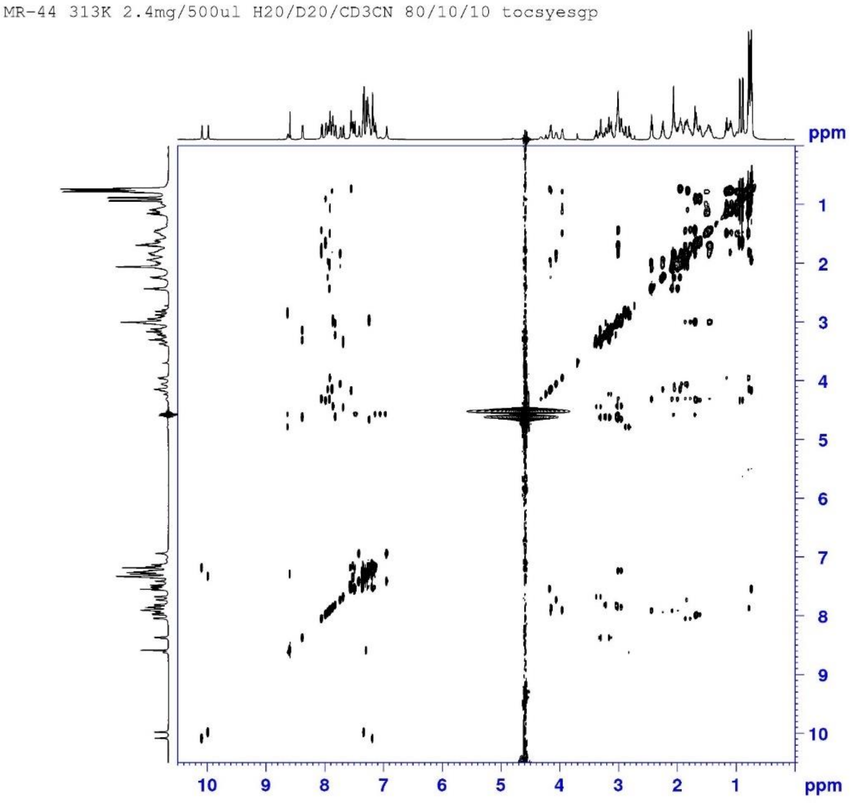

Peptide **3** - TOCSY 600 MHz

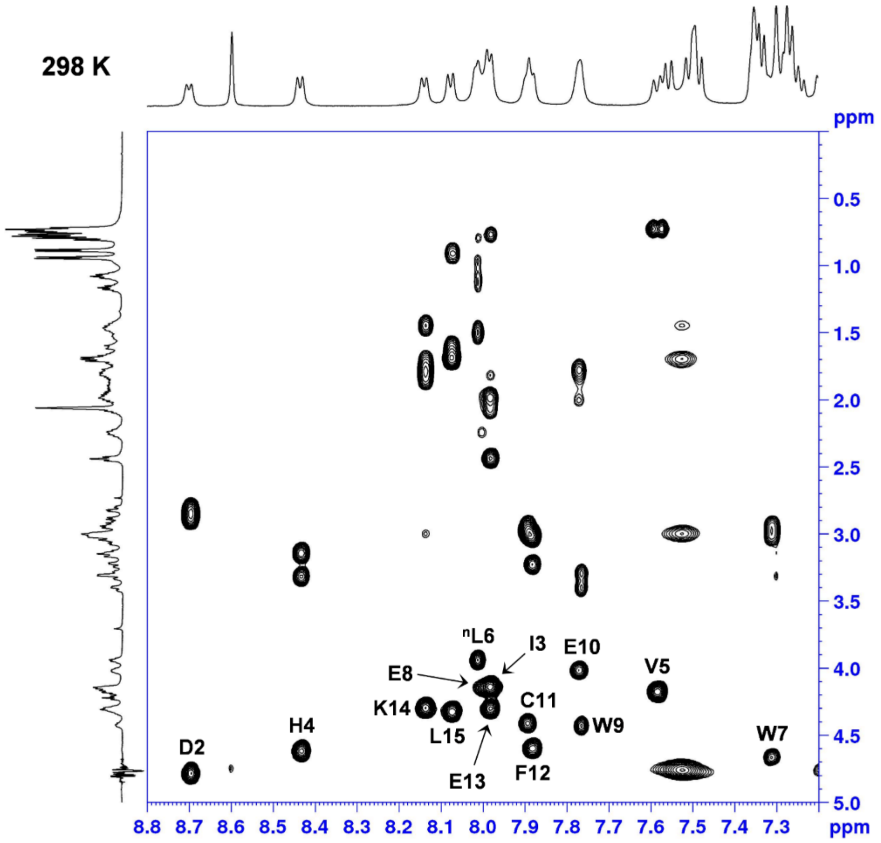

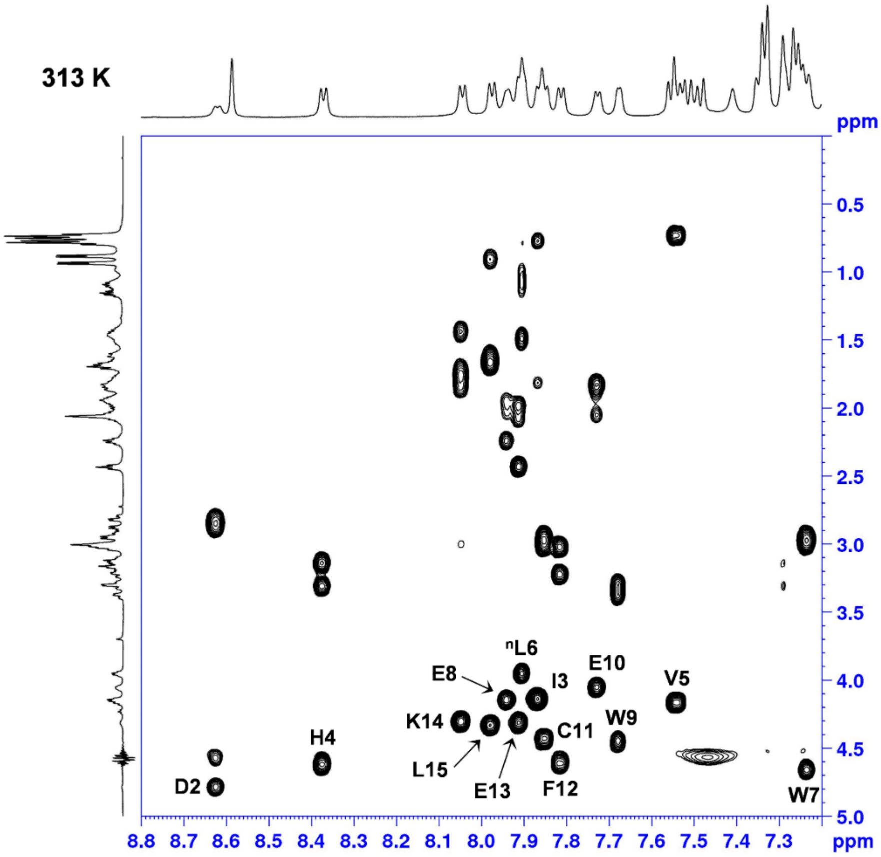

Peptide **3** NOESY 600 MHz

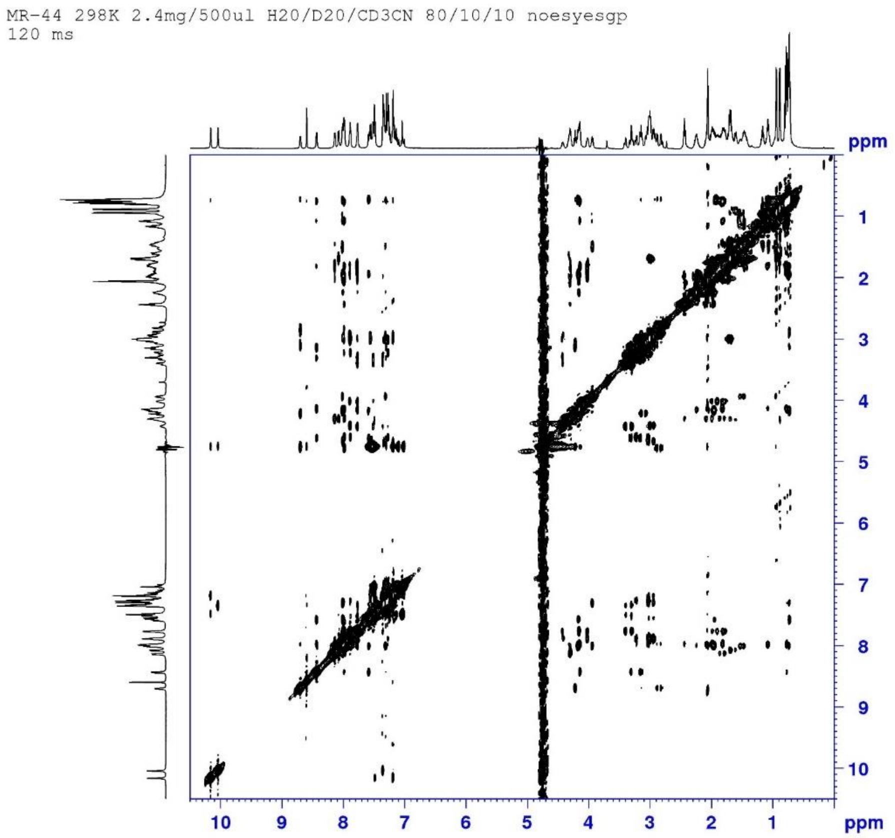

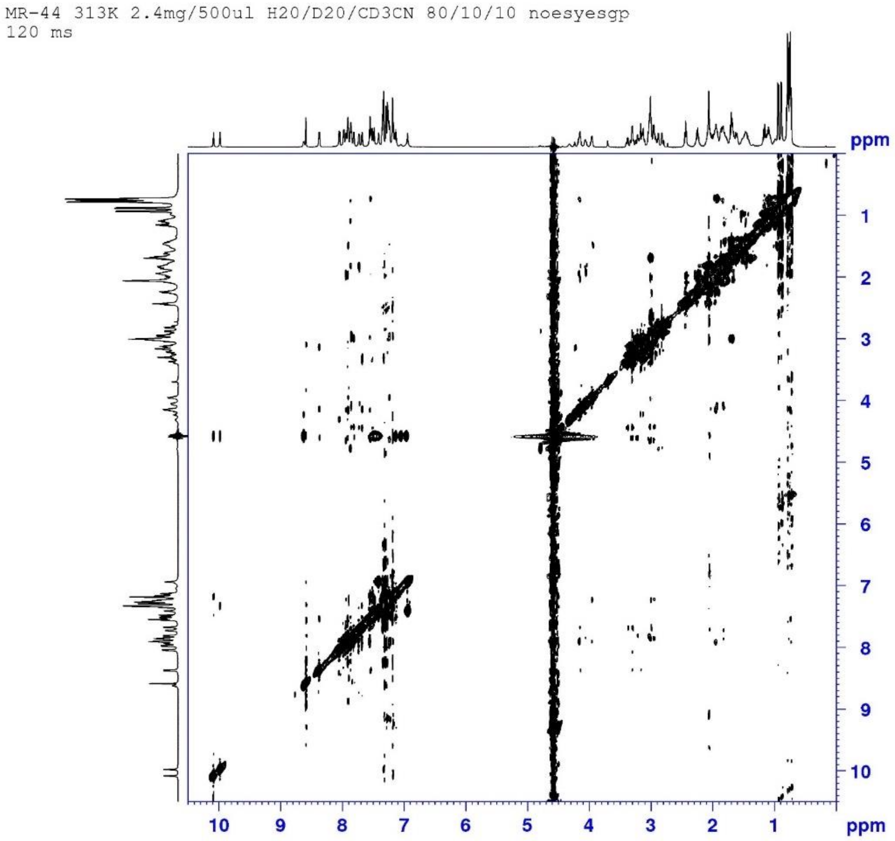

7.11.6 Peptide **3c**

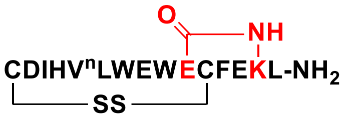

^{1}H 600 MHz

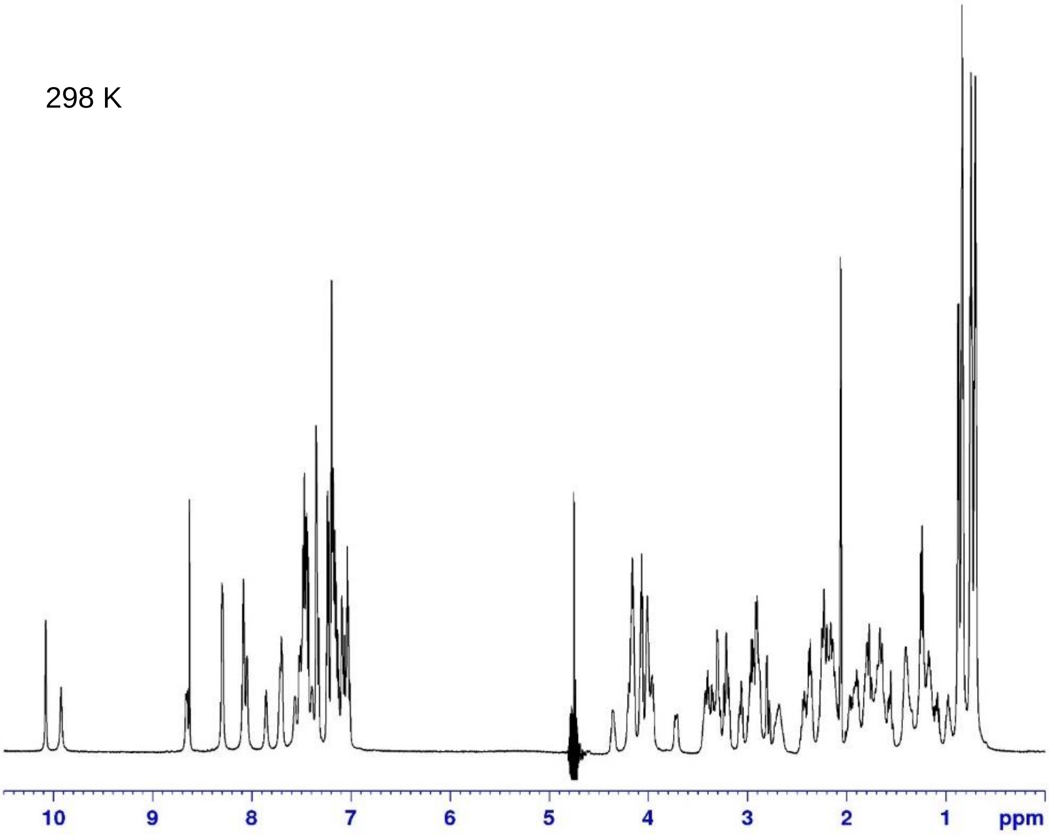

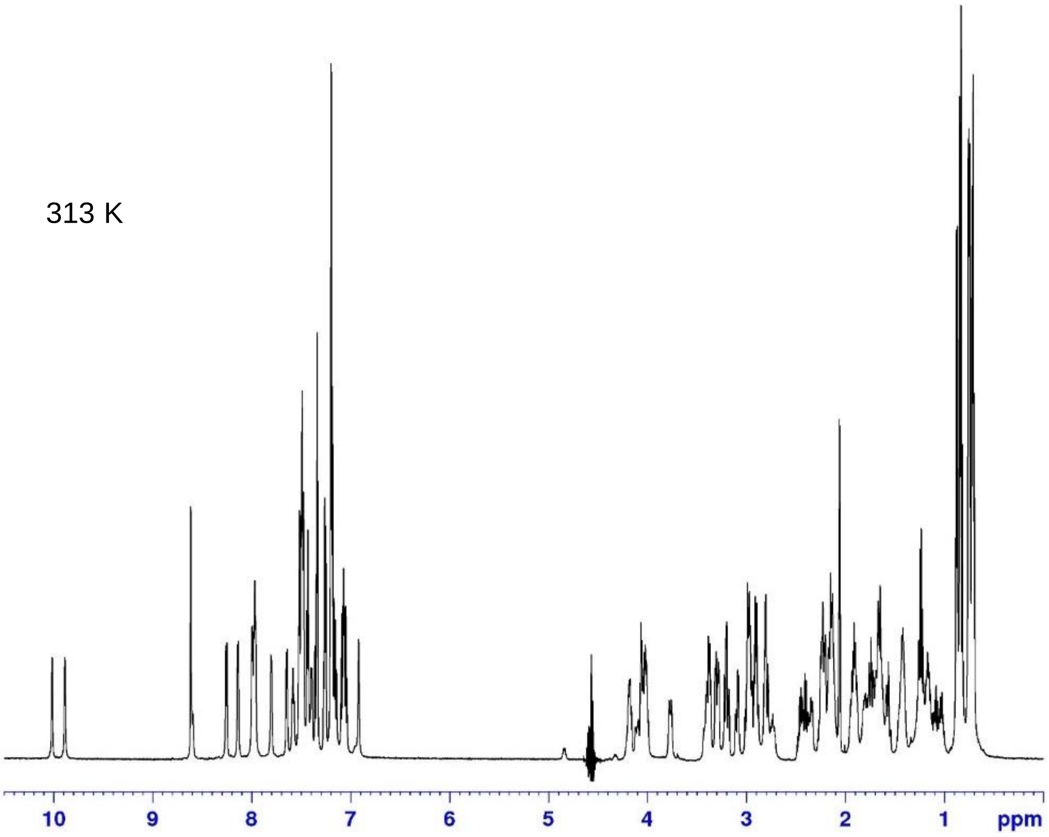

Peptide **3c** TOCSY 600 MHz

SB-VII-20 298K 2.2mg/500ul H2O/D2O/CD3CN 80/10/10 tocsyesgp

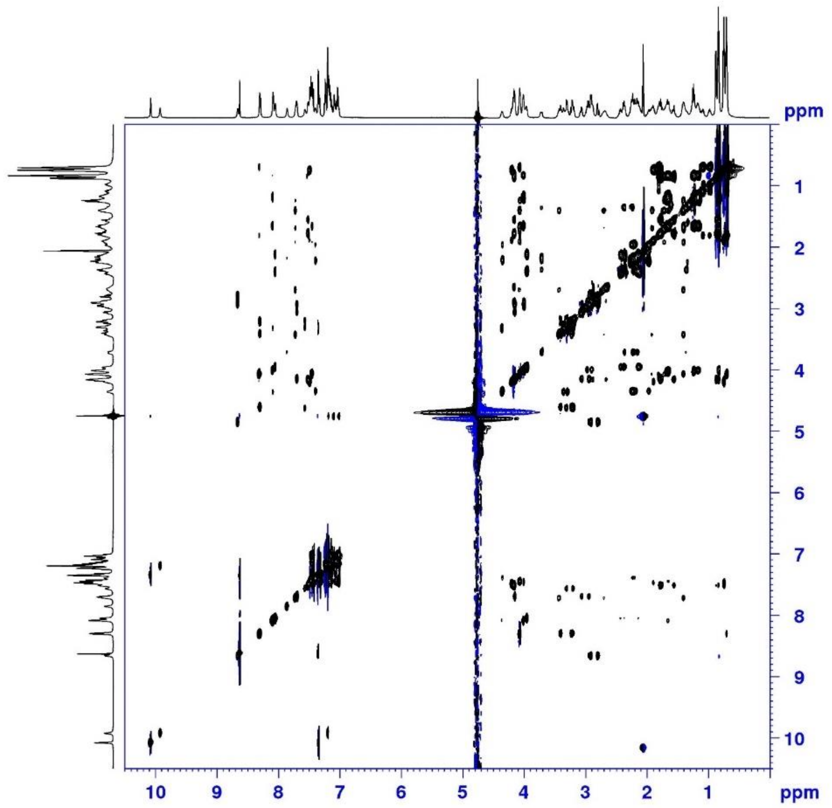

SB-VII-20 313K 2.2mg/500ul H2O/D2O/CD3CN 80/10/10 tocsyesgp

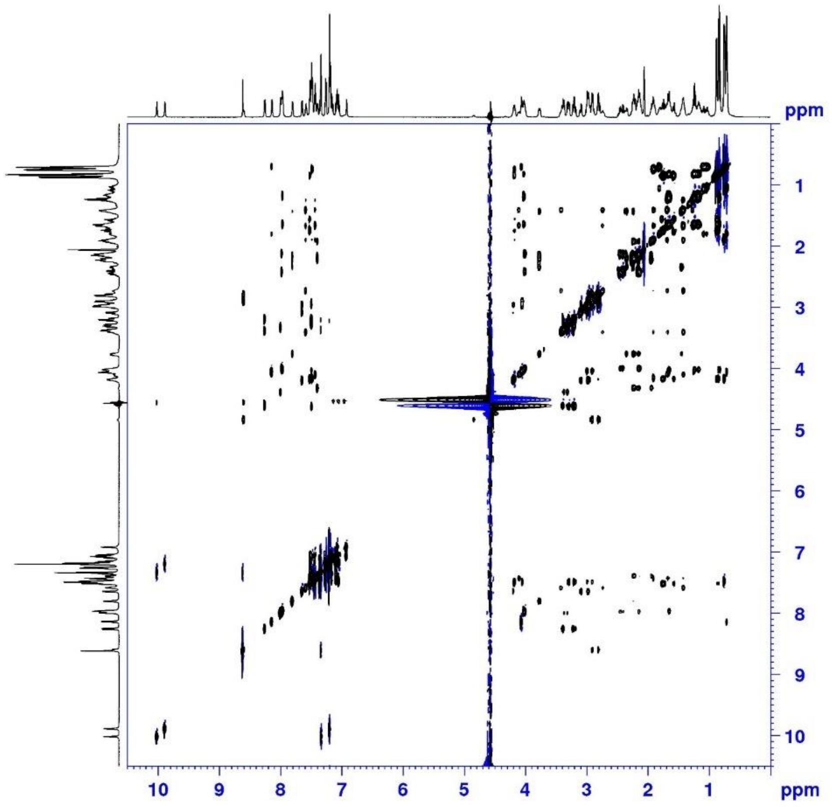

Peptide **3c** - TOCSY 600 MHz

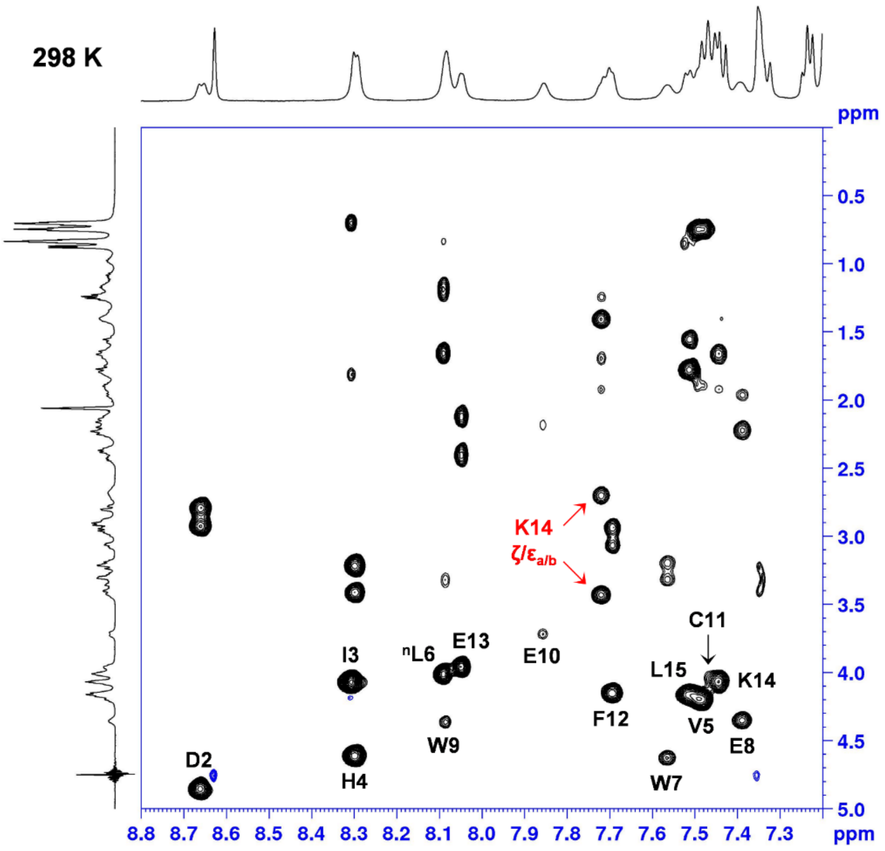

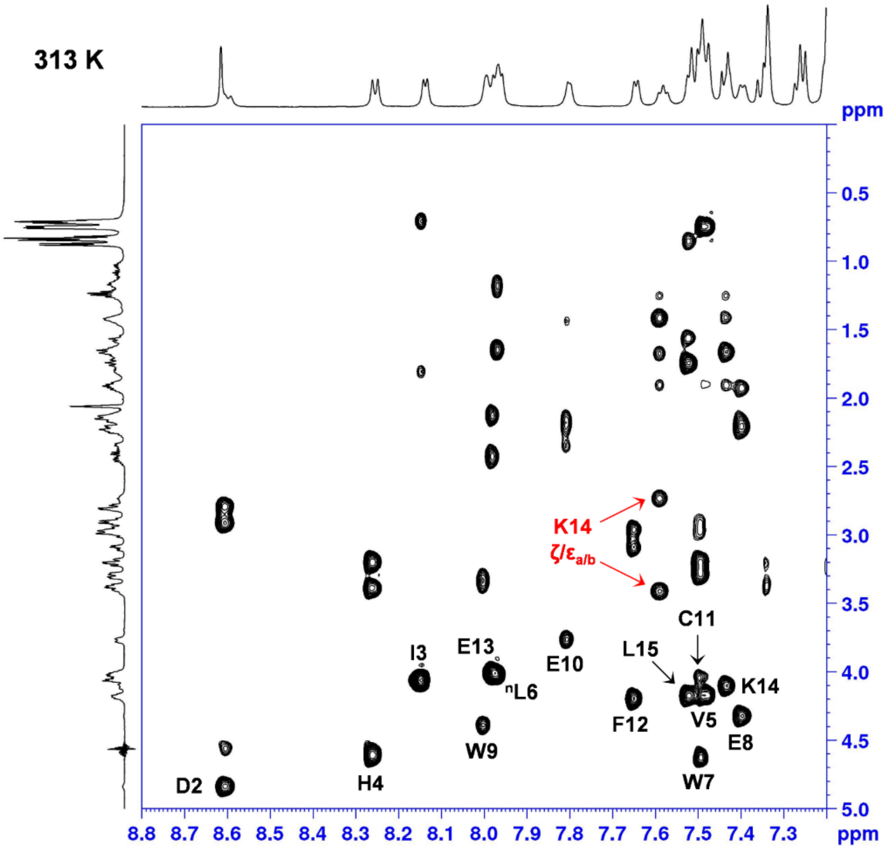

Peptide **3c** NOESY 600 MHz

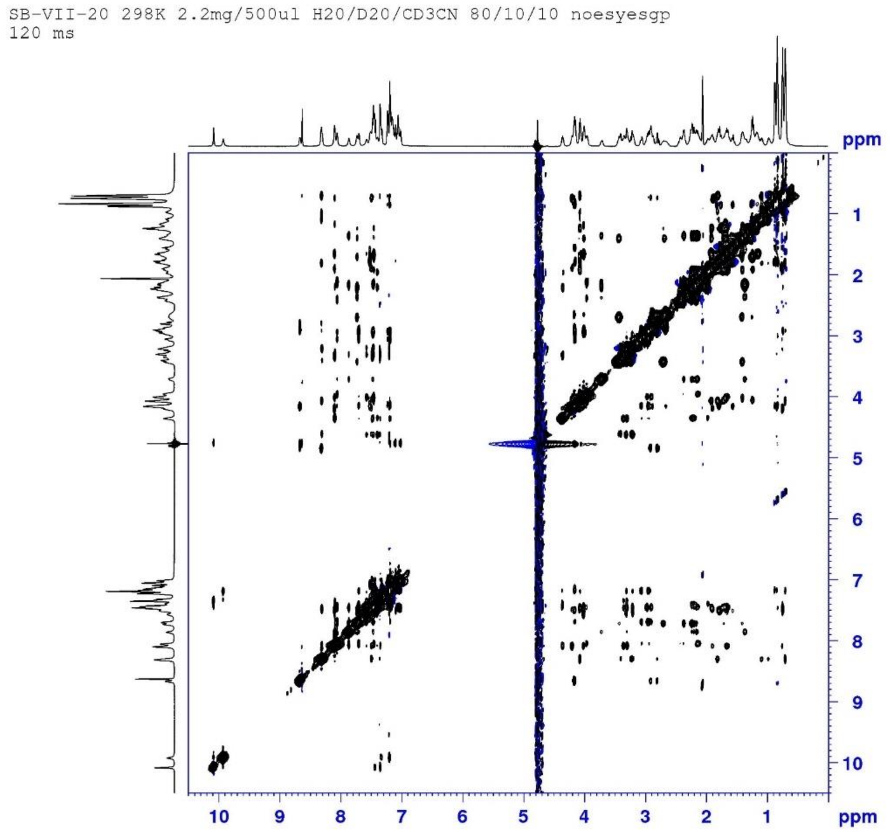

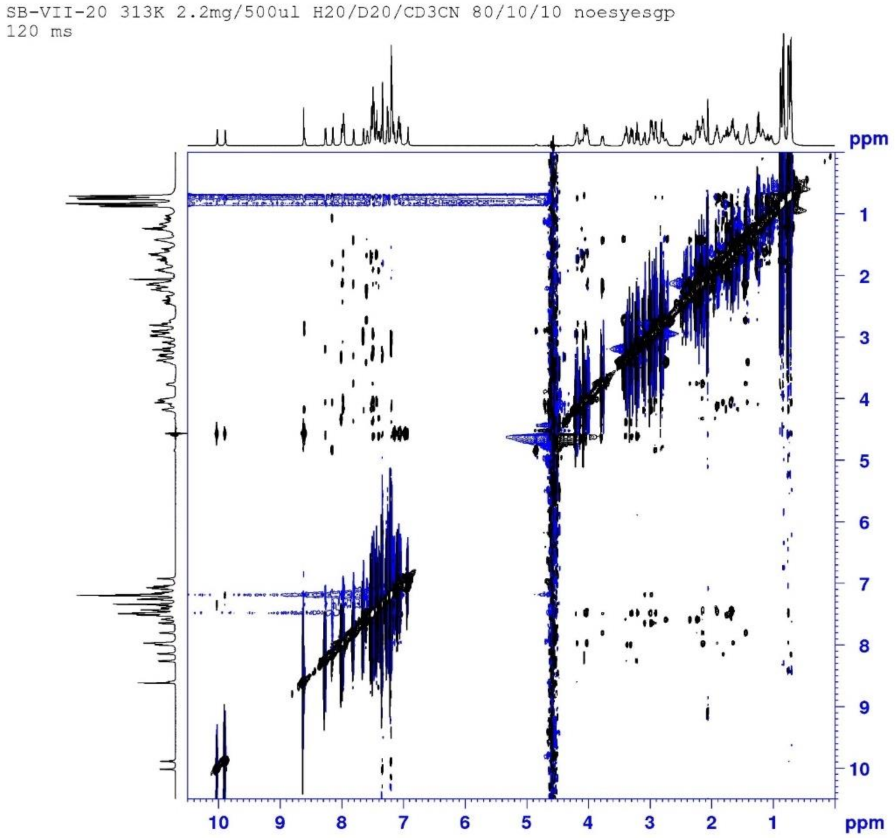

7.11.7 Peptide **4**

CDIHV^{n}LWEWKCFEEL-NH$_2$
SS

^{1}H 600 MHz

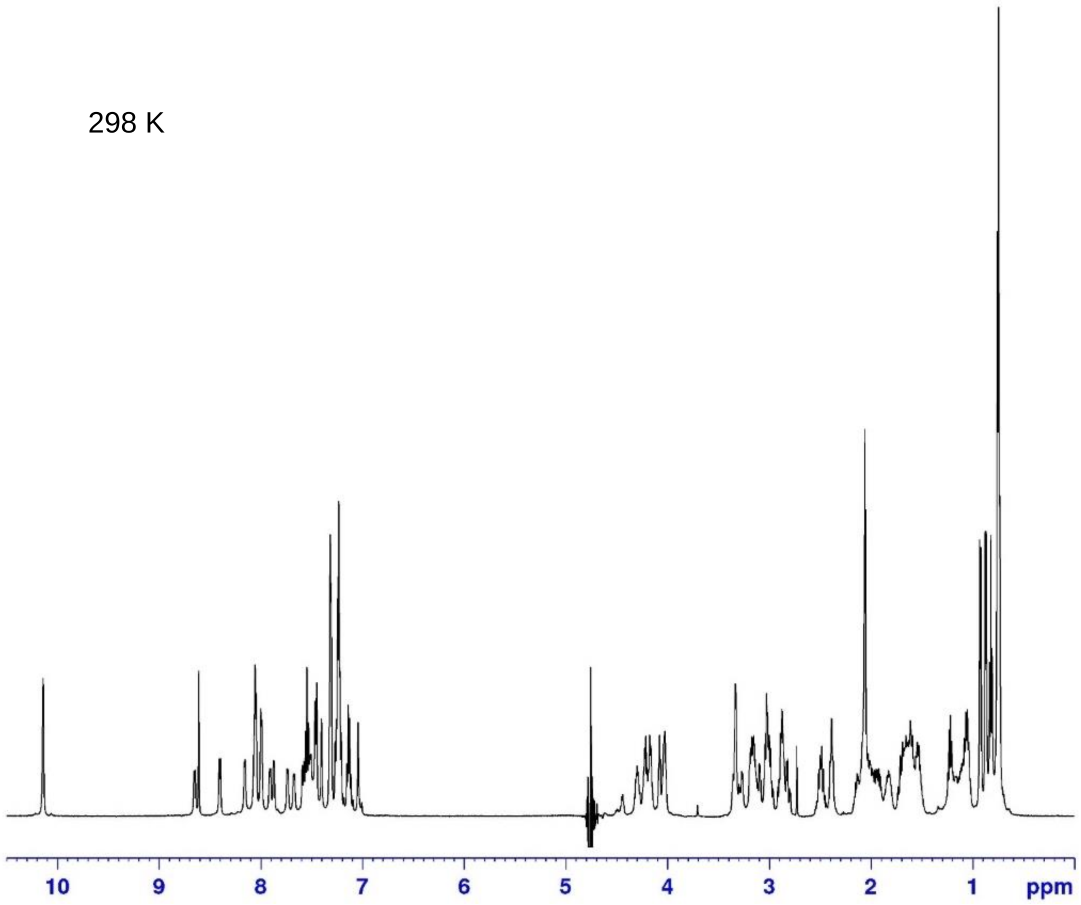

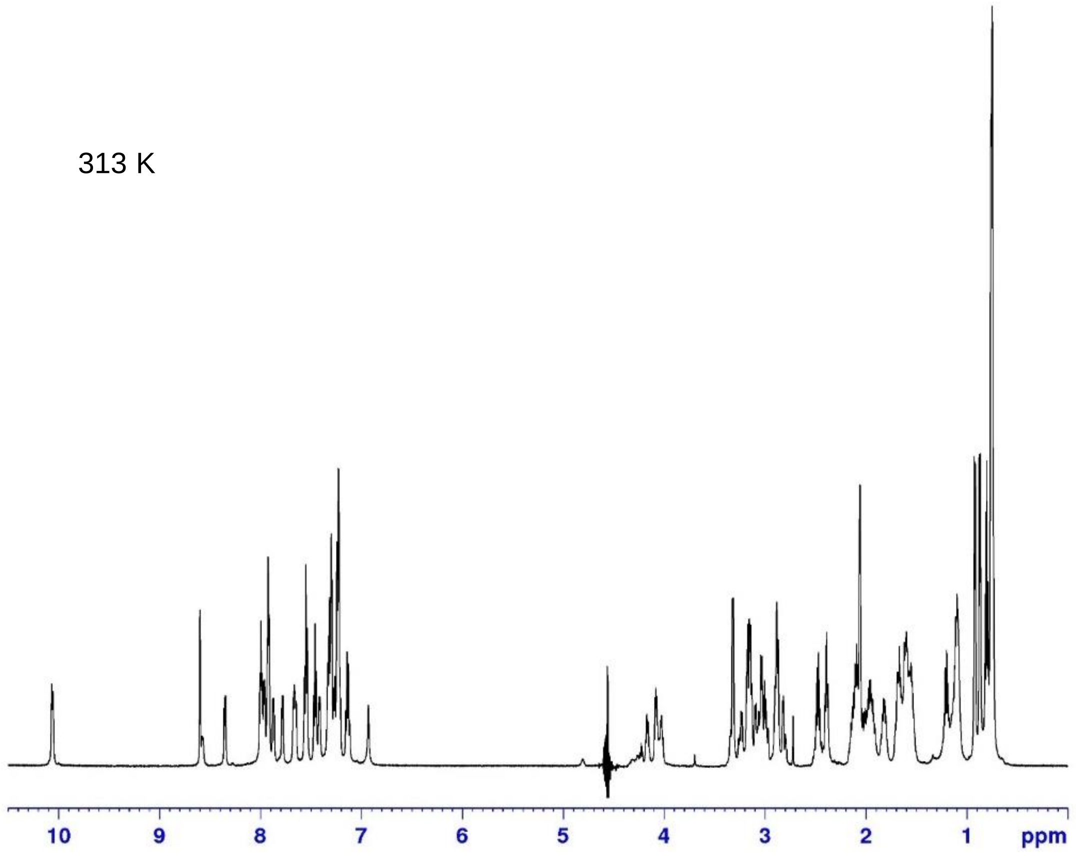

Peptide **4** - TOCSY 600 MHz

SB-VII-15 298K 2.4mg/500ul H20/D20/CD3CN 80/10/10 tocsyesgp

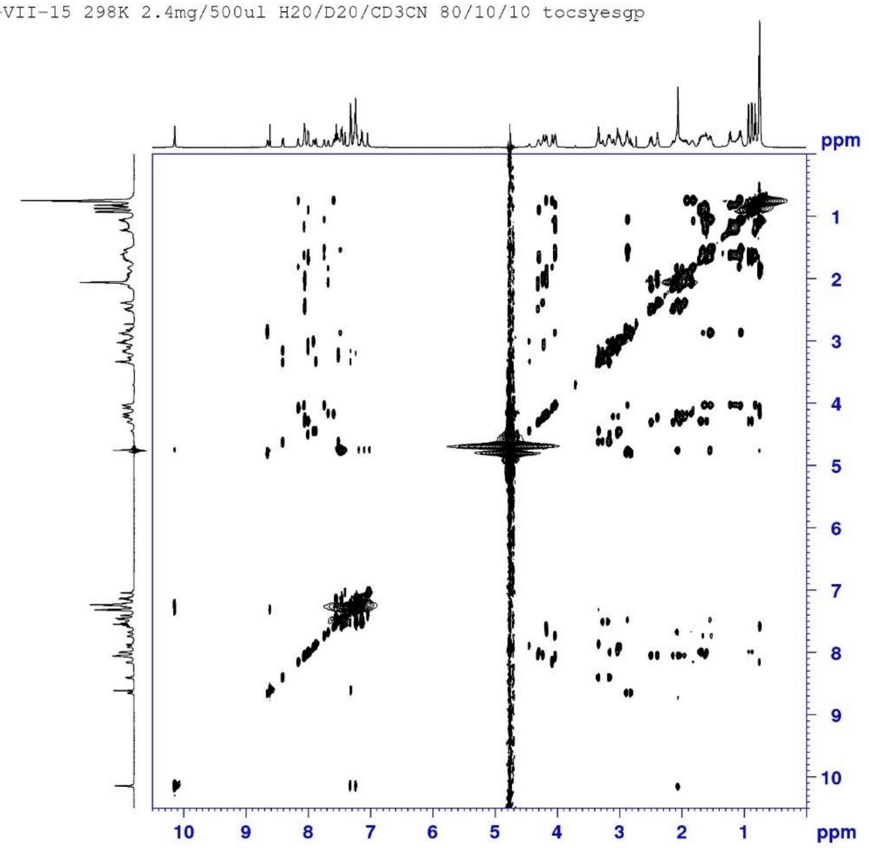

SB-VII-15 313K 2.4mg/500ul H20/D20/CD3CN 80/10/10 tocsyesgp

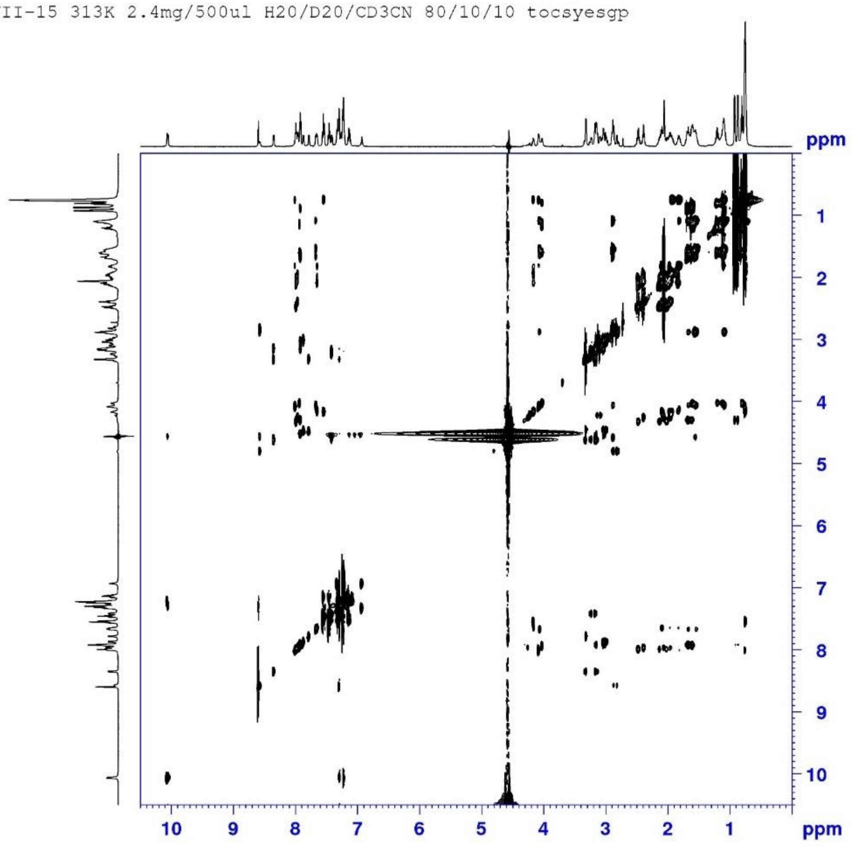

Peptide **4** - TOCSY 600 MHz

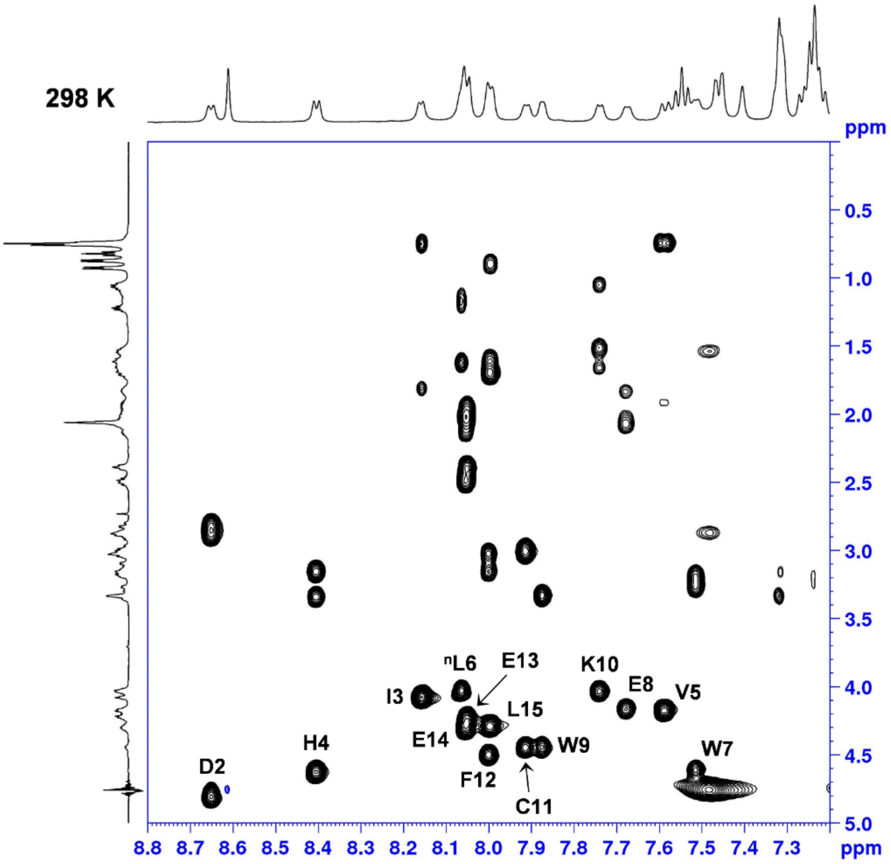

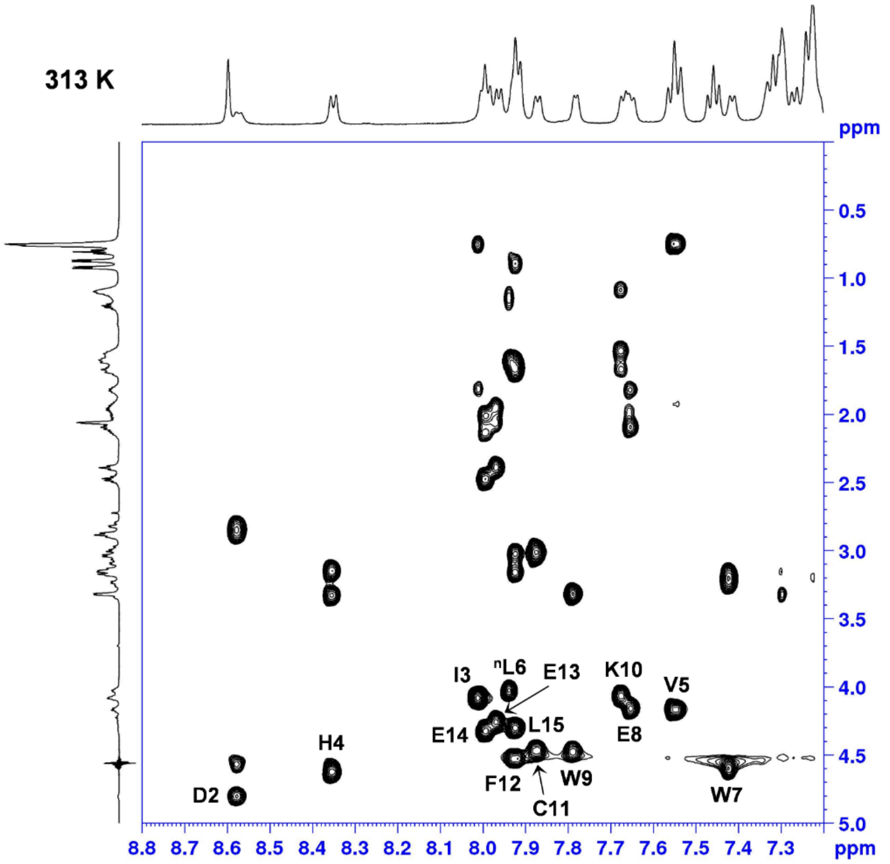

Peptide **4** NOESY 600 MHz

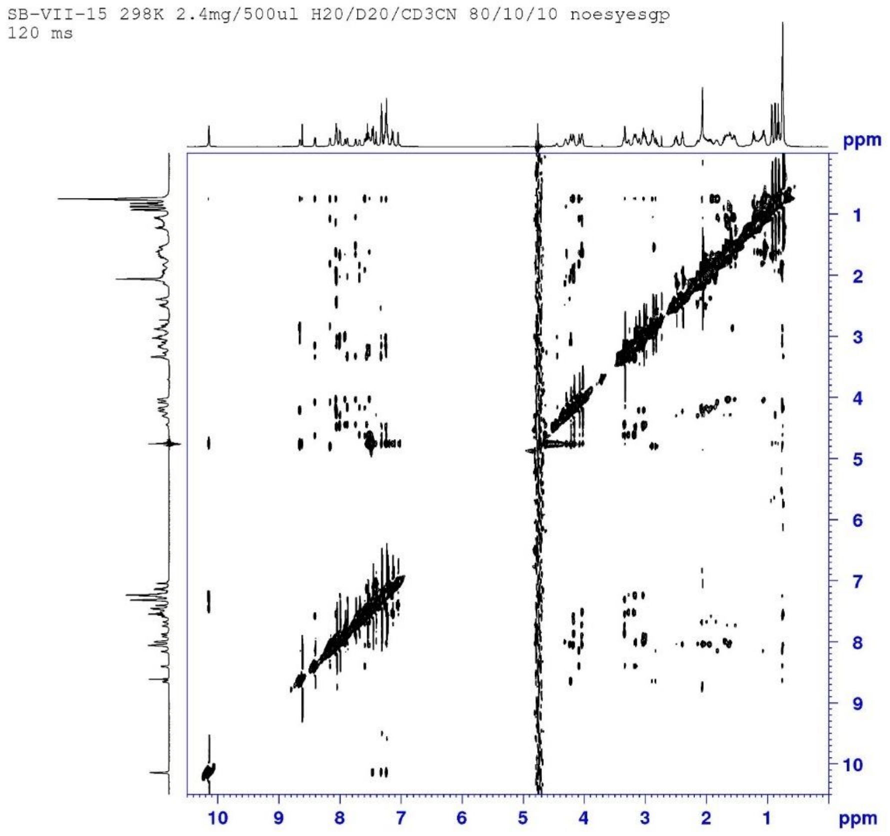

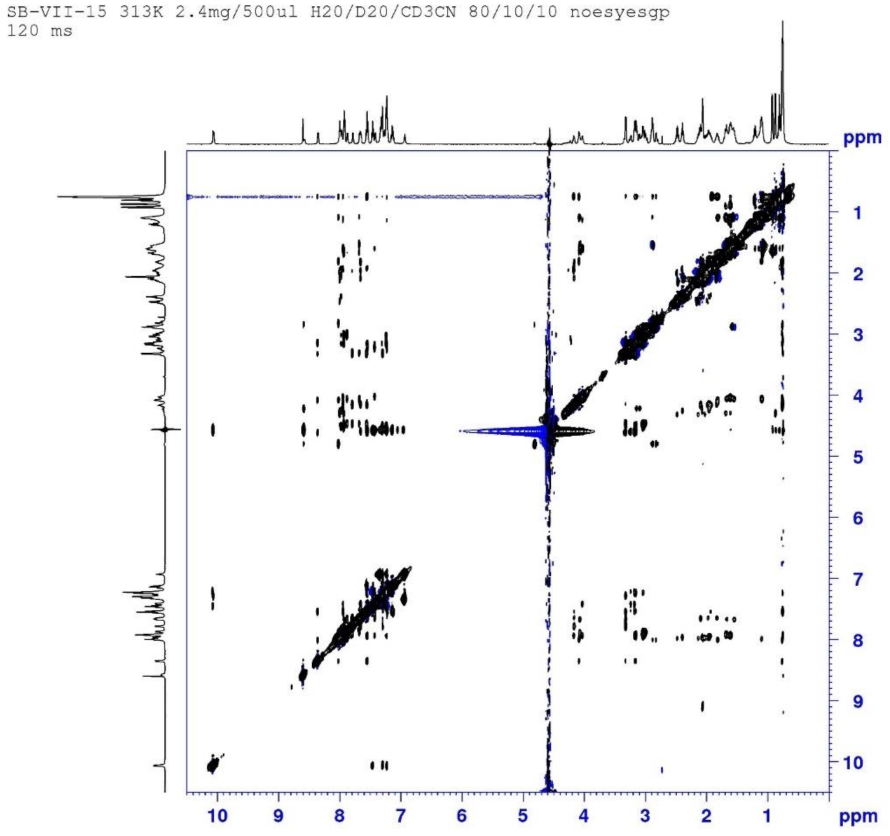

7.11.8 Peptide **4c**

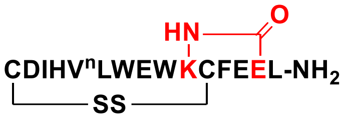

^{1}H 600 MHz

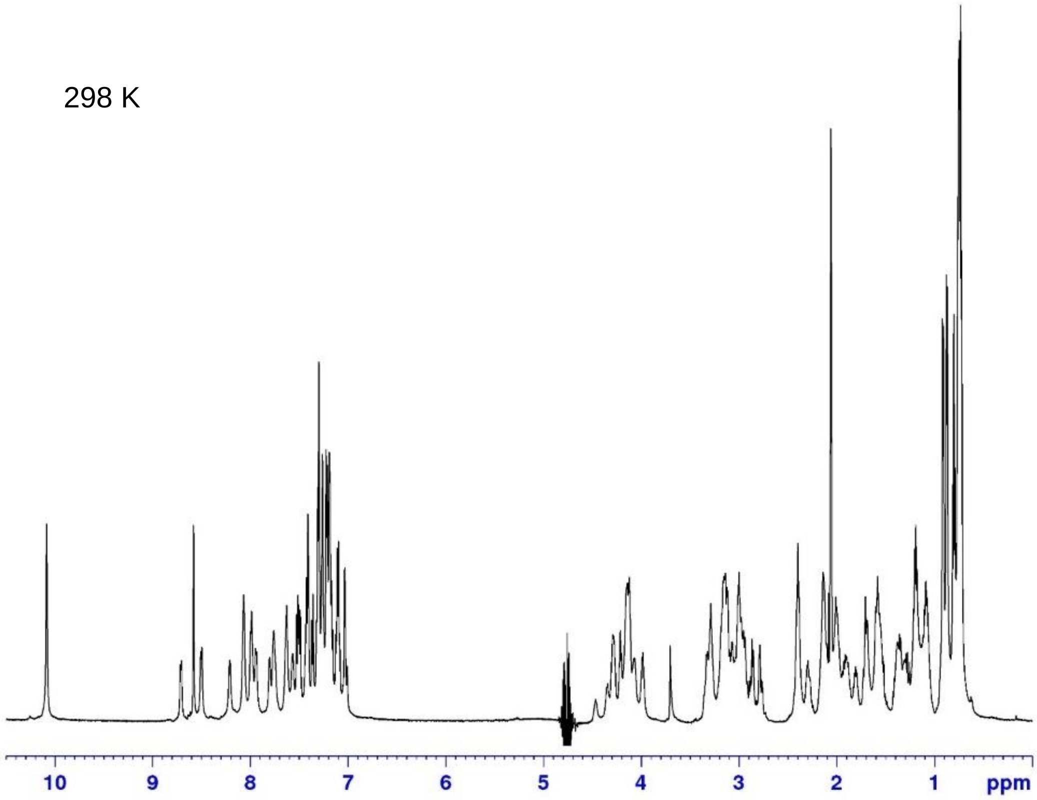

298 K

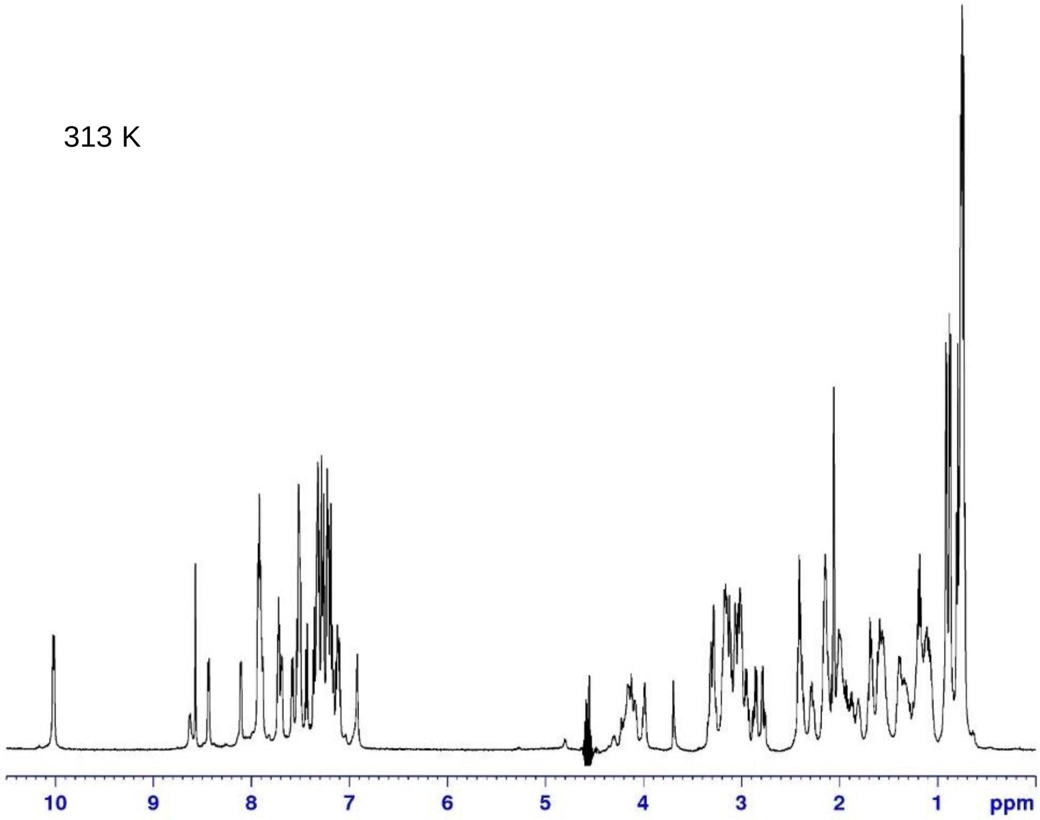

313 K

Peptide **4c** TOCSY 600 MHz

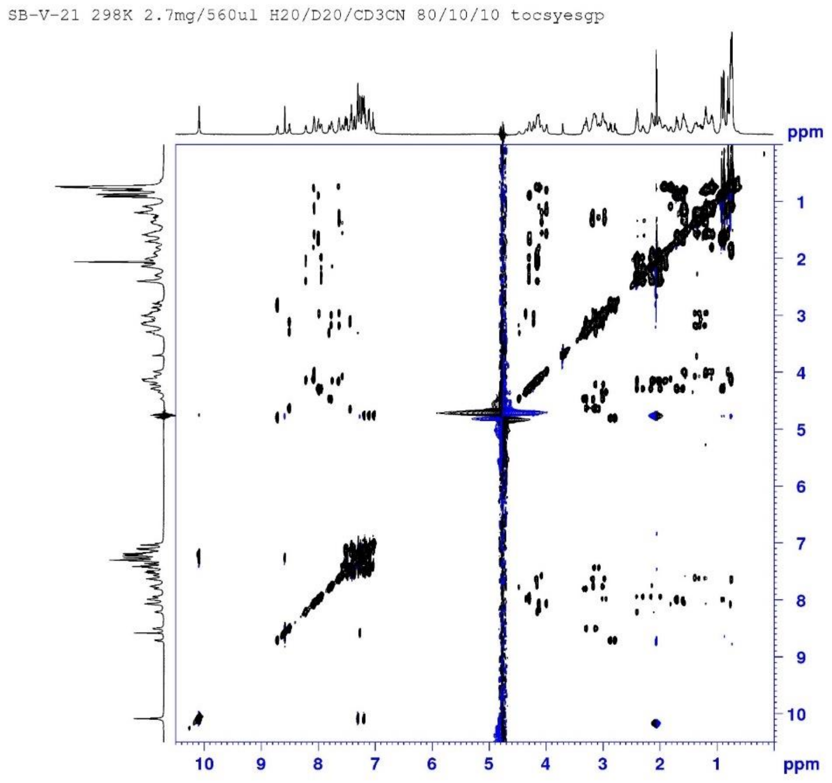

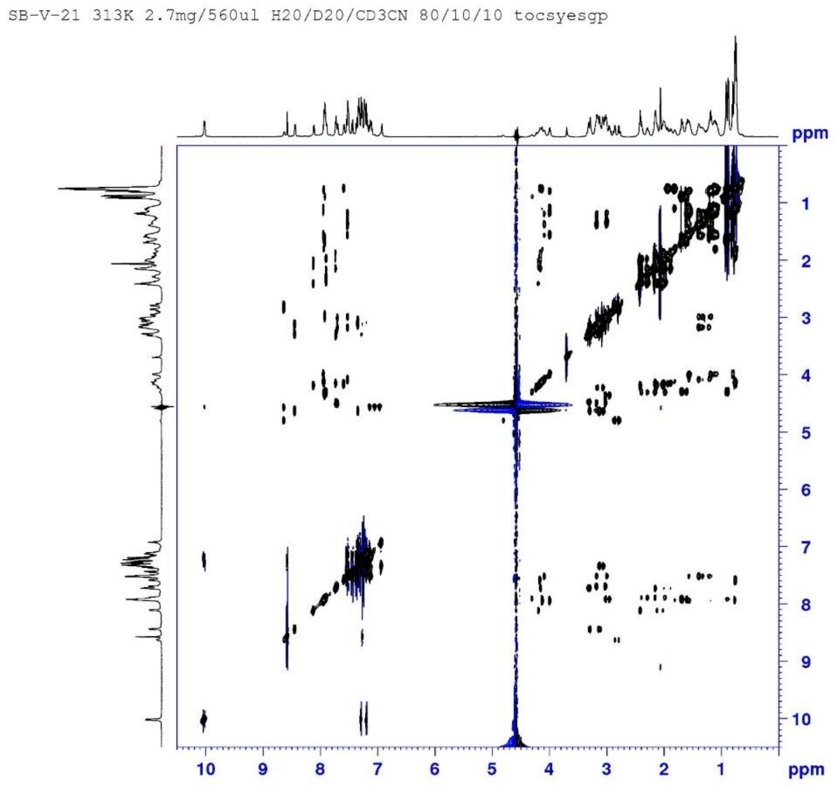

Peptide **4c** - TOCSY 600 MHz

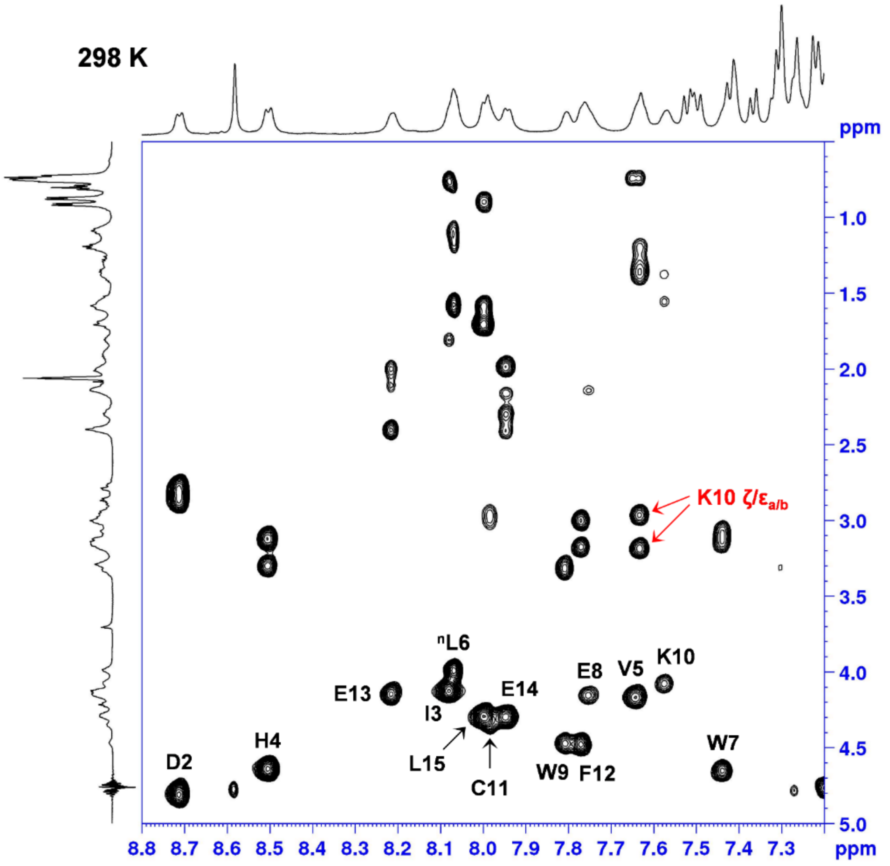

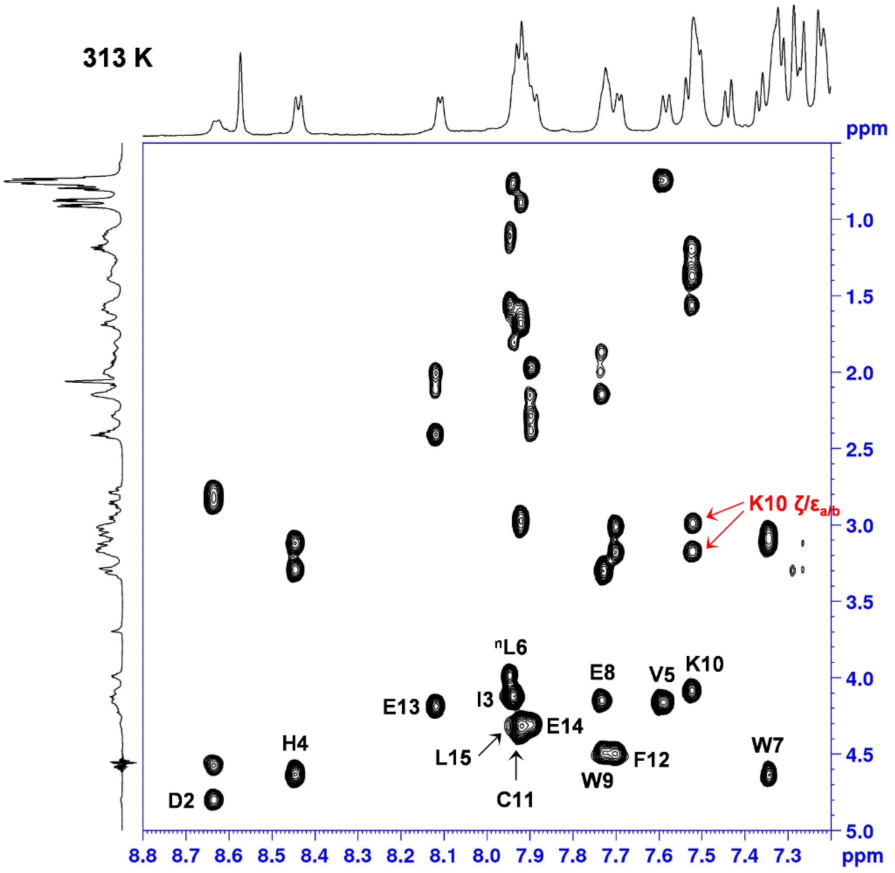

Peptide **4c** NOESY 600 MHz

SB-V-21 298K 2.7mg/560ul H20/D20/CD3CN 80/10/10 noesyesgp
120 ms

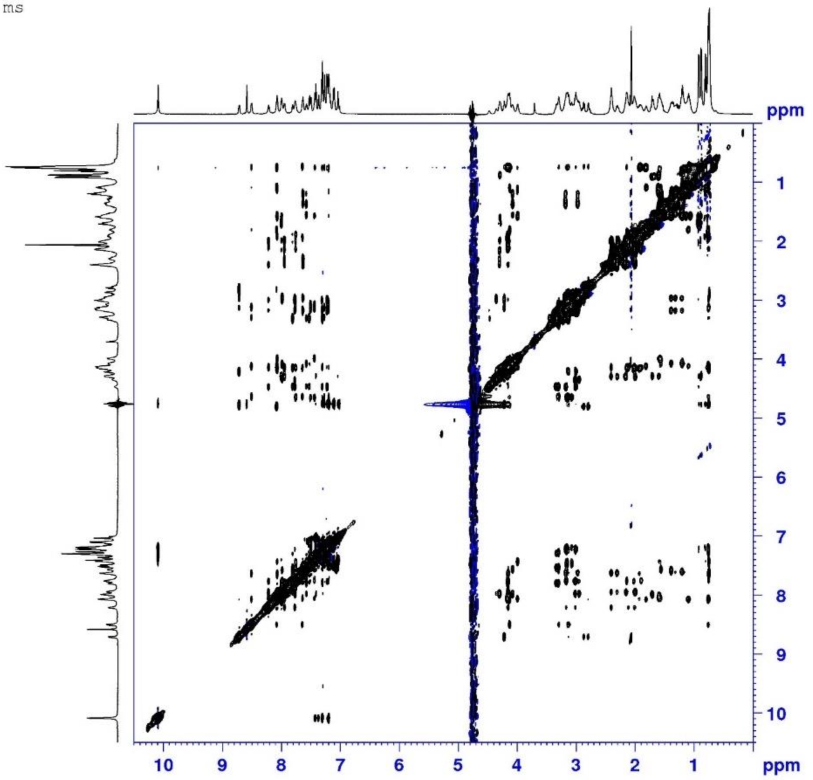

SB-V-21 313K 2.7mg/560ul H20/D20/CD3CN 80/10/10 noesyesgp
120 ms

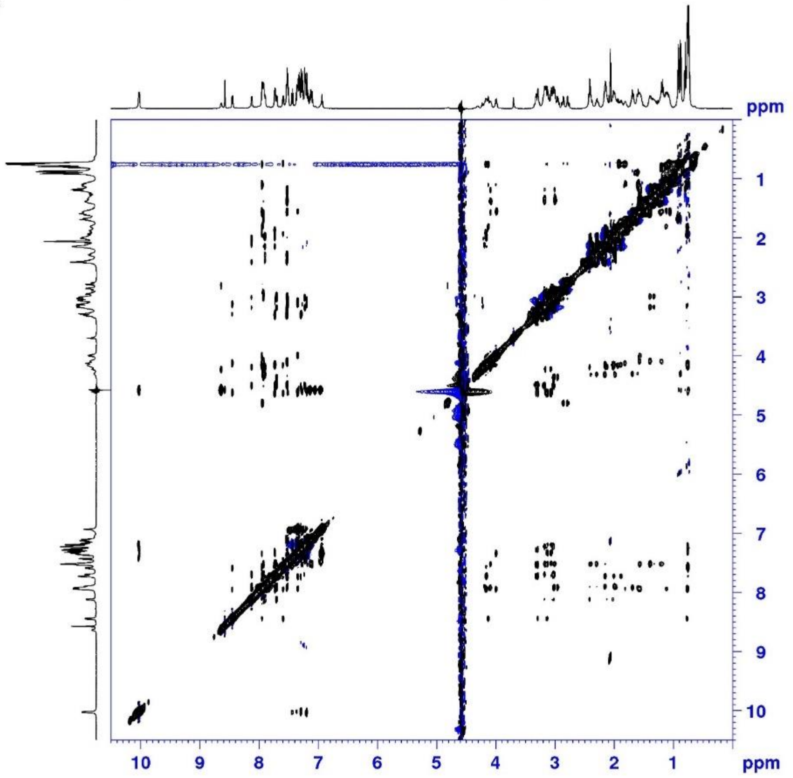

Supplement: Supplementary file 1 — Supporting Information [file CHEM-28-0-s001.pdf]
